# Stigma measurement in health: a systematic review

**DOI:** 10.1016/j.eclinm.2025.103360

**Published:** 2025-07-24

**Authors:** Sara Malone, Lara Counts, Luke Zabotka, Anneliese Williams, Nele Loecher, Kayla Wynja, Gemma Bryan, Robin Tanner, Ana Cáceres-Serrano, Gia Ferrara, Lucia Fuentes, Tharwa Bilbeisi, Marissa Maheu, Benjamin K. Oelkers, Muna Ogwo, Lauren Yaeger, Asya Agulnik, Dylan Graetz, RESPECT Team, Shayla Lawrence, Jocelyn Rivera, Farris Abutineh, Doris Maldonado

**Affiliations:** a School of Public Health, Washington University in St. Louis, St. Louis, MO, USA; b Department of Global Pediatric Medicine, St. Jude Children’s Research Hospital, Memphis, TN, USA; c Department of Public Health, Purdue University, West Lafayette, IN, USA; d Department of Psychology and Biobehavioral Sciences, St. Jude Children’s Research Hospital, Memphis, TN, USA; e University of Tennessee Health Science Center, Memphis, TN, USA; f Unidad Nacional de Oncología Pediátrica, Guatemala City, Guatemala; g Becker Medical Library, Washington University in St. Louis School of Medicine, St. Louis, MO, USA

**Keywords:** Stigma, Health, Discrimination, Measurement

## Abstract

**Background:**

Stigma experienced by individuals with disease is a barrier to health-seeking behaviors and outcomes. Our aim was to systematically review how stigma has been defined and measured and identify gaps in approaches to measurement and intervention.

**Methods:**

A systematic review was conducted following PRISMA guidelines. Databases were searched for stigma measurement in health through July 2024. 8123 citations were screened. Data on definitions, measurement, psychometrics, and interventions were extracted. PROSPERO: CRD42023433176.

**Findings:**

We identified 2267 studies (2605 tools) from 101 countries. 396 (15.2%) tools focused on development and 2369 (91.0%) applied tools. Most tools assessed adults (77.2%). Over 750 stigma tools were identified; many tools were adapted (n = 674) or shortened (n = 446). 117 studies reported effective interventions, primarily in adults. Key gaps included lack of consensus on definitions, limited pediatric-focused research, and insufficient attention to structural drivers of stigma.

**Interpretation:**

This review calls for standardized, context-sensitive stigma measurement and interventions applicable across conditions and settings. Addressing these gaps is crucial to reducing the global burden of stigma and enhancing health outcomes. Future research should focus on unified conceptual approaches and definitions to develop globally adaptable tools and scalable interventions that address both the experiences and structural drivers of stigma.

**Funding:**

Components for the programs involved in this work have been funded by 10.13039/100007737St. Jude Children's Research Hospital, the 10.13039/100000054National Cancer Institute (3POCA021765-44S2), the 10.13039/100012524American Lebanese Syrian Associated Charities, and 10.13039/100014571Siteman Cancer Center. The views in this paper are those of the authors and don’t necessarily reflect the funding agencies. The funding agencies were not involved in the writing or submitting of this work.

**Research in context**

**Evidence before this study**

Stigma has been inconsistently defined and measured in a myriad of ways, limiting comparative analyses and comprehensive interventions. Tools to measure stigma vary in their conceptual foundations, rigor, adaptability, and potential applicability across different populations and contexts. To advance quality of life and improve outcomes for those with illness, it is imperative to unify our understanding of stigma. This systematic review searched Embase 1947-, Ovid Medline 1946-, Scopus 1823-, Cochrane Central Register of Controlled Trials (CENTRAL), Cochrane Database of Systematic Reviews (CDSR), Cumulated Index to Nursing and Allied Health Literature (CINAHL Plus) 1937-, APA PsycInfo 1800s-, Global Health 1973-, and Clinicaltrials.gov 1997- to explore how studies have measured stigma of individuals with health conditions and their caregivers.

**Added value of this study**

This systematic review provides a comprehensive review of individual stigma measurement. Stigma has been studied in 101 countries, and over 750 unique tools have been used to measure stigma in research settings. However, definitions of stigma are not commonly adopted, and the concept of stigma has been conceptualized inconsistently. This study highlights the lack of unified measurement tools, making it impossible to characterize both unified assessments and specific characteristics of the experience of stigma. Additionally, the study identifies notable gaps in the study of stigma, particularly in pediatric settings and in evidence-based interventions.

**Implications of all the available evidence**

This systematic review offers an overview of how we conceptualize and measure stigma in health conditions globally. There remains a need for unified measurement on individual stigma that cuts across cultural, contextual, and health system boundaries. This study will inform future research agendas, measurement, and intervention on individual stigma experiences for patients as well as their caregivers.

## Introduction

Globally, stigma remains a pervasive barrier to health and wellbeing,^1^, ^2^, ^3^ with long-term consequences that include impacts on health-seeking behaviors (e.g., delays in care, treatment abandonment) and health outcomes (e.g., disease transmission, reduced quality of life).^4^, ^5^, ^6^, ^7^, ^8^, ^9^, ^10^ Stigma has been conceptualized as the discrimination individuals face due to a specific disease, or the negative association between a group of people who share certain characteristics and a specific diagnosis, and involves components of both internal perception and external experiences.^11^, ^12^, ^13^ These impacts are exacerbated by intersecting and compounding stigma experiences, for example, those related to poverty,^14^ age,^15^ disabilities,^16^ and cultural differences.^17^ To address these gaps and ultimately intervene on stigma, we need a comprehensive understanding of how stigma manifests across diverse contexts, conditions, and populations. However, current approaches to studying stigma–including definitions, conceptual models, measurement tools, and interventions—have been siloed, limiting our ability to develop shared frameworks and tools as well as our capability to compare findings across studies. Highly individualized stigma strategies have resulted in marginal progress within certain diseases (e.g., mental health) but have contributed to non-unified research that cannot answer questions across settings and conditions, generating a barrier to effective and scalable strategies to reduce stigma.^18^^,^^19^

In addition to novel tool development, many studies have adapted tools that assess persons affected by stigma of various health conditions; however, is unclear if they remain valid and applicable once adapted across contexts, health conditions, and age groups.^20^ Barriers to using stigma tools across contexts include logistical barriers such as language translations as well as conceptual barriers, meaning stigma might be understood differently across diseases or settings.^21^ This limits our ability to draw comparisons, identify shared mechanisms, and develop effective transferable interventions to reduce stigma.^22^ A more unified approach is essential to reduce the burden of stigma and improve health outcomes worldwide.

For this systematic review, we sought to examine how stigma has been defined, conceptualized, and measured across health conditions globally to identify gaps and similarities that can be used to unify the approach to measuring stigma. We have focused on the stigma of the individuals impacted by disease (patients and caregivers) rather than community or healthcare professional stigma to capture those most proximally affected. Our review includes a synthesis of definitions, which entail key components of stigma, and interventions to reduce stigma that have been tested across health conditions. We aimed to answer the following questions: (1) How has stigma been conceptualized and measured across health conditions globally? And (2) What tools are used to measure stigma across health conditions? By systematically reviewing and evaluating the psychometric properties, applicability, and potential adaptability of stigma measurement tools, this review aims to provide a foundation for more robust and equitable approaches to measuring and addressing stigma across diverse health conditions and settings. This knowledge is essential to advance our understanding of stigma, enable the study of stigma in different cultures or regions, develop targeted interventions to reduce stigma, and ultimately inform global initiatives and policy efforts.

## Methods

This systematic review was registered in PROSPERO (CRD42023433176), an international registry for systematic review protocols, and performed according to the PRISMA guidelines. A medical librarian (LY) searched the literature for records including concepts of persons affected by stigma, self-stigma, and scales, instruments, measurements, or outcomes. The librarian (LY) created search strategies using keywords and controlled vocabulary in Embase 1947-, Ovid Medline 1946-, Scopus 1823-, Cochrane Central Register of Controlled Trials (CENTRAL), Cochrane Database of Systematic Reviews (CDSR), Cumulated Index to Nursing and Allied Health Literature (CINAHL Plus) 1937-, APA PsycInfo 1800s-, Global Health 1973-, and Clinicaltrials.gov 1997-. All search strategies were completed and run on 12/28/2022, with no added limits, and 14,663 results were returned. Duplicate records (N = 6532) were deleted after using the de-duplication processes described in “De-duplication of database search results for systematic reviews in EndNote.” Covidence.org, a website used to manage systematic review projects,^23^ removed 8 additional duplicates resulting in a total of 8123 publications moved to screening. Fully reproducible search strategies for each database can be found in Appendix A. All searches were updated on 7/26/2024 by rerunning the search from inception to 7/26/2024, resulting in 2304 new citations after deduplication in Covidence.

Papers were included (Appendix A) if they reported stigma measurement in a health setting, including either behavioral or physical health, and used a quantitative tool. For this review, we accepted stigma as labeled by the authors and did not use set established definitions or criteria. Stigma measurement consisted of either a complete tool or a single domain of another measure (i.e., patient-reported quality of life). Papers were required to be full-length, peer-reviewed, and could include the measurement of stigma of patients or immediate caregivers. There were no restrictions on the study type, including papers of all designs (cross-sectional, longitudinal, randomized trials, etc.). Papers could be written in English, Spanish, or Arabic, as these are languages spoken by our research team. Papers were excluded if they were abstracts, conference proceedings, conceptual papers, dissertations, governmental reports, or review papers. For this review, we considered adapted or shortened tools as unique from those in their original form.

Abstract and full text screening were conducted in two sequential rounds. Covidence was used to screen the abstract and full text of each article. Two reviewers, out of a group of 15 fully trained reviewers, independently screened article titles and abstracts (round 1) and full texts (round 2). If conflicting reviews occurred during either round, a third reviewer independently adjudicated. All reviewers were trained in inclusion/exclusion criteria and had expertise in pediatric oncology, global health, and/or stigma. Study data were collected and managed using REDCap (Research Electronic Data Capture) tools hosted at Washington University in St. Louis.^24^^,^^25^ All full-text articles included in the screening process were independently extracted by two reviewers in REDCap. A third party reviewed and resolved all discrepancies.

### Data analysis

For all included studies, reviewers extracted the following information: 1) WHO region and country where the study was conducted; 2) the health condition of which stigma was being studied; 3) operationalization and definition of stigma; 4) type of stigma measured and approach (e.g., using patients directly or collecting proxy reports from a caregiver or other individual); 5) age of the population; 6) how stigma was measured within the tool; and 7) information about the tool, including the language of administration and the number of items and domains within the tool. Our extraction and analysis categorized studies into two categories: 1) those that developed and/or psychometrically tested a tool, and 2) those that applied a stigma tool in a study. Reviewers extracted additional data from studies that developed stigma measures and/or conducted psychometric analyses to identify tool development methods and describe how psychometric testing was conducted. We assessed adaptations and instances of shortening the tools within papers that utilized previously existing tools and reviewed all tool development papers for specifications on reliability and validity analyses. Studies that applied stigma measurement were analyzed for sample size, study design, and interventions used to address stigma.

We used narrative synthesis to answer our study questions regarding measurement approaches, applications, and other aspects of stigma measurement.^26^ Due to the diversity in paper types and size of sample, we were unable to conduct meta-analyses as well as formal quality appraisals. We did complete subanalyses to help answer the primary study questions. Because of our interest in the measurement of stigma globally, we analyzed the data with attention to regional differences and country income levels—allowing us to examine how stigma has been conceptualized and studied in various contexts. Additionally, we explored how stigma has been measured across age groups, specifically looking for differences in pediatric and adult stigma assessment.

### Role of funding source

The funder of this study had no role in the study design, data collection, data analysis, data interpretation, or writing of the report.

## Results

**Fig. 1:**
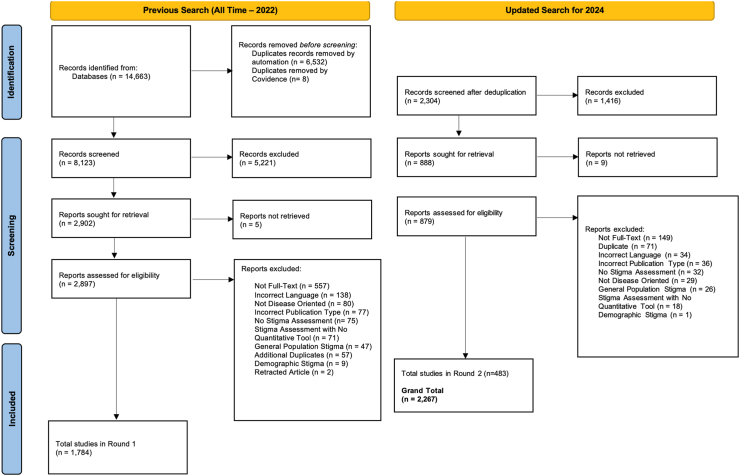
PRISMA diagram for the review.

In the initial round, we included 2897 papers for full-text review (Appendix B), ultimately including 1784 studies that met our inclusion criteria (Fig. 1—PRISMA diagram). During the updated search, another 879 papers were included for full-text review and an additional 483 met our inclusion criteria. In total, across both searches, 2267 studies met our inclusion criteria and were extracted (Table 1). Within some studies, multiple tools were used to measure stigma—in these instances, information was pulled for each tool that was used in the study separately. Ultimately, 2605 tools were extracted from the 2267 studies. Studies were noted as development and/or application, but these categories were not mutually exclusive—149 papers both developed and applied tools, resulting in their inclusion in both categories.

**Table 1:** Study characteristics stratified by development or application classification (N = 2267 papers).

	Tool development papers N = 363 (16.0%)^a^	Tool application papers N = 2080 (91.8%)^a^
**Population of interest**		
Adult (18+)	273 (75.2%)	1608 (77.3%)
Pediatric (<18)	10 (2.8%)	63 (3.0%)
Both	44 (12.1%)	195 (9.4%)
Not Specified	40 (11.0%)	225 (10.8%)
**Respondent**		
Patient stigma	354 (97.5%)	2000 (96.2%)
Patient proxy	6 (1.7%)	35 (1.7%)
Caregiver stigma	21 (5.8%)	110 (5.3%)
**Total participants**		
0–100	**–**	556 (26.8%)
101–250	**–**	782 (37.6%)
251–500	**–**	421 (20.2%)
501–1000	**–**	181 (8.7%)
1001–2000	**–**	95 (4.6%)
2001+	**–**	43 (2.1%)
**Number of stigma tools in the study**		
1	288 (79.3%)	1826 (87.8%)
2	62 (17.1%)	211 (10.1%)
3+	13 (3.6%)	42 (2.0%)
**Health condition**		
Physical	272 (75.0%)	1428 (68.7%)
Behavioral	95 (26.2%)	692 (33.3%)
**WHO region**		
African	45 (12.4%)	298 (14.3%)
Region of Americas	113 (31.1%)	514 (24.7%)
South-East Asia	28 (7.7%)	208 (10.0%)
European	97 (26.7%)	482 (23.2%)
Eastern Mediterranean	26 (7.2%)	113 (5.4%)
Western Pacific	62 (17.1%)	430 (20.7%)
Not specified	14 (3.9%)	101 (4.9%)
**Income level**^b^		
High income	188 (51.8%)	938 (45.1%)
Upper middle income	89 (24.5%)	538 (25.9%)
Lower middle income	41 (11.3%)	312 (15.0%)
Low income	12 (3.3%)	130 (6.2%)

a Note: the percentages do not add to 100% because some papers both developed and applied tools, resulting in their inclusion in both categories. Also, some categories had multiple options, resulting in papers fitting more than one category (for example, a study taking place in more than one WHO region, income level) and being included. Therefore, the numbers within each demographic can add up to more than the total number of papers.

b Income levels exclude nations that were unspecified in the publication or took place as part of a multinational collaboration.

**Fig. 2:**
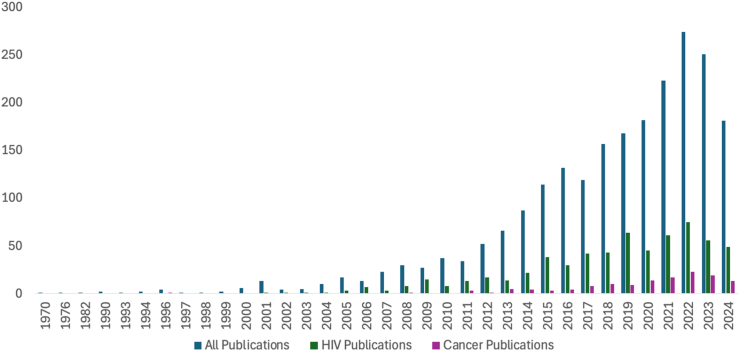
Quantity of publications per year for all publications, HIV stigma publications, and cancer stigma publications.

**Fig. 3:**
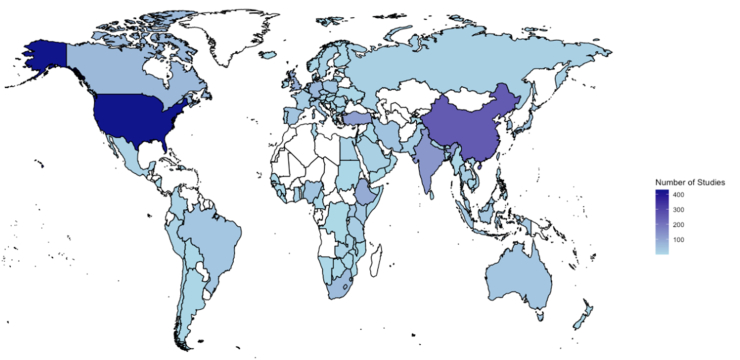
World map displaying the number of included studies by country. The shading corresponds to the number of studies originating from each country.

More studies were published in 2022 (274, 12.1%) than any other year. The number of publications per year rapidly increased over time (Fig. 2). HIV (human immunodeficiency virus) was the most commonly studied disease across all years, and the number of publications related to stigma in HIV and cancer rose dramatically beginning in 2010. Publications restricted to only assessing cancer stigma rose from a total of 2 documented publications in 2009 to 135 in 2024, while papers restricted to only assessing HIV/AIDS stigma rose from went from 40 to 617. Studies were conducted in 101 unique countries with <20% from any one country. Most work was performed in single locations, and very few papers (n = 72; 3.2%) were published using data or work across multiple countries. There were studies from every World Health Organization (WHO) Region and across all national income levels. When grouping the studies into WHO regions, most studies were conducted in the Americas (n = 566, 25.0%), followed by the European Region (n = 537, 23.7%) and the Western Pacific Region (n = 467, 20.6%) (Fig. 3). Studies were primarily developed and carried out in high-income countries (n = 1,029, 45.4%), with the number of studies decreasing along with World Bank Income category (Table 1). Commonly used languages for the tools were English (n = 476; 18.3%), Standard Chinese (n = 240, 9.2%), and Spanish (n = 114, 4.4%). Over 250 tools were described as being adapted by translation for use in specific local languages. This process was not always described, leaving the procedures and rigor of translation processes unclear.

### Defining stigma

Stigma definitions were included in 1019 (44.9%) studies. The use of definitions was not stratified by region or specific disease type within the review. The most common definition used was Goffman’s; 131 (5.8%) papers used this definition or referred to Goffman’s conceptualization when they introduced stigma. Goffman’s definition of stigma, included in these manuscripts, is ‘the situation of the individual who is disqualified from full social acceptance due to a trait which is deeply discrediting'.^27^ Other common definitions included Corrigan (n = 45, 2%) and Link and Phelan (n = 65, 3%). Corrigan, whose work builds on Goffman’s, described stigma as ‘a set of negative attitudes, beliefs, thoughts and behaviors that affect one person or the public and cause fear, rejection and discriminatory behavior.’^28^ Link and Phelan expanded this definition to say that stigma is the co-occurrence of ‘labeling, stereotyping, separation, status loss, and discrimination'.^12^ Further, there was a lack of consistency in the use of similar definitions by region or diagnosis (Table 2). Very few studies (n = 15, <1%) reference the World Health Organization conceptualization of stigma, which is as ‘a mark of shame, disgrace or disapproval which results in an individual being rejected, discriminated against, and excluded from participating in a number of different areas of society’.^29^ When conceptualizing stigma, papers frequently described the experienced stigma specifically associated with the disease of interest rather than referring to stigma as a social phenomenon defined across diseases or settings. Aside from strict definitions, authors commonly referred to stigma both as a social process as well as a psychosocial challenge.

**Table 2:** Frequently used stigma definitions and their regions with frequently observed disorders.

Region and Condition	Corrigan	Goffman	Link and Phelan	Total
**African region**	**1**	**12**	**5**	**18**
Epilepsy	0	1	0	1
HIV/AIDS	0	10	5	15
Schizophrenia	1	1	0	2
**American region**	**10**	**23**	**17**	**50**
Bipolar disorder	3	1	2	6
Depression	4	2	3	9
Epilepsy	0	1	2	3
HIV/AIDS	0	18	7	25
Schizophrenia	3	1	3	7
**Eastern Med. region**	**5**	**4**	**2**	**11**
Bipolar disorder	2	0	0	2
Depression	1	0	0	1
Epilepsy	0	1	0	1
HIV/AIDS	0	1	0	1
Schizophrenia	2	2	2	6
**European region**	**11**	**16**	**9**	**36**
Bipolar disorder	2	2	0	4
Depression	3	5	2	10
Epilepsy	0	2	3	5
HIV/AIDS	0	4	1	5
Schizophrenia	6	3	3	12
**Southeast Asia region**	**3**	**12**	**9**	**24**
Bipolar disorder	1	1	1	3
Depression	1	4	4	9
Epilepsy	0	0	1	1
HIV/AIDS	0	4	0	4
Schizophrenia	1	3	3	7
**Western Pacific region**	**13**	**11**	**11**	**35**
Bipolar disorder	3	0	2	5
Depression	4	3	3	10
Epilepsy	0	1	1	2
HIV/AIDS	0	4	2	6
Schizophrenia	6	3	3	12
**Total**	**43**	**78**	**53**	**174**

Tabulations with an unspecified region were excluded from the table. Additionally, these conditions were selected based on the most frequently reported condition globally, rather than by region.

Studies used different conceptual approaches to assess stigma. Some authors solely assessed stigma, whereas others assessed stigma as a component of a larger outcome of interest, such as quality of life. For tools that solely measured stigma, including some studies that used more than one tool (n = 2,283, 87.6%), there were often different components of stigma operationalized as separate domains within the tool. Some studies defined these domains of stigma, including internalized stigma, associative stigma, and anticipated stigma. However, not all studies measured these domains as distinct within their tools, rather the different stigma conceptualizations were grouped. Across all tools used to measure stigma, the median number of domains was 4, ranging between 0 and 11 on any given instrument. The 55.1% of studies that did not define stigma referenced it as related to the condition of interest or generically described the impact of stigma, or its manifestations, such as poor mental health or associated health outcomes.^30^^,^^31^

### Population of interest

**Fig. 4:**
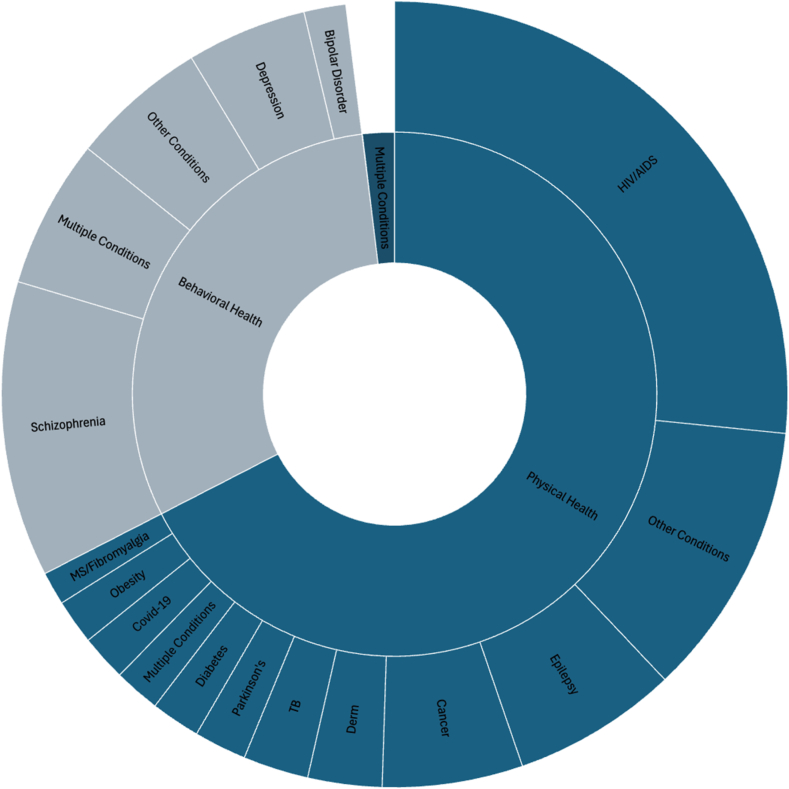
Study frequencies across various disease domains. Abbreviations: MS (Multiple Sclerosis), TB (Tuberculosis), Derm (Dermatological conditions), HIV/AIDS (Human Immunodeficiency Virus/Acquired Immunodeficiency Virus).

We assessed the condition studied at the study level, with some papers including both physical and behavioral health diagnoses (n = 43, 1.9%). More than half of the publications assessed the stigma related to a physical condition: 1573 (69.4%). Within the category of physical condition, 632 (40.2%) focused on HIV/AIDS (Fig. 4). Other diseases included epilepsy (175, 11.1%), cancer (144, 9.2%), and Parkinson’s (56, 3.6%). Of the behavioral health studies (737, 32.5%), the majority focused on schizophrenia (276, 37.5%), depression (112, 15.2%) and bipolar disorder (39, 5.3%). 416 papers in both physical and mental health (14%) assessed more than one disease, assessing some component of the intersection of the two diseases. Most assessments of stigma, given that some studies used multiple assessments within one study, were focused on the measurement of the patient experience of stigma (n = 2,491, 95.6%). Very few assessments used proxy measures (35, 1.3%) or assessed caregiver stigma as a separate construct (129, 5%).

Most tools used focused on exclusively adult populations 2012 (77.2%), with only 79 (3.0%) tools deploying or developing a stigma tool on a strictly pediatric population. An additional 235 (9.0%) tools included both pediatric and adult populations with a focus on the adolescent and young adult population (ages 10–24).

### Measuring stigma

Over 750 unique tools were utilized to measure stigma. The tools utilized ranged from 1 to 64 items, with a median of 16 items. Some (n = 34) tools self-described as developing or validating tools but lacked both reliability and validity testing. As previously mentioned, most tools focused specifically on stigma (n = 2,283, 87.6%). However, 253 (9.7%) tools assessed a more global or broad outcome, such as patient wellbeing, and included at least one domain that measured stigma. The rest of the tools (n = 69, 2.6%) did not specify how they were measuring stigma throughout their tool use. The most common response option included using Likert scales to assess the extent of stigma experienced.

#### Measurement development

Of the studies presented, 363 (16%) reported developing or validating a measure of stigma somewhere in their work, with the development of 396 (15.2%) of tools. This included studies that created a new tool, tested a tool within a new setting, or conducted psychometric work to describe the reliability or validity of a previously developed tool. In development papers, reliability was most often presented (n = 318, 87.6%). This was followed by construct validity (n = 255, 70.2%), which was either described or highlighted by correlating the analysis to another valid measure. There were also studies where individuals reported developing or using a novel tool for their study and calculated Cronbach’s alpha before analyzing the collected data. Many tools were developed for use within each disease of interest (Table 3). Table 4 highlights the most commonly used tools within our review and information about their development and use.

**Table 3:** Overview of instruments by health condition of interest.

Health Condition	Number of tools	Development/validation	Tested for reliability	Tested for construct validity	Shortened	Adapted
**Physical health**	1764	291	247	199	42	42
Human immunodeficiency virus/acquired immunodeficiency syndrome	773	93	75	52	18	16
Epilepsy	200	27	23	21	4	2
Irritable bowel syndrome/disease	20	6	5	5	2	2
Parkinsons	58	7	6	6	2	0
Dementia	18	4	4	4	0	0
Leprosy	31	3	2	1	1	1
Obesity	50	12	10	8	1	2
Dermatological conditions	101	18	15	16	3	3
Tuberculosis	71	14	12	9	2	4
Covid-19	47	15	12	10	0	4
Hepatitis C	17	2	1	1	0	0
MS/Fibromyalgia	37	10	10	8	4	1
Cancer	150	39	37	33	4	2
Type 1 diabetes mellitus	32	9	9	9	0	1
Type 2 diabetes mellitus	55	12	12	12	1	1
Asthma/chronic obstructive pulmonary disease	14	2	2	2	0	0
Sickle cell anemia	7	1	1	0	0	0
Other	261	62	54	44	9	7
**Behavioral health**	885	105	96	77	22	10
Schizophrenia	457	43	40	36	11	4
Depression	280	40	38	28	11	4
Anxiety	134	23	21	16	6	2
Post traumatic stress disorder	46	10	10	6	2	0
Personality disorder	66	12	12	9	3	0
Bipolar disorder	218	21	19	15	6	2
Eating disorder	19	5	4	4	0	0
Autism spectrum disorder	10	1	1	0	0	0
Attention deficit hyperactivity disorder/attention deficit disorder	22	4	4	3	0	0
Alcohol use disorder	78	15	11	12	1	1
Other substance use disorder	131	23	19	15	3	1
Other intellectual disorder	8	2	2	1	0	0
Other behavioral	384	58	54	45	12	4

The table represents a total number of tools per disease, including repeated tools. Development/Validation represents all tools utilized in a development/validation study. The columns tested for reliability, construct validity, adapted, and shortened include tools utilized in development/validation studies only.

**Table 4:** Most used measures and properties of their development.

#### Measurement application

Most studies (n = 2,080, 91.8%) applied an existing stigma measure to a specific health population, even if they also conducted some aspect of measure development in their work. 2369 (91%) tools were applied in these papers. In these studies, the most frequently used scales were the original forms of the: HIV Stigma Scale (n = 247), Internalized Stigma of Mental Illness Inventory (n = 218), and Stigma Scale for Chronic Illness (n = 74). These tools were also frequently adapted and shortened for use.

Within measurement application studies, many tools were adapted for use in different populations, diseases, and settings. Adaptations included shortening of tools, select use of specific domains/subscales, validated adaptations, and non-validated adaptations. The most common adaptations were to the HIV Stigma Scale (122 adapted versions) and the Internalized Stigma of Mental Illness Scale (108 adapted versions). Each of these tools has been adapted and applied in other health conditions. Many studies shortened previously validated measures to apply them to new settings. Only some of the studies in which tools were shortened conducted new psychometric analyses prior to use. Taking both approaches into account, a total of 418 (18.4%) papers adapted existing measures with 60 (2.6%) studies shortened existing tools. Within these studies, tool-level adaptations were reported as necessary for application in new settings or different diseases (n = 674, 28.4%). Most often, adaptations included translating the tool for a specific context or editing the tool to change the disease it referenced. For example, the Epilepsy Stigma Scale is adapted from the Stigma Scale for Chronic Illness and was subsequently validated and used on individuals with epilepsy.^32^ Commonly studied diseases provided a unique opportunity to understand how many tools have been created and used to study stigma within one condition. For example, we examined the measures for HIV and epilepsy since they were the most common diseases analyzed (reported above). Excluding unnamed measures, considering modifications and diverse naming mechanisms, and including adapted versions of previously validated tools, there were 466 tools for stigma related to HIV and 134 for epilepsy, respectively.

### Stigma interventions

This review identified 208 (9.2%) papers describing patient-level interventions targeting stigma across adult (164), pediatric (6), mixed adult and pediatric (17), and unspecified (21) populations. Implemented interventions included psychosocial (65), psychoeducation (47), pharmacological (10), surgical (10), not described (5) care models (3), procedural (2), or other (6), with definitions found in Appendix C. Some studies (60) included combinations of interventions. Interventions were implemented across a multitude of physical and behavioral health conditions (e.g., cancer, HIV, diabetes, and depression) and occurred in various healthcare and community settings. These interventions were led by researchers, clinicians, organizations, and/or peers. Usually, stigma was measured using tests pre- and post-interventions. Post-intervention evaluations had a large range in timing, with some evaluations immediately after interventions to others up to six years post-intervention delivery.

Of these 208 intervention papers, 117 reported effectiveness at reducing stigma in adult (96), mixed (5), pediatric (3), and unspecified (13) populations. The majority of effective interventions were psychosocial (31), psychoeducational (21) or both psychosocial and psychoeducational (30), implemented within a clinical setting (78) and by a research team (23). Effective interventions were equally split between individual delivery (58), group delivery (43), both (6). Described interventions were largely used only in one study, although a few evidence-based care programs were described repeatedly, including the Step up! SOS program and the Honest, Open, Proud program.^33^^,^^34^

## Discussion

This systematic review synthesizes the conceptualization and measurement of stigma amongst persons affected by health conditions, ranging across diverse health conditions and settings, drawing on 2267 studies from 101 countries and including patient-level interventions. Our findings reveal key patterns, gaps, and next steps necessary for advancing stigma research and policy. Studies from all WHO regions and income levels demonstrate that stigma is a universal experience. However, the field remains fragmented due to disparate definitions and a proliferation of measurement tools across contexts. This fragmentation limits our ability to compare findings, generalize results, and develop effective cross-cutting interventions. To address these challenges, it is necessary to align stigma conceptualization, measurement, and intervention development, with particular attention to context, culture, language, and the unique needs of specific populations, such as children.

Prior systematic reviews of stigma of individuals have largely focused on a particular disease type or geographic region. These reviews provide deep insight into populations of interest, however, they may have also propagated siloed work. Uniquely, our review highlights the stigma as a shared human experience, regardless of disease or culture.^35^^,^^36^ Measuring stigma globally, using the shared experience approach, allows for (1) empirical assessment of cross-cultural experience of stigma and (2) identification of required adaptations to detect nuance in the different experiences of stigma across disease or culture.

A central finding of this review is the lack of consensus in defining and conceptualizing stigma. Even within specific regions or diseases, different definitions were utilized. While frameworks such as those by Goffman and Corrigan have been most widely used, they are not consistently adopted or integrated across all studies of stigma. Instead, many studies employed unique definitions tailored to specific diseases or populations. Moving forward, the field should work toward a unified conceptual framework that captures the multidimensional nature of stigma—including its drivers, dimensions, and impacts—while allowing for contextual and cultural adaptations. Given that stigma definitions were included in less than half of the papers in the sample, a simple yet critical recommendation is for future studies to explicitly state their definitions and conceptual frameworks, including the specific stigma domains or dimensions measured.

The lack of a unified definition of stigma has led to the creation of numerous measurement tools, with over 750 unique instruments identified in this review across different regions and disease types. While stigma is often viewed as context-dependent, this does not fully explain the proliferation of tools within similar cultural, geographic, and population contexts (e.g., for the same disease type). This absence of standardization poses significant challenges for cross-cultural research and comparisons of stigma within and across health conditions. This has also resulted in duplicative and siloed work in areas where rigorous measures exist. Additionally, there are numerous studies employing non-validated tools, potentially resulting in findings that are not appropriately understood or contextualized. Although some tools, like the HIV Stigma Scale, have gained widespread adoption, many others have been developed or adapted for specific populations without sufficient psychometric validation. Nearly a quarter of studies reported adapting tools in some way, which can hinder their utility and the ability to compare stigma prevalence. Future work should prioritize developing tools that balance localization with the ability to facilitate meaningful comparisons across contexts. While adapting tools to local contexts is important, it often conflicts with the need for standardized, validated measures that can be applied across diverse settings. Moving forward, efforts should focus on first using tools that are already applicable and validated. If more tools for stigma are needed, tool developers should focus on creating multilingual, multidimensional tools that are applicable across diseases and resource levels. When this is done, rigorous translation methods should be used to maintain construct validity.^37^ These efforts will ensure that stigma measurement can appropriately guide intervention development.

An additional gap identified in this review is the lack of stigma research in pediatric populations, with only 3.1% of studies focusing on children. Stigma manifests uniquely in pediatrics, impacting health, development, and well-being of both patients and caregivers in ways that are not yet fully understood.^38^ Without tailored tools and interventions, we remain unable to address the long-term consequences of stigma in this vulnerable population. Future research must prioritize the development of pediatric-specific stigma measures and interventions, ensuring they are integrated into health systems to support improved health outcomes. Additionally, we identified gaps in the study of stigma associated with specific disease types, such as rare diseases or other infectious diseases beyond HIV. Further, there are many regions where stigma is still understudied.

While there is robust literature describing the occurrence of health-related stigma, our review identified a paucity of studies evaluating interventions to reduce stigma (only 208 or 9%). Most of these interventions addressed disease-specific stigma, lacked scalability, and focused on addressing the consequences of stigma rather than targeting its root causes, such as structural biases and discriminatory policies.^39^ Future work should be conducted to more deeply review this literature and assess priorities for intervening and addressing individual stigma. This review highlights that interventions should address the experiences, impacts, and drivers of individual stigma, with a focus on integrating intervention efforts into health systems to ensure sustainability and scalability.

While this review provides a comprehensive overview of individual stigma across health conditions and settings, it is limited by including only English, Spanish, and Arabic studies. This limitation, however, likely minimally impacted our findings as less than 10% (100 of the 1310) excluded full text studies were due to language. The heterogeneity of studies precluded a meta-analysis as well as formal quality appraisal, underscoring the need for greater alignment in stigma conceptualization and measurement. Future research should aim to achieve this alignment and conduct formal quality appraisals, enabling meta-analyses that can provide a clearer understanding of stigma prevalence and its impact on intervention effectiveness, patient care, and policy. Additionally, future work should capture and study aspects of public stigma that fall outside the scope of this review. Other measures, studies, and interventions should investigate these additional levels of stigma, as they all relate to the health and well-being of individuals and populations.

In conclusion, this review highlights an urgent need for alignment in stigma research, including conceptualization, measurement and intervention. To advance this field, we must promote standardized, contextually sensitive assessment tools and interventions focused on vulnerable and understudied populations. Additionally, stigma research must integrate efforts into health systems and practice to understand and address the pervasive effects of stigma on health outcomes. This work requires a coordinated, multidisciplinary approach that bridges research, practice, and policy gaps, ultimately improving care for stigmatized populations.

## Contributors

SM and DG conceptualized this study and led all aspects. SM assisted with data collection, data analysis, and led manuscript drafting. LC, LZ, AW, NL, KW, GB, RT, ACS, GF, LF, TB, MM, BO, MO all contributed to data extraction. LC, LZ, KW contributed to resolving conflicts, data management, and data analysis. AA contributed to data review and manuscript drafting. LY conducted the search and contributed to the design and conceptualization. SL, JR, FA, and DM are included in group authorship and participated in early data collection. All authors have participated according to ICMJE criteria. All authors had access to the data and verified the results in the study. All authors have revised the manuscript, reviewed the work, and were responsible for the decision to submit the manuscript.

## Data sharing statement

Data was collected for this study. Data and data dictionaries will be made upon request. The datasets presented in this article can be obtained via requests directed to Dr. Sara Malone (sara.malone@wustl.edu).

## Editor note

The Lancet Group takes a neutral position with respect to territorial claims in published maps and institutional affiliations.

## Declaration of interests

Individuals involved in this work (SM, DG, AA, BO) have been funded by St. Jude Children’s Research Hospital, the National Cancer Institute (3POCA021765-44S2), the American Lebanese Syrian Associated Charities, and Siteman Cancer Center.

## Appendix A Supplementary data

Appendix C

Authorship Team
