# Supplementary material for: Stigma measurement in health: a systematic review
Source: eClinicalMedicine. 2025 Jul 24;86:103360. doi: 10.1016/j.eclinm.2025.103360 (PMC12311962; doi:10.1016/j.eclinm.2025.103360)
Supplement: Appendix A [file mmc1.docx]

**Appendix A**

**Full Search Strategies**

**Embase**
Date Searched: 12/28/2022

Applied Database Supplied Limits: none

Number of Results: 4,486

Date updated: 7/26/2024

Results: 5,627

Date updated: 3/12/2025

Results: 6,174

Full Search Strategy:

('stigma'/exp OR 'self stigma'/exp OR ((patient OR anticipat* OR illness* OR chronic OR disease* OR hiv OR AIDS OR cancer OR internalized OR perceived OR background OR self) NEAR/3 stigma*):ti,ab,kw) AND ('patient'/exp OR 'AIDS patient'/exp OR 'cancer patient'/exp OR 'critically ill patient'/exp OR 'hospital patient'/exp OR 'Human immunodeficiency virus infected patient'/exp OR 'immunocompromised patient'/exp OR 'mental patient'/exp OR 'rehabilitation patient'/exp OR (patient* OR sufferer*):ti,ab,kw) AND ('scale'/exp OR 'clinical assessment tool'/exp OR 'instruments'/exp OR 'measurement'/exp OR 'outcomes'/exp OR 'outcome'/exp OR 'outcome assessment'/exp OR 'psychometry'/exp OR 'psychometry'/exp OR 'validation study'/exp OR 'internalized stigma of mental illness scale'/exp OR 'perceived stigmatization questionnaire'/exp OR (scale OR scales OR CIASS OR ‘clinical assessment tool*’ OR ‘clinical instrument*’ OR instrument OR instruments OR measurement* OR outcome* OR psychometric* OR psychometry OR psychomimetic OR Valid*):ti,ab,kw)

**Ovid Medline**
Date Searched: 12/8/2022
Applied Database Supplied Limits: none
Number of Results: 2,067

Date updated: 7/26/2024

Results: 2,579

Date updated: 3/12/2025

Results: 2,852

Full Search Strategy:

(exp Social Stigma/ OR ((patient OR anticipat* OR illness* OR chronic OR disease* OR hiv OR AIDS OR cancer OR internalized OR perceived OR background OR self) ADJ3 stigma*).ti,ab,kf.) AND (exp Patients/ OR exp Inpatients/ OR exp Mentally Ill Persons/ OR (patient* OR sufferer*).ti,ab,kf.) AND (exp Patient Reported Outcome Measures/ OR exp Outcome Assessment, Health Care/ OR exp "Outcome and Process Assessment, Health Care"/ OR exp Patient Outcome Assessment/ OR exp Validation Study/ OR (scale OR scales OR CIASS OR clinical assessment tool* OR clinical instrument* OR instrument OR instruments OR measurement* OR outcome* OR psychometric* OR psychometry OR psychomimetic OR Valid*).ti,ab,kf.)

**Scopus**
Date Searched: 12/28/2022
Applied Database Supplied Limits: none
Number of Results: 3,510

Date updated: 7/26/2024

Results: 4,364

Date updated: 3/12/2025

Results: 4,765

Full Search Strategy:

((TITLE-ABS-KEY((patient OR anticipat* OR illness* OR chronic OR disease* OR hiv OR AIDS OR cancer OR internalized OR perceived OR background OR self) W/3 stigma*))) AND ((TITLE-ABS-KEY(patient* OR sufferer*))) AND ((TITLE-ABS-KEY(scale OR scales OR CIASS OR “clinical assessment tool*” OR “clinical instrument*” OR instrument OR instruments OR measurement* OR outcome* OR psychometric* OR psychometry OR psychomimetic OR Valid*)))

**The Cochrane Library**

Date Searched: 12/28/2022
Applied Database Supplied Limits: none
Number of Results

CENTRAL: 467

CDSR: 3

Date updated: 7/26/2024

Results

CENTRAL: 617

CDSR: 3

Date updated: 3/12/2025

Results

CENTRAL: 653

CDSR: 3

Full Search Strategy:

([mh “Social Stigma”] OR ((patient OR anticipat* OR illness* OR chronic OR disease* OR hiv OR AIDS OR cancer OR internalized OR perceived OR background OR self) NEAR/3 stigma*):ti,ab,kw) AND ([mh “Patients”] OR [mh “Inpatients”] OR [mh “Mentally Ill Persons”] OR (patient* OR sufferer*):ti,ab,kw) AND ([mh “Patient Reported Outcome Measures”] OR [mh “Outcome Assessment, Health Care”] OR [mh “Outcome and Process Assessment, Health Care”] OR [mh “Patient Outcome Assessment”] OR [mh “Validation Study”] OR (scale OR scales OR CIASS OR “clinical assessment tool*” OR “clinical instrument*” OR instrument OR instruments OR measurement* OR outcome* OR psychometric* OR psychometry OR psychomimetic OR Valid*):ti,ab,kw)

**CINAHL Plus**
Date Searched: 12/28/2022
Applied Database Supplied Limits: none
Number of Results: 2,278

Date updated: 7/26/2024

Results: 2,643

Date updated: 3/12/2025

Results: 2,832

Full Search Strategy:

1. (MH "Stigma") OR TI ((patient OR anticipat* OR illness* OR chronic OR disease* OR hiv OR AIDS OR cancer OR internalized OR perceived OR background OR self) N3 stigma*) OR AB ((patient OR anticipat* OR illness* OR chronic OR disease* OR hiv OR AIDS OR cancer OR internalized OR perceived OR background OR self) N3 stigma*)

AND

1. (MH "Patients+") OR (MH "Cancer Patients") OR (MH "Critically Ill Patients") OR (MH "HIV-Positive Persons+") OR (MH "Inpatients") OR (MH "Psychiatric Patients+") OR (MH "Rehabilitation Patients") OR TI (patient* OR sufferer*) OR AB (patient* OR sufferer*)

AND

1. (MH "Scales") OR (MH "Clinical Assessment Tools+") OR (MH "Instrument Validation") OR (MH "Outcome Assessment") OR (MH "Validation Studies") OR TI (scale OR scales OR CIASS OR “clinical assessment tool*” OR “clinical instrument*” OR instrument OR instruments OR measurement* OR outcome* OR psychometric* OR psychometry OR psychomimetic OR Valid*) OR AB (scale OR scales OR CIASS OR “clinical assessment tool*” OR “clinical instrument*” OR instrument OR instruments OR measurement* OR outcome* OR psychometric* OR psychometry OR psychomimetic OR Valid*)

**APA PsycInfo**
Date Searched: 12/28/2022
Applied Database Supplied Limits: none
Number of Results: 1,408

Date updated: 7/26/2024

Results: 1,707

Date updated: 3/12/2025

Results: 1,455

Full Search Strategy:

1. DE "Stigma" OR DE "Self-Stigma" OR TI ((patient OR anticipat* OR illness* OR chronic OR disease* OR hiv OR AIDS OR cancer OR internalized OR perceived OR background OR self) N3 stigma*) OR AB ((patient OR anticipat* OR illness* OR chronic OR disease* OR hiv OR AIDS OR cancer OR internalized OR perceived OR background OR self) N3 stigma*)

AND

1. DE "Patients" OR DE "Psychiatric Patients" OR DE "Hospitalized Patients" OR TI (patient* OR sufferer*) OR AB (patient* OR sufferer*)

AND

1. DE "Social and Interpersonal Measures" AND DE "Measurement" OR DE "Measurement Models" OR DE "Psychophysical Measurement" AND DE "Test Validity" OR DE "Statistical Validity" OR TI (scale OR scales OR CIASS OR “clinical assessment tool*” OR “clinical instrument*” OR instrument OR instruments OR measurement* OR outcome* OR psychometric* OR psychometry OR psychomimetic OR Valid*) OR AB (scale OR scales OR CIASS OR “clinical assessment tool*” OR “clinical instrument*” OR instrument OR instruments OR measurement* OR outcome* OR psychometric* OR psychometry OR psychomimetic OR Valid*)

**Global Health**
Date Searched: 12/28/2022
Applied Database Supplied Limits: none
Number of Results: 44

Date updated: 7/26/2024

Results: 562

Date updated: 3/12/2025

Results: 902

Full Search Strategy:

1. TI ((patient OR anticipat* OR illness* OR chronic OR disease* OR hiv OR AIDS OR cancer OR internalized OR perceived OR background OR self) N3 stigma*) OR AB ((patient OR anticipat* OR illness* OR chronic OR disease* OR hiv OR AIDS OR cancer OR internalized OR perceived OR background OR self) N3 stigma*)

AND

1. TI (patient* OR sufferer*) OR AB (patient* OR sufferer*)

AND

1. TI (scale OR scales OR CIASS OR “clinical assessment tool*” OR “clinical instrument*” OR instrument OR instruments OR measurement* OR outcome* OR psychometric* OR psychometry OR psychomimetic OR Valid*) OR AB (scale OR scales OR CIASS OR “clinical assessment tool*” OR “clinical instrument*” OR instrument OR instruments OR measurement* OR outcome* OR psychometric* OR psychometry OR psychomimetic OR Valid*)

**ClinicalTrials.gov**
Date Searched: 12/8/2022

Number of Results: 0

Date updated: 7/26/2024

Results: 52

Date updated: 3/12/2025

Results: 59

Full Search Strategy:

(‘self-stigma’ OR ‘patient stigma’ OR ‘perceived stigma’ OR ‘internalized stigma’) AND (patient*) AND (scale OR CIASS OR “clinical assessment tool*” OR instrument OR measurement* OR outcome* OR psychometric* OR psychometry OR Valid*)

Inclusion/Exclusion Criteria

|  | ***Include*** | ***Exclude*** |
| --- | --- | --- |
| ***Type*** | - **Full-length research paper** - **Peer-reviewed** - **Observational studies** | - **Systematic review** - **Perspective/descriptive papers** - **Dissertations** - **Reports** - **Trial Registrations** |
| ***Focus*** | - **Stigma (any form)** - **Psychosocial tools that assess stigma** - **Internalized and externalized stigma** | - **Stigma related to individual identity or any characteristic other than a medical diagnosis** - **Tools assessing depression, anxiety** |
| ***Language*** | - **Written in English, Spanish, or Arabic** | - **Papers written in any other language** - **Only summary or abstract is available in English, Spanish, or Arabic** |
| ***Research participant*** | - **Individuals or patients with a disorder as the participant** - **Patient family participant** - **Caregiver participants** | - **Participant of the study being the general public/population/community** - **Participant of the study being clinician/clinical staff** |
| ***Discipline*** | - **Tools (measure, instrument, survey) that assess stigma** - **Utilization of stated tool** - **Development of stated tool including validation** - **Description of stated tool** | - **Tools measuring outcomes or conditions other than stigma** |
| ***Population*** | - **Adult** - **Pediatric** - **All medical conditions** | - **Individuals not diagnosed at any time with disease** |
