# Supplementary material for: Stigma measurement in health: a systematic review
Source: eClinicalMedicine. 2025 Jul 24;86:103360. doi: 10.1016/j.eclinm.2025.103360 (PMC12311962; doi:10.1016/j.eclinm.2025.103360)
Supplement: Appendix B [file mmc2.pdf]

Author	Publication Year	Title	Journal Name
Valerie A Earnshaw	2015	Intersectionality of internalized HIV stigma and internalized substance use stigma: Implications for depressive symptoms	Journal of Health Psychology
A Alswat, K. : Waslallah Alsuwat, R. : Metaeb Aljaed, K. : M Almalki, D. A.	2020	Prevalence and Impact of Weight Self Stigmatization on Type II Diabetes Glycemic and Cardiovascular Markers Control	Pakistan journal of biological sciences : PJBS
Abadiga, M. : Mosisa, G. : Amente, T. : Oluma, A.	2019	Health-related quality of life and associated factors among epileptic patients on treatment follow up at public hospitals of Wollega zones, Ethiopia, 2018	BMC research notes
Abaynew, Y. : Deribew, A. : Deribe, K.	2011	Factors associated with late presentation to HIV/AIDS care in South Wollo ZoneEthiopia: A case-control study	AIDS Research and Therapy
Abbamonte, J. M. : Ramlagan, S. : Lee, T. K. : Cristofari, N. V. : Weiss, S. M. : Peltzer, K. : Sifunda, S. : Jones, D. L.	2020	Stigma interdependence among pregnant HIV-infected couples in a cluster randomized controlled trial from rural South Africa	Social Science & Medicine
Abboud, S. : Noureddine, S. : Huijer, H. A. S. : Dejong, J. : Mokhbat, J.	2010	Quality of life in people living with HIV/AIDS in Lebanon	AIDS Care - Psychological and Socio-Medical Aspects of AIDS/HIV
Abdallah, K. : Buscetta, A. : Cooper, K. : Byeon, J. : Crouch, A. : Pink, S. : Minniti, C. : Bonham, V. L.	2020	Emergency Department Utilization for Patients Living With Sickle Cell Disease: Psychosocial Predictors of Health Care Behaviors	Annals of Emergency Medicine
Abdisa, E. : Fekadu, G. : Girma, S. : Shibiru, T. : Tilahun, T. : Mohamed, H. : Wakgari, A. : Takele, A. : Abebe, M. : Tsegaye, R.	2020	Self-stigma and medication adherence among patients with mental illness treated at Jimma University Medical Center, Southwest Ethiopia	International Journal of Mental Health Systems
Abdisa, E. : Tolesa, T. : Abadiga, M.	2021	Prevalence of Depressive Symptoms and Its Associated Factors among People Living with HIV Attending Public Hospitals of Nekemte Town, Western Ethiopia, 2021	Behavioural Neurology
Abebe, G. : Ayano, G. : Andargie, G. : Getachew, M. : Tesfaw, G.	2016	Prevalence and factors associated with anxiety among patients with common skin disease on follow up at Alert Referral Hospital, Addis Ababa, Ethiopia	African Journal of Psychiatry (South Africa)
Abebe, H. : Shumet, S. : Nassir, Z. : Agidew, M. : Abebaw, D.	2019	Prevalence of Depressive Symptoms and Associated Factors among HIV-Positive Youth Attending ART Follow-Up in Addis Ababa, Ethiopia	AIDS Research and Treatment
Abel, E. : Rew, L. : Gortner, E. : Delville, C. L.	2004	Cognitive reorganization and stigmatization among persons with HIV	Journal of Advanced Nursing (Wiley-Blackwell)
Acosta, F. J. : Aguilar, E. J. : Cejas, M. R. : Gracia, R.	2013	Beliefs about illness and their relationship with hopelessness, depression, insight and suicide attempts in schizophrenia	Psychiatria Danubina
Addis, B. : Minyihun, A. : Aschalew, A. Y.	2021	Health-related quality of life and associated factors among patients with epilepsy at the University of Gondar comprehensive specialized hospital, northwest Ethiopia	Quality of Life Research
Addis, B. : Wolde, M. : Minyihun, A. : Aschalew, A. Y.	2021	Prevalence of depression and associated factors among patients with epilepsy at the University of Gondar Comprehensive Specialized Hospital, Northwest Ethiopia, 2019	PLoS ONE
Adelekan, B. : Andrew, N. : Nta, I. : Gomwalk, A. : Ndembi, N. : Mensah, C. : Dakum, P. : Aliyu, A.	2019	Social barriers in accessing care by clients who returned to HIV care after transient loss to follow-up	AIDS Research and Therapy
Adeosun, Increase Ibukun : Adegbohun, Abosede Adekeji : Jeje, Oyetayo Oyewunmi : Adewumi, Tomilola Adejoke	2014	Experiences of discrimination by people with schizophrenia in Lagos, Nigeria	Journal of Public Mental Health
Adewuya, A. O. : Owoeye, A. O. : Erinfolami, A. O. : Ola, B. A.	2011	Correlates of self-stigma among outpatients with mental illness in Lagos, Nigeria	The International journal of social psychiatry
Adjei, P. : Nkromah, K. : Akpalu, A. : Laryea, R. : Osei Poku, F. : Ohene, S. : Puplampu, P. : Twumasi Aboagye, E.	2018	A cross-sectional comparative study of perceived stigma between patients with epilepsy and patients living with HIV/AIDS in Accra, Ghana	Epilepsy and Behavior
Aguiar, M. I. : Braga, V. A. : Garcia, J. H. : Lima, C. A. : Almeida, P. C. : Souza, A. M. : Rolim, I. L.	2016	Quality of life in liver transplant recipients and the influence of sociodemographic factors	Revista da Escola de Enfermagem da U S P
Ahmad, S. : Ismail, A. I. : Khan, T. M. : Akram, W. : Mohd Zim, M. A. : Ismail, N. E.	2017	Linguistic validation of stigmatisation degree, self-esteem and knowledge questionnaire among asthma patients using Rasch analysis	Journal of Asthma
Ahmad, Sohail : Ismail, Ahmad Izuanuddin : Zim, Mohd Arif Mohd : Ismail, Nahlah Elkudssiah	2019	Assessment of Self-Stigma, Self-Esteem, and Asthma Control: A Preliminary Cross-Sectional Study Among Adult Asthmatic Patients in Selangor, Malaysia	Frontiers in public health
Ahmed, Abdullahi : Osinubi, Medinat O. : Fasiku, Mojirola M. : Uthman, Muhammed M. B. : Soyannwo, Tolulope : Jimoh, Saheed O. : Olubodun, Tope	2022	Psychological and stigma profiles of HIV patients in a North-Central city of Nigeria	Journal of family medicine and primary care
Ahmed, A. O. : Birgenheir, D. : Buckley, P. F. : Mabe, P. A.	2013	A psychometric study of recovery among Certified Peer Specialists	Psychiatry Research
Ahmed, A. : Saqlain, M. : Umair, M. M. : Hashmi, F. K. : Saeed, H. : Amer, M. : Blebil, A. Q. : Dujaili, J. A.	2021	Stigma, Social Support, Illicit Drug Use, and Other Predictors of Anxiety and Depression Among HIV/AIDS Patients in Pakistan: A Cross-Sectional Study	Frontiers in public health
Ahmed Suleiman, M. M. : Sahal, N. : Sodemann, M. : El Sony, A. : Aro, A. R.	2013	Tuberculosis stigma in Gezira State, Sudan: A case-control study	International Journal of Tuberculosis and Lung Disease
Airlie, J. : Baker, G. A. : Smith, S. J. : Young, C. A.	2001	Measuring the impact of multiple sclerosis on psychosocial functioning: The development of a new self-efficacy scale	Clin. Rehabil.
Ak, P. D. : Atakli, D. : Yuksel, B. : Guveli, B. T. : Sari, H.	2015	Stigmatization and social impacts of epilepsy in Turkey	Epilepsy and Behavior
Akdağ, E. M. : Kotan, V. O. : Kose, S. : Tıkır, B. : Aydemir, M. Ç : Okay, İ T. : Göka, E. : Özkaya, G.	2018	The relationship between internalized stigma and treatment motivation, perceived social support, depression and anxiety levels in opioid use disorder	Psychiatry and Clinical Psychopharmacology
Akhtar, N. : Batool, I.	2021	Social stigma perceived by patients of tuberculosis: Gender, age and marital status based differences	Rawal Medical Journal
Akoglu, G. : Yildiz, I. : Karaismailoğlu, E. : Esme, P.	2021	Disease severity and poor mental health are the main predictors of stigmatization in patients with hidradenitis suppurativa	Dermatologic Therapy
Al Eid, N. A. : Arnout, B. A. : Alqahtani, M. M. J. : Fadhel, F. H. : Abdelmotelab, A. S.	2021	The mediating role of religiosity and hope for the effect of self-stigma on psychological well-being among COVID-19 patients	Work (Reading, Mass.)
Al Raqaishi, Huda : Al Qadire, Mohammad : Alzaabi, Omar : Al Omari, Omar	2022	Health-Related Stigma, Social Support, Self-Efficacy, and Self-Care Actions Among Adults With Sickle Cell Disease in Oman	Clinical Nursing Research
Al-Zamel, Lamia A. : Al-Thunayan, Shatha F. : Al-Rasheed, Afnan A. : Alkathiri, Munirah A. : Alamri, Faisal : Alqahtani, Faleh : Alali, Amer S. : Almohammed, Omar A. : Asiri, Yousif A. : Bashatah, Adel S. : AlRuthia, Yazed	2021	Validation and Cultural Adaptation of Explanatory Model Interview Catalogue (EMIC) in Assessing Stigma among Recovered Patients with COVID-19 in Saudi Arabia	International journal of environmental research and public health
Alasmawi, Khulood : Mann, Farhana : Lewis, Gemma : White, Sarah : Mezey, Gill : Lloyd-Evans, Brynmor	2020	To what extent does severity of loneliness vary among different mental health diagnostic groups: A cross-sectional study	International Journal of Mental Health Nursing
Alateeq, D. : AlDaoud, A. : AlHadi, A. : AlKhalaf, H.	2016	The experience and impact of stigma in Saudi people with a mood disorder	European Psychiatry
Albikawi, Z. : Abuadas, M.	2021	Quality of life and self-stigma of schizophrenia patient's caregiver tool: Development and validation using classical test theory and rasch analysis	South African Journal of Psychiatry
Alchawa, Mohamad : Naja, Sarah : Ali, Khaled : Kehyayan, Vahe : Haddad, Peter Michael : Bougmiza, Iheb	2022	COVID-19 perceived stigma among survivors: A cross-sectional study of prevalence and predictors	The European journal of psychiatry
Alemayehu, A. T. : Deribe, K. : Tomczyk, S. : Mousley, E. : Davey, G.	2015	Stigma towards a neglected tropical disease: Felt and enacted stigma scores among podoconiosis patients in Northern Ethiopia	American Journal of Tropical Medicine and Hygiene
Alemayehu, Y. : Asfaw, H. : Girma, M.	2022	Magnitude and factors associated with treatment non-adherence among patients with depressive disorders at St. Amanuel Mental Specialized Hospital, Addis Ababa, Ethiopia, 2019: A cross sectional study	PLoS ONE
Alemayehu, Yadeta : Demilew, Demeke : Asfaw, Getachew : Asfaw, Henock : Alemnew, Nigus : Tadesse, Agitu	2020	Internalized Stigma and Associated Factors among Patients with Major Depressive Disorder at the Outpatient Department of Amanuel Mental Specialized Hospital, Addis Ababa, Ethiopia, 2019: A Cross-Sectional Study	Psychiatry journal
Alemu, A. : Meskele, M. : Darebo, T. D. : Handiso, T. B. : Abebe, A. : Paulos, K.	2022	Perceived HIV Stigma and Associated Factors Among Adult ART Patients in Wolaita Zone, Southern Ethiopia	HIV/AIDS - Research and Palliative Care
Alemu, W. G. : Tilahun, S. Y. : Bekele, E. : Eshitu, B. : Kerebih, H.	2022	Prevalence and associated factors of perceived stigma among medically ill patients on follow-up screened positive for depression in Ethiopia: Facility-based cross-sectional study	BMJ Open
Algarin, A. B. : Li, Y. : Cohen, R. A. : Cook, C. L. : Brumback, B. : Cook, R. L. : Ibañez, G. E.	2021	HIV-related stigma and life goals among people living with HIV (PLWH) in Florida	Quality of Life Research
Algarin, A. B. : Sheehan, D. M. : Varas-Diaz, N. : Fennie, K. P. : Zhou, Z. : Spencer, E. C. : Cook, R. L. : Morano, J. P. : Ibanez, G. E.	2020	Health Care-Specific Enacted HIV-Related Stigma's Association with Antiretroviral Therapy Adherence and Viral Suppression among People Living with HIV in Florida	AIDS Patient Care and STDs
Algarin, A. B. : Zhou, Z. : Cook, C. L. : Cook, R. L. : Ibañez, G. E.	2019	Age, Sex, Race, Ethnicity, Sexual Orientation: Intersectionality of Marginalized-Group Identities and Enacted HIV-Related Stigma Among People Living with HIV in Florida	AIDS Behav.
Ali, A. : King, M. : Strydom, A. : Hassiotis, A.	2015	Self-reported stigma and symptoms of anxiety and depression in people with intellectual disabilities: Findings from a cross sectional study in England	J. Affective Disord.

Ali, A. M. : Green, J.	2019	Factor structure of the depression anxiety stress Scale-21 (DASS-21): Unidimensionality of the Arabic version among Egyptian drug users	Substance abuse treatment, prevention, and policy
Alkathiri, M. A. : Almohammed, O. A. : Alqahtani, F. : Alruthia, Y.	2022	Associations of Depression and Anxiety with Stigma in a Sample of Patients in Saudi Arabia Who Recovered from COVID-19	Psychol. Res. Behav. Manage.
Allerby, K. : Sameby, B. : Brain, C. : Joas, E. : Quinlan, P. : Sjöström, N. : Burns, T. : Waern, M.	2015	Stigma and burden among relatives of persons with schizophrenia: Results from the Swedish COAST study	Psychiatr. Serv.
Alonso, J. : Buron, A. : Bruffaerts, R. : He, Y. : Posada-Villa, J. : Lepine, J. P. : Angermeyer, M. C. : Levinson, D. : De Girolamo, G. : Tachimori, H. : Mneimneh, Z. N. : Medina-Mora, M. E. : Ormel, J. : Scott, K. M. : Gureje, O. : Haro, J. M. : Gluzman, S. : Lee, S. : Vilagut, G. : Kessler, R. C. : Von Korff, M.	2008	Association of perceived stigma and mood and anxiety disorders: Results from the World Mental Health Surveys	Acta Psychiatr. Scand.
Alpsoy, E. : Polat, M. : FettahlıoGlu-Karaman, B. : Karadag, A. S. : Kartal-Durmazlar, P. : YalCın, B. : Emre, S. : Didar-Balcı, D. : Bilgic-Temel, A. : Arca, E. : Koca, R. : Gunduz, K. : Borlu, M. : Ergun, T. : Dogruk-Kacar, S. : Cordan-Yazici, A. : Dursun, P. : Bilgic, O. : Gunes-Bilgili, S. : Sendur, N. : Baysal, O. : Halil-Yavuz, I. : Yagcioglu, G. : Yilmaz, E. : Kavuzlu, U. : Senol, Y.	2017	Internalized stigma in psoriasis: A multicenter study	Journal of Dermatology
Alpsoy, E. : Polat, M. : Yavuz, I. H. : Kartal, P. : Balci, D. D. : Karadag, A. S. : Bilgic, A. : Arca, E. : Karaman, B. F. : Emre, S. : Adisen, E. : Sendur, N. : Bilgic, O. : Yazici, A. C. : Yalcin, B. : Koca, R. : Gunduz, K. : Borlu, M. M. : Ergun, T. : Dursun, P. : Bilgili, S. G. : Adanir, A. S. : Gulekon, A. : Yagcioglu, G. : Yilmaz, E. : Kavuzlu, U. : Senol, Y.	2020	Internalized stigma in pediatric psoriasis: A comparative multicenter study	Annals of Dermatology
Alsayed, N. S. : Sereika, S. M. : Albrecht, S. A. : Terry, M. A. : Erlen, J. A.	2017	Testing a model of health-related quality of life in women living with HIV infection	Qual. Life Res.
Altraide, D. : Okeafor, C. : Otike-Odibi, B.	2021	Psychiatric morbidity among dermatology patients: A hospital-based cross-sectional study	Journal of Dermatology and Dermatologic Surgery
Alvidrez, J. : Snowden, L. R. : Rao, S. M. : Boccellari, A.	2009	Psychoeducation to address stigma in black adults referred for mental health treatment: a randomized pilot study	Community Mental Health Journal
Alvidrez, J. : Snowden, L. R. : Patel, S. G.	2010	The relationship between stigma and other treatment concerns and subsequent treatment engagement among black mental health clients	Issues in Mental Health Nursing
Amaresha, A. C. : Kalmady, S. V. : Joseph, B. : Agarwal, S. M. : Narayanaswamy, J. C. : Venkatasubramanian, G. : Muralidhar, D. : Subbakrishna, D. K.	2018	Short term effects of brief need based psychoeducation on knowledge, self-stigma, and burden among siblings of persons with schizophrenia: A prospective controlled trial	Asian Journal of Psychiatry
Amico, K. Rivet : Crawford, Jessica : Ubong, Ini : Lindsey, Jane C. : Gaur, Aditya H. : Horvath, Keith : Goolsby, Rachel : Mueller Johnson, Megan : Dallas, Ronald : Heckman, Barbara : Filipowicz, Teresa : Polier, Melissa : Rupp, Betty M. : Hudgens, Michael	2021	Correlates of High HIV Viral Load and Antiretroviral Therapy Adherence Among Viremic Youth in the United States Enrolled in an Adherence Improvement Intervention	AIDS Patient Care & STDs
Amini-Tehrani, M. : Zamanian, H. : Daryaafzoon, M. : Andikolaei, S. : Mohebbi, M. : Imani, A. : Tahmasbi, B. : Foroozanfar, S. : Jalali, Z.	2021	Body image, internalized stigma and enacted stigma predict psychological distress in women with breast cancer: A serial mediation model	Journal of advanced nursing
Ammirati, R. J. : Lamis, D. A. : Campos, P. E. : Farber, E. W.	2015	Optimism, well-being, and perceived stigma in individuals living with HIV	AIDS Care - Psychological and Socio-Medical Aspects of AIDS/HIV
Amore, M. : Murri, M. B. : Calcagno, P. : Rocca, P. : Rossi, A. : Aguglia, E. : Bellomo, A. : Blasi, G. : Carpiniello, B. : Cuomo, A. : Dell'Osso, L. : Di Giannantonio, M. : Giordano, G. M. : Marchesi, C. : Monteleone, P. : Montemagni, C. : Oldani, L. : Pompili, M. : Roncone, R. : Rossi, R. : Siracusano, A. : Vita, A. : Zeppegno, P. : Corso, A. : Arzani, C. : Galderisi, S. : Maj, M. : Petrilli, G. : Respino, M. : Papalino, M. : Falsetti, A. : Calia, V. M. : Barlati, S. : Deste, G. : Turrina, C. : Pinna, F. : Lai, A. : Di Santa Sofia, S. L. : Signorelli, M. S. : Petralia, A. : Pettorruso, M. : Barone, G. : Salone, A. : Piegari, G. : Aiello, C. : Brando, F. : Giuliani, L. : Altamura, M. : Carnevale, R. : Padalino, F. : Giusti, L. : Salza, A. : Ussorio, D. : Pizziconi, G. : Santarelli, V. : Pacitti, F. : De Bartolomeis, A. : Gambaro, E. : Gattoni, E. : Gramaglia, C. : De Panfilis, C. : Ossola, P. : Tonna, M. : Carmassi, C. : Carpita, B. : Cremone, I. : Comparelli, A. : Corigliano, V. : Brugnoli, R. : Corrivetti, G. : Cascino, G. : Del Buono, G. : Fagiolini, A. : Bolognesi, S. : Goracci, A. : Di Lorenzo, G. : Niolu, C. : Ribolsi, M. : Bellino, S. : Bozzatello, P. : Brasso, C.	2020	The association between insight and depressive symptoms in schizophrenia: Undirected and Bayesian network analyses	European Psychiatry
Amri, N. S. : Zakaria, R. : Mohamad, N.	2022	Stigma and Depression among Tuberculosis Patients in Kedah, Malaysia	IIUM Med. J. Malaysia.
Anagnostouli, M. : Katsavos, S. : Artemiadis, A. : Zacharis, M. : Argyrou, P. : Theotoka, I. : Christidi, F. : Zalonis, I. : Liappas, I.	2016	Determinants of stigma in a cohort of hellenic patients suffering from multiple sclerosis: A cross-sectional study	BMC Neurology
Anand, T. : Kumar, D. A. : Sharma, N. : Saha, R. : Krishnamurthy, L. : Singh, S. V. : Ingle, G. K.	2014	Perception of stigma towards TB among patients on DOTS & patients attending general OPD in Delhi	The Indian journal of tuberculosis
Anand, Tanya : Kandasamy, Arun : Suman, L. N.	2022	Self-stigma, hope for future, and recovery: An exploratory study of men with early-onset substance use disorder	Industrial psychiatry journal
Anderson, J. D. : Li, X. : Qiao, S. : Zhou, Y. : Shen, Z.	2020	The mediating effects of functions of social support on HIV-related trauma and health-related quality of life for PLHIV in China	AIDS Care - Psychological and Socio-Medical Aspects of AIDS/HIV
Andersson, K. : Ozanne, A. : Zelano, J. : Malmgren, K. : Chaplin, J. E.	2022	Perceived stigma in adults with epilepsy in Sweden and associations with country of birth, socioeconomic status, and mental health	Epilepsy and Behavior
Andu, E. : Wagenaar, B. H. : Kemp, C. G. : Nevin, P. E. : Simoni, J. M. : Andrasik, M. : Cohn, S. E. : French, A. L. : Rao, D.	2018	Risk and protective factors of posttraumatic stress disorder among African American women living with HIV	AIDS Care - Psychological and Socio-Medical Aspects of AIDS/HIV
Anish, T. S. : Vijaykumar, K. : Simi, S. M.	2011	Determinants of rapid progression to immunodeficiency syndrome among people infected with human immunodeficiency virus, Kerala, India	Indian Journal of Sexually Transmitted Diseases
Ansari, Eram : Mishra, Sudha : Tripathi, Adarsh : Kar, Sujita Kumar : Dalal, Pronob Kumar	2020	Cross-sectional study of internalised stigma and medication adherence in patients with obsessive compulsive disorder	General psychiatry
Arbisi, P. A. : Rusch, L. : Polusny, M. A. : Thuras, P. : Erbes, C. R.	2013	Does cynicism play a role in failure to obtain needed care? Mental health service utilization among returning U.S. National guard soldiers	Psychol. Assess.
Arcêncio, R. A. : Crispim, J. de A. : Touso, M. M. : Popolin, M. P. : Rodrigues, L. B. B. : Freitas, I. M. de : Yamamura, M. : Santos Neto, M.	2014	Preliminary validation of an instrument to assess social support and tuberculosis stigma in patients' families	Public Health Action
Arda Sürücü, Hamdiye : Baran Durmaz, Gülbeyaz : Turan, Engin	2020	Does Type 1 Diabetic Adolescents' Fear of Stigmatization Predict a Negative Perception Insulin Treatment?	Clinical Nursing Research
Arda Sürücü, H. : Okur Arslan, H. : Çetik, S.	2020	Self-care, A1C and stigmatization as predictors of a negative perception of insulin among adults with type 2 diabetes: a hospital-based study in Turkey	J. Health Res.
Aryal, S. : Badhu, A. : Pandey, S. : Bhandari, A. : Khatiwoda, P. : Khatiwada, P. : Giri, A.	2012	Stigma related to tuberculosis among patients attending DOTS clinics of Dharan municipality	Kathmandu University medical journal (KUMJ)
Asgarian, F. S. : Keyhani, A. : Moghadasi, A. N. : Sahraian, M. A. : Etesam, F.	2020	Predictors of suicidal ideation among Iranian patients with multiple sclerosis	Iranian Journal of Neurology
Ashaba, C. : Musoke, D. : Wafula, S. T. : Konde-Lule, J.	2021	Stigma among tuberculosis patients and associated factors in urban slum populations in Uganda	African Health Sciences
Asrat, Biksegn : Ayenalem, Alem Eskeziya : Yimer, Tenaw	2018	Internalized Stigma among Patients with Mental Illness Attending Psychiatric Follow-Up at Dilla University Referral Hospital, Southern Ethiopia	Psychiatry journal
Assefa, D. : Shibre, T. : Asher, L. : Fekadu, A.	2012	Internalized stigma among patients with schizophrenia in Ethiopia: A cross-sectional facility-based study	BMC Psychiatry
Au, Chi-Hung : Wong, Corine Sau-Man : Law, Chi-Wing : Wong, Ming-Cheuk : Chung, Ka-Fai	2019	Self-stigma, stigma coping and functioning in remitted bipolar disorder	General Hospital Psychiatry
Aukst Margetic, B. : Kukulj, S. : Galic, K. : Saric Zolj, B. : Jakšić, N.	2020	Personality and Stigma in Lung Cancer Patients	Psychiatria Danubina
Aukst-Margetić, B. : Jakšić, N. : Boričević Maršanić, V. : Jakovljević, M.	2014	Harm avoidance moderates the relationship between internalized stigma and depressive symptoms in patients with schizophrenia	Psychiatry Research
Aung, M. N. : Moolphate, S. : Kitajima, T. : Siriwarothai, Y. : Takamtha, P. : Katanyoo, C. : Okamura, H. : Field, M. : Noyama, O. : Deerojanawong, J. : Klinbuayaem, V.	2017	Perceived stigma of HIV patients receiving task-shifted primary care service and its relation to satisfaction with health service	Journal of Infection in Developing Countries
Aung, S. : Hardy, N. : Chrysanthopoulou, S. : Htun, N. : Kyaw, A. : Tun, M. S. : Aung, K. W. : Kantor, R. : Rana, A.	2022	Evaluation of peer-to-peer HIV counseling in Myanmar: a measure of knowledge, adherence, and barriers	AIDS Care - Psychological and Socio-Medical Aspects of AIDS/HIV
Aurpibul, L. : Sophonphan, J. : Malee, K. : Kerr, S. J. : Sun, L. P. : Ounchanum, P. : Kosalaraksa, P. : Ngampiyaskul, C. : Kanjanavanit, S. : Chettra, K. : Suwanlerk, T. : Mellins, C. A. : Paul, R. : Robbins, R. N. : Ananworanich, J. : Puthanakit, T.	2021	HIV-related enacted stigma and increase frequency of depressive symptoms among Thai and Cambodian adolescents and young adults with perinatal HIV	International Journal of STD and AIDS
Aurpibul, L. : Tangmunkongvorakul, A. : Jirattikorn, A. : Ayuttacorn, A. : Musumari, P. M. : Srithanaviboonchai, K.	2022	Depressive symptoms, HIV disclosure, and HIV-related stigma among migrant workers living with HIV in Chiang Mai, Thailand	AIDS Care - Psychological and Socio-Medical Aspects of AIDS/HIV
Austin, J. K. : Perkins, S. M. : Dunn, D. W.	2014	A model for internalized stigma in children and adolescents with epilepsy	Epilepsy Behav.

Ayalew, Mohammed : Workicho, Abdulhalik : Tesfaye, Elias : Hailesilasie, Hailemariam : Abera, Mubarek	2019	Burden among caregivers of people with mental illness at Jimma University Medical Center, Southwest Ethiopia: a cross-sectional study	Annals of General Psychiatry
Ayar, D. : Karasu, F. : Sahpolat, M.	2022	The relationship between levels of solution-focused thinking and internalized stigma and social functionality in mental disorders	Perspectives in psychiatric care
Aydemir, N.	2020	Overprotection and determinants of overprotection in adults with epilepsy	Seizure
Aydemir, O. : Akkaya, C.	2011	Association of social anxiety with stigmatisation and low self-esteem in remitted bipolar patients	Acta Neuropsychiatrica
Aytac, I.	2020	Stigma in head and neck tuberculosis patients: evaluation and analysis	Annals of Medical Research
Bagchi, A. D. : Thompson, A. : Damas, K. : Corasim, E.	2020	Step UP! To Stamp Out Stigma: adapting and testing a bystander intervention to reduce HIV-related stigma	Journal of public health (germany)
Bagley, C.	2005	Robustness of two single-item self-esteem measures: Cross-validation with a measure of stigma in a sample of psychiatric patients	Perceptual and Motor Skills
Bagley, Christopher : King, Michael	2005	Exploration of three stigma scales in 83 users of mental health services: Implications for campaigns to reduce stigma	Journal of Mental Health
Bahar, Zuhal : Cal, Ayse : Beser, Ayse : Cavusoglu, Figen : Deveci, Aydin : Badur, Selim : Bahar, Ismail Hakki	2021	A study on the adaptation of the hiv/aids-related stigma scale into turkish	Perspectives in Psychiatric Care
Bahm, A. : Forchuk, C.	2009	Interlocking oppressions: The effect of a comorbid physical disability on perceived stigma and discrimination among mental health consumers in Canada	Health Soc. Care Community
Baik, Dawon : Liu, Jianfang : Cho, Hwayoung : Schnall, Rebecca	2020	Factors Related to Biological Sex Differences in Engagement with Healthcare Providers in Persons Living with HIV	AIDS and behavior
Bajaj, Jitin : Tripathi, Manjari : Dwivedi, Rekha : Sapra, Savita : Gulati, Sheffali : Garg, Ajay : Tripathi, Madhavi : Bal, Chandra S. : Chandra, Sarat P.	2018	Does surgery help in reducing stigma associated with drug refractory epilepsy in children?	Epilepsy & behavior : E&B
Baker, G. A. : Brooks, J. : Buck, D. : Jacoby, A.	2000	The stigma of epilepsy: A European perspective	Epilepsia
Bakolis, I. : Thornicroft, G. : Vitoratou, S. : Rüsch, N. : Bonetto, C. : Lasalvia, A. : Evans-Lacko, S.	2019	Development and validation of the DISCUS scale: A reliable short measure for assessing experienced discrimination in people with mental health problems on a global level	Schizophrenia Research
Baksi, Altun : Arda Surucu, Hamdiye : Genc, Hasan	2021	Hope, Sociodemographic and Clinical Characteristics as Predictors of Stigma-Related Negative Discrimination Experiences of Patients With Primary Malignant Brain Tumor	The Journal of neuroscience nursing : journal of the American Association of Neuroscience Nurses
Balakrishnan, K. : Edwards, T. C. : Perkins, J. A.	2012	Functional and symptom impacts of pediatric head and neck lymphatic malformations: Developing a patient-derived instrument	Otolaryngology - Head and Neck Surgery (United States)
Baldwin, M. L. : DeSerpa, A. C. : Shields, M. : Marcus, S. C.	2022	Workplace Disclosure of Serious Mental Illness: Theory and Evidence	Journal of Mental Health Policy and Economics
Ballesteros, Javier : Martinez-Gines, Maria L. : Garcia-Dominguez, Jose M. : Forero, Lucia : Prefasi, Daniel : Maurino, Jorge : W. Impact Study Group	2019	Assessing Stigma in Multiple Sclerosis: Psychometric Properties of the Eight-Item Stigma Scale for Chronic Illness (SSCI-8)	International journal of MS care
Bandyopadhyay, A. : Samanta, S. : Mukherjee, A. : Bhattacherjee, S.	2022	Assessing the prevalence and socio-demographic determinants of the unsolved problem of perceived social stigma: A study among leprosy-affected persons in North Bengal, India	Med. J. Babylon.
Barke, A. : Nyarko, S. : Klecha, D.	2011	The stigma of mental illness in Southern Ghana: attitudes of the urban population and patients' views	Social psychiatry and psychiatric epidemiology
Barke, J. : Coad, J. : Harcourt, D.	2016	The Role of Appearance in Adolescents' Experiences of Neurofibromatosis Type 1: A Survey of Young People and Parents	Journal of genetic counseling
Barker, David : Enimil, Anthony : Galárraga, Omar : Bosomtwe, Dennis : Mensah, Nicholas : Thamotharan, Sneha : Henebeng, Esther : Brown, Larry : Kwara, Awewura	2019	In-Clinic Adolescent Peer Group Support for Engagement in Sub-Saharan Africa: A Feasibility and Acceptability Trial	Journal of the International Association of Providers of AIDS Care
Barlati, Stefano : Morena, Donato : Nibbio, Gabriele : Cacciani, Paolo : Corsini, Paola : Mosca, Alessandra : Deste, Giacomo : Accardo, Vivian : Turrina, Cesare : Valsecchi, Paolo : Vita, Antonio	2021	Internalized stigma among people with schizophrenia: Relationship with socio-demographic, clinical and medication-related features	Schizophrenia Research
Barlati, S. : Nibbio, G. : Morena, D. : Cacciani, P. : Corsini, P. : Mosca, A. : Deste, G. : Accardo, V. : Regina, V. : Lisoni, J. : Turrina, C. : Valsecchi, P. : Vita, A.	2022	Autistic Symptoms in Schizophrenia: Impact on Internalized Stigma, Well-Being, Clinical and Functional Characteristics	Frontiers in Psychiatry
Barmada, H. H. : Patil, D. : Roberts, S. P. : Colon-Rivera, H. A. : Chang, G.	2018	Perceived Stigma and Satisfaction with Care among Veterans Receiving Methadone Maintenance Treatment: A Pilot Study	Addict. Disord. Treat.
Barradas, S. : Finck, C.	2021	Relationship between stigma and health-related quality of life in patients diagnosed with HIV	HIV and AIDS Review
Barroso, J. : Relf, M. V. : Williams, M. S. : Arscott, J. : Moore, E. D. : Caiola, C. : Silva, S. G.	2014	A randomized controlled trial of the efficacy of a stigma reduction intervention for HIV-infected women in the deep south	AIDS Patient Care and STDs
Basha, E. A. : Derseh, B. T. : Wubetu, A. D. : Engidaw, N. A. : Gizachew, K. D.	2021	Factors Affecting Social Support Status of People Living with HIV/AIDS at Selected Hospitals of North Shewa Zone, Amhara Region, Ethiopia	Journal of Tropical Medicine
Basoglu Koseahmet, F. : Polat, B. : Gozubatik-Celik, R. G. : Baytekin, I. : Soylu, M. G. : Ceyhan Dirican, A. : Ozturk, M.	2022	An invisible cause of disability: stigma in migraine and epilepsy	Neurol. Sci.
Bassirnia, A. : Briggs, J. : Kopeykina, I. : Mednick, A. : Yaseen, Z. : Galynker, I.	2015	Relationship between personality traits and perceived internalized stigma in bipolar patients and their treatment partners	Psychiatry Research
Bautista, R. E. D. : Shapovalov, D. : Shoraka, A. R.	2015	Factors associated with increased felt stigma among individuals with epilepsy	Seizure
Baybaş, S. : Yıldırım, Z. : Özhan, H. E. : Dirican, A. : Dirican, A.	2017	Development and validation of the stigma scale for epilepsy in Turkey	Epilepsy and Behavior
Bayomy, H. E. : Albedaiwi, Y. : Alabdulatif, S. K. A. : Almutairi, F. S. F. : Aloufi, F. A. S. : Alruwaili, R. H. A. : Salama, B. : Alenezy, A.	2022	Psoriasis in Northern Saudi Arabia: Clinical features and implications for quality of life	J. Public Health Res.
Baysan Arabaci, L. : Ayakdaş Dağli, D. : Taş, G. : Büyükbayram Arslan, A.	2020	Stigmatization and Social Functioning Levels of Patients With Alcohol Use Disorders	Journal of addictions nursing
Bédard, Sarah : Sasewich, Hannah : Culling, Jessica : Turner, Simon R. : Pellizzari, Janelle : Johnson, Scott : Bédard, Eric L. R.	2022	Stigma in Early-Stage Lung Cancer	Annals of Behavioral Medicine
Bediako, S. M. : Lanzkron, S. : Diener-West, M. : Onojobi, G. : Beach, M. C. : Haywood, C.	2016	The Measure of Sickle Cell Stigma: Initial findings from the Improving Patient Outcomes through Respect and Trust study	Journal of health psychology
Been, S. K. : Van De Vijver, D. A. M. C. : Nieuwkerk, P. T. : Brito, I. : Stutterheim, S. E. : Bos, A. E. R. : Wolfers, M. E. G. : Pogány, K. : Verbon, A.	2016	Risk factors for non-adherence to cART in immigrants with HIV living in the Netherlands: Results from the Rotterdam ADherence (ROAD) project	PLoS ONE
Bega, D. : Palmentera, P. : Wagner, A. : Hovde, M. : Barish, B. : Kwasny, M. J. : Simuni, T.	2017	Laughter is the best medicine: The Second City® improvisation as an intervention for Parkinson's disease	Parkinsonism and Related Disorders
Beitollahi, M. : Forouzi, M. A. : Tirgari, B. : Jahani, Y.	2022	Fatigue, stigma, and mood in patients with multiple sclerosis: effectiveness of guided imagery	BMC Neurology
Ben-Shlomo, Y. : Camfield, L. : Warner, T.	2002	What are the determinants of quality of life in people with cervical dystonia?	Journal of Neurology, Neurosurgery & Psychiatry
Bengochea-Seco, R. : Arrieta-Rodríguez, M. : Fernández-Modamio, M. : Santacoloma-Cabero, I. : Gómez de Tojeiro-Roce, J. : García-Polavieja, B. : Santos-Zorrozúa, B. : Gil-Sanz, D.	2018	Adaptation into Spanish of the Internalised Stigma of Mental Illness scale to assess personal stigma	Revista de Psiquiatría y Salud Mental
Berg, Rigmor C. : Carter, Dakota : Ross, Michael W.	2017	A Mixed-Method Study on Correlates of HIV-Related Stigma Among Gay and Bisexual Men in the Southern United States	JANAC: Journal of the Association of Nurses in AIDS Care
Berge, Melinda : Ranney, Molly	2005	Self-esteem and stigma among persons with schizophrenia: implications for mental health	Care management journals : Journal of case management ; The journal of long term home health care
Berger, B. E. : Ferrans, C. E. : Lashley, F. R.	2001	Measuring stigma in people with HIV: psychometric assessment of the HIV Stigma Scale	Research in Nursing & Health
Berry, C. : Greenwood, K.	2018	Direct and indirect associations between dysfunctional attitudes, self-stigma, hopefulness and social inclusion in young people experiencing psychosis	Schizophr. Res.
Bhana, A. : Mellins, C. A. : Small, L. : Nestadt, D. F. : Leu, C. S. : Petersen, I. : Machanyangwa, S. : McKay, M.	2016	Resilience in perinatal HIV+ adolescents in South Africa	AIDS Care - Psychological and Socio-Medical Aspects of AIDS/HIV
Bhwana, D. : Das, L. : Challe, D. : Makunde, H. W. : Mmbando, B. P. : Colebunders, R.	2021	A peer support group intervention to decrease epilepsy related stigma in an onchocerciasis endemic area in Mahenge, Tanzania	Epilepsia
Bielen, Ivan : Friedrich, Latica : Sruk, Ana : Prvan, Miljenka Planjar : Hajnsek, Sanja : Petelin, Zeljka : Susak, Renata : Candrlic, Marija : Jacoby, Ann	2014	Factors associated with perceived stigma of epilepsy in Croatia: a study using the revised Epilepsy Stigma Scale	Seizure

Bin Kassim, Mohd Shaiful Azlan : Muhamad, Nor Asiah : bin Ramli, Muhd Hafizuddin Taufik : Azman, Azlinda : Hashim, Mohd Hazrin Hasim : bin Bistari, Hanif : binti Ahmad, Fazila Haryati : Shahein, Nik Adilah Binti : bin Rezali, Muhammad Solihin : Chan Ying, Ying : Sahril, Norhafizah Binti : Bt Ab Wahab, Nor' Ain : Bin Abd Mutalip, Mohd Hatta : binti Ahmad, Noor Ani	2020	DEVELOPMENT OF THE MALAY VERSION OF THE ENGLISH HIV STIGMA QUESTIONNAIRE: A REVIEW	International Journal of Public Health & Clinical Sciences (IJPHCS)
Bint-E-Saif, S. : Shahzad, S.	2020	Urdu translation and adaptation of the HIV stigma scale in Pakistan injectable drug users with HIV	Journal of the Pakistan Medical Association
Bipeta, R. : Yerramilli, S. S. R. R. : Pillutla, S. V.	2020	Perceived stigma in remitted psychiatric patients and their caregivers and its association with self-esteem, quality of life, and caregiver depression	East Asian Archives of Psychiatry
Blake, A. : Asnani, V. : Leger, R. R. : Odesina, V. : Wagner, L. : Knight-Madden, J. : Asnani, M.	2016	Stigma and illness uncertainty: Adding to the burden of sickle cell disease	West Indian Medical Journal
Blake Helms, C. : Atkins, Ghislaine : Clay, Olivio : Turan, Bulent : Turan, Janet : Kempf, Mirjam-Colette : Raper, James : Mugavero, Michael	2017	Interpersonal Mechanisms Contributing to the Association Between HIV-Related Internalized Stigma and Medication Adherence	AIDS & Behavior
Bleich, S. N. : Gudzune, K. A. : Bennett, W. L. : Jarlenski, M. P. : Cooper, L. A.	2013	How does physician BMI impact patient trust and perceived stigma?	Preventive Medicine
Blixen, C. : Ogede, D. : Briggs, F. : Aebi, M. E. : Burant, C. : Wilson, B. : Terashima, J. P. : Sajatovic, M.	2020	Correlates of stigma in people with epilepsy	J. Clin. Neurol.
Bogart, Laura M. : Barreras, Joanna L. : Gonzalez, Ana : Klein, David J. : Marsh, Terry : Agniel, Denis : Pantalone, David W.	2021	Pilot Randomized Controlled Trial of an Intervention to Improve Coping with Intersectional Stigma and Medication Adherence Among HIV-Positive Latinx Sexual Minority Men	AIDS & Behavior
Bogart, L. M. : Matovu, J. K. B. : Wagner, G. J. : Green, H. D. : Storholm, E. D. : Klein, D. J. : Marsh, T. : MacCarthy, S. : Kambugu, A.	2020	A Pilot Test of Game Changers, a Social Network Intervention to Empower People with HIV to be Prevention Advocates in Uganda	AIDS Behav.
Bogart, L. M. : Wagner, G. J. : Galvan, F. H. : Landrine, H. : Klein, D. J. : Sticklor, L. A.	2011	Perceived discrimination and mental health symptoms among black men with HIV	Cultural Diversity and Ethnic Minority Psychology
Boland, S. E. : Street, R. L. : Persky, S.	2019	Weight-related genomic information and provider communication approach: Looking through the lens of patient race	Personalized Medicine
Bond, V. : Floyd, S. : Fenty, J. : Schaap, A. : Godfrey-Faussett, P. : Claassens, M. : Shanaube, K. : Ayles, H. : Hargreaves, J. R.	2017	Secondary analysis of tuberculosis stigma data from a cluster randomised trial in Zambia and South Africa (ZAMSTAR)	The international journal of tuberculosis and lung disease : the official journal of the International Union against Tuberculosis and Lung Disease
Bonetto, C. : Pace, D. : Bodini, L. : Colombi, M. : Van Bortel, T. : Lasalvia, A.	2022	Development and psychometric validation of new questionnaires assessing experienced discrimination and internalised stigma among people with Covid-19	Epidemiology and Psychiatric Sciences
Borecki, L. : Gozdzik-Zelazny, A. : Pokorski, M.	2010	Personality and perception of stigma in psychiatric patients with depressive disorders	European Journal of Medical Research
Botchway, M. : Davis, R. E. : Merchant, A. T. : Appiah, L. T. : Moore, S.	2021	Diabetes-related stigma and its influence on social networks, social support, and HbA1c in Ghana	Ethn. Dis.
Botero-Rodríguez, F. : Hernandez, M. C. : Uribe-Restrepo, J. M. : Cabariqe, C. : Fung, C. : Priebe, S. : Gómez-Restrepo, C.	2021	Experiences and outcomes of group volunteer befriending with patients with severe mental illness: an exploratory mixed-methods study in Colombia	BMC Psychiatry
Botha, U. A. : Koen, L. : Niehaus, D. J. H.	2006	Perceptions of a South African schizophrenia population with regards to community attitudes towards their illness	Social Psychiatry and Psychiatric Epidemiology
Bovell-Ammon, B. J. : Kimmel, S. D. : Cheng, D. M. : Truong, V. : Michals, A. : Vetrova, M. : Hook, K. : Idrisov, B. : Blokhina, E. : Krupitsky, E. : Samet, J. H. : Lunze, K.	2023	Incarceration history, antiretroviral therapy, and stigma: A cross-sectional study of people with HIV who inject drugs in St. Petersburg, Russia	International Journal of Drug Policy
Boyd, Jennifer E. : Otilingam, Poorni G. : DeForge, Bruce R.	2014	Brief Version of the Internalized Stigma of Mental Illness (ISMI) Scale: Psychometric Properties and Relationship to Depression, Self Esteem, Recovery Orientation, Empowerment, and Perceived Devaluation and Discrimination	Psychiatric Rehabilitation Journal
Boyes, M. E. : Cluver, L. D. : Meinck, F. : Casale, M. : Newnham, E.	2019	Mental health in South African adolescents living with HIV: correlates of internalising and externalising symptoms	AIDS Care - Psychological and Socio-Medical Aspects of AIDS/HIV
Boyes, M. E. : Pantelic, M. : Casale, M. : Toska, E. : Newnham, E. : Cluver, L. D.	2020	Prospective associations between bullying victimisation, internalised stigma, and mental health in South African adolescents living with HIV	Journal of Affective Disorders
Boyle, M. P. : Fearon, A. N.	2018	Self-stigma and its associations with stress, physical health, and health care satisfaction in adults who stutter	J. Fluency Disord.
Bozdağ, N. : Çuhadar, D.	2022	Internalized stigma, self-efficacy and treatment motivation in patients with substance use disorders	Journal of Substance Use
Bozinoff, Nikki : Anderson, Bradley J. : Bailey, Genie L. : Stein, Michael D.	2018	Correlates of Stigma Severity Among Persons Seeking Opioid Detoxification	Journal of addiction medicine
Brain, C. : Sameby, B. : Allerby, K. : Quinlan, P. : Joas, E. : Lindström, E. : Burns, T. : Waern, M.	2014	Stigma, discrimination and medication adherence in schizophrenia: Results from the Swedish COAST study	Psychiatry Research
Brandelli Costa, A. : de Moura Filho, J. B. : Silva, J. M. : Beloqui, J. A. : Espindola, Y. : de Araujo, C. F. : Aloia, S. A. V. : de Lima, C. E.	2022	Key and general population HIV-related stigma and discrimination in HIV-specific health care settings: results from the Stigma Index Brazil	AIDS Care - Psychological and Socio-Medical Aspects of AIDS/HIV
Brener, L. : Broady, T. : Cama, E. : Hopwood, M. : de Wit, J. B. F. : Treloar, C.	2020	The role of social support in moderating the relationship between HIV centrality, internalised stigma and psychological distress for people living with HIV	AIDS Care - Psychological and Socio-Medical Aspects of AIDS/HIV
Brener, L. : Wilson, H. : Jackson, L. C. : Johnson, P. : Saunders, V. : Treloar, C.	2016	Experiences of diagnosis, care and treatment among Aboriginal people living with hepatitis C	Aust. New Zealand J. Public Health
Breuer, L. : Barker, C.	2015	Online Support Groups for Depression: Benefits and Barriers	SAGE Open
Brewer, R. : Hood, K. B. : Moore, M. : Spieldenner, A. : Daunis, C. : Mukherjee, S. : Smith-Davis, M. : Brown, G. : Bowen, B. : Schneider, J. A.	2020	An Exploratory Study of Resilience, HIV-Related Stigma, and HIV Care Outcomes Among Men who have Sex with Men (MSM) Living with HIV in Louisiana	AIDS Behav.
Bricker, Jonathan B. : Watson, Noreen L. : Heffner, Jaimee L. : Sullivan, Brianna : Mull, Kristin : Kwon, Diana : Westmaas, Johann Lee : Ostroff, Jamie	2020	A Smartphone App Designed to Help Cancer Patients Stop Smoking: Results From a Pilot Randomized Trial on Feasibility, Acceptability, and Effectiveness	JMIR formative research
Brizzi, K. : Deki, S. : Tshering, L. : Clark, S. J. : Nirola, D. K. : Patenaude, B. N. : McKenzie, E. D. : McLane, H. C. : Casha, S. S. : Dorji, C. : Mateena, F. J.	2016	Knowledge, attitudes and practices regarding epilepsy in the Kingdom of Bhutan	International Health
Broersma, F. : Oeseburg, B. : Dijkstra, J. : Wynia, K.	2018	The impact of self-perceived limitations, stigma and sense of coherence on quality of life in multiple sclerosis patients: results of a cross-sectional study	Clinical rehabilitation
Brohan, E. : Thornicroft, G. : Rüsch, N. : Lasalvia, A. : Campbell, M. M. : Yalçınkaya-Alkar, Ö : Lanfredi, M. : Ochoa, S. : Üçok, A. : Tomás, C. : Fadipe, B. : Sebes, J. : Fiorillo, A. : Sampogna, G. : Paula, C. S. : Valverde, L. : Schomerus, G. : Klemm, P. : Ouali, U. : Castelein, S. : Alexová, A. : Oexle, N. : Guimarães, P. N. : Sportel, B. E. : Chang, C. C. : Li, J. : Shanthi, C. : Reneses, B. : Bakolis, I. : Evans-Lacko, S.	2022	Measuring discrimination experienced by people with a mental illness: replication of the short-form DISCUS in six world regions	Psychological medicine
Brooke-Sumner, C. : Selohilwe, O. : Mazibuko, M. S. : Petersen, I.	2018	Process Evaluation of a Pilot Intervention for Psychosocial Rehabilitation for Service Users with Schizophrenia in North West Province, South Africa	Community Ment. Health J.
Brooks, D. N. : Hallam, R. S.	1998	Attitudes to hearing difficulty and hearing aids and the outcome of audiological rehabilitation	British Journal of Audiology
Brown, H. W. : Wise, M. : Westenberg, D. : Schmuhl, N. B. : Brezoczky, K. L. : Rogers, R. G. : Constantine, M. L.	2016	Validation of an instrument toassess barriers to care-seeking for accidental bowel leakage	Female Pelvic Medicine and Reconstructive Surgery
Brown, M. J. : Serovich, J. M. : Kimberly, J. A. : Hu, J.	2016	Psychological reactance and HIV-related stigma among women living with HIV	AIDS Care Psychol. Socio-Med. Asp. AIDS HIV
Brunette, M. F. : Mueser, K. T. : Babbin, S. : Meyer-Kalos, P. : Rosenheck, R. : Correll, C. U. : Cather, C. : Robinson, D. G. : Schooler, N. R. : Penn, D. L. : Addington, J. : Estroff, S. E. : Gottlieb, J. : Glynn, S. M. : Marcy, P. : Robinson, J. : Kane, J. M.	2018	Demographic and clinical correlates of substance use disorders in first episode psychosis	Schizophr. Res.
Bu, Xiaofan : Li, Shuangshuang : Cheng, Andy S. K. : Ng, Peter H. F. : Xu, Xianghua : Xia, Yimin : Liu, Xiangyu	2022	Breast Cancer Stigma Scale: A Reliable and Valid Stigma Measure for Patients With Breast Cancer	Frontiers in psychology
Buchman-Wildbaum, T. : Váradi, E. : Schmelowszky, Á : Griffiths, M. D. : Demetrovics, Z. : Urbán, R.	2020	The paradoxical role of insight in mental illness: The experience of stigma and shame in schizophrenia, mood disorders, and anxiety disorders	Archives of psychiatric nursing
Buchman-Wildbaum, T. : Váradi, E. : Schmelowszky, Á : Griffiths, M. D. : Demetrovics, Z. : Urbán, R.	2020	Targeting the problem of treatment non-adherence among mentally ill patients: The impact of loss, grief and stigma	Psychiatry Research
Buck, D. : Jacoby, A. : Baker, G. A. : Chadwick, D. W.	1997	Factors influencing compliance with antiepileptic drug regimes	SEIZURE
Budhwani, H. : Robles, G. : Starks, T. J. : MacDonell, K. K. : Dinaj, V. : Naar, S.	2021	Healthy Choices Intervention is Associated with Reductions in Stigma Among Youth Living with HIV in the United States (ATN 129)	AIDS Behav.
Budhwani, H. : Yigit, I. : Ofotokun, I. : Konkle-Parker, D. J. : Cohen, M. H. : Wingood, G. M. : Metsch, L. R. : Adimora, A. A. : Taylor, T. N. : Wilson, T. E. : Weiser, S. D. : Kempf, M. C. : Sosanya, O. : Gange, S. : Kassaye, S. : Turan, B. : Turan, J. M.	2021	Examining the Relationships between Experienced and Anticipated Stigma in Health Care Settings, Patient-Provider Race Concordance, and Trust in Providers among Women Living with HIV	AIDS Patient Care and STDs
Buer, C. S. : Henning, O. : Nakken, K. O. : Lossius, M.	2021	People with epilepsy still feel stigmatized	Epilepsia

Burke, S. E. : Calabrese, S. K. : Dovidio, J. F. : Levina, O. S. : Uusküla, A. : Niccolai, L. M. : Abel-Ollo, K. : Heimer, R.	2015	A tale of two cities: Stigma and health outcomes among people with HIV who inject drugs in St. Petersburg, Russia and Kohtla-Järve, Estonia	Social Science and Medicine
Burnett-Zeigler, I. : Kim, H. M. : Chiang, C. : Kavanagh, J. : Zivin, K. : Rockefeller, K. : Sirey, J. A. : Kales, H. C.	2014	The association between race and gender, treatment attitudes, and antidepressant treatment adherence	International Journal of Geriatric Psychiatry
Buseh, A. G. : Kelber, S. T. : Hewitt, J. B. : Stevens, P. E. : Park, C. G.	2006	Perceived stigma and life satisfaction: experiences of urban African American men living with HIV/AIDS	International Journal of Men's Health
Busel, Aaron G. : Kelbe, Sheryl T. : Steven, Patricia E. : Park, Chang Gi	2012	Neighborhood Social Disorder and Stigma Associated with Quality of Life among Urban HIV-Positive African-American Men: A Mediation Model	Journal of National Black Nurses Association
Cabello González, C. : Trandafir, P. C.	2018	The study of health-related quality of life using PDQ39 in patients with Parkinson's disease treated with advanced therapies	Revista Cientifica de la Sociedad Espanola de Enfermeria Neurologica
Cai, C. : Yu, L.	2017	Quality of Life in Patients With Schizophrenia in China: Relationships Among Demographic Characteristics, Psychosocial Variables, and Symptom Severity	Journal of psychosocial nursing and mental health services
Cai, H. : Bai, W. : Du, X. : Zhang, L. : Zhang, L. : Li, Y. C. : Liu, H. Z. : Tang, Y. L. : Jackson, T. : Cheung, T. : An, F. R. : Xiang, Y. T.	2022	COVID-19 vaccine acceptance and perceived stigma in patients with depression: a network perspective	Transl. Psychiatry
Cai, Y. : Zhang, Y. : Cao, W. : Hou, F. : Xin, M. : Guo, V. Y. : Deng, Y. : Wang, S. : You, X. : Li, J.	2022	Preliminary validation of the Chinese version of the Shame and Stigma Scale among patients with facial disfigurement from nasopharyngeal carcinoma	PloS one
Calugi, S. : Dalle Grave, R.	2020	Psychological features in obesity: A network analysis	International Journal of Eating Disorders
Cama, Elena : Brener, Loren : Slavin, Sean : de Wit, John	2020	The relationship between negative responses to HIV status disclosure and psychosocial outcomes among people living with HIV	Journal of Health Psychology
Campbell, Duncan : Bonner, Laura : Bolkan, Cory : Lanto, Andrew : Zivin, Kara : Waltz, Thomas : Klap, Ruth : Rubenstein, Lisa : Chaney, Edmund : Campbell, Duncan G. : Bonner, Laura M. : Bolkan, Cory R. : Lanto, Andrew B. : Waltz, Thomas J. : Rubenstein, Lisa V. : Chaney, Edmund F.	2016	Stigma Predicts Treatment Preferences and Care Engagement Among Veterans Affairs Primary Care Patients with Depression	Annals of Behavioral Medicine
Campo-Arias, Adalberto : Ceballos-Ospino, Guillermo A. : Herazo, Edwin	2020	Barriers to access to mental health services among Colombia outpatients	International Journal of Social Psychiatry
Can, G. : Tanrıverdi, D.	2015	Social functioning and internalized stigma in individuals diagnosed with substance use disorder	Archives of psychiatric nursing
Cano-De-La-Cuerda, R. : Vela-Desojo, L. : Miangolarra-Page, J. C. : Macías-Macías, Y.	2014	Isokinetic dynamometry as a technologic assessment tool for trunk rigidity in Parkinson's disease patients	NeuroRehabilitation
Cantisano, N. : Rimé, B. : Munoz Sastre, M. T.	2015	The importance of quality over in quantity in the social sharing of emotions (SSE) in people living with HIV/AIDS	Psychology, health & medicine
Cantisano, N. : Rimé, B. : Afzali, M. H. : Muñoz-Sastre, M. T.	2016	Health outcomes resulting from the quality of emotional expression in HIV/AIDS patients	Rev. Eur. Psychol. Appl.
Çapar, M. : Kavak, F.	2019	Effect of internalized stigma on functional recovery in patients with schizophrenia	Perspectives in psychiatric care
Căpățînă, O. : Micluția, I.	2018	Internalized stigma as a predictor of quality of life in schizophrenia	J. Evid.-Based Psychother.
Căpățînă, O. : Pojoga, C. : Savu, B. : Fadgyas-Stănculete, M.	2021	Predictors of quality of life and functioning in schizophrenia	Cognition, Brain, Behavior. An Interdisciplinary Journal
Capistrant, B. D. : Friedemann-Sánchez, G. : Pendsey, S.	2019	Diabetes stigma, parent depressive symptoms and Type-1 diabetes glycemic control in India	Social work in health care
Caqueo-Urízar, A. : Boyer, L. : Urzúa, A. : Williams, D. R.	2019	Self-stigma in patients with schizophrenia: a multicentric study from three Latin-America countries	Social psychiatry and psychiatric epidemiology
Caqueo-Urízar, A. : Ponce-Correa, F. : Semir-González, C. : Urzúa, A.	2022	Latent Profiles of Premorbid Adjustment in Schizophrenia and Their Correlation with Measures of Recovery	Journal of Clinical Medicine
Caqueo-Urízar, A. : Urzúa, A. : Loundon, A. : Boucekine, M. : Fond, G. : Boyer, L.	2019	The latin american version of the internalized stigma of mental illness scale (la-ismi): A multicentric validation study from three latin american countries	Health and Quality of Life Outcomes
Caqueo-Urizar, Alejandra : Urzua, Alfonso : Habib, Julia : Loundou, Anderson : Boucekine, Mohamed : Boyer, Laurent : Fond, Guillaume	2020	Relationships between social stigma, stigma experience and self-stigma and impaired quality of life in schizophrenia across three Latin-American countries	European archives of psychiatry and clinical neuroscience
Cardarelli, R. : Weis, S. : Adams, E. : Radaford, D. : Vecino, I. : Munguia, G. : Johnson, K. L. : Fulda, K. G.	2008	General health status and adherence to antiretroviral therapy	J. Int. Assoc. Phys. AIDS
Caricati, L. : Soli, B.	2022	Factor structure of an Italian adaptation of the Perceived Stigma Scale in Inflammatory Bowel Disease (PSS-IBD-Iv): A preliminary investigation	Psicol. Salute
Carlozzi, N. E. : Goodnight, S. : Kratz, A. L. : Stout, J. C. : McCormack, M. K. : Paulsen, J. S. : Boileau, N. R. : Cella, D. : Ready, R. E.	2019	Validation of Neuro-QoL and PROMIS Mental Health Patient Reported Outcome Measures in Persons with Huntington Disease	Journal of Huntington's Disease
Carlucci, J. G. : Kamanga, A. : Sheneberger, R. : Shepherd, B. E. : Jenkins, C. A. : Spurrier, J. : Vermund, S. H.	2008	Predictors of adherence to antiretroviral therapy in rural Zambia	Journal of Acquired Immune Deficiency Syndromes
Carod-Artal, F. J. : Martinez-Martin, P. : Vargas, A. P.	2007	Independent validation of SCOPA-psychosocial and metric properties of the PDQ-39 Brazilian version	Movement Disorders
Carrizosa, C. M. : Blumberg, E. J. : Hovell, M. F. : Martinez-Donate, A. P. : Garcia-Gonzalez, G. : Lozada, R. : Kelley, N. J. : Hofstetter, C. R. : Sipan, C. L.	2010	Determinants and prevalence of late HIV testing in Tijuana, Mexico	AIDS Patient Care & STDs
Carter-Harris, L.	2015	Lung cancer stigma as a barrier to medical help-seeking behavior: Practice implications	Journal of the American Association of Nurse Practitioners
Carter-Harris, L. : Hall, L. A.	2014	Development of a short version of the cataldo lung cancer stigma scale	Journal of Psychosocial Oncology
Carter-Harris, Lisa : Hermann, Carla P. : Schreiber, Judy : Weaver, Michael T. : Rawl, Susan M.	2014	Lung Cancer Stigma Predicts Timing of Medical Help-Seeking Behavior	Oncology Nursing Forum
Casanovas Taltavull, T. : Jané Cabré, L. : Herdman, M. : Casado Collado, A. : Pubill, B. P. : Fabregat Prous, J.	2007	Validation of the Spanish Version of the Liver Disease Quality of Life Instrument Among Candidates for Liver Transplant	Transplantation Proceedings
Casillas, J. N. : Schwartz, L. F. : Gildner, J. L. : Crespi, C. M. : Ganz, P. A. : Kahn, K. L. : Stuber, M. L. : Bastani, R. : Morales, S. : Quintana, K. : Gonzalez, A. : Casas, E. : Barboa, E.	2021	Engaging Latino Adolescent and Young Adult (AYA) Cancer Survivors in Their Care: Piloting a Photonovela Intervention	Journal of cancer education : the official journal of the American Association for Cancer Education
Castañeda-Daniels, N. P. : Campo-Arias, A. : Pedrozo-Pupo, J. C.	2021	Assessment of validity and reliability of the Tuberculosis Related Stigma Scale in Colombian patients	medRxiv
Castañeda-Daniels, N. P. : Campo-Arias, A. : Pedrozo-Pupo, J. C.	2022	Assessment of validity and reliability of the tuberculosis related stigma scale in Colombian patients	Current Medical Research and Opinion
Castillo-Triviño, T. : Gómez-Ballesteros, R. : Borges, M. : Martín-Martínez, J. : Sotoca, J. : Alonso, A. : Caminero, A. B. : Borrega, L. : Sánchez-Menoyo, J. L. : Barrero-Hernández, F. J. : Calles, C. : Brieva, L. : Blasco-Quílez, M. R. : García-Soto, J. D. : del Campo-Amigo, M. : Navarro-Cantó, L. : Agüera, E. : Garcés-Redondo, M. : Carmona, O. : Gabaldón-Torres, L. : Forero, L. : Hervás, M. : Mauriño, J. : Sainz de la Maza, S.	2022	Long-term prognosis communication preferences in early-stage relapsing-remitting multiple sclerosis	Multiple Sclerosis and Related Disorders
Cataldo, J. K.	2013	Lung cancer stigma, anxiety, depression and symptom severity	Journal of Thoracic Oncology
Cataldo, J. K. : Slaughter, R. : Jahan, T. M. : Pongquan, V. L. : Hwang, W. J.	2011	Measuring stigma in people with lung cancer: Psychometric testing of the Cataldo Lung Cancer Stigma Scale	Oncology Nursing Forum
Catthoor, K. : Feenstra, D. J. : Hutsebaut, J. : Schrijvers, D. : Sabbe, B.	2015	Adolescents with personality disorders suffer from severe psychiatric stigma: Evidence from a sample of 131 patients	Adolescent Health, Medicine and Therapeutics
Catthoor, K. : Schrijvers, D. : Hutsebaut, J. : Feenstra, D. : Sabbe, B.	2015	Psychiatric stigma in treatment-seeking adults with personality problems: Evidence from a sample of 214 patients	Frontiers in Psychiatry
Catucci Boza, Juliana : Giongo, Natalia : Machado, Priscilla : Horn, Roberta : Fabbrin, Amanda : Cestari, Tania	2016	Quality of Life Impairment in Children and Adults with Vitiligo: A Cross-Sectional Study Based on Dermatology-Specific and Disease-Specific Quality of Life Instruments	Dermatology (Basel, Switzerland)
Cavazos-Rehg, P. : Byansi, W. : Doroshenko, C. : Neilands, T. B. : Anako, N. : Sensoy Bahar, O. : Kasson, E. : Nabunya, P. : Mellins, C. A. : Ssewamala, F. M.	2021	Evaluating potential mediators for the impact of a family-based economic intervention (Suubi+Adherence) on the mental health of adolescents living with HIV in Uganda	Social Science and Medicine
Cavelti, M. : Kvrgic, S. : Beck, E. M. : Rüsch, N. : Vauth, R.	2012	Self-stigma and its relationship with insight, demoralization, and clinical outcome among people with schizophrenia spectrum disorders	Compr. Psychiatry
Cavelti, M. : Rüsch, N. : Vauth, R.	2014	Is living with psychosis demoralizing?: Insight, self-stigma, and clinical outcome among people with schizophrenia across 1 year	Journal of Nervous and Mental Disease
Cengiz, G. F. : Tanık, N.	2020	Who is more important in stigmatization, family or friends?	Epilepsy and Behavior
Cerit, C. : Filizer, A. : Tural, Ü : Tufan, A. E.	2012	Stigma: A core factor on predicting functionality in bipolar disorder	Comprehensive Psychiatry
Chace Dwyer, S. : Jain, A. : Liambila, W. : Warren, C. E.	2021	The role of unintended pregnancy in internalized stigma among women living with HIV in Kenya	BMC Women's Health
Chai, Y. C. : Mahadevan, R. : Ng, C. G. : Chan, L. F. : Md Dai, F.	2018	Caregiver depression: The contributing role of depression in patients, stigma, social support and religiosity	The International journal of social psychiatry
Chaka, A. : Awoke, T. : Yohannis, Z. : Ayano, G. : Tareke, M. : Abate, A. : Nega, M.	2018	Determinants of depression among people with epilepsy in Central Ethiopia	Annals of General Psychiatry
Chakrapani, V. : Kershaw, T. : Kaur, J. : Shunmugam, M. : Nelson, R. : Vijin, P. P. : Rajan, M. : Subramanian, T.	2022	Associations between sexual stigma, enacted HIV stigma, internalized HIV stigma and homonegativity, and depression: testing an extended minority stress model among men who have sex with men living with HIV in India	AIDS Care - Psychological and Socio-Medical Aspects of AIDS/HIV
Chambers, S. K. : Baade, P. : Youl, P. : Aitken, J. : Occhipinti, S. : Vinod, S. : Valery, P. C. : Garvey, G. : Fong, K. M. : Ball, D. : Zorbas, H. : Dunn, J. : O'Connell, D. L.	2015	Psychological distress and quality of life in lung cancer: The role of health-related stigma, illness appraisals and social constraints	Psycho-Oncology

Chambers, S. K. : Morris, B. A. : Clutton, S. : Foley, E. : Giles, L. : Schofield, P. : O'Connell, D. : Dunn, J.	2015	Psychological wellness and health-related stigma: a pilot study of an acceptance-focused cognitive behavioural intervention for people with lung cancer	European journal of cancer care
Chan, B. T. : Pradeep, A. : Chandrasekaran, E. : Prasad, L. : Murugesan, V. : Kumarasamy, N. : Mayer, K. H. : Tsai, A. C.	2019	Reliability, Validity, and Factor Structure of the Internalized AIDS-Related Stigma Scale in Southern India	Journal of the International Association of Providers of AIDS Care
Chan, K. K. S. : Fung, W. T. W.	2019	The impact of experienced discrimination and self-stigma on sleep and health-related quality of life among individuals with mental disorders in Hong Kong	Quality of Life Research
Chan, Kevin Ka Shing : Fung, Winnie Tsz Wa : Leung, Donald Chi Kin : Tsui, Jack Ka Chun	2022	The impact of perceived and internalised stigma on clinical and functional recovery among people with mental illness	Health & Social Care in the Community
Chan, R. C. H. : Mak, W. W. S.	2016	Common sense model of mental illness: Understanding the impact of cognitive and emotional representations of mental illness on recovery through the mediation of self-stigma	Psychiatry Research
Chan, R. C. H. : Mak, W. W. S.	2019	Cognitive, Regulatory, and Interpersonal Mechanisms of HIV Stigma on the Mental and Social Health of Men Who Have Sex With Men Living With HIV	Am. J. Men's Health
Chan, S. K. W. : Kao, S. Y. S. : Leung, S. L. : Hui, C. L. M. : Lee, E. H. M. : Chang, W. C. : Chen, E. Y. H.	2019	Relationship between neurocognitive function and clinical symptoms with self-stigma in patients with schizophrenia-spectrum disorders	Journal of Mental Health
Chang, C. C. : Chang, K. C. : Hou, W. L. : Yen, C. F. : Lin, C. Y. : Potenza, M. N.	2020	Measurement invariance and psychometric properties of Perceived Stigma toward People who use Substances (PSPS) among three types of substance use disorders: Heroin, amphetamine, and alcohol	Drug and Alcohol Dependence
Chang, C. C. : Wu, T. H. : Chen, C. Y. : Wang, J. D. : Lin, C. Y.	2014	Psychometric evaluation of the internalized stigma of mental illness scale for patients with mental illnesses: Measurement invariance across time	PLoS ONE
Chang, C. C. : Wu, T. H. : Chen, C. Y. : Lin, C. Y.	2016	Comparing Self-stigma between People with Different Mental Disorders in Taiwan	Journal of Nervous and Mental Disease
Chang, C. : Wang, T. J. : Chen, M. J. : Liang, S. Y. : Wu, S. F. : Bai, M. J.	2021	Factors influencing readiness to change in patients with alcoholic liver disease: A cross-sectional study	Journal of psychiatric and mental health nursing
Chang, K. C. : Chen, H. P. : Huang, S. W. : Chen, J. S. : Potenza, M. N. : Pakpour, A. H. : Lin, C. Y.	2022	Comparisons of psychological distress and self-stigma among three types of substance use disorders receiving treatment-as-usual approaches: real-world data from a 9-month longitudinal study	Therapeutic Advances in Chronic Disease
Chang, K. C. : Strong, C. : Pakpour, A. H. : Griffiths, M. D. : Lin, C. Y.	2020	Factors related to preventive COVID-19 infection behaviors among people with mental illness	Journal of the Formosan Medical Association
Chang, Y. H. : Chang, K. C. : Hou, W. L. : Lin, C. Y. : Griffiths, M. D.	2021	Internet gaming as a coping method among schizophrenic patients facing psychological distress	Journal of Behavioral Addictions
Chapman, Lambert Crystal Msph C. P. A. C. : Fazeli, Pariya L. : Yigit, Ibrahim : Elopre, Latesha Msph : King, Kiko : Kempf, Mirjam-Colette : Guthrie, Barbara Faan : Mugavero, Michael J. MHSc	2022	The Mediating Role of Social Support and Resilience Between HIV-Related Stigmas and Patient Activation Among Young Black Women Living With HIV in the Southern United States: A Cross-sectional Study	JANAC: Journal of the Association of Nurses in AIDS Care
Charles, H. : Manoranjitham, S. D. : Jacob, K. S.	2007	Stigma and explanatory models among people with schizophrenia and their relatives in Vellore, South India	International Journal of Social Psychiatry
Chaudhary, R. G. : Changhulani, A. R. : Malhotra, S. D. : Parikh, N. R. : Shah, M. J. : Chaudhary, A. R. : Chaudhary, A. R.	2021	Dermatology Life Quality Index and Social Stigma among Patients of Hansen's Disease	Indian Journal of Leprosy
Chee, C. Y. I. : Ng, T. P. : Kua, E. H.	2005	Comparing the stigma of mental illness in a general hospital with a state mental hospital: A Singapore study	Social Psychiatry and Psychiatric Epidemiology
Chekole, Y. A. : Tarekegn, D.	2021	HIV-related perceived stigma and associated factors among patients with HIV, Dilla, Ethiopia: A cross-sectional study	Annals of Medicine and Surgery
Chemhaka, G. B. : Simelane, M. S. : Moyo, S. : Shongwe, M. C.	2021	Prevalence and sociodemographic determinants of alcohol consumption among adults living with HIV in Eswatini	African Journal of AIDS Research
Chen, C. : Baral, S. : Comins, C. A. : McIngana, M. : Wang, L. : Phetlhu, D. R. : Mulumba, N. : Guddera, V. : Young, K. : Mishra, S. : Hausler, H. : Schwartz, S. R.	2022	HIV- and sex work-related stigmas and quality of life of female sex workers living with HIV in South Africa: a cross-sectional study	BMC Infectious Diseases
Chen, C. : Gonzales, L.	2022	Understanding weight stigma in eating disorder treatment: Development and initial validation of a treatment-based stigma scale	Journal of health psychology
Chen, C. : Huang, J. : Zhao, J. : Zhang, Y. : Yu, N. X.	2020	Resilience moderated the predictive effect of dual stigma on distress among chinese newly diagnosed hiv-positive men who have sex with men	AIDS Education and Prevention
Chen, Emily : Chang, Wing : Hui, Christy : Chan, Sherry : Lee, Edwin : Chen, Eric : Chen, Emily S. M. : Chang, Wing Chung : Hui, Christy L. M. : Chan, Sherry K. W. : Lee, Edwin Ho Ming : Chen, Eric Y. H.	2016	Self-stigma and affiliate stigma in first-episode psychosis patients and their caregivers	Social Psychiatry & Psychiatric Epidemiology
Chen, Xu : Du, Liang : Wu, Ruiheng : Xu, Jia : Ji, Haoqiang : Zhang, Yu : Zhu, Xuexue : Zhou, Ling	2021	Tuberculosis-related stigma and its determinants in Dalian, Northeast China: a cross-sectional study	BMC Public Health
Chen, X. : Liao, Z. : Huang, S. : Huang, Q. : Lin, S. : Li, Y. : Shao, T. : Tang, Y. : Hao, J. : Qi, J. : Cai, Y. : Wang, M. : Shen, H.	2022	Stigmatizing Attitudes Toward COVID-19 Among Patients, Their Relatives and Healthy Residents in Zhangjiajie	Frontiers in public health
Chen, Xiaoyun : Mao, Yixuan : Kong, Linghua : Li, Guopeng : Xin, Minglan : Lou, Fenglan : Li, Ping	2016	Resilience moderates the association between stigma and psychological distress among family caregivers of patients with schizophrenia	Personality and Individual Differences
Chen, Y. L. : Chang, C. C. : Chen, Y. M. : Liu, T. L. : Hsiao, R. C. : Chou, W. J. : Yen, C. F.	2021	Association between affiliate stigma and depression and its moderators in caregivers of children with attention-deficit/hyperactivity disorder	Journal of Affective Disorders
Cheong, K. C. : Ghazali, S. M. : Zamri, A. S. S. M. : Cheong, Y. L. : Iderus, N. H. M. : Nagalingam, T. : Ruslan, Q. : Omar, M. A. : Yusoff, A. F.	2022	Gender Differences in Factors Associated with the Total Delay in Treatment of Pulmonary Tuberculosis Patients: A Cross-Sectional Study in Selangor, Malaysia	International Journal of Environmental Research and Public Health
Chiang, S. : Skolnick, G. : Naidoo, S. : Smyth, M. : Patel, K.	2022	Outcomes of Endoscopic Repair for Syndromic Craniosynostosis	Cleft Palate-Craniofacial Journal
Chiang, Y. S. : Chang, Y. C. : Liu, Y. P. : Tzeng, W. C.	2021	Quality of life in patients with comorbid serious mental illness and chronic diseases: A structural equation model	Journal of advanced nursing
Chien, W. T. : Chan, S. W. C. : Yeung, F. K. K. : Chiu, H. F. K. : Ng, B. F. L.	2015	Perceived stigmatisation of patients with mental illness and its psychosocial correlates: A prospective cohort study	Hong Kong Medical Journal
Chien, Wai - Tong : Lam, Claire K. K. : Ng, Bacon F. L.	2015	Predictors of levels of functioning among Chinese people with severe mental illness: a 12-month prospective cohort study	Journal of Clinical Nursing (John Wiley & Sons, Inc.)
Chime, O. H. : Arinze-Onyia, S. U. : Ossai, E. N.	2019	Examining the effect of peer-support on self-stigma among persons living with hiv/aids	Pan African Medical Journal
Chin, E. D. : Armstrong, D.	2019	Anticipated stigma and healthcare utilization in COPD and neurological disorders	Appl. Nurs. Res.
Chircop, C. : Dingli, N. : Aquilina, A. : Zrinzo, L. : Aquilina, J.	2018	MRI-verified "asleep" deep brain stimulation in Malta through cross border collaboration: clinical outcome of the first five years	British Journal of Neurosurgery
Cho, J. : Choi, E. K. : Kim, S. Y. : Shin, D. W. : Cho, B. L. : Kim, C. H. : Koh, D. H. : Guallar, E. : Bardwell, W. A. : Park, J. H.	2013	Association between cancer stigma and depression among cancer survivors: A nationwide survey in Korea	Psycho-Oncology
Cho, Sung Eun : Kwon, Myoungjin : Kim, Sun Ae	2022	Influence of Diabetes Knowledge, Self-Stigma, and Self-Care Behavior on Quality of Life in Patients with Diabetes	Healthcare (2227-9032)
Cho, S. : Ryu, E.	2021	The mediating effect of resilience on happiness of advanced lung cancer patients	Supportive Care Cancer
Choi, P. : Kavasery, R. : Desai, M. M. : Kamarulzaman, A. : Altice, F. L.	2010	Prevalence and correlates of community re-entry challenges faced by HIV-infected male prisoners in Malaysia	International Journal of STD and AIDS
Chong, Eddie S. K : Mak, Winnie W. S : Tam, Terence C. Y : Zhu, Chen : Chung, Rita W. Y	2017	Impact of perceived HIV stigma within men who have sex with men community on mental health of seropositive MSM	AIDS Care
Chowdhury, A. N. : Sanyal, D. : Bhattacharya, A. : Dutta, S. K. : De, R. : Banerjee, S. : Bhattacharya, K. : Palit, S. : Bhattacharya, P. : Mondal, R. K. : Weiss, M. G.	2001	Prominence of Symptoms and Level of Stigma among Depressed Patients in Calcutta	J. Indian Med. Assoc.
Christopoulos, K. A. : Neilands, T. B. : Hartogensis, W. : Geng, E. H. : Sauceda, J. : Mugavero, M. J. : Crane, H. M. : Fredericksen, R. J. : Moore, R. D. : Mathews, W. C. : Mayer, K. H. : Chander, G. : Hurt, C. B. : Johnson, M. O.	2019	Internalized HIV Stigma Is Associated with Concurrent Viremia and Poor Retention in a Cohort of US Patients in HIV Care	Journal of Acquired Immune Deficiency Syndromes
Christopoulos, K. A. : Neilands, T. B. : Dilworth, S. : Lisha, N. : Sauceda, J. : Mugavero, M. J. : Crane, H. M. : Fredericksen, R. J. : Mathews, W. C. : Moore, R. D. : Mayer, K. H. : Napravnik, S. : Johnson, M. O.	2020	Internalized HIV stigma predicts subsequent viremia in US HIV patients through depressive symptoms and antiretroviral therapy adherence	AIDS (London, England)
Chuang, S. P. : Wu, J. Y. W. : Wang, C. S.	2019	Self-perception of mental illness, and subjective and objective cognitive functioning in people with schizophrenia	Neuropsychiatric Disease and Treatment
Chung, Ka-Fai : Tse, Samson : Lee, Chit-Tat : Wong, Michael Ming-Cheuk : Chan, Wing-Man	2019	Experience of stigma among mental health service users in Hong Kong: Are there changes between 2001 and 2017?	International Journal of Social Psychiatry

Cinculova, A. : Prasko, J. : Kamaradova, D. : Ociskova, M. : Latalova, K. : Vrbova, K. : Kubinek, R. : Mainerova, B. : Grambal, A. : Tichackova, A.	2017	Adherence, self-stigma and discontinuation of pharmacotherapy in patients with anxiety disorders - cross-sectional study	Neuroendocrinology Letters
Clark, H. J. : Lindner, G. : Armistead, L. : Austin, B.	2003	Stigma, disclosure, and psychological functioning among HIV-infected and non-infected African-American women	Women & Health
Clement, Sarah : Brohan, Elaine : Jeffery, Debra : Henderson, Claire : Hatch, Stephani L. : Thornicroft, Graham	2012	Development and psychometric properties the Barriers to Access to Care Evaluation scale (BACE) related to people with mental ill health	BMC Psychiatry
Clement, S. : Williams, P. : Farrelly, S. : Hatch, S. L. : Schauman, O. : Jeffery, D. : Henderson, R. C. : Thornicroft, G.	2015	Mental health-related discrimination as a predictor of low engagement with mental health services	Psychiatr. Serv.
Closson, K. : Palmer, A. K. : Collins, A. B. : Salters, K. : Zhang, W. : Montaner, J. S. G. : Hogg, R. S. : Parashar, S.	2018	Factors associated with low neighborhood cohesion among women living with HIV impacted by social-structural inequities in British Columbia	AIDS Care - Psychological and Socio-Medical Aspects of AIDS/HIV
Cococcia, S. : Lenti, M. V. : Mengoli, C. : Klersy, C. : de Andreis, F. B. : Secco, M. : Ghorayeb, J. : Delliponti, M. : Corazza, G. R. : Di Sabatino, A.	2021	Validation of the Italian translation of the perceived stigma scale and resilience assessment in inflammatory bowel disease patients	World Journal of Gastroenterology
Colbert, A. M. : Kim, K. H. : Sereika, S. M. : Erlen, J. A.	2010	An examination of the relationships among gender, health status, social support, and HIV-related stigma	JANAC: Journal of the Association of Nurses in AIDS Care
Collier, S. : Singh, R. : Semeere, A. : Byakwaga, H. : Laker-Oketta, M. : McMahon, D. E. : Chemtai, L. : Grant, M. : Butler, L. : Bogart, L. : Bassett, I. V. : Kiprono, S. : Maurer, T. : Martin, J. : Busakhala, N. : Freeman, E. E.	2022	Telling the story of intersectional stigma in HIV-associated Kaposi's sarcoma in western Kenya: a convergent mixed-methods approach	Journal of the International AIDS Society
Conner, K. O. : Copeland, V. C. : Grote, N. K. : Koeske, G. : Rosen, D. : Reynolds, C. F. : Brown, C.	2010	Mental health treatment seeking among older adults with Depression: The impact of stigma and race	Am. J. Geriatr. Psychiatry
Conner, K. O. : McKinnon, S. A. : Ward, C. J. : Reynolds, C. F. : Brown, C.	2015	Peer Education as a Strategy for Reducing Internalized Stigma among Depressed Older Adults	Psychiatr. Rehab. J.
Cook, Jonathan E. : Germano, Adriana L. : Stadler, Gertraud	2016	An Exploratory Investigation of Social Stigma and Concealment in Patients with Multiple Sclerosis	International journal of MS care
Cook, Paul F. : Hartson, Kimberly R. : Schmiege, Sarah J. : Jankowski, Catherine : Starr, Whitney : Meek, Paula	2016	Bidirectional Relationships Between Fatigue and Everyday Experiences in Persons Living With HIV	Research in Nursing & Health
Corallo, F. : De Cola, M. C. : Lo Buono, V. : Di Lorenzo, G. : Bramanti, P. : Marino, S.	2017	Observational study of quality of life of Parkinson's patients and their caregivers	Psychogeriatrics
Coreil, Jeannine : Lauzardo, Michael : Clayton, Heather	2010	Stigma and Therapy Completion for Latent Tuberculosis among Haitian-origin Patients	Florida public health review
Corker, Elizabeth : Brown, June : Henderson, Claire	2016	How does self stigma differ across people with psychiatric diagnoses and rheumatoid arthritis, and how does it impact on self-esteem and empowerment?	Psychology, Health & Medicine
Corker, E. : Henderson, R. C. : Lempp, H. : Brown, J. S. L.	2016	Internalised stigma in people with rheumatoid arthritis: A cross sectional study to establish the psychometric properties of the ISMI-RA	BMC Musculoskeletal Disorders
Corless, I. B. : Hoyt, A. J. : Tyer-Viola, L. : Sefcik, E. : Kemppainen, J. : Holzemer, W. L. : Eller, L. S. : Nokes, K. : Phillips, J. C. : Dawson-Rose, C. : Rivero-Mendez, M. : lipinge, S. : Chaiphibalsarisdi, P. : Portillo, C. J. : Chen, W. T. : Webel, A. R. : Brion, J. : Johnson, M. O. : Voss, J. : Hamilton, M. J. : Sullivan, K. M. : Kirksey, K. M. : Nicholas, P. K.	2017	90-90-90-Plus: Maintaining Adherence to Antiretroviral Therapies	AIDS Patient Care and STDs
Corrigan, P. W. : Michaels, P. J. : Powell, K. : Bink, A. : Sheehan, L. : Schmidt, A. : Apa, B. : Al-Khouja, M.	2016	Who comes out with their mental illness and how does it help?	J. Nerv. Ment. Dis.
Corrigan, Patrick W. : Nieweglowski, Katherine	2021	Difference as an indicator of the self-stigma of mental illness	Journal of Mental Health
Costain, G. : Esplen, M. J. : Toner, B. : Scherer, S. W. : Meschino, W. S. : Hodgkinson, K. A. : Bassett, A. S.	2014	Evaluating genetic counseling for individuals with schizophrenia in the molecular age	Schizophrenia Bulletin
Costelloe, Stephanie : Kemppainen, Jeanne : Brion, John : MacKain, Sally : Reid, Paula : Frampton, Art : Rigsbee, Elizabeth	2015	Impact of anxiety and depressive symptoms on perceptions of stigma in persons living with HIV disease in rural versus urban North Carolina	AIDS Care
Cox, C. K. : Schimpf, M. O. : Berger, M. B.	2021	Stigma Associated with Pelvic Floor Disorders	Female Pelvic Medicine and Reconstructive Surgery
Cramer, R. J. : Burks, A. C. : Plöderl, M. : Durgampudi, P.	2017	Minority stress model components and affective well-being in a sample of sexual orientation minority adults living with HIV/AIDS	AIDS Care - Psychological and Socio-Medical Aspects of AIDS/HIV
Cramer, R. J. : Colbourn, S. L. : Gemberling, T. M. : Graham, J. : Stroud, C. H.	2015	Substance-related coping, HIV-related factors, and mental health among an HIV-positive sexual minority community sample	AIDS Care Psychol. Socio-Med. Asp. AIDS HIV
Crişan, Cătălin : Vlasin, Nicoleta : Dutchievici, Irina : Nemeş, Andrea : Micluţia, Ioana	2016	Awareness of illness, depression and self-stigma in Romanian patients with schizophrenia	Cognition, Brain, Behavior: An Interdisciplinary Journal
Criswell, K. R. : Owen, J. E. : Thornton, A. A. : Stanton, A. L.	2016	Personal responsibility, regret, and medical stigma among individuals living with lung cancer	J. Behav. Med.
Crocker, H. : Jenkinson, C. : Peters, M.	2018	Quality of life in coeliac disease: item reduction, scale development and psychometric evaluation of the Coeliac Disease Assessment Questionnaire (CDAQ)	Alimentary Pharmacology and Therapeutics
Crockett, Kaylee B. : Kalichman, Seth C. : Kalichman, Moira O. : Cruess, Dean G. : Katner, Harold P.	2019	Experiences of HIV-related discrimination and consequences for internalised stigma, depression and alcohol use	Psychology & Health
Çuhadar, Döndü : Olcay Çam, M.	2014	Effectiveness of Psychoeducation in Reducing Internalized Stigmatization in Patients With Bipolar Disorder	Archives of Psychiatric Nursing
Cullen, B. A. : Mojtabai, R. : Bordbar, E. : Everett, A. : Nugent, K. L. : Eaton, W. W.	2017	Social network, recovery attitudes and internal stigma among those with serious mental illness	The International journal of social psychiatry
da Silva, A. G. : Leal, V. P. : da Silva, P. R. : Freitas, F. C. : Linhares, M. N. : Walz, R. : Malloy-Diniz, L. F. : Diaz, A. P. : Palha, A. P.	2020	Difficulties in activities of daily living are associated with stigma in patients with parkinson's disease who are candidates for deep brain stimulation	Brazilian Journal of Psychiatry
Da Silva, N. : Augustin, M. : Hilbring, C. : Braren-Von Stulpnagel, C. C. : Sommer, R.	2022	Psychological (co)morbidity in patients with psoriasis: The impact of pruritus and anogenital involvement on symptoms of depression and anxiety and on body dysmorphic concerns - A cross-sectional study	BMJ Open
Dafsari, H. S. : Reker, P. : Stalinski, L. : Silverdale, M. : Rizos, A. : Ashkan, K. : Barbe, M. T. : Fink, G. R. : Evans, J. : Steffen, J. : Samuel, M. : Dembek, T. A. : Visser-Vandewalle, V. : Antonini, A. : Ray-Chaudhuri, K. : Martinez-Martin, P. : Timmermann, L.	2018	Quality of life outcome after subthalamic stimulation in Parkinson's disease depends on age	Movement Disorders
Dahiru, T. : Iliyasu, Z. : Aliyu, M. H.	2022	Social participation restriction among persons with leprosy discharged from a multidrug therapy clinic in northern Nigeria	Trans. R. Soc. Trop. Med. Hyg.
Dai, Z. : Xiao, W. : Wang, H. : Wu, Y. : Huang, Y. : Si, M. : Fu, J. : Chen, X. : Jia, M. : Leng, Z. : Cui, D. : Dong, L. : Mak, W. W. S. : Su, X.	2022	Influencing factors of anxiety and depression of discharged COVID-19 patients in Wuhan, China	PLoS ONE
Dalky, Heyam F. : Gharaibeh, Huda : Faleh, Reem	2019	Psychosocial Burden and Stigma Perception of Jordanian Patients With Epilepsy	Clinical nursing research
Dalrymple, J. : Appleby, J.	2000	Cross sectional study of reporting of epileptic seizures to general practitioners	Br. Med. J.
Damant, R. W. : Rourke, L. : Cui, Y. : Lam, G. Y. : Smith, M. P. : Fuhr, D. P. : Tay, J. K. : Varughese, R. A. : Laratta, C. R. : Lau, A. : Wong, E. Y. : Stickland, M. K. : Ferrara, G.	2023	Reliability and validity of the post COVID-19 condition stigma questionnaire: A prospective cohort study	eClinicalMedicine
Danker, H. : Wollbrück, D. : Singer, S. : Fuchs, M. : Brähler, E. : Meyer, A.	2010	Social withdrawal after laryngectomy	Eur. Arch. Oto-Rhino-Laryngol.
Daryaafzoon, M. : Amini-Tehrani, M. : Zohrevandi, Z. : Hamzehlouiyan, M. : Ghotbi, A. : Zarrabi-Ajami, S. : Zamanian, H.	2020	Translation and Factor Analysis of the Stigma Scale for Chronic Illnesses 8-Item Version Among Iranian Women With Breast Cancer	Asian Pacific journal of cancer prevention : APJCP
Datta, S. : Bhattacherjee, S. : Sherpa, P. L. : Banik, S.	2016	Perceived HIV related stigma among patients attending ART center of a tertiary care center in rural West Bengal, India	Journal of Clinical and Diagnostic Research
Davila, Jessica A. : Cabral, Howard J. : Maskay, Manisha H. : Marcus, Ruthanne : Yuan, Yiyang : Chisolm, Nicole : Belton, Pamela : McKeithan, Lisa : Rajabuin, Serena	2018	Risk factors associated with multi-dimensional stigma among people living with HIV/AIDS who are homeless/unstably housed	AIDS Care
Davtyan, M. : Frederick, T. : Taylor, J. : Christensen, C. : Brown, B. J. : Nguyen, A. L.	2022	Determinants of COVID-19 vaccine acceptability among older adults living with HIV	Medicine
Davtyan, M. : Uraga, S. : Wilson, M. L. : Frederick, T.	2022	Internalized HIV-related stigma in women of color obtaining care at an HIV specialty center in Los Angeles County, California	AIDS Care - Psychological and Socio-Medical Aspects of AIDS/HIV
Dayapoglu, Nuray : Ayyildiz, Nese Iscan : Seker, Demet	2020	Fear of negative evaluation and the concealment of their disease by epilepsy patients	Epilepsy & behavior : E&B
Dayapoglu, Nuray : Yildiz, Esra : Tan, Mehtap : Genc, Fatma	2021	Perceived Stigma and Associated Factors among Patients with Epilepsy in Eastern Turkey: A Cross Sectional Study	International Journal of Caring Sciences
de Almeida Crispim, J. : da Silva, L. M. C. : Yamamura, M. : Popolin, M. P. : Ramos, A. C. V. : Arroyo, L. H. : Queiroz, A. A. R. : de Souza Belchior, A. : dos Santos, D. T. : Pieri, F. M. : Rodrigues, L. B. B. : Protti, S. T. : Pinto, I. C. : Palha, P. F. : Arcêncio, R. A.	2017	Validity and reliability of the tuberculosis-related stigma scale version for Brazilian Portuguese	BMC Infectious Diseases
de Filippis, R. : Menculini, G. : D'Angelo, M. : Carbone, E. A. : Tortorella, A. : De Fazio, P. : Steardo, L.	2022	Internalized-stigma and dissociative experiences in bipolar disorder	Frontiers in Psychiatry
de Jong, S. : van Donkersgoed, R. J. M. : Timmerman, M. E. : Aan Het Rot, M. : Wunderink, L. : Arends, J. : van Der Gaag, M. : Aleman, A. : Lysaker, P. H. : Pijnenborg, G. H. M.	2019	Metacognitive reflection and insight therapy (MERIT) for patients with schizophrenia	Psychological medicine
De La Cruz, Natalie : Davies, Susan : Stewart, Katharine	2011	Religion, Relationships and Reproduction: Correlates of Desire for a Child Among Mothers Living with HIV	AIDS & Behavior

de Oliveira Freitas, N. : Forero, C. G. : Caltran, M. P. : Alonso, J. : Spadoti Dantas, R. A. : Piccolo, M. S. : Farina, J. A. : Lawrence, J. W. : Rossi, L. A.	2018	Validation of the perceived stigmatization questionnaire for Brazilian adult burn patients	PLoS ONE
de Oliveira Freitas, N. : Pitta, N. C. : Dantas, R. A. S. : Farina, J. A. : Rossi, L. A.	2020	Comparison of the perceived stigmatization measures between the general population and burn survivors in Brazil	Burns
de Oliveira Freitas, Noélle : Paes Caltran, Marina : Spadoti Dantas, Rosana Aparecida : Aparecida Rossi, Lidia	2014	Translation and cultural adaptation of the Perceived Stigmatization Questionnaire for burn victims in Brazil	Revista da Escola de Enfermagem da USP
de Souza, E. A. P. : Borges, K. : Miyazaki, M. C. O. S. : Oliveira, K. S. : Nakano, T. C.	2016	Validity and reliability evidence of the questionnaire for illness representation, the impact of epilepsy, and stigma (QIRIS)	Arquivos de Neuro-Psiquiatria
de Zeeuw, J. : Douwstra, M. : Omansen, T. F. : Sopoh, G. E. : Johnson, C. : Phillips, R. O. : Alferink, M. : Saunderson, P. : Van der Werf, T. S. : Dijkstra, P. U. : Stienstra, Y.	2014	Psychometric Properties of the Participation Scale among Former Buruli Ulcer Patients in Ghana and Benin	PLoS Neglected Tropical Diseases
de Zeeuw, J. : Omansen, T. F. : Douwstra, M. : Barogui, Y. T. : Agossadou, C. : Sopoh, G. E. : Phillips, R. O. : Johnson, C. : Abass, K. M. : Saunderson, P. : Dijkstra, P. U. : van der Werf, T. S. : Stientstra, Y.	2014	Persisting Social Participation Restrictions among Former Buruli Ulcer Patients in Ghana and Benin	PLoS Neglected Tropical Diseases
Decean, L. : Badea, M. : Ilies, R. : Sasu, A. : Rus, V. : Mihai, A.	2022	Psoriasis-Related Stigma: Is There More to Uncover?	Journal of Interdisciplinary Medicine
Deering, K. N. : Logie, C. : Krüsi, A. : Ranville, F. : Braschel, M. : Duff, P. : Shannon, K.	2021	Prevalence and Correlates of HIV Stigma Among Women Living with HIV in Metro Vancouver, Canada	AIDS Behav.
Dehnavi, A. Z. : Daneshpazhooh, M. : Dehghani, A. : Balighi, K. : Azizpour, A. : Yaseri, M. : Ebrahimi, S. M. S. : Mahmoudi, H.	2022	Stigma Experience in Patients With Vitiligo: A Comprehensive Study in a Skin Hospital	Acta Med. Iran.
Deleo, F. : Quintas, R. : Pastori, C. : Pappalardo, I. : Didato, G. : Di Giacomo, R. : de Curtis, M. : Villani, F.	2020	Quality of life, psychiatric symptoms, and stigma perception in three groups of persons with epilepsy	Epilepsy and Behavior
DeMarco, R. F. : Chan, K.	2013	The Sistah Powah structured writing intervention: A feasibility study for aging, low-income, HIV-positive black women	Am. J. Health Promot.
Demirel, O. F. : Mayda, P. Y. : Yıldız, N. : Sağlam, H. : Koçak, B. T. : Habip, Z. : Kadak, M. T. : Balcıoğlu, İ : Kocazeybek, B.	2018	Self-stigma, depression, and anxiety levels of people living with HIV in Turkey	European Journal of Psychiatry
den Daas, C. : van den Berk, G. E. L. : Kleene, M. J. T. : de Munnik, E. S. : Lijmer, J. G. : Brinkman, K.	2019	Health-related quality of life among adult HIV positive patients: assessing comprehensive themes and interrelated associations	Quality of life research : an international journal of quality of life aspects of treatment, care and rehabilitation
Deng, C. : Lu, Q. : Yang, L. : Wu, R. : Liu, Y. : Li, L. : Cheng, S. : Wei, S. : Wang, Y. : Huang, Y. : Fu, L. : Yue, Z.	2019	Factors associated with stigma in community-dwelling stroke survivors in China: A cross-sectional study	Journal of the Neurological Sciences
Depla, M. F. I. : de Graaf, R. : van Weeghel, J. : Heeren, T. J.	2005	The role of stigma in the quality of life of older adults with severe mental illness	International Journal of Geriatric Psychiatry
Deres, A. T. : Bürkner, P. C. : Klauke, B. : Buhlmann, U.	2020	The role of stigma during the course of inpatient psychotherapeutic treatment in a German sample	Clinical psychology & psychotherapy
Deribew, A. : Tesfaye, M. : Hailmichael, Y. : Negussu, N. : Daba, S. : Wogi, A. : Belachew, T. : Apers, L. : Colebunders, R.	2009	Tuberculosis and HIV co-infection: Its impact on quality of life	Health and Quality of Life Outcomes
Deribew, A. : Tesfaye, M. : H.Michael, Y. : Apers, L. : Duchateau, L. : Colebunders, R.	2011	Common mental disorders in TB/HIV co-infected patients in Ethiopia	European Psychiatry
Derose, K. P. : Han, B. : Armenta, G. : Palar, K. : Then-Paulino, A. : Jimenez-Paulino, G. : Sheira, L. A. : Acevedo, R. : Lugo, C. : Veloz, I. : Donastorg, Y. : Wagner, G.	2022	Exploring antiretroviral therapy adherence, competing needs, and viral suppression among people living with HIV and food insecurity in the Dominican Republic	AIDS Care - Psychological and Socio-Medical Aspects of AIDS/HIV
Derost, P. P. : Ouchchane, L. : Morand, D. : Ulla, M. : Llorca, P. M. : Barget, M. : Debilly, B. : Lemaire, J. J. : Durif, F.	2007	Is DBS-STN appropriate to treat severe Parkinson disease in an elderly population?	Neurology
Desalegn, S. Y. : Asaye, M. M. : Temesgan, W. Z. : Badi, M. B.	2022	Antenatal depression and associated factors among HIV-positive pregnant women in South Gondar zone public health facilities, northwest Ethiopia, a cross-sectional study	Clinical Epidemiology and Global Health
Desta, F. : Tasew, A. : Tekalegn, Y. : Zenbaba, D. : Sahiledengle, B. : Assefa, T. : Negash, W. : Tahir, A. : Regasa, T. : Mamo, A. : Teferu, Z. : Solomon, D. : Gezahegn, H. : Bekele, K. : Regassa, Z. : Atlaw, D.	2022	Prevalence of depression and associated factors among people living with HIV/AIDS in public hospitals of Southeast Ethiopia	BMC Psychiatry
Devins, G. M. : Stam, H. J. : Koopmans, J. P.	1994	Psychosomal impact of laryngectomy mediated by perceived stigma and illness intrusiveness	CAN. J. PSYCHIATRY
Dewan, Mashael F. : Hassouneh, Dena : Song, MinKyoung : Lyons, Karen S.	2020	Development of the Breast Cancer Stigma Scale for Arab Patients	Asia-Pacific journal of oncology nursing
Dhungana, S. : Tulachan, P. : Chapagai, M. : Pant, S. B. : Lama, P. Y. : Upadhyaya, S.	2022	Internalized stigma in patients with schizophrenia: A hospital-based cross-sectional study from Nepal	PLoS ONE
Dickerson, F. B. : Sommerville, J. : Origoni, A. E. : Ringel, N. B. : Parente, F.	2002	Experiences of stigma among outpatients with schizophrenia	Schizophrenia Bulletin
DiIorio, C. : McCarty, F. : DePadilla, L. : Resnicow, K. : Holstad, M. M. : Yeager, K. : Sharma, S. M. : Morisky, D. E. : Lundberg, B.	2009	Adherence to antiretroviral medication regimens: A test of a psychosocial model	AIDS and Behavior
DiIorio, C. : Osborne Shafer, P. : Letz, R. : Henry, T. : Schomer, D. L. : Yeager, K.	2003	The association of stigma with self-management and perceptions of health care among adults with epilepsy	Epilepsy and Behavior
DiIorio, C. : Shafer, P. O. : Letz, R. : Henry, T. R. : Schomer, D. L. : Yeager, K. : Epstein, C. M. : Pennell, P. : Helmers, S. : Clemons, S. : Drislane, F. W. : Schachter, S. C. : Krishnamurthy, K. B. : Chang, B. : Sundstrom, D. : Geary, K. : Jordan, K.	2004	Project EASE: A study to test a psychosocial model of epilepsy medication management	Epilepsy and Behavior
Dikec, G. : Kutlu, Y.	2016	Effectiveness of Adherence Therapy for People With Schizophrenia in Turkey: A Controlled Study	Archives of psychiatric nursing
DiLorenzo, M. A. : Parcesepe, A. : Tymejczyk, O. : Hoffman, S. : Elul, B. : Weiser, S. D. : Remien, R. H. : Kulkarni, S. G. : Gadisa, T. : Melaku, Z. : Nash, D.	2019	Psychosocial Factors Associated with Food Insufficiency Among People Living with HIV/AIDS (PLWH) Initiating ART in Ethiopia	AIDS and behavior
Dimitropoulos, G. : Freeman, V. E. : Bellai, K. : Olmsted, M.	2013	Inpatients with severe anorexia nervosa and their siblings: Non-shared experiences and family functioning	European Eating Disorders Review
Dimitropoulos, G. : McCallum, L. : Colasanto, M. : Freeman, V. E. : Gadalla, T.	2016	The effects of stigma on recovery attitudes in people with anorexia nervosa in intensive treatment	J. Nerv. Ment. Dis.
Dimitrov, D. : Matusiak, Ł : Szepietowski, J. C.	2019	Stigmatization in Arabic psoriatic patients in the United Arab Emirates - A cross sectional study	Postepy Dermatologii i Alergologii
Dimitrov, Dimitre : Matusiak, Lukasz : Evers, Andrea : Jafferany, Mohammad : Szepietowski, Jacek	2019	Arabic language skin-related stigmatization instruments: Translation and validation process	Advances in clinical and experimental medicine : official organ Wroclaw Medical University
Ditchman, Nicole : Sheehan, Lindsay : Rafajko, Sean : Haak, Christopher : Kazukauskas, Kelly	2016	Predictors of social integration for individuals with brain injury: An application of the ICF model	Brain Injury
Do, N. T. : Phiri, K. : Bussmann, H. : Gaolathe, T. : Marlink, R. G. : Wester, C. W.	2010	Psychosocial factors affecting medication adherence among HIV-1 infected adults receiving combination antiretroviral therapy (cART) in Botswana	AIDS Research and Human Retroviruses
Doganavsargil-Baysal, O. : Cinemre, B. : Senol, Y. : Barcin, E. : Gokmen, Z.	2017	Epilepsy and stigmatization in Turkey	Epilepsy and Behavior
Doganavsargil-Baysal, O. : Senol, Y. : Coskun, H. S.	2019	Cancer stigma scale: Validity and reliability study of the Turkish version of the Cataldo Lung Cancer Stigma Scale for all cancer subtypes	Journal of Oncological Science
Dong, Ning : Chen, Wei-Ti : Lu, Hongzhou : Zhu, Zheng : Hu, Yan : Bao, Meijuan	2020	Unmet needs of symptom management and associated factors among the HIV-positive population in Shanghai, China: A cross-sectional study	Applied Nursing Research
Đorić, Stefan N.	2020	HIV-related stigma and subjective well-being: The mediating role of the Belief in a Just World	Journal of Health Psychology
Døssing, M. : Nilsson, K. K. : Svejstrup, S. R. : Sørensen, V. V. : Straarup, K. N. : Hansen, T. B.	2015	Low self-compassion in patients with bipolar disorder	Comprehensive Psychiatry
Dow, D. E. : Mmbaga, B. T. : Gallis, J. A. : Turner, E. L. : Gandhi, M. : Cunningham, C. K. : O'Donnell, K. E.	2020	A group-based mental health intervention for young people living with HIV in Tanzania: results of a pilot individually randomized group treatment trial	BMC public health
Dow, D. E. : Turner, E. L. : Shayo, A. M. : Mmbaga, B. : Cunningham, C. K. : O'Donnell, K.	2016	Evaluating mental health difficulties and associated outcomes among HIV-positive adolescents in Tanzania	AIDS Care - Psychological and Socio-Medical Aspects of AIDS/HIV
Dowshen, N. : Binns, H. J. : Garofalo, R.	2009	Experiences of HIV-related stigma among young men who have sex with men	AIDS Patient Care and STDs
Drapalski, A. L. : Aakre, J. : Brown, C. H. : Romero, E. : Lucksted, A.	2021	The Ending Self-Stigma for Posttraumatic Stress Disorder (ESS-P) Program: Results of a Pilot Randomized Trial	J. Trauma. Stress
Drapalski, Amy L. : Lucksted, Alicia : Perrin, Paul B. : Aakre, Jennifer M. : Brown, Clayton H. : DeForge, Bruce R. : Boyd, Jennifer E.	2013	A model of internalized stigma and its effects on people with mental illness	Psychiatric services (Washington, D.C.)
Drapalski, A. L. : Medoff, D. : Dixon, L. : Bellack, A.	2016	The reliability and validity of the Maryland Assessment of Recovery in Serious Mental Illness Scale	Psychiatry Res.
Drazic, Y. N. : Caltabiano, M. L.	2013	Chronic hepatitis B and C: Exploring perceived stigma, disease information, and health-related quality of life	Nursing & health sciences
Drenkard, C. : Theis, K. A. : Daugherty, T. T. : Helmick, C. G. : Dunlop-Thomas, C. : Bao, G. : Aspey, L. : Lewis, T. T. : Sam Lim, S.	2022	Depression, stigma and social isolation: the psychosocial trifecta of primary chronic cutaneous lupus erythematosus, a cross-sectional and path analysis	Lupus Science and Medicine
Drent, H. M. : van den Hoofdakker, B. : Buitelaar, J. K. : Hoekstra, P. J. : Dietrich, A.	2022	Factors Related to Perceived Stigma in Parents of Children and Adolescents in Outpatient Mental Healthcare	International Journal of Environmental Research and Public Health
Drewes, Jochen : Langer, Phil C. : Ebert, Jennifer : Kleiber, Dieter : Gusy, Burkhard	2021	Associations Between Experienced and Internalized HIV Stigma, Adversarial Growth, and Health Outcomes in a Nationwide Sample of People Aging with HIV in Germany	AIDS & Behavior
Du, L. : Chen, X. : Zhu, X. : Zhang, Y. : Wu, R. : Xu, J. : Ji, H. : Zhou, L. : Lu, X.	2020	Determinants of medication adherence for pulmonary tuberculosis patients during continuation phase in Dalian, Northeast China	Patient Preference and Adherence
Du, Mengran : Zhao, Jian : Zhang, Jianxin : Lau, Joseph T. F : Mo, Phoenix K. H : Li, Jinghua	2018	Depression and social support mediate the effect of HIV self-stigma on condom use intentions among Chinese HIV-infected men who have sex with men	AIDS Care

Duan, W. : Wang, Z.	2019	Dispositional mindfulness promotes public health of the obesity population by reducing perceived discrimination and weight stigma concerns	Journal of Public Health (Germany)
Dubreucq, J. : Plasse, J. : Gabayet, F. : Faraldo, M. : Blanc, O. : Chereau, I. : Cervello, S. : Couhet, G. : Demily, C. : Guillard-Bouhet, N. : Gouache, B. : Jaafari, N. : Legrand, G. : Legros-Lafarge, E. : Pommier, R. : Quilès, C. : Straub, D. : Verdoux, H. : Vignaga, F. : Massoubre, C. : Franck, N.	2020	Self-stigma in serious mental illness and autism spectrum disorder: Results from the REHABase national psychiatric rehabilitation cohort	European psychiatry : the journal of the Association of European Psychiatrists
Dubreucq, J. : Plasse, J. : Gabayet, F. : Faraldo, M. : Blanc, O. : Chereau, I. : Cervello, S. : Couhet, G. : Demily, C. : Guillard-Bouhet, N. : Gouache, B. : Jaafari, N. : Legrand, G. : Legros-Lafarge, E. : Pommier, R. : Quilès, C. : Straub, D. : Verdoux, H. : Vignaga, F. : Massoubre, C. : Franck, N.	2022	Stigma resistance is associated with advanced stages of personal recovery in serious mental illness patients enrolled in psychiatric rehabilitation	Psychological medicine
Dubreucq, M. : Plasse, J. : Gabayet, F. : Blan, O. : Chereau, I. : Cervello, S. : Couhet, G. : Demily, C. : Guillard-Bouhet, N. : Gouache, B. : Jaafari, N. : Legrand, G. : Legros-Lafarge, E. : Mora, G. : Pommier, R. : Quilès, C. : Verdoux, H. : Vignaga, F. : Massoubre, C. : Franck, N. : Dubreucq, J.	2021	Sex Differences in Recovery-Related Outcomes and Needs for Psychiatric Rehabilitation in People With Schizophrenia Spectrum Disorder	Journal of Clinical Psychiatry
Duko, Bereket : Bedaso, Asres : Ayano, Getinet : Yohannis, Zegeye	2019	Perceived Stigma and Associated Factors among Patient with Tuberculosis, Wolaita Sodo, Ethiopia: Cross-Sectional Study	Tuberculosis research and treatment
Duko, B. : Gebeyehu, A. : Ayano, G.	2015	Prevalence and correlates of depression and anxiety among patients with tuberculosis at WolaitaSodo University Hospital and Sodo Health Center, WolaitaSodo, South Ethiopia, Cross sectional study	BMC Psychiatry
Duko, B. : Geja, E. : Zewude, M. : Mekonen, S.	2018	Prevalence and associated factors of depression among patients with HIV/AIDS in Hawassa, Ethiopia, cross-sectional study	Annals of General Psychiatry
Duko, B. : Toma, A. : Abraham, Y.	2019	Prevalence and correlates of common mental disorder among HIV patients attending antiretroviral therapy clinics in Hawassa City, Ethiopia	Annals of General Psychiatry
Duko, B. : Toma, A. : Asnake, S. : Abraham, Y.	2019	Depression, anxiety and their correlates among patients with HIV in South Ethiopia: An institution-based cross-sectional study	Frontiers in Psychiatry
Duran, S. : Öz, Y. C.	2022	The Impact of Family Environment on Self-Stigmatization and Social Functionality Among Schizophrenic Patients	Ethiop. J. Health Dev.
Dussault, Josée M. : Zimba, Chifundo : Malava, Jullita : Akello, Harriet : Stockton, Melissa A. : Udedi, Michael : Gaynes, Bradley N. : Hosseinipour, Mina C. : Pence, Brian W. : Masiye, Jones	2021	'thandi should feel embarrassed': Describing the validity and reliability of a tool to measure depression-related stigma among patients with depressive symptoms in malawi	Social Psychiatry and Psychiatric Epidemiology: The International Journal for Research in Social and Genetic Epidemiology and Mental Health Services
Earnshaw, V. A. : Bogart, L. M. : Laurenceau, J. P. : Chan, B. T. : Maughan-Brown, B. G. : Dietrich, J. J. : Courtney, I. : Tshabalala, G. : Orrell, C. : Gray, G. E. : Bangsberg, D. R. : Katz, I. T.	2018	Internalized HIV stigma, ART initiation and HIV-1 RNA suppression in South Africa: exploring avoidant coping as a longitudinal mediator	Journal of the International AIDS Society
Earnshaw, Valerie A. : Kidman, Rachel C. : Violari, Avy	2018	Stigma, Depression, and Substance Use Problems Among Perinatally HIV-Infected Youth in South Africa	AIDS & Behavior
Earnshaw, Valerie A. : Rosenthal, Lisa : Lang, Shawn M.	2016	Stigma, activism, and well-being among people living with HIV	AIDS Care
Eaton, L. A. : Allen, A. : Maksut, J. L. : Earnshaw, V. : Watson, R. J. : Kalichman, S. C.	2020	HIV microaggressions: a novel measure of stigma-related experiences among people living with HIV	Journal of behavioral medicine
Ebrahim, O. S. : Al-Attar, G. S. T. : Gabra, R. H. : Osman, D. M. M.	2020	Stigma and burden of mental illness and their correlates among family caregivers of mentally ill patients	J. Egypt. Public Health Assoc.
Echenique, M. : Illa, L. : Saint-Jean, G. : Avellaneda, V. B. : Sanchez-Martinez, M. : Eisdorfer, C.	2013	Impact of a secondary prevention intervention among HIV-positive older women	AIDS Care - Psychological and Socio-Medical Aspects of AIDS/HIV
Edelman, E. : Lunze, Karsten : Cheng, Debbie : Lioznov, Dmitry : Quinn, Emily : Gnatienko, Natalia : Bridden, Carly : Chaisson, Christine : Walley, Alexander : Krupitsky, Evgeny : Raj, Anita : Samet, Jeffrey	2017	HIV Stigma and Substance Use Among HIV-Positive Russians with Risky Drinking	AIDS & Behavior
Ediati, A. : Zulfa Juniarto, A. : Birnie, E. : Okkerse, J. : Wisniewski, A. : Drop, S. : Faradz, S. M. H. : Dessens, A.	2017	Social stigmatisation in late identified patients with disorders of sex development in Indonesia	BMJ Paediatr. Open
Edmonds, A. : Haley, D. F. : Tong, W. : Kempf, M. C. : Rahangdale, L. : Adimora, A. A. : Anastos, K. : Cohen, M. H. : Fischl, M. : Wilson, T. E. : Wingood, G. : Konkle-Parker, D.	2021	Associations between population density and clinical and sociodemographic factors in women living with HIV in the Southern United States	AIDS Care - Psychological and Socio-Medical Aspects of AIDS/HIV
Egbe, T. O. : Nge, C. A. : Ngouekam, H. : Asonganyi, E. : Nsagha, D. S.	2020	Stigmatization among People Living with HIV/AIDS at the Kumba Health District, Cameroon	J. Int. Assoc. Providers AIDS Care
Eguzo, Kelechi : Oluoha, Chukwuemeka : Onwueyi, Nancy : Okon, Kingsley : Laskar, Farhana Yasmin : Egharevba, Peace : Mbogu, Florence : King, Udemeh : Inyang, Nse : Nnah, Kingsley : Ekanem, Uwemedimbuk	2022	Psychosocial Outcomes and Stigma Among Cancer Patients Undergoing Navigation in Southern Nigeria	Journal of Oncology Navigation & Survivorship
Eichler, Martin : Hechtner, Marlene : Wehler, Beatrice : Buhl, Roland : Stratmann, Jan : Sebastian, Martin : Schmidberger, Heinz : Peuser, Jessica : Kortsik, Cornelius : Nestle, Ursula : Wiesemann, Sebastian : Wirtz, Hubert : Wehler, Thomas : Blettner, Maria : Singer, Susanne	2018	Psychological distress in lung cancer survivors at least 1 year after diagnosis-Results of a German multicenter cross-sectional study	Psycho-Oncology
Eidenmueller, K. : Grimm, F. : Hermann, D. : Frischknecht, U. : Kiefer, F. : Dziobek, I. : Bekier, N. K.	2022	Exploring impaired insight in opioid addiction: The role of self-stigma	Heroin Addiction and Related Clinical Problems
Eisapareh, K. : Nazari, M. : Kaveh, M. H. : Ghahremani, L. : Parkestani, K. N. : Ur Rehman, A.	2022	Relationship between social support and social stigma among AIDS patients in Shiraz	HIV and AIDS Review
Ekstrand, M. L. : Heylen, E. : Mazur, A. : Steward, W. T. : Carpenter, C. : Yadav, K. : Sinha, S. : Nyamathi, A.	2018	The Role of HIV Stigma in ART Adherence and Quality of Life Among Rural Women Living with HIV in India	AIDS Behav.
El-Tantawy, A. M. : Raya, Y. M. : Al-Yahya, A. : Mohamed, S. A. : Youssef, U. M.	2014	Stigma and expressed emotions: A study of people with severe psychiatric illnesses and their family members	Middle East Curr. Psychiatry
Elafros, M. A. : Bowles, R. P. : Atadzhanov, M. : Mbewe, E. : Haworth, A. : Chomba, E. : Birbeck, G. L.	2015	Reexamining epilepsy-associated stigma: validation of the Stigma Scale of Epilepsy in Zambia	Qual. Life Res.
Elafros, M. A. : Gardiner, J. C. : Sikazwe, I. : Okulicz, J. F. : Paneth, N. : Chomba, E. : Birbeck, G. L.	2018	Evaluating layered stigma from comorbid HIV and epilepsy among Zambian adults	eNeurologicalSci
Eldridge-Smith, Elizabeth Devon : Loew, Megan : Stepleman, Lara M.	2021	The adaptation and validation of a stigma measure for individuals with multiple sclerosis	Disability and rehabilitation
Elgohari, H. M. : Bassiony, M. M. : Sehlo, M. G. : Youssef, U. M. : Ali, H. M. : Shahin, I. : Elrafey, D. S. : Mahdy, R. S.	2021	COVID-19 Infection Stigma Scale: psychometric properties	Egyptian Journal of Neurology, Psychiatry and Neurosurgery
Elizondo, J. E. : Treviño, A. C. : Violant, D.	2015	Dentistry and HIV/AIDS related stigma	Revista de saude publica
Elkington, K. : McKinnon, K. : Mann, C. : Collins, P. : Leu, C. : Wainberg, M.	2010	Perceived mental illness stigma and HIV risk behaviors among adult psychiatric outpatients in Rio de Janeiro, Brazil	Community Mental Health Journal
Elle Saine, M. : Moore, T. M. : Szymczak, J. E. : Bamford, L. P. : Barg, F. K. : Mitra, N. : Schnittker, J. : Holmes, J. H. : Lo Re, V.	2020	Validation of a modified Berger HIV stigma scale for use among patients with hepatitis C virus (HCV) infection	PLoS ONE
Elsayed, H. : O'Connor, C. : Leyritana, K. : Salvana, E. : Cox, S. E.	2021	Depression, Nutrition, and Adherence to Antiretroviral Therapy in Men Who Have Sex With Men in Manila, Philippines	Frontiers in public health
Elson, J. L. : Cadogan, M. : Apabhai, S. : Whittaker, R. G. : Phillips, A. : Trennell, M. I. : Horvath, R. : Taylor, R. W. : McFarland, R. : McColl, E. : Turnbull, D. M. : Gorman, G. S.	2013	Initial development and validation of a mitochondrial disease quality of life scale	Neuromuscular Disorders
Emlet, C. A.	2005	Measuring stigma in older and younger adults with HIV/AIDS: an analysis of an HIV Stigma Scale and initial exploration of subscales	Research on Social Work Practice
Emlet, C. A.	2006	A comparison of HIV stigma and disclosure patterns between older and younger adults living with HIV/AIDS	AIDS Patient Care & STDs
Emlet, C. A.	2007	Experiences of stigma in older adults living with HIV/AIDS: a mixed-methods analysis	AIDS Patient Care & STDs
Emlet, Charles A. : Brennan, David J. : Brennenstuhl, Sarah : Rueda, Sergio : Hart, Trevor A. : Rourke, Sean B.	2013	Protective and risk factors associated with stigma in a population of older adults living with HIV in Ontario, Canada	AIDS Care
Endeshaw, M. : Walson, J. : Rawlins, S. : Dessie, A. : Alemu, S. : Andrews, N. : Rao, D.	2014	Stigma in Ethiopia: Association with depressive symptoms in people with HIV	AIDS Care - Psychological and Socio-Medical Aspects of AIDS/HIV
Eneanya, Obiora A. : Garske, Tini : Donnelly, Christl A.	2019	The social, physical and economic impact of lymphedema and hydrocele: a matched cross-sectional study in rural Nigeria	BMC Infectious Diseases
Erdoğan, E. : Demir, S.	2022	The Effect of Solution Focused Group Psychoeducation Applied to Schizophrenia Patients on Self-Esteem, Perception of Subjective Recovery and Internalized Stigmatization	Issues in mental health nursing
Erdogan, Z. : Kurcer, M. A. : Kurtuncu, M. : Catalcam, S.	2018	Validity and reliability of the Turkish version of the weight self-stigma questionnaire	Journal of the Pakistan Medical Association
Ernst, J. : Mehnert, A. : Dietz, A. : Hornemann, B. : Esser, P.	2017	Perceived stigmatization and its impact on quality of life - results from a large register-based study including breast, colon, prostate and lung cancer patients	BMC Cancer
Ersoy, M. A. : Varan, A.	2007	Reliability and validity of the Turkish version of the internalized stigma of mental illness scale	Türk psikiyatri dergisi = Turkish journal of psychiatry
Ertugrul, A. : Uluğ, B.	2004	Perception of stigma among patients with schizophrenia	Social Psychiatry and Psychiatric Epidemiology
Esan, O. : Esan, A.	2018	Sexual Dysfunction Among Patients With Schizophrenia in Southwest Nigeria	Journal of sex & marital therapy
Espinosa, R. : Valiente, C. : Rigabert, A. : Song, H.	2016	Recovery style and stigma in psychosis: The healing power of integrating	Cogn. Neuropsych.
Esser, P. : Mehnert, A. : Johansen, C. : Hornemann, B. : Dietz, A. : Ernst, J.	2018	Body image mediates the effect of cancer-related stigmatization on depression: A new target for intervention	Psycho-Oncology
Etesam, Farnaz : Assarian, Fatemeh : Hosseini, Hamed : Ghoreishi, Fatemeh Sadat	2014	Stigma and its Determinants among Male Drug Dependents Receiving Methadone Maintenance Treatment	Archives of Iranian Medicine (AIM)
Etowa, J. : Hannan, J. : Babatunde, S. : Etowa, E. B. : Mkandawire, P. : Phillips, J. C.	2020	HIV-Related Stigma Among Black Mothers in Two North American and One African Cities	J Racial Ethn Health Disparities

Evangeli, M. : Newell, M. L. : Richter, L. : McGrath, N.	2014	The association between self-reported stigma and loss-to-follow up in treatment eligible HIV positive adults in rural Kwazulu-Natal, South Africa	PLoS ONE
Evers, A. W. M. : Duller, P. : Van De Kerkhof, P. C. M. : Van Der Valk, P. G. M. : De Jong, E. M. G. J. : Gerritsen, M. J. P. : Otero, E. : Verhoeven, E. W. M. : Verhaak, C. M. : Kraaimaat, F. W.	2008	The Impact of Chronic Skin Disease on Daily Life (ISDL): A generic and dermatology-specific health instrument	Br. J. Dermatol.
Ezeamama, A. E. : Guwatudde, D. : Wang, M. : Bagenda, D. : Brown, K. : Kyeyune, R. : Smith, Emily : Wamani, H. : Manabe, Y. C. : Fawzi, W. W.	2016	High perceived social standing is associated with better health in HIV-infected Ugandan adults on highly active antiretroviral therapy	Journal of behavioral medicine
Ezeamama, Amara E. : Woolfork, Makhabele N. : Guwatudde, David : Bagenda, Danstan : Manabe, Yukari C. : Fawzi, Wafaie W. : Smith Fawzi, Mary C.	2016	Depressive and Anxiety Symptoms Predict Sustained Quality of Life Deficits in HIV-Positive Ugandan Adults Despite Antiretroviral Therapy: A Prospective Cohort Study	Medicine
Ezzedine, K. : Shourick, J. : Bergqvist, C. : Misery, L. : Chuberre, B. : Kerob, D. : Halioua, B. : Le Fur, G. : Paul, C. : Richard, M. A. : Taieb, C.	2022	Patient Unique Stigmatization Holistic tool in dermatology (PUSH-D): Development and validation of a dermatology-specific stigmatization assessment tool	Journal of the European Academy of Dermatology and Venereology
Fabian, K. E. : Huh, D. : Kemp, C. G. : Nevin, P. E. : Simoni, J. M. : Andrasik, M. : Turan, J. M. : Cohn, S. E. : Mugavero, M. J. : Rao, D.	2019	Moderating Factors in an Anti-stigma Intervention for African American Women with HIV in the United States: A Secondary Analysis of the UNITY Trial	AIDS Behav.
Facente, S. N. : Lam-Hine, T. : Bhatta, D. N. : Hecht, J.	2022	Impact of Racial Categorization on Effect Estimates: An HIV Stigma Analysis	American Journal of Epidemiology
Fadipe, B. : Adebowale, T. O. : Ogunwale, A. : Fadipe, Y. O. : Ojeyinka, A. H. A. : Olagunju, A. T.	2018	Internalized stigma in schizophrenia: a cross-sectional study of prevalence and predictors	Int. J. Cult. Ment. Health
Fadzil, N. A. : Othman, Z. : Mustafa, M.	2016	Stigma in Malay patients with HIV/AIDS in Malaysia	International Medical Journal
Fahoum, K. : Al-Krenawi, A.	2021	Perceptions of stigma toward mental illness in Arab society in Israel	Int. Soc. Work.
Fan, C. W. : Chang, K. C. : Lee, K. Y. : Yang, W. C. : Pakpour, A. H. : Potenza, M. N. : Lin, C. Y.	2022	Rasch Modeling and Differential Item Functioning of the Self-Stigma Scale-Short Version among People with Three Different Psychiatric Disorders	International Journal of Environmental Research and Public Health
Fang-fang, Xu : Wei-hua, Yu : Mei, Yu : Sheng-qin, Wang : Gui-hua, Zhou	2019	The correlation between stigma and adjustment in patients with a permanent colostomy in the Midlands of China	World Council of Enterostomal Therapists Journal
Farabee, D. : Hall, E. : Zaheer, A. : Joshi, V.	2019	The impact of perceived stigma on psychiatric care and outcomes for correctional mental health patients	Psychiatry Research
Farah, Z. : Sahar, E. : Rim, J. : Dhouha, B. : Jihen, A. : Rym, G.	2022	Perceived Stigma and Burden in Tunisian Natural Caregivers of Patients with Schizophrenia	Clinical Schizophrenia and Related Psychoses
Farber, E. W. : Shahane, A. A. : Brown, J. L. : Campos, P. E.	2014	Perceived stigma reductions following participation in mental health services integrated within community-based HIV primary care	AIDS Care Psychol. Socio-Med. Asp. AIDS HIV
Farzand, Maryam : Baysen, Engin	2018	Group differences on affiliate stigma experienced by family caregivers of psychiatric patients	Quality & Quantity: International Journal of Methodology
Fathy, H. : El Ray, L. : Madbouly, N. : Shawky-Kamal, Y. : El-Nawawy, Y.	2017	Stigma, self-esteem, and depression in adolescent patients with epilepsy	Middle East Curr. Psychiatry
Fawale, M. B. : Owolabi, M. O. : Ogunniyi, A.	2014	Effects of seizure severity and seizure freedom on the health-related quality of life of an African population of people with epilepsy	Epilepsy Behav.
Fawzi, Mounir H. : Said, Nagwa S. : Fawzi, Maggie M. : Kira, Ibrahim A. : Fawzi, Mohab M. : Abdel-Moety, Hanaa	2016	Psychiatric referral and glycemic control of Egyptian type 2 diabetes mellitus patients with depression	General hospital psychiatry
Fekete, E. M. : Williams, S. L. : Skinta, M. D. : Bogusch, L. M.	2016	Gender differences in disclosure concerns and HIV-related quality of life	AIDS Care - Psychological and Socio-Medical Aspects of AIDS/HIV
Fekete, Erin M. : Williams, Stacey L. : Skinta, Matthew D.	2018	Internalised HIV-stigma, loneliness, depressive symptoms and sleep quality in people living with HIV	Psychology & Health
Feldhaus, T. : Falke, S. : von Gruchalla, L. : Maisch, B. : Uhlmann, C. : Bock, E. : Lencer, R.	2018	The impact of self-stigmatization on medication attitude in schizophrenia patients	Psychiatry Res.
Feng, L. S. : Li, X. Y. : Wang, H. R. : Zhan, J. J. : Chen, D. : Wang, Y. F.	2018	Development and validation of the cancer self-perceived discrimination scale for Chinese cancer patients	Health and Quality of Life Outcomes
Fereshtehnejad, S. M. : Ghazi, L. : Shafieesabet, M. : Shahidi, G. A. : Delbari, A. : Lökk, J.	2014	Motor, psychiatric and fatigue features associated with nutritional status and its effects on quality of life in Parkinson's disease patients	PLoS ONE
Fernandes, P. T. : Salgado, P. C. B. : Noronha, A. L. A. : Sander, J. W. : Li, L. M.	2007	Stigma scale of epilepsy: Validation process	Arquivos de Neuro-Psiquiatria
Fernando, S. M. : Deane, F. P. : McLeod, H. J. : Davis, E. L.	2017	A carer burden and stigma in schizophrenia and affective disorders: Experiences from Sri Lanka	Asian Journal of Psychiatry
Fernando, S. M. : Deane, F. P. : McLeod, H. J.	2017	The delaying effect of stigma on mental health help-seeking in Sri Lanka	Asia-Pacific Psychiatry
Ferrante, J. M. : Seaman, K. : Bator, A. : Ohman-Strickland, P. : Gundersen, D. : Clemow, L. : Puhl, R.	2016	Impact of perceived weight stigma among underserved women on doctor-patient relationships	Obesity Science and Practice
Filiatreau, L. M. : Wright, M. : Kimaru, L. : Gómez-Olivé, F. X. : Selin, A. : Twine, R. : Kahn, K. : Pettifor, A.	2020	Correlates of ART Use Among Newly Diagnosed HIV Positive Adolescent Girls and Young Women Enrolled in HPTN 068	AIDS Behav.
Fitri, Saima : Tahlil, Teuku : Susanti, Suryane Sulistiana	2022	Stigma in Tuberculosis Sufferers: A Study in Simeulue Regency, Aceh Indonesia	International Journal of Nursing Education
Fitts, W. : Rahamatou, N. T. : Abass, C. F. : Vogel, A. C. : Ghislain, A. H. : Sakadi, F. : Hongxiang, Q. : Conde, M. L. : Baldé, A. T. : Hamani, A. B. D. : Bah, A. K. : Anand, P. : Patenaude, B. : Mateen, F. J.	2019	School status and its associations among children with epilepsy in the Republic of Guinea	Epilepsy and Behavior
Fletcher, A. : Nye, H. : Mayeku, J. : Boling, W.	2015	Evaluation of stigmatization and quality of life at long term follow-up in patients with surgery for epilepsy in Uganda	Epilepsy Currents
Flickinger, Tabor E. : Grabowski, Marika : Waldman, Ava Lena : Dillingham, Rebecca : DeBolt, Claire : Kosmacki, Alison : Xie, Alice : Reynolds, George : Conaway, Mark : Cohn, Wendy F. : Ingersoll, Karen	2018	Addressing Stigma Through a Virtual Community for People Living with HIV: A Mixed Methods Study of the PositiveLinks Mobile Health Intervention	AIDS & Behavior
Fongkaew, W. : Viseskul, N. : Suksatit, B. : Settheekul, S. : Chontawan, R. : Grimes, R. M. : Grimes, D. E.	2014	Verifying quantitative stigma and medication adherence scales using qualitative methods among thai youth living with HIV/AIDS	J. Int. Assoc. Providers AIDS Care
Forsgren, L. : Ghanean, H. : Jacobsson, L. : Richter, J.	2013	On the experience of stigma by persons with epilepsy in Sweden and Iran - A comparative study	Seizure
Foster, P. P. : Gaskins, S. W.	2009	Older African Americans' management of HIV/AIDS stigma	AIDS Care Psychol. Socio-Med. Asp. AIDS HIV
France, N. F. : Macdonald, S. H. F. : Conroy, R. R. : Chiroro, P. : Cheallaigh, D. N. : Nyamucheta, M. : Mapanda, B. : Shumba, G. : Mudede, D. : Byrne, E.	2019	'We are the change' - An innovative community-based response to address self-stigma: A pilot study focusing on people living with HIV in Zimbabwe	PLoS ONE
Franklin, Hannah : Tora, Abebayehu : Deribe, Kebede : Reda, Ayalu A. : Davey, Gail	2013	Development of a scale to measure stigma related to podoconiosis in Southern Ethiopia	BMC public health
Franklin, S. : Mouliom, A. : Sinkala, E. : Kanunga, A. : Helova, A. : Dionne-Odom, J. : Turan, J. M. : Vinikoor, M.	2018	Hepatitis B virus contact disclosure and testing in Lusaka, Zambia: A mixed-methods study	BMJ Open
Fredericksen, R. J. : Gibbons, L. E. : Fitzsimmons, E. : Nance, R. M. : Schafer, K. R. : Batey, D. S. : Loo, S. : Dougherty, S. : Mathews, W. C. : Christopoulos, K. : Mayer, K. H. : Mugavero, M. J. : Kitahata, M. M. : Crane, P. K. : Crane, H. M.	2021	Impact and correlates of sub-optimal social support among patients in HIV care	AIDS Care - Psychological and Socio-Medical Aspects of AIDS/HIV
Freire de Aguiar, Maria Isis : Pacífico Alves, Naiana : Batista Braga, Violante Augusta : Alves Souza, Ângela Maria : Marques Araújo, Michell Ângelo : de Almeida, Paulo César	2018	ASPECTOS PSICOSSOCIAIS DA QUALIDADE DE VIDA DE RECEPTORES DE TRANSPLANTE HEPÁTICO	Texto & Contexto Enfermagem
Fresán, A. : Robles-García, R. : Madrigal, E. : Tovilla-Zarate, C. A. : Martínez-López, N. : Arango de Montis, I.	2018	Demographic and clinical features related to perceived discrimination in schizophrenia	Psychiatry Research
Fröhlich, E. : Sassenrath, C. : Nadji-Ohl, M. : Unteroberdörster, M. : Rückriegel, S. : von der Brelie, C. : Roder, C. : Forster, M. T. : Schommer, S. : Löhr, M. : Pala, A. : Goebel, S. : Mielke, D. : Gerlach, R. : Renovanz, M. : Wirtz, C. R. : Onken, J. : Czabanka, M. : Tatagiba, M. S. : Rohde, V. : Ernestus, R. I. : Vajkoczy, P. : Gansland, O. : Coburger, J.	2022	Resilience in Lower Grade Glioma Patients	Cancers
Fu, B. : Qin, N. : Cheng, L. : Tang, G. : Cao, Y. : Yan, C. : Huang, X. : Yan, P. : Zhu, S. : Lei, J.	2015	Development and validation of an Infertility Stigma Scale for Chinese women	Journal of Psychosomatic Research
Fu, S. N. : Chin, W. Y. : Wong, C. K. H. : Yeung, V. T. F. : Yiu, M. P. : Tsui, H. Y. : Chan, K. H.	2013	Development and validation of the Chinese attitudes to starting insulin questionnaire (Ch-ASIQ) for primary care patients with type 2 diabetes	PLoS ONE
Fu, Sau Nga : Wong, Carlos King Ho : Chin, Weng Yee : Luk, Wan	2015	Association of more negative attitude towards commencing insulin with lower glycosylated hemoglobin (HbA1c) level: a survey on insulin-naive type 2 diabetes mellitus Chinese patients	Journal of diabetes and metabolic disorders
Fuge, Terefe Gone : Tsourtos, George : Miller, Emma R.	2022	Factors affecting optimal adherence to antiretroviral therapy and viral suppression amongst HIV-infected prisoners in South Ethiopia: a comparative cross-sectional study	AIDS Research & Therapy
Fujisawa, D. : Umezawa, S. : Fujimori, M. : Miyashita, M.	2021	Prevalence and associated factors of perceived cancer-related stigma in Japanese cancer survivors	Japanese Journal of Clinical Oncology
Funderburk, J. A. : McCormick, B. P. : Austin, J. K.	2007	Does attitude toward epilepsy mediate the relationship between perceived stigma and mental health outcomes in children with epilepsy?	Epilepsy Behav.
Fung, K. M. T. : Tsang, H. W. H. : Corrigan, P. W. : Lam, C. S. : Cheng, W. M.	2007	Measuring self-stigma of mental illness in China and its implications for recovery	Int. J. Soc. Psychiatry
Fung, K. M. T. : Tsang, H. W. H. : Chan, F.	2010	Self-stigma, stages of change and psychosocial treatment adherence among chinese people with schizophrenia: A path analysis	Soc. Psychiatry Psychiatr. Epidemiol.
Fung, V. S. C. : Herawati, L. : Wan, Y. : Boyle, R. : Hughes, A. : Lueck, C. : Silburn, P. : Snow, B. : Stell, R. : Temlett, J.	2009	Quality of life in early Parkinson's disease treated with levodopa/carbidopa/entacapone	Movement Disorders
Fuster-Ruizdeapodaca, M. J. : Laguía, A. : Safreed-Harmon, K. : Lazarus, J. V. : Cenoz, S. : Del Amo, J.	2019	Assessing quality of life in people with HIV in Spain: Psychometric testing of the Spanish version of WHOQOL-HIV-BREF	Health and Quality of Life Outcomes

Fuster-RuizdeApodaca, Maria Jose : Safreed-Harmon, Kelly : Pastor de la Cal, Marta : Laguia, Ana : Naniche, Denise : Lazarus, Jeffrey V.	2021	Development of a Clinic Screening Tool to Identify Burdensome Health-Related Issues Affecting People Living With HIV in Spain	Frontiers in psychology
Gabbidon, Jheanell : Brohan, Elaine : Clement, Sarah : Henderson, R. Claire : Thornicroft, Graham : Miriad Study Group	2013	The development and validation of the Questionnaire on Anticipated Discrimination (QUAD)	BMC psychiatry
Gabriel, A. : Violato, C.	2010	The development and psychometric assessment of an instrument to measure attitudes towards depression and its treatments in patients suffering from non-psychotic depression	Journal of Affective Disorders
Gabriel, D. : Ventura, M. : Samões, R. : Freitas, J. : Lopes, J. : Ramalheira, J. : Martins da Silva, A. : Chaves, J.	2020	Social impairment and stigma in genetic generalized epilepsies	Epilepsy and Behavior
Gaebel, W.	2016	Promoting stigma coping and empowerment: Results from the multi-center clinical trial STEM	European Psychiatry
Galderisi, S. : Rossi, A. : Rocca, P. : Bertolino, A. : Mucci, A. : Bucci, P. : Rucci, P. : Gibertoni, D. : Aguglia, E. : Amore, M. : Blasi, G. : Comparelli, A. : Di Giannantonio, M. : Goracci, A. : Marchesi, C. : Monteleone, P. : Montemagni, C. : Pinna, F. : Roncone, R. : Siracusano, A. : Stratta, P. : Torti, M. C. : Vita, A. : Zeppegno, P. : Chieffi, M. : Maj, M. : Piegari, G. : Merlotti, E. : Rocco, M. : Campana, T. : Plescia, G. : Montefusco, V. : Porcelli, S. : Barlati, S. : Carpiniello, B. : Signorelli, M. S. : Acciavatti, T. : Bellomo, A. : Respino, M. : Di Emidio, G. : Giusti, L. : Oldani, L. : Tomasetti, C. : Tenconi, E. : Ossola, P. : Dell'Osso, L. : Squarcione, C. : Brugnoli, R. : Niolu, C. : Bartoli, L. : Bolognesi, S. : Frieri, T.	2016	Pathways to functional outcome in subjects with schizophrenia living in the community and their unaffected first-degree relatives	Schizophrenia Research
Galderisi, S. : Rucci, P. : Kirkpatrick, B. : Mucci, A. : Gibertoni, D. : Rocca, P. : Rossi, A. : Bertolino, A. : Strauss, G. P. : Aguglia, E. : Bellomo, A. : Murri, M. B. : Bucci, P. : Carpiniello, B. : Comparelli, A. : Cuomo, A. : De Berardis, D. : Dell'Osso, L. : Di Fabio, F. : Gelao, B. : Marchesi, C. : Monteleone, P. : Montemagni, C. : Orsenigo, G. : Pacitti, F. : Roncone, R. : Santonastaso, P. : Siracusano, A. : Vignapiano, A. : Vita, A. : Zeppegno, P. : Maj, M.	2018	Interplay among psychopathologic variables, personal resources, context-related factors, and real-life functioning in individuals with schizophrenia a network analysis	JAMA Psychiatry
Gallo, Veronica Del Valle : Burrone, Maria Soledad : Fernandez, Alicia Ruth : Boyd, Jennifer E. : Abeldano, Roberto Ariel	2017	Psychometric properties of the Internalized Stigma of Mental Illness scale adapted for people who use psychoactive substances	Revista de la Facultad de Ciencias Medicas (Cordoba, Argentina)
Galvan, F. H. : Davis, E. M. : Banks, D. : Bing, E. G.	2008	HIV stigma and social support among African Americans	AIDS Patient Care & STDs
Gamarel, Kristi E. : Kuo, Caroline C. : Boyes, Mark E. : Cluver, Lucie D.	2017	The dyadic effects of HIV stigma on the mental health of children and their parents in South Africa	Journal of HIV/AIDS & Social Services
Garapati, P. : Pal, B. : Siddiqui, N. A. : Bimal, S. : Das, P. : Murti, K. : Pandey, K.	2018	Knowledge, stigma, health seeking behaviour and its determinants among patients with post kalaazar dermal leishmaniasis, Bihar, India	PLoS ONE
Garfin, D. R. : Shin, S. S. : Ekstrand, M. L. : Yadav, K. : Carpenter, C. L. : Sinha, S. : Nyamathi, A. M.	2019	Depression, social support, and stigma as predictors of quality of life over time: results from an Asha-based HIV/AIDS intervention in India	AIDS Care - Psychological and Socio-Medical Aspects of AIDS/HIV
Garg, R. : Gupta, A. : Kundal, D. : Singh, S.	2021	Impact of family stigma and caregiver burden on quality of life among wives of patients with alcohol and opioid use disorder	Asian Journal of Pharmaceutical and Clinical Research
Garverich, S. : Prener, C. G. : Guyer, M. E. : Lincoln, A. K.	2021	What matters: Factors impacting the recovery process among outpatient mental health service users	Psychiatric rehabilitation journal
Garzón, J. E. C. : Torres, D. P. C. : Mendoza, E. M. L. : Fernandez, D. Y. B.	2018	Demographic factors, sexual practices and HIV characteristics associated with stigma perception	Enferm. Global
Gaudiano, Brandon A. : Miller, Ivan W.	2013	Self-stigma and attitudes about treatment in depressed patients in a hospital setting	International Journal of Social Psychiatry
Gaur, V. : Salvi, D. : Gautam, M. : Sangwan, V. : Tambi, T. : Kalia, A. : Singh, N.	2022	Psychiatric comorbidity in clinically stable COVID-19 patients	Indian J. Psychiatry
Gaziel, M. : Hasson-Ohayon, I. : Morag-Yaffe, M. : Schapir, L. : Zalsman, G. : Shoval, G.	2015	Insight and satisfaction with life among adolescents with mental disorders: Assessing associations with self-stigma and parental insight	European Psychiatry
Ge, C. : Zhang, H. : Zhu, G. : Cao, A. : Zhang, J.	2021	Intervention study of Snyder's hope theory on the stigma of stroke in young and middle-aged patients: a randomised trial	Annals of palliative medicine
Gelaw, M. M. : Zeleke, E. G. : Asres, M. S. : Reta, M. M.	2020	One-third of perinatal women living with HIV had perinatal depression in gondar town health facilities, northwest Ethiopia	HIV/AIDS - Research and Palliative Care
Gelaye, H. : Andualem, A.	2022	Quality of life and associated factors among family caregivers of individuals with psychiatric illness at DRH, South Wollo, Ethiopia, 2020	Scientific reports
George, S. : McGrath, N.	2019	Social support, disclosure and stigma and the association with non-adherence in the six months after antiretroviral therapy initiation among a cohort of HIV-positive adults in rural KwaZulu-Natal, South Africa*	AIDS Care Psychol. Socio-Med. Asp. AIDS HIV
Getnet, A. : Sintayehu Bitew, M. : Iyasu, A. S. : Afenigus, A. D. : Haile, D. : Amha, H.	2022	Stigma and determinant factors among patients with mental disorders: Institution-based cross-sectional study	SAGE Open Medicine
Getnet, A. : Woldeyohannes, S. M. : Bekana, L. : Mekonen, T. : Fekadu, W. : Menberu, M. : Yimer, S. : Assaye, A. : Belete, A. : Belete, H.	2016	Antiepileptic Drug Nonadherence and Its Predictors among People with Epilepsy	Behav. Neurol.
Ghanean, H. : Jacobsson, L. : Nojomy, M.	2013	Self-perception of stigma in persons with epilepsy in Tehran, Iran	Epilepsy and Behavior
Ghosh, Abhishek : Roub, Fazle : Pillai, Renjith R. : Mahintamani, Tathagata : Basu, Debasish : Subodh, B. N. : Mattoo, S. K.	2022	Course and Correlates of Stigma in Patients on Opioid Agonist Treatment: A Prospective Study from an Outpatient Treatment Program in India	Indian journal of psychological medicine
Ghosh, Papiya : Balasundaram, Sivaprakash : Sankaran, Avudaiappan : Chandrasekaran, Vigneshvar : Sarkar, Sukanto : Choudhury, Sunayana	2022	Factors associated with medication non-adherence among patients with severe mental disorder - A cross sectional study in a tertiary care centre	Exploratory research in clinical and social pharmacy
Gibbons, Carrie : Dubois, Sacha : Morris, Kelly : Parker, Barbara : Maxwell, Hillary : Bédard, Michel	2012	The Development of a Questionnaire to Explore Stigma from the Perspective of Individuals With Serious Mental Illness	Canadian Journal of Community Mental Health
Gierk, B. : Löwe, B. : Murray, A. M. : Kohlmann, S.	2018	Assessment of perceived mental health-related stigma: The Stigma-9 Questionnaire (STIG-9)	Psychiatry Research
Ginsburg, I. H. : Link, B. G.	1993	PSYCHOSOCIAL CONSEQUENCES OF REJECTION AND STIGMA FEELINGS IN PSORIASIS PATIENTS	Int. J. Dermatol.
Girma, E. : Tesfaye, M. : Froeschl, G. : Möller-Leimkühler, A. M. : Dehning, S. : Müller, N.	2013	Facility based cross-sectional study of self stigma among people with mental illness: Towards patient empowerment approach	International Journal of Mental Health Systems
Girma, M. : Wodajo, S. : Ademe, S. : Edmealem, A. : Wsilasie, M. : Mesafint, G.	2020	Health-related quality of life and associated factors among type two diabetic patients on follow-up in dessie comprehensive specialized hospital, dessie, North East Ethiopia, 2020	Diabetes, Metabolic Syndrome and Obesity: Targets and Therapy
Glanz, Bonnie I. : Zurawski, Jonathan : Gonzalez, Cindy T. : Shamah, Rebecca : Ratajska, Adrianna : Chitnis, Tanuja : Weiner, Howard L. : Healy, Brian C.	2020	Comparison of health-related quality of life across treatment groups in individuals with multiple sclerosis	Multiple sclerosis and related disorders
Goepfert, N. C. : Conrad Von Heydendorff, S. : Dreßing, H. : Bailer, J.	2019	Effects of stigmatizing media coverage on stigma measures, self-esteem, and affectivity in persons with depression - An experimental controlled trial	BMC Psychiatry
Göksu, E. Ö : Katı, Ş D.	2021	Internal Stigmatization and Mental Health in Patients with Stroke	Turk Noroloji Dergisi
Golay, P. : Moga, M. : Devas, C. : Staecheli, M. : Poisat, Y. : Israël, M. : Suter, C. : Silva, B. : Morandi, S. : Ferrari, P. : Favrod, J. : Bonsack, C.	2021	Measuring the paradox of self-stigma: psychometric properties of a brief scale	Annals of General Psychiatry
Golden, J. : O'Dwyer, A. M. : Conroy, R. M.	2005	Depression and anxiety in patients with hepatitis C: Prevalence, detection rates and risk factors	General Hospital Psychiatry
Golub, S. A. : Tomassilli, J. C. : Parsons, J. T.	2009	Partner serostatus and disclosure stigma: Implications for physical and mental health outcomes among HIV-positive adults	AIDS and Behavior
Golub, Sarit : Rendina, H. : Gamarel, Kristi	2013	Identity-Related Growth and Loss in a Sample of HIV-Positive Gay and Bisexual Men: Initial Scale Development and Psychometric Evaluation	AIDS & Behavior
Gomes, D. R. A. S. : Zanetti, A. C. G. : Miasso, A. I. : Castro, F. F. S. : Vedana, K. G. G.	2021	Internalized Stigma in People with Mood Disorders: Predictors and Associated Factors	J. Nerv. Ment. Dis.
Gómez, C. A. : Tat, S. A. : Allen, D. : Gordon, D. : Browe, D.	2017	What will it take to end the HIV/AIDS epidemic? Linking the most disenfranchised into care through outreach	AIDS Patient Care STDs
Gómez, L. J. : van Wijk, R. : van Selm, L. : Rivera, A. : Barbosa, M. C. : Parisi, S. : van Brakel, W. H. : Arevalo, J. : Quintero, W. : Waltz, M. : Puchner, K. P.	2020	Stigma, participation restriction and mental distress in patients affected by leprosy, cutaneous leishmaniasis and Chagas disease: a pilot study in two co-endemic regions of eastern Colombia	Trans. R. Soc. Trop. Med. Hyg.
Gonçalves-Pereira, M. : Xavier, M. : van Wijngaarden, B. : Papoila, A. L. : Schene, A. H. : Caldas-de-Almeida, J. M.	2013	Impact of psychosis on Portuguese caregivers: a cross-cultural exploration of burden, distress, positive aspects and clinical-functional correlates	Social psychiatry and psychiatric epidemiology
Gong, Y. : Yan, S. : Qiu, L. : Zhang, S. : Lu, Z. : Tong, Y. : Fang, P. : Yin, X.	2018	Prevalence of depressive symptoms and related risk factors among patients with tuberculosis in China: A multistage cross-sectional study	American Journal of Tropical Medicine and Hygiene

Gonzales, Lauren : Yanos, Philip T. : Stefancic, Ana : Alexander, Mary Jane : Harney-Delehanty, Brianna	2018	The Role of Neighborhood Factors and Community Stigma in Predicting Community Participation Among Persons With Psychiatric Disabilities	Psychiatric Services
Gonzalez, J. M. : Perlick, D. A. : Miklowitz, D. J. : Kaczynski, R. : Hernandez, M. : Rosenheck, R. A. : Culver, J. L. : Ostacher, M. J. : Bowden, C. L. : Gonzalez, Jodi M. : Perlick, Deborah A. : Miklowitz, David J. : Kaczynski, Richard : Hernandez, Melissa : Rosenheck, Robert A. : Culver, Jenifer L. : Ostacher, Michael J. : Bowden, Charles L.	2007	Factors associated with stigma among caregivers of patients with bipolar disorder in the STEP-BD study	Psychiatric Services
González-Menéndez, A. : Faedo, T. A. : González-Pando, D. : Ordoñez-Camblor, N. : García-Vega, E. : Paino, M.	2021	Psychological inflexibility in people with chronic psychosis: The mediating role of self-stigma and social functioning	International Journal of Environmental Research and Public Health
Goodin, B. R. : Owens, M. A. : White, D. M. : Strath, L. J. : Gonzalez, C. : Rainey, R. L. : Okunbor, J. I. : Heath, S. L. : Turan, J. M. : Merlin, J. S.	2018	Intersectional health-related stigma in persons living with HIV and chronic pain: implications for depressive symptoms	AIDS Care - Psychological and Socio-Medical Aspects of AIDS/HIV
Gottert, A. : Friedland, B. : Geibel, S. : Nyblade, L. : Baral, S. D. : Kentutsi, S. : Mallouris, C. : Sprague, L. : Hows, J. : Anam, F. : Amanyeiwe, U. : Pulerwitz, J.	2019	The People Living with HIV (PLHIV) Resilience Scale: Development and Validation in Three Countries in the Context of the PLHIV Stigma Index	AIDS & Behavior
Goyal, A. K. : Bakshi, J. : Panda, N. K. : Kapoor, R. : Vir, D. : Kumar, K. : Aneja, P. : Singh, B. : Gupta, M. : Walia, S. S.	2021	Translation and Validation of Shame and Stigma Scale for Head and Neck Cancer into the Hindi Language	J. Maxillofac. Oral Surg.
Goyal, S. : Sudhir, P. M. : Sharma, M. P.	2020	Illness perceptions and health beliefs in persons with common mental disorders	Asian Journal of Psychiatry
Grambal, A. : Prasko, J. : Kamaradova, D. : Latalova, K. : Holubova, M. : Marackova, M. : Ociskova, M. : Slepecky, M.	2016	Self-stigma in borderline personality disorder - Cross-sectional comparison with schizophrenia spectrum disorder, major depressive disorder, and anxiety disorders	Neuropsychiatric Disease and Treatment
Graves, K. D. : Jensen, R. E. : Canar, J. : Perret-Gentil, M. : Leventhal, K. G. : Gonzalez, F. : Caicedo, L. : Jandorf, L. : Kelly, S. : Mandelblatt, J.	2012	Through the lens of culture: Quality of life among Latina breast cancer survivors	Breast Cancer Research and Treatment
Greeff, M. : Uys, L. R. : Wantland, D. : Makoae, L. : Chirwa, M. : Dlamini, P. : Kohi, T. W. : Mullan, J. : Naidoo, J. R. : Cuca, Y. : Holzemer, W. L.	2010	Perceived HIV stigma and life satisfaction among persons living with HIV infection in five African countries: a longitudinal study	International Journal of Nursing Studies
Griffiths, K. M. : Christensen, H. : Jorm, A. F. : Evans, K. : Groves, C.	2004	Effect of web-based depression literacy and cognitive-behavioural therapy interventions on stigmatising attitudes to depression: Randomised controlled trial	Br. J. Psychiatry
Griffiths, Scott : Costa, Daniel S. J. : Boyd, Jennifer E. : Murray, Stuart B. : Mitchison, Deborah : Mond, Jonathan M.	2016	Facilitating research on eating disorders stigma: validation of the internalised stigma of mental illness scale for use in people with eating disorders	Advances in Eating Disorders
Griffiths, S. : Mond, J. M. : Murray, S. B. : Touyz, S.	2015	The prevalence and adverse associations of stigmatization in people with eating disorders	International Journal of Eating Disorders
Griffiths, S. : Mond, J. M. : Murray, S. B. : Thornton, C. : Touyz, S.	2015	Stigma resistance in eating disorders	Social psychiatry and psychiatric epidemiology
Griffiths, Scott : Mond, Jonathan M. : Li, Zhicheng : Gunatilake, Sanduni : Murray, Stuart B. : Sheffield, Jeanie : Touyz, Stephen	2015	Self-stigma of seeking treatment and being male predict an increased likelihood of having an undiagnosed eating disorder	International Journal of Eating Disorders
Grothe, Lydia : Grothe, Matthias : Wingert, Judith : Schomerus, Georg : Speerforck, Sven	2022	Stigma in Multiple Sclerosis: The Important Role of Sense of Coherence and Its Relation to Quality of Life	International Journal of Behavioral Medicine
Grover, S. : Avasthi, A. : Singh, A. : Dan, A. : Neogi, R. : Kaur, D. : Lakdawala, B. : Rozatkar, A. R. : Nebhinani, N. : Patra, S. : Sivashankar, P. : Subramanyam, A. A. : Tripathi, A. : Gania, A. M. : Singh, G. P. : Behere, P.	2017	Stigma experienced by patients with severe mental disorders: A nationwide multicentric study from India	Psychiatry Research
Grover, S. : Avasthi, A. : Singh, A. : Dan, A. : Neogi, R. : Kaur, D. : Lakdawala, B. : Rozatkar, A. R. : Nebhinani, N. : Patra, S. : Sivashankar, P. : Subramanyam, A. A. : Tripathi, A. : Gania, A. M. : Singh, G. P. : Behere, P.	2017	Stigma experienced by caregivers of patients with severe mental disorders: A nationwide multicentric study	The International journal of social psychiatry
Grover, Sandeep : Aneja, Jitender : Hazari, Nandita : Chakrabarti, Subho : Avasthi, Ajit	2019	Stigma and its Correlates among Caregivers of Patients with Bipolar Disorder	Indian journal of psychological medicine
Grover, S. : Hazari, N. : Aneja, J. : Chakrabarti, S. : Sharma, S. : Avasthi, A.	2016	Recovery and its correlates among patients with bipolar disorder: A study from a tertiary care centre in North India	The International journal of social psychiatry
Grover, S. : Hazari, N. : Aneja, J. : Chakrabarti, S. : Avasthi, A.	2016	Stigma and its correlates among patients with bipolar disorder: A study from a tertiary care hospital of North India	Psychiatry Research
Grover, Sandeep : Mehra, Aseem : Dogra, Sunil : Hazari, Nandita : Malhora, Nidhi : Narang, Tarun : Sahoo, Swapanjeet : Sharma, Sunil : Handa, Sanjeev : Avasthi, Ajit	2021	Internalized Stigma and Psychiatric Morbidity among Patients with Psoriasis: A Study from North India	Indian dermatology online journal
Grover, S. : Sahoo, S. : Chakrabarti, S. : Avasthi, A.	2018	Association of internalized stigma and insight in patients with schizophrenia	Int. J. Cult. Ment. Health
Grover, S. : Sahoo, S. : Nehra, R.	2019	A comparative study of childhood/adolescent and adult onset schizophrenia: does the neurocognitive and psychosocial outcome differ?	Asian Journal of Psychiatry
Grover, S. : Sahoo, S. : Mishra, E. : Gill, K. S. : Mehra, A. : Nehra, R. : Suman, A. : Bhalla, A. : Puri, G. D.	2021	Fatigue, perceived stigma, self-reported cognitive deficits and psychological morbidity in patients recovered from COVID-19 infection	Asian Journal of Psychiatry
Gruber, M. T. : Witte, O. W. : Grosskreutz, J. : Prell, T.	2020	Association between malnutrition, clinical parameters and health-related quality of life in elderly hospitalized patients with Parkinson's disease: A cross-sectional study	PLoS ONE
Guadagnoli, L. : Taft, T. H. : Keefer, L.	2017	Stigma perceptions in patients with eosinophilic gastrointestinal disorders	Diseases of the Esophagus
Guadagnoli, L. : Taft, T. H.	2020	Internalized Stigma in Patients with Eosinophilic Gastrointestinal Disorders	Journal of Clinical Psychology in Medical Settings
Guan, Ziyao : Huang, Chongmei : Wiley, James A. : Sun, Mei : Bai, Xiaoling : Tang, Siyuan	2020	Internalized stigma and its correlates among family caregivers of patients diagnosed with schizophrenia in Changsha, Hunan, China	Journal of Psychiatric & Mental Health Nursing (John Wiley & Sons, Inc.)
Guan, Z. : Wang, Y. : Lam, L. : Cross, W. : Wiley, J. A. : Huang, C. : Bai, X. : Sun, M. : Tang, S.	2021	Severity of illness and distress in caregivers of patients with schizophrenia: Do internalized stigma and caregiving burden mediate the relationship?	Journal of advanced nursing
Guízar, D. : Fresán, A. : Saracco, R. : Escamilla, R. : Almanza, F. : Robles-García, R. : Tovilla-Zarate, C. A.	2015	Personality traits and internalized stigma in patients with schizophrenia	Personal. Traits and Types: Perceptions, Gend. Differ. and Impact on Behav.
Gul, Zeynep Bastug : Atakli, Hayrunisa Dilek	2021	Effect of the COVID-19 pandemic on drug compliance and stigmatization in patients with epilepsy	Epilepsy & behavior : E&B
Guo, H. : Ren, Y. : Huang, B. : Wang, J. : Yang, X. : Wang, Y.	2022	Psychological Status, Compliance, Serum Brain-Derived Neurotrophic Factor, and Nerve Growth Factor Levels of Patients with Depression after Augmented Mindfulness-Based Cognitive Therapy	Genetics research
Guo, Yi : Qu, Shumin : Qin, Hongyun	2018	Study of the relationship between self-stigma and subjective quality of life for individuals with chronic schizophrenia in the community	General psychiatry
Guo, Y. : Wichaidit, W. : Du, Y. : Liu, J. : Chongsuvivatwong, V.	2022	Mediation of the association between stigma and HIV status and fertility intention by fertility desire among heterosexual couples living with HIV in Kunming, China	PLoS ONE
Gupta, A. K. : Venkataraman, M. : Quinlan, E. M. : Gupta, M. A. : Anbalagan, N. : Lyons, R.	2021	More than nail deep: The effect of efinaconazole 10% treatment on the quality of life in patients with onychomycosis: A post hoc study	Skin Appendage Disorders
Gupta, Nilu : Mohanty, Sandhyrani	2016	Stigma and expressed emotion in spouses of schizophrenic patients	Indian Journal of Community Psychology
Gupta, P.	2018	Internalized stigma and its correlates among treatment seeking natural opiates users in india: A cross-sectional observational study	Indian Journal of Psychiatry
Gupta, R. : Avasthi, A. : Chawla, Y. K. : Grover, S.	2020	Psychiatric Morbidity, Fatigue, Stigma and Quality of Life of Patients With Hepatitis B Infection	Journal of Clinical and Experimental Hepatology
Gyawali, S. : Sarkar, S. : Balhara, Y. P. S. : Kumar, S. : Patil, V. : Singh, S.	2018	Perceived stigma and its correlates among treatment seeking alcohol and opioid users at a tertiary care centre in India	Asian Journal of Psychiatry
Habib, Z. : Kausar, R. : Kamran, F.	2021	Handling traumatic experiences in facially disfigured female burn survivors	Burns
Hacimusalar, Y. : Sezgin Doğan, E.	2019	Assessment of the functioning levels and related factors in patients with bipolar disorder during remission	Noropsikiyatri Arsivi
Hack, S. M. : Muralidharan, A. : Brown, C. H. : Drapalski, A. L. : Lucksted, A. A.	2020	Stigma and discrimination as correlates of mental health treatment engagement among adults with serious mental illness	Psychiatr. Rehab. J.
Hadera, Etsedingl : Salelew, Endalamaw : Girma, Eshetu : Dehning, Sandra : Adorjan, Kristina : Tesfaye, Markos	2019	Magnitude and Associated Factors of Perceived Stigma among Adults with Mental Illness in Ethiopia	Psychiatry journal
Haghighat, Rahman	2005	The development of an instrument to measure stigmatization: Factor analysis and origin of stigmatization	The European Journal of Psychiatry
Hain, B. : Langer, L. : Hünnemeyer, K. : Rudofsky, G. : Zech, U. : Wild, B.	2015	Translation and Validation of the German Version of the Weight Self-Stigma Questionnaire (WSSQ)	Obesity Surgery
Hajda, M. : Kamaradova, D. : Latalova, K. : Prasko, J. : Ociskova, M. : Mainerova, B. : Cinculova, A. : Vrbova, K. : Kubinek, R. : Tichackova, A.	2015	Self-stigma, treatment adherence, and medication discontinuation in patients with bipolar disorders in remission - a cross sectional study	Activitas Nervosa Superior Rediviva
Halada, S. : Baran, J. A. : Isaza, A. : Patel, T. : Sisko, L. : Kazahaya, K. : Adzick, N. S. : Katowitz, W. R. : Magee, L. : Bauer, A. J.	2022	Impact of Definitive Surgery for Graves' Disease on Adolescent Disease-Specific Quality of Life and Psychosocial Functioning	Thyroid : official journal of the American Thyroid Association
Halkitis, P. N. : Perez-Figueroa, R. E. : Carreiro, T. : Kingdon, M. J. : Kupprat, S. A. : Eddy, J.	2014	Psychosocial burdens negatively impact HIV antiretroviral adherence in gay, bisexual, and other men who have sex with men aged 50 and older	AIDS Care Psychol. Socio-Med. Asp. AIDS HIV

Hamann, Heidi A. : Shen, Megan J. : Thomas, Anna J. : Craddock Lee, Simon J. : Ostroff, Jamie S.	2018	Development and Preliminary Psychometric Evaluation of a Patient-Reported Outcome Measure for Lung Cancer Stigma: The Lung Cancer Stigma Inventory (LCSI)	Stigma and health
Hamann, J. : Bühner, M. : Rüsch, N.	2017	Self-stigma and consumer participation in shared decision making in mental health services	Psychiatr. Serv.
Hamilton, Alexander James : Caskey, Fergus J. : Casula, Anna : Inward, Carol D. : Ben-Shlomo, Yoav	2018	Associations with Wellbeing and Medication Adherence in Young Adults Receiving Kidney Replacement Therapy	Clinical journal of the American Society of Nephrology : CJASN
Hamilton, J. B. : Deal, A. M. : Moore, A. D. : Best, N. C. : Galbraith, K. V. : Muss, H.	2013	Psychosocial predictors of depression among older African American patients with cancer	Oncology Nursing Forum
Hamra, M. : Ross, M. W. : Karuri, K. : Orrs, M. : D'Agostino, A.	2005	The relationship between expressed HIV/AIDS-related stigma and beliefs and knowledge about care and support of people living with AIDS in families caring for HIV-infected children in Kenya	AIDS Care Psychol. Socio-Med. Asp. AIDS HIV
Hamra, M. : Ross, M. W. : Orrs, M. : D'Agostino, A.	2006	Relationship between expressed HIV/AIDS-related stigma and HIV-beliefs/knowledge and behaviour in families of HIV infected children in Kenya	Tropical Medicine and International Health
Han, S. H. : Kim, B. : Lee, S. A.	2015	Contribution of the family environment to depression in Korean adults with epilepsy	Seizure
Han, S. H. : Kim, K. T. : Ryu, H. U. : Lee, S. A. : Cho, Y. J. : Kim, J. H. : Kang, K. W. : Shin, D. J. : Lee, G. H. : Hwang, K. J. : Kim, Y. S. : Kim, J. B. : Kim, J. E. : Lee, S. Y. : Seo, J. G.	2019	Factors associated with social anxiety in South Korean adults with epilepsy	Epilepsy and Behavior
Han, Y. : Zhong, R. : Yang, J. : Guo, X. : Zhang, H. : Zhang, X. : Liu, Y. : Lin, W.	2023	Alexithymia and related factors among patients with epilepsy	Epilepsy and Behavior
Hansen, B. : Szaflarski, M. : Bebin, E. M. : Szaflarski, J. P.	2018	Affiliate stigma and caregiver burden in intractable epilepsy	Epilepsy and Behavior
Hansen, U. M. : Olesen, K. : Willaing, I.	2020	Diabetes stigma and its association with diabetes outcomes: a cross-sectional study of adults with type 1 diabetes	Scandinavian journal of public health
Hansen, U. M. : Willaing, I. : Ventura, A. D. : Olesen, K. : Speight, J. : Browne, J. L.	2018	Stigma Perceived and Experienced by Adults with Type 1 Diabetes: Linguistic Adaptation and Psychometric Validation of the Danish Version of the Type 1 Diabetes Stigma Assessment Scale (DSAS-1 DK)	Patient
Hansson, L. M. : Rasmussen, F.	2014	Association between perceived health care stigmatization and BMI change	Obes. Facts
Harangozo, J. : Reneses, B. : Brohan, E. : Sebes, J. : Csukly, G. : López-Ibor, J. J. : Sartorius, N. : Rose, D. : Thornicroft, G.	2014	Stigma and discrimination against people with schizophrenia related to medical services	International Journal of Social Psychiatry
Hargreaves, J. R. : Krishnaratne, S. : Mathema, H. : Lilleston, P. S. : Sievwright, K. : Mandla, N. : Mainga, T. : Vermaak, R. : Piwowar-Manning, E. : Schaap, A. : Donnell, D. : Ayles, H. : Hayes, R. J. : Hoddinott, G. : Bond, V. : Stangl, A.	2018	Individual and community-level risk factors for HIV stigma in 21 Zambian and South African communities: Analysis of data from the HPTN071 (PopART) study	AIDS
Hariz, G. M. : Limousin, P. : Zrinzo, L. : Tripoliti, E. : Aviles-Olmos, I. : Jahanshahi, M. : Hamberg, K. : Foltynie, T.	2013	Gender differences in quality of life following subthalamic stimulation for Parkinson's disease	Acta Neurologica Scandinavica
Harper, G. W. : Lemos, D. : Hosek, S. G.	2014	Stigma reduction in adolescents and young adults newly diagnosed with HIV: Findings from the project ACCEPT intervention	AIDS Patient Care and STDs
Harper, K. J. : Osborn, C. Y. : Mayberry, L. S.	2018	Patient-perceived family stigma of Type 2 diabetes and its consequences	Families, systems & health : the journal of collaborative family healthcare
Harpur, R. A. : Thompson, M. : Daley, D. : Abikoff, H. : Sonuga-Barke, E. J. S.	2008	The attention-deficit/hyperactivity disorder medication-related attitudes of patients and their parents	Journal of Child and Adolescent Psychopharmacology
Harris, Lesley M. : Crawford, Timothy N. : Kerr, Jelani C. : Thomas, Tammi Alvey : Schmidt, Verena	2020	African American Older Adults Living with HIV: Exploring Stress, Stigma, and Engagement in HIV Care	Journal of Health Care for the Poor & Underserved
Hasan, A. A. : Alasmee, N.	2022	Evaluation of the impact of a self-stigma reduction programme on psychosocial outcomes among people with schizophrenia spectrum disorder	J. Ment. Health
Hasan Shiri, F. : Mohtashami, J. : Nasiri, M. : Manoochehri, H. : Rohani, C.	2018	Stigma and Related Factors in Iranian People with Cancer	Asian Pacific journal of cancer prevention : APJCP
Hasson-Ohayon, I. : Ehrlich-Ben Or, S. : Vahab, K. : Amiaz, R. : Weiser, M. : Roe, D.	2012	Insight into mental illness and self-stigma: The mediating role of shame proneness	Psychiatry Res.
Hawro, M. : Maurer, M. : Weller, K. : Maleszka, R. : Zalewska-Janowska, A. : Kaszuba, A. : Gerlicz-Kowalczuk, Z. : Hawro, T.	2017	Lesions on the back of hands and female gender predispose to stigmatization in patients with psoriasis	J. Am. Acad. Dermatol.
Hayes-Larson, E. : Hirsch-Moverman, Y. : Saito, S. : Frederix, K. : Pitt, B. : Maama-Maime, L. : Howard, A. A.	2017	Depressive symptoms and hazardous/harmful alcohol use are prevalent and correlate with stigma among TB-HIV patients in Lesotho	International Journal of Tuberculosis and Lung Disease
Hayward, P. : Wong, G. : Bright, J. A. : Lam, D.	2002	Stigma and self-esteem in manic depression: An exploratory study	Journal of Affective Disorders
Hazumi, M. : Okazaki, E. : Usuda, K. : Kataoka, M. : Nishi, D.	2022	Relationship between attitudes toward COVID-19 infection, depression and anxiety: a cross-sectional survey in Japan	BMC Psychiatry
He, S. : Ke, X. J. : Wu, Y. : Kong, X. Y. : Wang, Y. : Sun, H. Q. : Xia, D. Z. : Chen, G. H.	2022	The stigma of patients with chronic insomnia: a clinical study	BMC Psychiatry
He, Y. Y. : Yin, A. C.	2021	Research on the effects of family care degree on stigma and psychosocial adaptation among the patients with Parkinson's disease†	Front. Nurs.
Hedayat, K. : Karbakhsh, M. : Ghiasi, M. : Goodarzi, A. : Fakour, Y. : Akbari, Z. : Ghayoumi, A. : Ghandi, N.	2016	Quality of life in patients with vitiligo: A cross-sectional study based on Vitiligo Quality of Life index (VitiQoL)	Health and Quality of Life Outcomes
Heggeness, Luke F. : Brandt, Charles P. : Paulus, Daniel J. : Lemaire, Chad : Zvolensky, Michael J.	2017	Stigma and disease disclosure among HIV+ individuals: the moderating role of emotion dysregulation	AIDS Care
Heinz, I. : Baldofski, S. : Beesdo-Baum, K. : Knappe, S. : Kohls, E. : Rummel-Kluge, C.	2021	"Doctor, my back hurts and I cannot sleep." Depression in primary care patients: Reasons for consultation and perceived depression stigma	PLoS ONE
Hémar, V. : Hessamfar, M. : Neau, D. : Vareil, M. O. : Rouanes, N. : Lazaro, E. : Duffau, P. : Cazanave, C. : Rispal, P. : Gaborieau, V. : Leleux, O. : Wittkop, L. : Bonnet, F. : Barger, D.	2022	A comprehensive analysis of excess depressive disorder in women and men living with HIV in France compared to the general population	Scientific reports
Henning, O. : Buer, C. : Nakken, K. O. : Lossius, M. I.	2021	People with epilepsy still feel stigmatized	Acta Neurologica Scandinavica
Heredia Montesinos, A. : Rapp, M. A. : Temur-Erman, S. : Heinz, A. : Hegerl, U. : Schouler-Ocak, M.	2012	The influence of stigma on depression, overall psychological distress, and somatization among female Turkish migrants	European Psychiatry
Hermann, Bruce P. : Whitman, Steven : Wyler, Allen R. : Anton, Michael T. : Vanderzwagg, R.	1990	Psychosocial predictors of psychopathology in epilepsy	The British Journal of Psychiatry
Hermanns, Melinda : Mastel-Smith, Beth : Donnell, Rachel : Quarles, Allison : Rodriguez, Melissa : Wang, Tianjing	2021	Counterpunching to improve the health of people with Parkinson's disease	Journal of the American Association of Nurse Practitioners
Hermanns, N. : Lilly, L. C. : Mader, J. K. : Aberer, F. : Ribitsch, A. : Kojzar, H. : Warner, J. : Pieber, T. R.	2015	Novel simple insulin delivery device reduces barriers to insulin therapy in type 2 diabetes: Results from a pilot study	Journal of Diabetes Science and Technology
Hernansaiz-Garrido, Helena : Alonso-Tapia, Jesús	2017	Internalized HIV Stigma and Disclosure Concerns: Development and Validation of Two Scales in Spanish-Speaking Populations	AIDS & Behavior
Herrmann, S. : McKinnon, E. : Hyland, N. B. : Lalanne, C. : Mallal, S. : Nolan, D. : Chassany, O. : Duracinsky, M.	2013	HIV-related stigma and physical symptoms have a persistent influence on health-related quality of life in Australians with HIV infection	Health and Quality of Life Outcomes
Heylen, Elsa : Panicker, Siju : Chandy, Sara : Steward, Wayne : Ekstrand, Maria	2015	Food Insecurity and Its Relation to Psychological Well-Being Among South Indian People Living with HIV	AIDS & Behavior
Hibbert, M. : Wolton, A. : Crenna-Jennings, W. : Benton, L. : Kirwan, P. : Lut, I. : Okala, S. : Ross, M. : Furegato, M. : Nambiar, K. : Douglas, N. : Roche, J. : Jeffries, J. : Reeves, I. : Nelson, M. : Weerawardhana, C. : Jamal, Z. : Hudson, A. : Delpech, V.	2018	Experiences of stigma and discrimination in social and healthcare settings among trans people living with HIV in the UK	AIDS Care - Psychological and Socio-Medical Aspects of AIDS/HIV
Hickey, M. D. : Ouma, G. B. : Mattah, B. : Pederson, B. : DesLauriers, N. R. : Mohamed, P. : Obanda, J. : Odhiambo, A. : Njoroge, B. : Otieno, L. : Zoughbie, D. E. : Ding, E. L. : Fiorella, K. J. : Bukusi, E. A. : Cohen, C. R. : Geng, E. H. : Salmen, C. R.	2021	The Kanyakla study: Randomized controlled trial of a microclinic social network intervention for promoting engagement and retention in HIV care in rural western Kenya	PLoS ONE
Hickman, Enith E. : Glass, Carol R. : Arnkoff, Diane B. : Fallot, Roger D.	2013	Religious coping, stigma, and psychological functioning among HIV-positive African American women	Mental Health, Religion & Culture
Hippman, C. : Ringrose, A. : Inglis, A. : Cheek, J. : Albert, A. Y. K. : Remick, R. : Honer, W. G. : Austin, J. C.	2016	A pilot randomized clinical trial evaluating the impact of genetic counseling for serious mental illnesses	Journal of Clinical Psychiatry
Ho, A. H. Y. : Potash, J. S. : Fong, T. C. T. : Ho, V. F. L. : Chen, E. Y. H. : Lau, R. H. W. : Au Yeung, F. S. W. : Ho, R. T. H.	2015	Psychometric properties of a Chinese version of the Stigma Scale: Examining the complex experience of stigma and its relationship with self-esteem and depression among people living with mental illness in Hong Kong	Comprehensive Psychiatry
Ho, Ryan Wui Hang : Chang, Wing Chung : Kwong, Vivian Wing Yan : Lau, Emily Sin Kei : Chan, Gloria Hoi Kei : Jim, Olivia Tsz Ting : Hui, Christy Lai Ming : Chan, Sherry Kit Wa : Lee, Edwin Ho Ming : Chen, Eric Yu Hai	2018	Prediction of self-stigma in early psychosis: 3-Year follow-up of the randomized-controlled trial on extended early intervention	Schizophrenia research
Ho, W. W. N. : Chiu, M. Y. L. : Lo, W. T. L. : Yiu, M. G. C.	2010	Recovery components as determinants of the health-related quality of life among patients with schizophrenia: structural equation modelling analysis	Australian & New Zealand Journal of Psychiatry
Hoang, D. : Dinh, A. T. : Groce, N. : Sullivan, L. E.	2015	Knowledge and perceptions of HIV-infected patients regarding HIV transmission and treatment in Ho Chi Minh City, Vietnam	Asia-Pacific journal of public health / Asia-Pacific Academic Consortium for Public Health

Hobson, Joanna M. : Gilstrap, Shannon R. : Owens, Michael A. : Gloston, Gabrielle F. : Ho, Michael D. : Gathright, Jenna M. : Dotson, Hannah F. : White, Dyan M. : Cody, Shameka L. : Justin Thomas, S. : Goodin, Burel R.	2022	Intersectional HIV and Chronic Pain Stigma: Implications for Mood, Sleep, and Pain Severity	Journal of the International Association of Providers of AIDS Care
Hofer, A. : Mizuno, Y. : Frajo-Apor, B. : Kemmler, G. : Suzuki, T. : Pardeller, S. : Welte, A. S. : Sondermann, C. : Mimura, M. : Wartelsteiner, F. : Fleischhacker, W. W. : Uchida, H.	2016	Resilience, internalized stigma, self-esteem, and hopelessness among people with schizophrenia: Cultural comparison in Austria and Japan	Schizophrenia Research
Hoffman, S. : Tymejczyk, O. : Kulkarni, S. : Lahuerta, M. : Gadisa, T. : Remien, R. H. : Melaku, Z. : Nash, D. : Elul, B.	2017	Stigma and HIV care continuum outcomes among ethiopian adults initiating ART	J. Acquired Immune Defic. Syndr.
Hojilla, J. C. : Santiago-Rodriguez, E. I. : Sterling, S. : Williams, E. C. : Leyden, W. : Hare, C. B. : Silverberg, M. J. : Satre, D. D.	2021	HIV Stigma and Its Associations with Longitudinal Health Outcomes Among Persons Living with HIV with a History of Unhealthy Alcohol Use	AIDS Behav.
Holmes-Truscott, E. : Browne, J. L. : Ventura, A. D. : Pouwer, F. : Speight, J.	2018	Diabetes stigma is associated with negative treatment appraisals among adults with insulin-treated Type 2 diabetes: results from the second Diabetes MILES - Australia (MILES-2) survey	Diabet. Med.
Holmes-Truscott, E. : Ventura, A. D. : Thuraisingam, S. : Pouwer, F. : Speight, J.	2020	Psychosocial moderators of the impact of diabetes stigma: Results from the second diabetes miles - australia (miles-2) study	Diabetes Care
Holtz, Carol : Sowell, Richard : VanBrackle, Lewis : Velasquez, Gabriela : Hernandez-Alonso, Virginia	2014	A Quantitative Study of Factors Influencing Quality of Life in Rural Mexican Women Diagnosed With HIV	JANAC: Journal of the Association of Nurses in AIDS Care
Holubova, M. : Prasko, J. : Matousek, S. : Latalova, K. : Marackova, M. : Vrbova, K. : Grambal, A. : Slepecky, M. : Zatkova, M.	2016	Comparison of self-stigma and quality of life in patients with depressive disorders and schizophrenia spectrum disorders - A cross-sectional study	Neuropsychiatric Disease and Treatment
Holubova, M. : Prasko, J. : Ociskova, M. : Marackova, M. : Grambal, A. : Slepecky, M.	2016	Self-stigma and quality of life in patients with depressive disorder: A cross-sectional study	Neuropsychiatric Disease and Treatment
Holubova, M. : Prasko, J. : Hruby, R. : Latalova, K. : Kamaradova, D. : Marackova, M. : Slepecky, M. : Gubova, T.	2016	Coping strategies and self-stigma in patients with schizophrenia-spectrum disorders	Patient Preference and Adherence
Holubova, M. : Prasko, J. : Latalova, K. : Ociskova, M. : Grambal, A. : Kamaradova, D. : Vrbova, K. : Hruby, R.	2016	Are self-stigma, quality of life, and clinical data interrelated in schizophrenia spectrum patients? A cross-sectional outpatient study	Patient Preference and Adherence
Holubova, M. : Prasko, J. : Ociskova, M. : Vanek, J. : Slepecky, M. : Zatkova, M. : Latalova, K. : Kolek, A.	2018	Three diagnostic psychiatric subgroups in comparison to self-stigma, quality of life, disorder severity and coping management cross-sectional outpatient study	Neuroendocrinology Letters
Holubova, Michaela : Prasko, Jan : Ociskova, Marie : Kantor, Krystof : Vanek, Jakub : Slepecky, Milos : Vrbova, Kristyna	2019	Quality of life, self-stigma, and coping strategies in patients with neurotic spectrum disorders: a cross-sectional study	Psychology research and behavior management
Holubova, M. : Prasko, J. : Hodny, F. : Vanek, J. : Slepecky, M. : Nesnidal, V.	2021	Self-stigma, severity of psychopatology, dissociation, parental style and comorbid personality disorder in patient with neurotic spectrum disorders Part 2: Therapeutic efficacy of intensive psychotherapeutic inpatients program	Neuro endocrinology letters
Holzemer, W. L. : Uys, L. R. : Chirwa, M. L. : Greeff, M. : Makoae, L. N. : Kohi, T. W. : Dlamini, P. S. : Stewart, A. L. : Mullan, J. : Phetlhu, R. D. : Wantland, D. : Durrheim, K.	2007	Validation of the HIV/AIDS Stigma Instrument - PLWA (HASI-P)	AIDS Care - Psychological and Socio-Medical Aspects of AIDS/HIV
Honeyman, C. : Patel, V. : Yonis, E. : Fell, M. : Demissie, Y. : Eshete, M. : Martin, D. : McGurk, M.	2020	Long-term outcomes associated with short-term surgical missions treating complex head and neck disfigurement in Ethiopia: A retrospective cohort study	Journal of plastic, reconstructive & aesthetic surgery : JPRAS
Hook, K. : Sereda, Y. : Rossi, S. : Koberna, S. : Vetrova, M. V. : Lodi, S. : Lunze, K.	2022	HIV, substance use, and intersectional stigma: Associations with mental health among persons living with HIV who inject drugs in Russia	AIDS and behavior
Horsselenberg, E. M. A. : Van Busschbach, J. T. : Aleman, A. : Pijnenborg, G. H. M.	2016	Self-stigma and its relationship with victimization, psychotic symptoms and self-esteem among people with schizophrenia spectrum disorders	PLoS ONE
Hosaka, K. : Mmbaga, B. T. : Shayo, A. : Gallis, J. A. : Turner, E. L. : O'Donnell, K. E. : Cunningham, C. K. : Boshe, J. J. : Dow, D. E.	2022	A Group-based Mental Health Intervention for Youth Living with HIV in Northern Tanzania: Secondary Analyses of a Pilot Trial	Pediatrics
Hoseinzadeh, Farzaneh : Miri, Sakineh : Foroughameri, Golnaz : Farokhzadian, Jamileh : Eslami Shahrbabaki, Mahin	2022	Stigma, burden of care, and family functioning in family caregivers of people with mental illnesses: a cross-sectional questionnaire survey	Social Work in Mental Health
Hou, M. : Mao, X. : Hou, X. : Li, K.	2021	Stigma and Associated Correlates of Elderly Patients With Parkinson's Disease	Frontiers in Psychiatry
Howland, M. : Levin, J. : Blixen, C. : Tatsuoka, C. : Sajatovic, M.	2016	Mixed-methods analysis of internalized stigma correlates in poorly adherent individuals with bipolar disorder	Comprehensive Psychiatry
Hrehorow, Ewa : Salomon, Joanna : Matusiak, Lukasz : Reich, Adam : Szepietowski, Jacek C.	2012	Patients with psoriasis feel stigmatized	Acta dermato-venereologica
Hsiao, C. Y. : Lu, H. L. : Tsai, Y. F.	2018	Effect of family sense of coherence on internalized stigma and health-related quality of life among individuals with schizophrenia	International journal of mental health nursing
Hu, R. : Wang, X. : Liu, Z. : Hou, J. : Liu, Y. : Tu, J. : Jia, M. : Liu, Y. : Zhou, H.	2022	Stigma, depression, and post-traumatic growth among Chinese stroke survivors: A longitudinal study examining patterns and correlations	Topics in Stroke Rehabilitation
Huang, C. H. : Li, S. M. : Shu, B. C.	2016	Exploring the Relationship Between Illness Perceptions and Negative Emotions in Relatives of People With Schizophrenia Within the Context of an Affiliate Stigma Model	The journal of nursing research : JNR
Huang, Feifei : Chen, Wei-Ti : Shiu, Cheng-Shi : Sun, Wenxiu : Toma, Lance : Luu, Binh Vinh : Ah-Yune, Judy	2020	Acculturation, HIV-Related Stigma, Stress, and Patient-Healthcare Provider Relationships Among HIV-Infected Asian Americans: A Path Analysis	Journal of Immigrant & Minority Health
Huang, F. : Chen, W. T. : Shiu, C. S. : Lin, S. H. : Tun, M. S. : Nwe, T. W. : Oo, Y. T. N. : Oo, H. N.	2021	Adaptation and validation of a culturally adapted HIV stigma scale in Myanmar	BMC Public Health
Huang, F. : Sun, W. : Zhang, L. : Lu, H. : Chen, W. T.	2022	Depressive symptoms mediate COVID-associated stigma and quality of life: Stigma instrument validation and path analysis	Journal of Affective Disorders
Huang, J. : Chagpar, A.	2018	Quality of life and body image as a function of time from mastectomy	Annals of Surgical Oncology
Huang, J. : Lin, D. : Yu, N. X.	2019	Variations in Dyadic Adjustment among Heterosexual HIV-Discordant Couples in Rural China: A Latent Profile Analysis	AIDS Patient Care and STDs
Huang, K. : Wu, Y. : He, Q. : Yang, H. : Du, Y. : Xiao, B. : Feng, L.	2022	Validity and reliability of the Chinese version of the epilepsy stigma scale	Epilepsy and Behavior
Huang, W. Y. : Chen, S. P. : Pakpour, A. H. : Lin, C. Y.	2018	The mediation role of self-esteem for self-stigma on quality of life for people with schizophrenia: A retrospectively longitudinal study	J. Pac. Rim Psychol.
Huang, Y. C. : Fan, J. Y. : Ro, L. S. : Lyu, R. K. : Chang, H. S. : Chen, S. T. : Hsu, W. C. : Chen, C. M. : Wu, Y. R.	2009	Validation of a Chinese version of disease specific quality of life scale (HFS-36) for hemifacial spasm in Taiwan	Health and Quality of Life Outcomes
Hübner, C. : Schmidt, R. : Selle, J. : Köhler, H. : Müller, A. : De Zwaan, M. : Hilbert, A.	2016	Comparing self-report measures of internalized weight stigma: The weight self-stigma questionnaire versus the weight bias internalization scale	PLoS ONE
Huis In 't Veld, D. : Pengpid, S. : Colebunders, R. : Peltzer, K.	2018	Body Mass Index and Waist Circumference in Patients with HIV in South Africa and Associated Socio-demographic, Health Related and Psychosocial Factors	AIDS and behavior
Hunt, W. T. N. : Hùng, N. T. : Tru'Ò'Ng, N. N. : Nikolaou, V. : Khoa, N. D. D. : Hôngly, T.	2018	A case-control study comparing the Dermatology Life Quality Index (DLQI) ratings of patients undergoing leprosy treatment, people cured of leprosy, and controls in Vietnam	Lepr. Rev.
Hutson, Sadie P. : Darlington, Caroline K. : Hall, Joanne M. : Heidel, R. Eric : Gaskins, Susan	2018	Stigma and Spiritual Well-being among People Living with HIV/AIDS in Southern Appalachia	Issues in Mental Health Nursing
Hyland, Kelly A. : Small, Brent J. : Gray, Jhanelle E. : Chiappori, Alberto : Creelan, Ben C. : Tanvetyanon, Tawee : Nelson, Ashley M. : Cessna-Palas, Julie : Jim, Heather S. L. : Jacobsen, Paul B.	2019	Loneliness as a mediator of the relationship of social cognitive variables with depressive symptoms and quality of life in lung cancer patients beginning treatment	Psycho-oncology
Ibikunle, Peter Olanrewaju : Nwokeji, Stella Chioma	2017	Assessment of stigma among people living with Hansen's disease in south-east Nigeria	Leprosy review
Ibrahim, Kusman : Kombong, Rita : Sriati, Aat	2019	The Difference of Perceived HIV Stigma between People Living with HIV Infection and Their Families	Nurse Media Journal of Nursing
Ibrahim, K. : Lindayani, L. : Emaliyawati, E. : Rahayu, U. : Nuraeni, A.	2020	Factors associated with adherence to antiretroviral therapy among people living with HIV infection in West Java Province, Indonesia	Malays. J. Med. Health Sci.
Idemudia, E. S. : Olasupo, M. O. : Modibo, M. W.	2018	Stigma and chronic illness: A comparative study of people living with HIV and/or AIDS and people living with hypertension in Limpopo Province, South Africa	Curationis
Ignatova, D. : Kamusheva, M. : Petrova, G. : Onchev, G.	2019	Burden of informal care for individuals with schizophrenia and affective disorders prior to hospital admission	European Journal of Psychiatry
Ignatova, D. : Kamusheva, M.	2022	THE BURDEN OF CARE AND ITS PREDICTORS IN SEVERE MENTAL ILLNESS: A FOLLOW-UP ONE YEAR AFTER HOSPITAL DISCHARGE	C. R. Acad. Bulgare Sci.
Ihalainen, N. : Loyttyniemi, E. : Valimaki, M.	2022	Self-stigma among clients of outpatient psychiatric clinics: A cross-sectional survey	PLoS ONE
Ilic, Marie : Reinecke, Jost : Bohner, Gerd : Hans-Onno, Röttgers : Beblo, Thomas : Driessen, Martin : Frommberger, Ulrich : Corrigan, Patrick William	2012	Protecting self-esteem from stigma: A test of different strategies for coping with the stigma of mental illness	International Journal of Social Psychiatry
Imran, N. : Afzal, H. : Aamer, I. : Hashmi, A. : Shabbir, B. : Asif, A. : Farooq, S.	2020	Scarlett letter: A study based on experience of stigma by COVID-19 patients in quarantine	Pakistan Journal of Medical Sciences

İnanç, L. : Ünal, Y. : Semiz, Ü B. : Kutlu, G.	2018	Do mentalization skills affect the perception of stigma in patients with epilepsy?	Epilepsy and Behavior
Ingvorsen, Emilie B. : Schnohr, Christina : Andersen, Terkel : Lehrmann, Lars : Funding, Eva : Poulsen, Lone H. : Holm, Karen B. : Laursen, Alex L. : Gerstoft, Jan : Bjorner, Jakob B.	2019	"Development in well-being and social function among Danish hemophilia patients with HIV: a three-wave panel study spanning 24 years"	BMC Public Health
İnkaya, B. : Karadağ, E.	2021	Turkish validity and reliability study of type 2 diabetes stigma assessment scale	Turkish Journal of Medical Sciences
Interian, A. : Ang, A. : Gara, M. A. : Link, B. G. : Rodriguez, M. A. : Vega, W. A.	2010	Stigma and depression treatment utilization among latinos: Utility of four stigma measures	Psychiatric Services
İpçi, K. : Yildiz, M. : İncedere, A. : Kiras, F. : Esen, D. : Gürcan, M. B.	2020	Subjective Recovery in Patients with Schizophrenia and Related Factors	Community mental health journal
Irmayati, Noverita : Yona, Sri : Waluyo, Agung	2019	HIV-related stigma, knowledge about HIV, HIV risk behavior and HIV testing motivation among women in Lampung, Indonesia	Enfermería Clínica
Ivezi, S. Ŝ : Sesar, M. A. : Muẑini, L.	2017	Effects of a group psychoeducation program on self-stigma, empowerment and perceived discrimination of persons with schizophrenia	Psychiatria Danubina
Jaapar, S. Z. S. : Rasdi, N. : Othman, Z. : Bakar, R. S. : Hussin, S. : Mohammad, J. A. M.	2020	The effect of psychosocial rehabilitation on perceived stigma among patients with schizophrenia in Kelantan, Malaysia	International Medical Journal
Jaber, A. A. S. : Khan, A. H. : Sulaiman, S. A. S. : Ahmad, N. : Anaam, M. S.	2016	Evaluation of health-related quality of life among tuberculosis patients in two cities in Yemen	PLoS ONE
Jacobs, W. : Das, E. : Schagen, S. B.	2017	Increased cognitive problem reporting after information about chemotherapy-induced cognitive decline: The moderating role of stigma consciousness	Psychology & Health
Jacobs, W. : Schagen, S. B. : Thijssen, M. : Das, E.	2019	Preventing adverse information effects on health outcomes: A self-affirmation intervention reduced information-induced cognitive decline in gastrointestinal cancer patients	Social Science and Medicine
Jacoby, Ann	1994	Felt versus enacted stigma: A concept revisited: Evidence from a study of people with epilepsy in remission	Social Science & Medicine
James, B. O. : Omoaregba, J. O.	2011	Prevalence and predictors of poor medication adherence among out-patients at a psychiatric hospital in Benin City, Nigeria	International Journal of Psychiatry in Clinical Practice
James, P. B. : Wardle, J. : Steel, A. : Adams, J.	2020	An assessment of Ebola-related stigma and its association with informal healthcare utilisation among Ebola survivors in Sierra Leone: A cross-sectional study	BMC Public Health
James, T. : Kutty, V. R. : Boyd, J. : Brzoska, P.	2016	Validation of the Malayalam version of the Internalized Stigma of Mental Illness (ISMI) scale	Asian Journal of Psychiatry
Jang, Nara : Bakken, Suzanne	2017	Relationships Between Demographic, Clinical, and Health Care Provider Social Support Factors and Internalized Stigma in People Living With HIV	JANAC: Journal of the Association of Nurses in AIDS Care
Jang, Y. : Ahn, S. H. : Lee, K. : Lee, J. : Kim, J. H.	2019	Psychometric evaluation of the Korean version of the Hepatitis B Quality of Life Questionnaire	PLoS ONE
Jankowiak, B. : Kowalewska, B. : Krajewska-Kulak, E. : Khvorik, D. F. : Niczyporuk, W.	2020	Relationship between self-esteem and stigmatization in psoriasis patients	Postepy Dermatologii i Alergologii
Jankowiak, Barbara : Kowalewska, Beata : Krajewska-Kulak, Elzbieta : Kowalczuk, Krystyna : Khvorik, Dzmitry F.	2021	The Sense of Stigmatization in Patients with Plaque Psoriasis	Dermatology (Basel, Switzerland)
Jarolimova, Jana : Yan, Joyce : Govere, Sabina : Ngobese, Nompumelelo : Shazi, Zinhle M. : Khumalo, Anele R. : Bunda, Bridget A. : Wara, Nafisa J. : Zionts, Danielle : Thulare, Hilary : Parker, Robert A. : Bogart, Laura M. : Bassett, Ingrid V.	2021	Medical Mistrust and Stigma Associated with COVID-19 Among People Living with HIV in South Africa	AIDS & Behavior
Jaworsky, D. : Logie, C. H. : Wagner, A. C. : Child, T. C. : Kaida, A. : de Pokomandy, A. : Webster, K. : Proulx-Boucher, K. : Sereda, P. : Loutfy, M.	2018	Geographic differences in the experiences of HIV-related stigma for women living with HIV in northern and rural communities of Ontario, Canada	Rural Remote Health
Jeong, J. S. : Kim, S. Y. : Kim, J. N.	2020	Ashamed Caregivers: Self-Stigma, Information, and Coping among Dementia Patient Families	Journal of health communication
Jhon, M. : Stewart, R. : Kim, J. W. : Kang, H. J. : Lee, J. Y. : Kim, S. W. : Shin, I. S. : Kim, J. M.	2021	Predictors and outcomes of experienced and anticipated discrimination in patients treated for depression: A 2-year longitudinal study	Journal of Affective Disorders
Jian, C. R. : Wang, P. W. : Lin, H. C. : Huang, M. F. : Yeh, Y. C. : Liu, T. L. : Chen, C. S. : Lin, Y. P. : Lee, S. Y. : Chen, C. H. : Wang, Y. C. : Chang, Y. P. : Chen, Y. L. : Yen, C. F.	2022	Association between Self-Stigma and Suicide Risk in Individuals with Schizophrenia: Moderating Effects of Self-Esteem and Perceived Support from Friends	Int. J. Environ. Res. Public Health
Jiang, N. : Zhang, Y. X. : Zhao, J. : Shi, H. Y. : Wang, T. : Jin, W. : Wang, J. W. : Yu, J. M.	2022	The mediator role of stigma in the association of mindfulness and social engagement among breast cancer survivors in China	Supportive Care in Cancer
Jin, J. H. : Lee, E. J.	2021	Structural Equation Model of the Quality of Working Life among Cancer Survivors Returning to Work	Asian Nursing Research
Jin, R. : Xie, T. : Zhang, L. : Gong, N. : Zhang, J.	2021	Stigma and its influencing factors among breast cancer survivors in China: A cross-sectional study	European journal of oncology nursing : the official journal of European Oncology Nursing Society
Jin, Yanfei : Ma, Hongmei : Jiménez-Herrera, Maria	2020	Self-disgust and stigma both mediate the relationship between stoma acceptance and stoma care self-efficacy	Journal of Advanced Nursing (John Wiley & Sons, Inc.)
Jin, Y. : Zheng, M. C. : Yang, X. : Chen, T. L. : Zhang, J. E.	2022	Patient delay to diagnosis and its predictors among colorectal cancer patients: A cross-sectional study based on the Theory of Planned Behavior	European journal of oncology nursing : the official journal of European Oncology Nursing Society
Jittimanee, S. X. : Nateniyom, S. : Kittikraisak, W. : Burapat, C. : Akksilp, S. : Chumpathat, N. : Sirinak, C. : Sattayawuthipong, W. : Varma, J. K.	2009	Social stigma and knowledge of tuberculosis and HIV among patients with both diseases in Thailand	PLoS ONE
Jiwanmall, Stephen A. : Kattula, Dheeraj : Nandyal, Munaf B. : Parvathareddy, Sandhiya : Kirubakaran, Richard : Jebasingh, Felix : Paul, Thomas V. : Thomas, Nihal : Kapoor, Nitin	2022	Weight Stigma in Patients With Obesity and Its Clinical Correlates: A Perspective From an Indian Bariatric Clinic	Cureus
Johnson, C. G. B. : Brodsky, J. L. : Cataldo, J. K.	2014	Lung cancer stigma, anxiety, depression, and quality of life	Journal of Psychosocial Oncology
Johnson, Eboneé T. : Yaghmaian, Rana A. : Best, Andrew : Chan, Fong : Burrell Jr, Reginald	2016	Evaluating the Measurement Structure of the Abbreviated HIV Stigma Scale in a Sample of African Americans Living With HIV/AIDS	Rehabilitation Research, Policy & Education
Johnson, Lee Ann : Schreier, Ann M. : Swanson, Melvin : Moye, Janet P. : Ridner, Sheila	2019	Stigma and Quality of Life in Patients With Advanced Lung Cancer	Oncology nursing forum
Johnson, M. O. : Neilands, T. B. : Koester, K. A. : Wood, T. : Sauceda, J. A. : Dilworth, S. E. : Mugavero, M. J. : Crane, H. M. : Fredericksen, R. J. : Mayer, K. H. : Mathews, W. C. : Moore, R. D. : Napravnik, S. : Christopoulos, K. A.	2019	Detecting Disengagement from HIV Care before It Is Too Late: Development and Preliminary Validation of a Novel Index of Engagement in HIV Care	Journal of Acquired Immune Deficiency Syndromes
Johnson, M. O. : Sevelius, J. M. : Dilworth, S. E. : Saberi, P. : Neilands, T. B.	2012	Preliminary support for the construct of health care empowerment in the context of treatment for human immunodeficiency virus	Patient Preference and Adherence
Johnson, M. : Samarina, A. : Xi, H. : Valdez Ramalho Madruga, J. : Hocqueloux, L. : Loutfy, M. : Fournelle, M. J. : Norton, M. : Van Wyk, J. : Zachry, W. : Martinez, M.	2015	Barriers to access to care reported by women living with HIV across 27 countries	AIDS Care Psychol. Socio-Med. Asp. AIDS HIV
Jones, Deborah L. : Zulu, Isaac : Vamos, Szonja : Cook, Ryan : Chitalu, Ndashi : Weiss, Stephen M.	2013	Determinants of engagement in HIV treatment and care among Zambians new to antiretroviral therapy	The Journal of the Association of Nurses in AIDS Care : JANAC
Jones, H. S. : Floyd, S. : Stangl, A. : Bond, V. : Hoddinott, G. : Pliakas, T. : Bwalya, J. : Mandla, N. : Moore, A. : Donnell, D. : Bock, P. : Fidler, S. : Hayes, R. : Ayles, H. : Hargreaves, J. R. : the, Hptn Study Team	2020	Association between HIV stigma and antiretroviral therapy adherence among adults living with HIV: baseline findings from the HPTN 071 (PopART) trial in Zambia and South Africa	Trop. Med. Int. Health
Jones, M. P. : Keefer, L. : Bratten, J. : Taft, T. H. : Crowell, M. D. : Levy, R. : Palsson, O.	2009	Development and initial validation of a measure of perceived stigma in irritable bowel syndrome	Psychology, Health and Medicine
Jormfeldt, H. : Arvidsson, B. : Svensson, B. : Hansson, L.	2008	Construct validity of a health questionnaire intended to measure the subjective experience of health among patients in mental health services	Journal of Psychiatric and Mental Health Nursing
Josephson, C. B. : Patten, S. B. : Bulloch, A. : Williams, J. V. A. : Lavorato, D. : Fiest, K. M. : Secco, M. : Jette, N.	2017	The impact of seizures on epilepsy outcomes: A national, community-based survey	Epilepsia
Juliano de Souza, Caliari : Sheila Araujo, Teles : Renata Karina, Reis : Gir, Elucir	2017	Factors related to the perceived stigmatization of people living with HIV	Revista da Escola de Enfermagem da USP
Jung, J. H. : Bae, E. Y. : Ko, J. Y.	2022	Factors associated with the mental health status of isolated COVID-19 patients in Korea	Infection, Disease and Health
Kaai, S. : Bullock, S. : Sarna, A. : Chersich, M. : Luchters, S. : Geibel, S. : Munyao, P. : Mandaliya, K. : Temmerman, M. : Rutenberg, N.	2010	Perceived stigma among patients receiving antiretroviral treatment: A prospective randomised trial comparing an m-DOT strategy with standard-of-care in Kenya	Sahara J
Kacar, Seval Dogruk : Soyucok, Ethem : Bagcioglu, Erman : Ozuguz, Pinar : Coskun, Kerem Senol : Asik, Ahmet Hakki : Mayda, Hasan	2016	The Perceived Stigma in Patients with Alopecia and Mental Disorder: A Comparative Study	International journal of trichology
Kagiura, F. : Fujii, T. : Kihana, N. : Maruyama, E. : Shimoji, Y. : Kakehashi, M.	2020	Brief HIV stigma scale for Japanese people living with HIV: validation and restructuring using questionnaire survey data	AIDS Care - Psychological and Socio-Medical Aspects of AIDS/HIV
Kahana, Boaz : Kahana, Eva : Deimling, Gary : Sterns, Samantha : Vangunten, Madeline	2011	Determinants of altered life perspectives among older-adult long-term cancer survivors	Cancer Nursing
Kahat, K.	2020	A study of relationship of social functioning and internalized stigma with insight in patients of schizophrenia and bipolar affective disorder	Indian Journal of Psychiatry
Kahn, Phoebe V. : Wishart, Heather A. : Randolph, Jennifer S. : Santulli, Robert B.	2016	Caregiver Stigma and Burden in Memory Disorders: An Evaluation of the Effects of Caregiver Type and Gender	Current gerontology and geriatrics research

Kaida, A. : Carter, A. : De Pokomandy, A. : Patterson, S. : Proulx-Boucher, K. : Nohpal, A. : Sereda, P. : Colley, G. : O'Brien, N. : Thomas-Pavanel, J. : Beaver, K. : Nicholson, V. J. : Tharao, W. : Fernet, M. : Otis, J. : Hogg, R. S. : Loutfy, M.	2015	Sexual inactivity and sexual satisfaction among women living with HIV in Canada in the context of growing social, legal and public health surveillance	Journal of the International AIDS Society
Kalantari, S. : Karbakhsh, M. : Kamiab, Z. : Kalantari, Z. : Sahraian, M. A.	2018	Perceived social stigma in patients with multiple sclerosis: A study from Iran	Acta Neurologica Taiwanica
Kalichman, Seth : Banas, Ellen : Kalichman, Moira : Mathews, Catherine	2020	Stigmatisation of alcohol use among people receiving antiretroviral therapy for HIV infection, Cape Town, South Africa	Global Public Health
Kalichman, Seth C. : Katner, Harold : El-Krab, Renee : Hill, Marnie : Ewing, Wendy : Kalichman, Moira O.	2021	HIV stigma experiences and alcohol use among patients receiving medical care in the rural south	Journal of Rural Mental Health
Kalichman, Seth C. : Mathews, Catherine : Banas, Ellen : Kalichman, Moira O.	2019	Treatment adherence in HIV stigmatized environments in South Africa: stigma avoidance and medication management	International Journal of STD & AIDS
Kalichman, S. C. : Simbayi, L. C. : Cloete, A. : Mthembu, P. P. : Mkhonta, R. N. : Ginindza, T.	2009	Measuring AIDS stigmas in people living with HIV/AIDS: The Internalized AIDS-Related Stigma Scale	AIDS Care - Psychological and Socio-Medical Aspects of AIDS/HIV
Kalichman, S. C. : Shkembi, B. : Eaton, L. A.	2022	Finding the Right Angle: A Geometric Approach to Measuring Intersectional HIV Stigma	AIDS Behav.
Kalichman, Seth C. : Shkembi, Bruno : Eaton, Lisa A.	2022	A novel psychometric approach to assessing intersectional hiv stigma: the geometric intersectional stigma scales	Journal of Behavioral Medicine
Kalichman, S. : Shkembi, B. : Hernandez, D. : Katner, H. : Thorson, K. R.	2019	Income Inequality, HIV Stigma, and Preventing HIV Disease Progression in Rural Communities	Prevention science : the official journal of the Society for Prevention Research
Kalisova, L. : Michalec, J. : Hadjipapanicolaou, D. : Raboch, J.	2018	Factors influencing the level of self-stigmatisation in people with mental illness	The International journal of social psychiatry
Kalomo, Eveline Ndinelao : Liao, Minli	2018	Burden of Care among Caregivers of Persons Living with HIV/AIDS in Rural Namibia: Correlates and Outcomes	Social Work in Public Health
Kamis, Gulsum Zuhal : Erden Aki, Sahinde Ozlem : Yildiz, Mevhibe Irem : Dogan Varan, Hacer : Dolgun, Anil Barak	2019	[The Validity and the Reliability of Turkish Version of the Self-Stigma of Depression Scale]	Depresyonda Kendini Damgalama Olcegi: Turkce Formu, Gecerlilik ve Guvenilirlik Calismasi.
Kamitani, Emiko : Chen, Jyu-Lin : Portillo, Carmen : Tokumoto, Jason : Dawson-Rose, Carol	2018	Shortened and Culturally Appropriate HIV Stigma Scale for Asians Living with HIV in the United States: Psychometric Analysis	JANAC: Journal of the Association of Nurses in AIDS Care
Kamitani, Emiko : Fukuoka, Yoshimi : Dawson-Rose, Carol	2015	Knowledge, Self-efficacy, and Self-perceived Risk for Cardiovascular Disease among Asians Living With HIV: The Influence of HIV Stigma and Acculturation	JANAC: Journal of the Association of Nurses in AIDS Care
Kamkuemah, M. : Gausi, B. : Oni, T. : Middelkoop, K.	2022	Multilevel correlates of abdominal obesity in adolescents and youth living with HIV in peri-urban Cape Town, South Africa	medRxiv
Kanemura, H. : Sano, F. : Sugita, K. : Aihara, M.	2014	Presence of monthly seizures affects perceived stigma in children with epilepsy	Journal of Huntington's Disease
Kanemura, H. : Sano, F. : Ohyama, T. : Sugita, K. : Aihara, M.	2015	Correlation between perceived stigma and EEG paroxysmal abnormality in childhood epilepsy	Epilepsy and Behavior
Kanemura, H. : Sano, F. : Ohyama, T. : Sugita, K. : Aihara, M.	2016	Seizure severity in children with epilepsy is associated with their parents' perception of stigma	Epilepsy and Behavior
Kang, EunKyo : Lee, Sun Young : Kim, Min Sun : Jung, Hyemin : Kim, Kyae Hyung : Kim, Kyoung Nam : Park, Hye Yoon : Lee, Yu Jin : Cho, Belong : Sohn, Jee Hoon	2021	The Psychological Burden of COVID-19 Stigma: Evaluation of the Mental Health of Isolated Mild Condition COVID-19 Patients	Journal of Korean medical science
Kang, E. : Rapkin, B. D. : DeAlmeida, C.	2006	Are psychological consequences of stigma enduring or transitory? A longitudinal study of HIV stigma and distress among Asians and Pacific Islanders living with HIV illness	AIDS Patient Care and STDs
Kang, N. E. : Kim, H. Y. : Kim, J. Y. : Kim, S. R.	2020	Relationship between cancer stigma, social support, coping strategies and psychosocial adjustment among breast cancer survivors	Journal of clinical nursing
Kanu, C. T. : Maduka, O. : Okeafor, C. U.	2017	Perceived stigma and highly active antiretroviral treatment adherence among persons living with HIV/AIDS in the University of Port Harcourt Teaching Hospital	Orient Journal of Medicine
Kanwal, F. : Spiegel, B. M. R. : Hays, R. D. : Durazo, F. : Han, S. B. : Saab, S. : Bolus, R. : Kim, S. J. : Gralnek, I. M.	2008	Prospective validation of the short form liver disease quality of life instrument	Alimentary Pharmacology and Therapeutics
Kao, Y. C. : Lien, Y. J. : Chang, H. A. : Wang, S. C. : Tzeng, N. S. : Loh, C. H.	2016	Evidence for the indirect effects of perceived public stigma on psychosocial outcomes: The mediating role of self-stigma	Psychiatry Research
Kaptein, A. A.	1982	Psychological correlates of length of hospitalization and rehospitalization in patients with acute, severe asthma	Soc. Sci. Med.
Kara, G. C. : Yalcin, B. M.	2022	Comparison of In-Person vs. Video Directly Observed Therapy (VDOT) on Stigma Levels in Tuberculosis Patients	Journal of the American Board of Family Medicine : JABFM
Karaçar, Y. : Bademli, K.	2022	Relationship between perceived social support and self stigma in caregivers of patients with schizophrenia	The International journal of social psychiatry
Karakaş, N. : Sarıtaş, S. Ç : Aktura, S. Ç : Karabulutlu, E. Y. : Oruç, F. G.	2022	Investigation of factors associated with stigma and social support in patients with epilepsy in Turkey: A cross-sectional study	Epilepsy and Behavior
Karakaş, S. A. : Okanlı, A. : Yılmaz, E.	2016	The Effect of Internalized Stigma on the Self Esteem in Patients with Schizophrenia	Archives of psychiatric nursing
Karbakhsh, M. : Hedayat, K. : Goodarzi, A. : Ghiasi, M. : Ghandi, N.	2021	Social participation in vitiligo patients and its association with quality of life	Iranian Journal of Dermatology
Karidi, M. V. : Stefanis, C. N. : Theleritis, C. : Tzedaki, M. : Rabavilas, A. D. : Stefanis, N. C.	2010	Perceived social stigma, self-concept, and self-stigmatization of patient with schizophrenia	Comprehensive Psychiatry
Karidi, M. V. : Vasilopoulou, D. : Savvidou, E. : Vitoratou, S. : Rabavilas, A. D. : Stefanis, C. N.	2014	Aspects of perceived stigma: The Stigma Inventory for Mental Illness, its development, latent structure and psychometric properties	Comprehensive Psychiatry
Karidi, M. V. : Vassilopoulou, D. : Savvidou, E. : Vitoratou, S. : Maillis, A. : Rabavilas, A. : Stefanis, C. N.	2015	Bipolar disorder and self-stigma: A comparison with schizophrenia	Journal of Affective Disorders
Karim, F. : Chowdhury, A. M. R. : Islam, A. : Weiss, M. G.	2007	Stigma, gender, and their impact on patients with tuberculosis in rural Bangladesh	Anthropology and Medicine
Karşidağ, S. : Çinar, N. : Şahin, Ş : Kotevoğlu, N. : Ateş, M. F.	2019	Validation and reliability study of the turkish version of the neuroquality of life (Neuro-qol)-stigma scale for neurological disorders	Turkish Journal of Medical Sciences
Kaşli, S. : Al, O. : Bademli, K.	2021	Internalized stigmatization and subjective recovery in individuals with chronic mental illness	The International journal of social psychiatry
Kato, A. : Fujimaki, Y. : Fujimori, S. : Isogawa, A. : Onishi, Y. : Suzuki, R. : Yamauchi, T. : Ueki, K. : Kadowaki, T. : Hashimoto, H.	2016	Association between self-stigma and self-care behaviors in patients with type 2 diabetes: A cross-sectional study	BMJ Open Diabetes Research and Care
Kato, A. : Fujimaki, Y. : Fujimori, S. : Isogawa, A. : Onishi, Y. : Suzuki, R. : Yamauchi, T. : Ueki, K. : Kadowaki, T. : Hashimoto, H.	2017	Psychological and behavioural patterns of stigma among patients with type 2 diabetes: A cross-sectional study	BMJ Open
Kato, A. : Fujimaki, Y. : Fujimori, S. : Isogawa, A. : Onishi, Y. : Suzuki, R. : Ueki, K. : Yamauchi, T. : Kadowaki, T. : Hashimoto, H.	2020	How self-stigma affects patient activation in persons with type 2 diabetes: A cross-sectional study	BMJ Open
Kato, A. : Fujimaki, Y. : Fujimori, S. : Isogawa, A. : Onishi, Y. : Suzuki, R. : Ueki, K. : Yamauchi, T. : Kadowaki, T. : Hashimoto, H.	2021	Associations between diabetes duration and self-stigma development in Japanese people with type 2 diabetes: A secondary analysis of cross-sectional data	BMJ Open
Kato, A. : Takada, M. : Hashimoto, H.	2014	Reliability and validity of the Japanese version of the Self-Stigma Scale in patients with type 2 diabetes	Health and Quality of Life Outcomes
Katz, S. : Nevid, J. S.	2005	Risk factors associated with posttraumatic stress disorder symptomatology in HIV-infected women	AIDS Patient Care & STDs
Katz-Saltzman, S. : Biegel, D. E. : Townsend, A.	2008	The impact of caregiver-care recipient relationship quality on family caregivers of women with substance-use disorders or co-occurring substance and mental disorders	Journal of Family Social Work
Kaushik, A. : Papachristou, E. : Dima, D. : Fewings, S. : Kostaki, E. : Ploubidis, G. B. : Kyriakopoulos, M.	2017	Measuring stigma in children receiving mental health treatment: Validation of the Paediatric Self-Stigmatization Scale (PaedS)	European Psychiatry
Kaushik, A. : Papachristou, E. : Telesia, L. : Dima, D. : Fewings, S. : Kostaki, E. : Gaete, J. : Ploubidis, G. B. : Kyriakopoulos, M.	2021	Experience of stigmatization in children receiving inpatient and outpatient mental health treatment: a longitudinal study	European Child and Adolescent Psychiatry
Kay, E. S. : Rice, W. S. : Crockett, K. B. : Atkins, G. C. : Scott Batey, D. : Turan, B.	2018	Experienced HIV-Related Stigma in Health Care and Community Settings: Mediated Associations With Psychosocial and Health Outcomes	Journal of Acquired Immune Deficiency Syndromes
Kazgan Kiliçaslan, A. : Yildiz, S. : Sirlier Emir, B. : Kurt, O.	2022	INTERNALIZED STIGMA, PERCEIVED SOCIAL SUPPORT, AND LIFE QUALITY IN PATIENTS ADMITTED TO A FORENSIC PSYCHIATRY UNIT	ITFD.
Kekwaletswe, C. T. : Morojele, N. K.	2014	Patterns and predictors of antiretroviral therapy use among alcohol drinkers at HIV clinics in Tshwane, South Africa	AIDS Care - Psychological and Socio-Medical Aspects of AIDS/HIV
Kemp, C. G. : Lipira, L. : Huh, D. : Nevin, P. E. : Turan, J. M. : Simoni, J. M. : Cohn, S. E. : Bahk, M. : Berzins, B. : Andrasik, M. : Mugavero, M. J. : Rao, D.	2019	HIV stigma and viral load among African-American women receiving treatment for HIV	AIDS
Kendra, M. S. : Mohr, J. J. : Pollard, J. W.	2014	The stigma of having psychological problems: Relations with engagement, working alliance, and depression in psychotherapy	Psychotherapy

Kent, G. : Al-Abadie, M.	1996	Factors affecting responses on Dermatology Life Quality Index items among vitiligo sufferers	Clinical and Experimental Dermatology
Kerrigan, Deanna : Vazzano, Andrea : Bertoni, Neilane : Malta, Monica : Bastos, Francisco Inacio	2017	Stigma, discrimination and HIV outcomes among people living with HIV in Rio de Janeiro, Brazil: The intersection of multiple social inequalities	Global Public Health
Kesande, C. : Bapolisi, A. : Kaggwa, M. M. : Nakimuli-Mpungu, E. : Maling, S. : Ashaba, S.	2022	Prevalence and factors associated with psychological distress among pregnant and non-pregnant youth living with HIV in rural Uganda: a comparative study	Psychology, health & medicine
Keshavarzpir, Z. : Seyedfatemi, N. : Mardani-Hamooleh, M. : Esmaeeli, N. : Boyd, J. E.	2021	The Effect of Psychoeducation on Internalized Stigma of the Hospitalized Patients with Bipolar Disorder: A Quasi-Experimental Study	Issues in mental health nursing
Khan, F. U. : Khan, F. U. : Hayat, K. : Chang, J. : Kamran, M. : Khan, A. : Malik, U. R. : Khan, A. : Fang, Y.	2021	Impact of protracted displacement on delay in the diagnosis associated with treatment outcomes: A cross-sectional study in internally displaced tuberculosis patients of Pakistan	International Journal of Environmental Research and Public Health
Khan, N. : Kausar, R. : Khalid, A. : Farooq, A.	2015	Gender differences among discrimination & stigma experienced by depressive patients in pakistan	Pakistan Journal of Medical Sciences
Khudhur, I. A. G. : Mehabes, F. J.	2012	Impact of epilepsy on patient's physical and psychosocial functioning: Iraqi study	Health Sci. J.
Khuong, L. Q. : Vu, T. V. T. : Huynh, V. A. N. : Thai, T. T.	2018	Psychometric properties of the medical outcomes study: social support survey among methadone maintenance patients in Ho Chi Minh City, Vietnam: a validation study	Substance abuse treatment, prevention, and policy
Kibria, M. G. : Islam, T. : Islam, M. T. : Kabir, R. : Ahmed, S. : Sultana, P.	2022	Stigma and its associated factors among patients with COVID-19 in Dhaka City: evidence from a cross-sectional investigation	PeerJ
Kilian, R. : Müller-Stierlin, A. : Söhner, F. : Beschoner, P. : Gündel, H. : Staiger, T. : Stiawa, M. : Becker, T. : Frasch, K. : Panzirsch, M. : Schmauß, M. : Krumm, S.	2020	Masculinity norms and occupational role orientations in men treated for depression	PLoS ONE
Kim, G. O. : Yoo, T. Y. : Kim, N. J. : Lee, H. J. : Jhon, M. : Kim, J. W. : Kang, H. J. : Kim, S. W. : Kim, J. M.	2020	Standardization of the discrimination and stigma scale-Korean version (Disc 12-k) in patients with depressive disorders	Psychiatry Investigation
Kim, S. Y. : Kim, J. M. : Kim, S. W. : Kang, H. J. : Lee, J. Y. : Bae, K. Y. : Shin, I. S. : Yoon, J. S.	2017	Perceived stigma and quality of life in patients following recovery from delirium	Journal of Clinical Psychiatry
Kim, W. J. : Song, Y. J. : Ryu, H. S. : Ryu, V. : Kim, J. M. : Ha, R. Y. : Lee, S. J. : Namkoong, K. : Ha, K. : Cho, H. S.	2015	Internalized stigma and its psychosocial correlates in Korean patients with serious mental illness	Psychiatry Research
King-Kallimanis, Bellinda L. : Oort, Frans J. : Lynn, Nancy : Schonfeld, Lawrence	2012	Testing the Assumption of Measurement Invariance in the SAMHSA Mental Health and Alcohol Abuse Stigma Assessment in Older Adults	Ageing international
Kingori, C. : Reece, M. : Obeng, S. : Murray, M. : Shacham, E. : Dodge, B. : Akach, E. : Ngatia, P. : Ojakaa, D.	2012	Impact of internalized stigma on HIV prevention behaviors among HIV-infected individuals seeking HIV care in Kenya	AIDS Patient Care and STDs
Kingori, Caroline : Reece, Michael : Obeng, Samuel : Murray, Maresa : Shacham, Enbal : Dodge, Brian : Akach, Emannuel : Ngatia, Peter : Ojakaa, David	2013	Psychometric Evaluation of a Cross-Culturally Adapted Felt Stigma Questionnaire Among People Living with HIV in Kenya	AIDS Patient Care & STDs
Kinson, R. M. : Hon, C. : Lee, H. : Abdin, E. B. : Verma, S.	2018	Stigma and discrimination in individuals with first episode psychosis; one year after first contact with psychiatric services	Psychiatry Research
Kinuthia, J. : Kohler, P. : Okanda, J. : Otieno, G. : Odhiambo, F. : John-Stewart, G.	2015	A community-based assessment of correlates of facility delivery among HIV-infected women in western Kenya	BMC Pregnancy Childbirth
Kipp, A. M. : Audet, C. M. : Earnshaw, V. A. : Owens, J. : McGowan, C. C. : Wallston, K. A.	2015	Re-validation of the Van Rie HIV/AIDS-related stigma scale for use with people living with HIV in the United States	PLoS ONE
Kipp, A. M. : Pungrassami, P. : Nilmanat, K. : Sengupta, S. : Poole, C. : Strauss, R. P. : Chongsuvivatwong, V. : Van Rie, A.	2011	Socio-demographic and AIDS-related factors associated with tuberculosis stigma in southern Thailand: a quantitative, cross-sectional study of stigma among patients with TB and healthy community members	BMC public health
Kipp, A. M. : Pungrassami, P. : Stewart, P. W. : Chongsuvivatwong, V. : Strauss, R. P. : Van Rie, A.	2011	Study of tuberculosis and AIDS stigma as barriers to tuberculosis treatment adherence using validated stigma scales	International Journal of Tuberculosis and Lung Disease
Kira, I. A. : Lewandowski, L. : Ashby, J. S. : Templin, T. : Ramaswamy, V. : Mohanesh, J.	2014	The Traumatogenic Dynamics of Internalized Stigma of Mental Illness Among Arab American, Muslim, and Refugee Clients	Journal of the American Psychiatric Nurses Association
Kirabira, J. : Ashaba, S. : Favina, A. : Maling, S. : Nansera, D. : Zanoni, B. C.	2022	Intrapersonal predictors of internalized stigma among school going adolescents living with HIV in southwestern Uganda	medRxiv
Kisala, P. A. : Tulsky, D. S. : Pace, N. : Victorson, D. : Choi, S. W. : Heinemann, A. W.	2015	Measuring stigma after spinal cord injury: Development and psychometric characteristics of the SCI-QOL Stigma item bank and short form	Journal of Spinal Cord Medicine
Kissane, D. W. : Patel, S. G. : Baser, R. E. : Bell, R. : Farberov, M. : Ostroff, J. S. : Li, Y. : Singh, B. : Kraus, D. H. : Shah, J. P.	2013	Preliminary evaluation of the reliability and validity of the Shame and Stigma Scale in head and neck cancer	Head and Neck
Kılıç, A. : Görmez, A. : Yeni Elbay, R. : Özer, B. U.	2022	Internalized stigma in obsessive compulsive disorder: Correlates and associations with quality of life	Archives of psychiatric nursing
Kılınçel, O. : Ay, R.	2021	Internalized stigma in physicians with mental illness	Klin. Psikiyatr. Derg.
Kleim, B. : Vauth, R. : Adam, G. : Stieglitz, R. D. : Hayward, P. : Corrigan, P.	2008	Perceived stigma predicts low self-efficacy and poor coping in schizophrenia	Journal of Mental Health
Kleinman, Nora J. : Manhart, Lisa E. : Mohanraj, Rani : Kumar, Shuba : Jeyaseelan, Lakshmanan : Rao, Deepa : Simoni, Jane M.	2015	Antiretroviral therapy adherence measurement in non-clinical settings in South India	AIDS Care
Kleinstäuber, M. : Wolf, L. : Jones, A. S. K. : Dalbeth, N. : Petrie, K. J.	2020	Internalized and Anticipated Stigmatization in Patients With Gout	ACR Open Rheumatology
Klingelhoefer, L. : Kaiser, M. : Sauerbier, A. : Untucht, R. : Wienecke, M. : Mammadova, K. : Falkenburger, B. : Gregor, O. : Chaudhuri, K. R. : Reichmann, H.	2021	Emotional well-being and pain could be a greater determinant of quality of life compared to motor severity in cervical dystonia	Journal of Neural Transmission
Knettel, B. A. : Wanda, L. : Amiri, I. : Myers, J. : Fernandez, K. M. : Muiruri, C. : Watt, M. H. : Mmbaga, B. T. : Relf, M. V.	2021	Assessing the Influence of Community Health Worker Support on Early Antiretroviral Therapy Adherence, Anticipated Stigma, and Mental Health among People Living with HIV in Tanzania	AIDS Patient Care and STDs
Koçak, M. B. : Şahin, A. R. : Güz, H. : Böke, Ö : Sarısoy, G. : Karabekiroğlu, A.	2022	The Relationship Between Suicide Attempts and Ideation with Depression, Insight, and Internalized Stigmatization in Schizophrenia	Anadolu Psikiyatri Dergisi
Kock, E. : Molteno, C. : Mfiki, N. : Kidd, M. : Ali, A. : King, M. : Strydom, A.	2012	Cross-cultural validation of a measure of felt stigma in people with intellectual disabilities	Journal of Applied Research in Intellectual Disabilities
Kolek, A. : Prasko, J. : Vanek, J. : Kantor, K. : Holubova, M. : Slepecky, M. : Nesnidal, V. : Latalova, K. : Ociskova, M. : Grambal, A.	2019	Severity of panic disorder, adverse events in childhood, dissociation, self-stigma and comorbid personality disorders: Part 1: Relationships between clinical, psychosocial and demographic factors in pharmacoresistant panic disorder patients	Neuroendocrinology Letters
Koller, M. : Kussman, J. : Lorenz, W. : Jenkins, M. : Voss, M. : Arens, E. : Richter, E. : Rothmund, M.	1996	Symptom reporting in cancer patients: The role of negative affect and experienced social stigma	CANCER
Komatsu, H. : Ono, T. : Onoguchi, G. : Tomita, H. : Kakuto, Y.	2021	Mediating effects of self-stigma and depression on the association between autistic symptoms and recovery in patients with schizophrenia-spectrum disorders: a cross-sectional study	BMC Psychiatry
Komolafe, M. A. : Sunmonu, T. A. : Afolabi, O. T. : Komolafe, E. O. : Fabusiwa, F. O. : Groce, N. : Kett, M. : Disu, J. O. : Ajiboye, J. K. : Olaniyan, S. O.	2012	The social and economic impacts of epilepsy on women in Nigeria	Epilepsy and Behavior
Kondrátová, L. : König, D. : Mladá, K. : Winkler, P.	2019	Correlates of Negative Attitudes towards Medication in People with Schizophrenia	Psychiatric Quarterly
Konradi, A.	2021	Stigma and psychological distress among pediatric participants in the FD/MAS Alliance Patient Registry	BMC Pediatrics
Konradi, A.	2022	Fibrous dysplasia patients with and without craniofacial involvement report reduced quality of life inclusive of stigma, depression, and anxiety	Chronic Illness
Konsztowicz, S. : Gelencser, C. R. : Otis, C. : Schmitz, N. : Lepage, M.	2021	Self-concept and Engagement in LiFe (SELF): A waitlist-controlled pilot study of a novel psychological intervention to target illness engulfment in enduring schizophrenia and related psychoses	Schizophr. Res.
Korkmaz, G. : Küçük, L.	2016	Internalized Stigma and Perceived Family Support in Acute Psychiatric In-Patient Units	Archives of psychiatric nursing
Kostic, Marina : Kocic, Biljana : Tiodorovic, Branislav	2016	Stigmatization and discrimination of patients with chronic hepatitis C	Vojnosanitetski pregled
Kotekoglu, D. : Parlakdag, A. : Koramaz, F. S. : Varol, G. : Aslankoc, V. : Bozkurt, S. : Memis, C. : Karakas, A. A. : Alpsoy, E.	2020	Internalized stigma in acne vulgaris and its relationship with quality of life, general health, body perception, and depression	Nigerian journal of clinical practice
Kowalewska, B. : Cybulski, M. : Jankowiak, B. : Krajewska-Kułak, E.	2020	Acceptance of Illness, Satisfaction with Life, Sense of Stigmatization, and Quality of Life among People with Psoriasis: A Cross-Sectional Study	Dermatology and Therapy
Kowalewska, B. : Jankowiak, B. : Krajewska-Kułak, E. : Milewski, R. : Sobolewski, M.	2021	Skin-Disease Specific and Generic Psychometric Measures in Patients with Psoriasis	Dermatology and Therapy
Kowalewska, B. : Jankowiak, B. : Cybulski, M. : Krajewska-Kulak, E. : Khvorik, D. F.	2021	Effect of disease severity on the quality of life and sense of stigmatization in psoriatics	Clin. Cosmet. Invest. Dermatol.
Kozlowski, A. J. : Cella, D. : Nitsch, K. P. : Heinemann, A. W.	2016	Evaluating Individual Change with the Quality of Life in Neurological Disorders (Neuro-QoL) Short Forms	Archives of Physical Medicine and Rehabilitation

Kozlowski, A. J. : Singh, R. : Victorson, D. : Miskovic, A. : Lai, J. S. : Harvey, R. L. : Cella, D. : Heinemann, A. W.	2015	Agreement Between Responses From Community-Dwelling Persons With Stroke and Their Proxies on the NIH Neurological Quality of Life (Neuro-QoL) Short Forms	Archives of Physical Medicine and Rehabilitation
Krasnoryadtseva, A. : Dalbeth, N. : Petrie, K.	2020	Does seeing personal medical images change beliefs about illness and treatment in people with gout? A randomised controlled trial	Psychology & health
Krikmann, Ü : Taba, P. : Lai, T. : Asser, T.	2008	Validation of an estonian version of the Parkinson's disease questionnaire (PDQ-39)	Health and Quality of Life Outcomes
Kroft, E. B. M. : De Jong, E. M. G. J. : Evers, A. W. M.	2009	Psychological distress in patients with morphea and eosinophilic fasciitis	Archives of Dermatology
Kronfli, Nadine : Lacombe-Duncan, Ashley : Wang, Lu : de Pokomandy, Alexandra : Kaida, Angela : Logie, Carmen H. : Conway, Tracey : Kennedy, V. Logan : Burchell, Ann N. : Tharao, Wangari : Pick, Neora : Kestler, Mary : Sereda, Paul : Loutfy, Mona	2017	Understanding the Correlates of Attrition Associated with Antiretroviral Use and Viral Suppression Among Women Living with HIV in Canada	AIDS Patient Care & STDs
Krüger, C. : Panske, A. : Schallreuter, K. U.	2014	Disease-related behavioral patterns and experiences affect quality of life in children and adolescents with vitiligo	International Journal of Dermatology
Krzyzanowski, D. : Agid, O. : Goghari, V. : Remington, G.	2021	Cognitive discrepancies, motivation and subjective well-being in people with schizophrenia	Schizophrenia Research: Cognition
Kular, A. : Perry, B. I. : Brown, L. : Gajwani, R. : Jasini, R. : Islam, Z. : Birchwood, M. : Singh, S. P.	2019	Stigma and access to care in first-episode psychosis	Early Intervention in Psychiatry
Kulesza, M. : Raguram, R. : Rao, D.	2014	Perceived mental health related stigma, gender, and depressive symptom severity in a psychiatric facility in South India	Asian J. Psychiatry
Kulesza, M. : Watkins, K. E. : Ober, A. J. : Osilla, K. C. : Ewing, B.	2017	Internalized stigma as an independent risk factor for substance use problems among primary care patients: Rationale and preliminary support	Drug and Alcohol Dependence
Kuloğlu Pazarcı, N. : Parasiz Yükselen, N. : Aydın, Ş : Ünlüsoy Acar, Z. : Necioğlu Örken, D.	2017	Validation and reliability study of the Turkish version of the stigma scale of epilepsy	Noropsikiyatr. Ars.
Kumar, M. : Ramanujam, B. : Barki, S. : Dwivedi, R. : Vibha, D. : Singh, R. K. : Tripathi, M.	2022	Impact of exercise as a complementary management strategy in people with epilepsy: a randomized controlled trial	Epilepsy & behavior
Kumar, N. : Colon-Zimmermann, K. : Fuentes-Casiano, E. : Liu, H. : Tatsuoka, C. : Cassidy, K. A. : Kahriman, M. : Chen, P. : Sajatovic, M.	2018	Clinical correlates of negative health events in a research sample with epilepsy	Epilepsy and Behavior
Kumar, N. : Unnikrishnan, B. : Thapar, R. : Mithra, P. : Kulkarni, V. : Holla, R. : Bhagawan, D. : Kumar, A.	2017	Stigmatization and Discrimination toward People Living with HIV/AIDS in a Coastal City of South India	Journal of the International Association of Providers of AIDS Care
Kumari, Pallavi : Ram, Daya : Nizamie, S. Haque : Goyal, Nishant	2009	Stigma and quality of life in individuals with epilepsy: A preliminary report	Epilepsy & Behavior
Kumari, Rajbala : Ranjan, Jay Kumar : Verma, Saroj : Asthana, Hari Shanker	2022	Hindi Adaptation and Psychometric Validation of the Affiliate Stigma Scale	Indian journal of psychological medicine
Kumari, S. : Banerjee, I. : Majhi, G. : Chaudhury, S. : Singh, A. : Verma, A.	2014	Felt stigma and self-esteem among psychiatric hospital outdoor and community camp attending patients	Med. J. Dr, D.Y. Patil. Univ.
Kuramochi, I. : Iwayama, T. : Horikawa, N. : Shimotsu, S. : Watanabe, S. : Yamanouchi, H. : Yoshimasu, H.	2021	Development and validation of the Epilepsy Self-Stigma Scale	Epilepsia Open
Kuramochi, I. : Iwayama, T. : Oga, K. : Shiganami, T. : Umemura, T. : Kobayashi, S. : Yasuda, T. : Yoshimasu, H.	2022	A study of factors influencing self-stigma in people with epilepsy: A nationwide online questionnaire survey in Japan	Epilepsia Open
Kuramochi, I. : Oga, K. : Iwayama, T. : Miyawaki, Y. : Ishihara, T. : Kobayashi, S. : Yoshimasu, H.	2020	Pilot trial of "Epi-school" group psychosocial education program for patients with epilepsy and their relatives in Japan	Epilepsy and Behavior
Kvrgic, S. : Cavelti, M. : Beck, E. M. : Rüsch, N. : Vauth, R.	2013	Therapeutic alliance in schizophrenia: The role of recovery orientation, self-stigma, and insight	Psychiatry Res.
Kwong, Kenny : Chung, Henry : Cheal, Karen : Chou, Jolene C. : Chen, Teddy	2013	Depression care management for Chinese Americans in primary care: a feasibility pilot study	Community mental health journal
Lacombe-Duncan, Ashley : Chuang, Deng-Min	2018	A Social Ecological Approach to Understanding Life Satisfaction among Socio-Economically Disadvantaged People Living with HIV/AIDS in Taiwan: Implications for Social Work Practice	British Journal of Social Work
Lacombe-Duncan, A. : Newman, P. A. : Bauer, G. R. : Logie, C. H. : Persad, Y. : Shokoohi, M. : O'Brien, N. : Kaida, A. : De Pokomandy, A. : Loutfy, M.	2019	Gender-affirming healthcare experiences and medical transition among transgender women living with HIV: A mixed-methods study	Sexual Health
Lacombe-Duncan, A. : Warren, L. : Kay, E. S. : Persad, Y. : Soor, J. : Kia, H. : Underhill, A. : Logie, C. H. : Kazemi, M. : Kaida, A. : de Pokomandy, A. : Loutfy, M.	2021	Mental health among transgender women living with HIV in Canada: findings from a national community-based research study	AIDS Care Psychol. Socio-Med. Asp. AIDS HIV
Lai, J. S. : Nowinski, C. J. : Zelko, F. : Wortman, K. : Burns, J. : Nordli, D. R. : Cella, D.	2015	Validation of the Neuro-QoL measurement system in children with epilepsy	Epilepsy and Behavior
Laird, K. T. : Smith, C. A. : Hollon, S. D. : Walker, L. S.	2020	Validation of the Health-Related Felt Stigma and Concealment Questionnaire	Journal of pediatric psychology
Lakuta, Patryk : Marcinkiewicz, Kamil : Bergler-Czop, Beata : Brzezinska-Wcislo, Ligia	2016	The relationship between psoriasis and depression: A multiple mediation model	Body image
Łakuta, P. : Marcinkiewicz, K. : Bergler-Czop, B. : Brzezinska-Wcisło, L.	2017	How does stigma affect people with psoriasis?	Postepy Dermatologii i Alergologii
Łakuta, P. : Marcinkiewicz, K. : Bergler-Czop, B. : Brzezińska-Wcisło, L. : Słomian, A.	2018	Associations between site of skin lesions and depression, social anxiety, body-related emotions and feelings of stigmatization in psoriasis patients	Postepy Dermatol. Alergol.
Łakuta, P. : Przybyła-Basista, H.	2017	Toward a better understanding of social anxiety and depression in psoriasis patients: The role of determinants, mediators, and moderators	Journal of Psychosomatic Research
Lallana, J. M. : Prefasi, D. : Casanova, B. : Diaz, L. F. : Saiz, A. : Calles, C. : Martínez-Ginés, M. L. : González-Suárez, I. : Boyero, S. : Pinel, L. R. : Sempere, A. P. : Lallana, V. M. : Querol, L. : Costa-Frossard, L. : De Castro-Trapiello, H. : Canal, N. : Maurino, J.	2021	Perception ofstigma in people with neuromyelitis optica spectrum disorders (PERSPECTIVESNMO Study)	Neurology
Lasalvia, A. : Bonetto, C. : Miglietta, E. : Giacco, D. : Nicaise, P. : Lorant, V. : Moskalewicz, J. : Welbel, M. : Bauer, M. : Pfennig, A. : Ruggeri, M. : Priebe, S.	2021	Comparing discrimination among people with schizophrenia, affective and anxiety disorders. A multilevel study in five European countries	Journal of Affective Disorders
Latalova, K. : Prasko, J. : Kamaradova, D. : Ociskova, M. : Cinculova, A. : Grambal, A. : Kubinek, R. : Mainerova, B. : Smoldasova, J. : Tichackova, A. : Sigmundova, Z.	2014	Self-stigma and suicidality in patients with neurotic spectrum disorder - A cross sectional study	Neuroendocrinology Letters
Lau, Y. W. : Picco, L. : Pang, S. : Jeyagurunathan, A. : Satghare, P. : Chong, S. A. : Subramaniam, M.	2017	Stigma resistance and its association with internalised stigma and psychosocial outcomes among psychiatric outpatients	Psychiatry Research
Law, Samuel F. : Sirotich, Frank : Sunderji, Nadiya : Simpson, Alexander : Nakhost, Arash	2021	The relationship between clinician leverage, patient experiences, and and the impact of stigma: a study in academic and community outpatient psychiatry settings	General hospital psychiatry
Lawes-Wickwar, S. : McBain, H. : Hirani, S. P. : Hurt, C. S. : Dunlop, N. : Solly, D. : Crampton, B. : Newman, S. P. : Ezra, D. G.	2021	Which factors impact on quality of life for adults with blepharospasm and hemifacial spasm?	Orbit (London)
Lawrence, J. W. : Fauerbach, J. A. : Heinberg, L. : Doctor, M.	2004	Visible vs Hidden Scars and Their Relation to Body Esteem	J. Burn Care Rehabil.
Lawrence, John W. : Rosenberg, Laura : Rimmer, Ruth B. : Thombs, Brett D. : Fauerbach, James A.	2010	Perceived Stigmatization and Social Comfort: Validating the Constructs and Their Measurement Among Pediatric Burn Survivors	Rehabilitation Psychology
Lebel, Sophie : Payne, Ada Y. M. : Mah, Kenneth : Irish, Jonathan : Rodin, Gary : Devins, Gerald M.	2016	Do stigma and its psychosocial impact differ between Asian-born Chinese immigrants and Western-born Caucasians with head and neck cancer?	Psychology, Health & Medicine
Lee, Hyunjin : Kwon, Myoungjin : Seo, Kawoun	2021	Validity and Reliability of the Korean Version of the Acceptance and Action Questionnaire-Stigma (AAQ-S-K)	Healthcare (Basel, Switzerland)
Lee, H. : Milev, R. : Paik, J. W.	2015	Comparison of stigmatizing experiences between Canadian and Korean patients with depression and bipolar disorders	Asia-Pacific Psychiatry
Lee, L. Y. : Tung, H. H. : Chen, S. C. : Fu, C. H.	2017	Perceived stigma and depression in initially diagnosed pulmonary tuberculosis patients	Journal of clinical nursing
Lee, R. S. : Kochman, A. : Sikkema, K. J.	2002	Internalized stigma among people living with HIV-AIDS	AIDS & Behavior
Lee, Sang-Ahm : Han, Su-Hyun : Cho, Yang-Je : Kim, Keun Tae : Kim, Ji-Eun : Shin, Dong Jin : Seo, Jong-Geun : Kim, Young-Soo : Ryu, Han Uk : Lee, Seo-Young : Kim, Jung Bin : Kang, Kyung-Wook : Kim, Shinhye : Kwon, Soonhak : Kim, Joonsik : Kim, Sunjun : Kim, Hyo Jeong : Eun, So-Hee : Hur, Yun Jung : Choi, Sun Ah : Yum, Mi-Sun : Park, Soyoung : Kim, Jee Hyun : Lee, Gha Hyun : Kim, Young Mi : Hwang, Kyoung Jin : Kim, Eun Young : Yeon, Gyu Min	2020	Does the new Korean term for epilepsy reduce the stigma for Korean adults with epilepsy?	Epilepsy & behavior : E&B
Lee, Sung-Jae : Li, Li : Lin, Chunqing : Tuan, Le Anh	2015	Challenges facing HIV-positive persons who use drugs and their families in Vietnam	AIDS Care
Lee, S. A.	2021	Felt stigma in seizure-free persons with epilepsy: Associated factors and its impact on health-related quality of life	Epilepsy and Behavior
Lee, S. A. : Cho, Y. J. : Ryu, H. U. : Kim, K. T. : Seo, J. G. : Kang, K. W. : Kim, J. E. : Kim, Y. S. : Kim, J. B. : Hwang, K. J. : Han, S. H. : Lee, G. H. : Shin, D. J. : Kim, J. H. : Lee, S. Y.	2021	Sex differences in seizure effects on social anxiety in persons with epilepsy	Epilepsy Behav.

Lee, S. A. : Han, S. H. : Cho, Y. J. : Kim, K. T. : Kim, J. E. : Shin, D. J. : Seo, J. G. : Kim, Y. S. : Ryu, H. U. : Lee, S. Y. : Kim, J. B. : Kang, K. W. : Kim, S. : Kwon, S. : Kim, J. : Kim, S. : Kim, H. J. : Eun, S. H. : Hur, Y. J. : Choi, S. A. : Yum, M. S. : Park, S. : Kim, J. H. : Lee, G. H. : Kim, Y. M. : Hwang, K. J. : Kim, E. Y. : Yeon, G. M.	2020	Factors associated with stigma and depressive symptoms in family members of patients with epilepsy	Epilepsy and Behavior
Lee, S. A. : Im, K. : Choi, E. J.	2022	Concealment behaviors in Korean adults with epilepsy: Their relationships to social anxiety and seizure severity independent of felt stigma	Epilepsy and Behavior
Lee, S. A. : Jeon, J. Y. : No, S. K. : Park, H. : Kim, O. J. : Kwon, J. H. : Jo, K. D.	2018	Factors contributing to anxiety and depressive symptoms in adults with new-onset epilepsy	Epilepsy and Behavior
Lee, S. A. : Kim, S. J. : Kim, H. J. : Lee, J. Y. : Kim, M. K. : Heo, K. : Kim, W. J. : Cho, Y. J. : Ji, K. W. : Park, K. I. : Kim, K. K. : Lee, E. M.	2020	Family cohesion is differently associated with felt stigma depending on enacted stigma in adults with epilepsy	Epilepsy and Behavior
Lee, S. A. : Lee, B. I. : Kim, W. J. : Lee, S. K. : Nam, H. : Hong, S. B. : Kim, J. H. : Song, H. K. : Kim, Y. I. : Sohn, Y. M. : Jung, K. Y. : Kim, J. H. : Jeong-Yeon, K. : No, S. K. : Lee, J. H. : Park, H. S. : Yi, S. D. : Park, S. P. : Kim, J. E. : Lee, J. J. : Kim, J. M. : Kim, H. W. : Park, H. M. : Han, S. J. : Kang, J. W. : Han, H. J. : Kim, H. Y. : Kim, O. J. : Lee, S. Y. : No, Y. J. : Huh, K. : Jo, J. W.	2015	Association of knowledge about epilepsy with mood and self-efficacy in Korean people with epilepsy	Epilepsy and Behavior
Lee, S. A. : Seo, J. Y. : Choi, E. J.	2022	Dual burdens of felt stigma and depressive symptoms in patients with epilepsy: Their association with social anxiety, marriage, and employment	Epilepsy and Behavior
Lei, H. : Tian, X. : Jin, Y. F. : Tang, L. : Chen, W. Q. : Jiménez-Herrera, M. F.	2021	The chain mediating role of social support and stigma in the relationship between mindfulness and psychological distress among Chinese lung cancer patients	Supportive Care in Cancer
Levin, M. E. : Petersen, J. M. : Durward, C. : Bingeman, B. : Davis, E. : Nelson, C. : Cromwell, S.	2021	A randomized controlled trial of online acceptance and commitment therapy to improve diet and physical activity among adults who are overweight/obese	Transl. Behav. Med.
Lezcano, E. : Gómez-Esteban, J. C. : Tijero, B. : Bilbao, G. : Lambarri, I. : Rodriguez, O. : Villoria, R. : Dolado, A. : Berganzo, K. : Molano, A. : de Gopegui, E. R. : Pomposo, I. : Gabilondo, I. : Zarranz, J. J.	2016	Long-term impact on quality of life of subthalamic nucleus stimulation in Parkinson's disease	Journal of Neurology
Li, G. : Ali, K. : Gao, X. : Lu, S. : Xu, W. : Zhu, X.	2022	Impact of Asymptomatic Neurosyphilis on Patients Quality of Life and Social Stigma	Psychol. Res. Behav. Manage.
Li, G. : Wang, G. : Hsu, F. C. : Xu, J. : Pei, X. : Zhao, B. : Shetty, A.	2020	Effects of depression, anxiety, stigma, and disclosure on health-related quality of life among chronic hepatitis B patients in Dalian, China	American Journal of Tropical Medicine and Hygiene
Li, Haochu : Chen, Xinguang : Yu, Bin	2016	Disclosure appraisal mediating the association between perceived stigma and HIV disclosure to casual sex partners among HIV+ MSM: a path model analysis	AIDS Care
Li, H. : Zhang, M. : Chen, L. : Zhang, J. : Pei, Z. : Hu, A. : Wang, Q.	2010	Nonmotor symptoms are independently associated with impaired health-related quality of life in Chinese patients with Parkinson's disease	Movement Disorders
Li, J. : Assanangkornchai, S. : Lu, L. : Jia, M. : McNeil, E. B. : You, J. : Chongsuvivatwong, V.	2016	Development of internalized and personal stigma among patients with and without HIV infection and occupational stigma among health care providers in Southern China	Patient Preference and Adherence
Li, J. : Huang, Y. G. : Ran, M. S. : Fan, Y. : Chen, W. : Evans-Lacko, S. : Thornicroft, G.	2018	Community-based comprehensive intervention for people with schizophrenia in Guangzhou, China: Effects on clinical symptoms, social functioning, internalized stigma and discrimination	Asian Journal of Psychiatry
Li, J. : Mo, P. K. H. : Kahler, C. W. : Lau, J. T. F. : Du, M. : Dai, Y. : Shen, H.	2016	Prevalence and associated factors of depressive and anxiety symptoms among HIV-infected men who have sex with men in China	AIDS Care - Psychological and Socio-Medical Aspects of AIDS/HIV
Li, L. : Lee, S. J. : Thammawijaya, P. : Jiraphongsa, C. : Rotheram-Borus, M. J.	2009	Stigma, social support, and depression among people living with HIV in Thailand	AIDS Care Psychol. Socio-Med. Asp. AIDS HIV
Li, Michael Jonathan : Murray, Jordan Keith : Suwanteerangkul, Jiraporn : Wiwatanadate, Phongtape	2014	Stigma, Social Support, and Treatment Adherence Among HIV-Positive Patients in Chiang Mai, Thailand	AIDS Education & Prevention
Li, M. : Yang, H. M. : Luo, D. X. : Chen, J. Z. : Shi, H. J.	2016	Multi-dimensional analysis on Parkinson's disease questionnaire-39 in Parkinson's patients treated with Bushen Huoxue Granule: A multicenter, randomized, double-blinded and placebo controlled trial	Complementary Therapies in Medicine
Li, S. : Li, Y. : Zhang, L. : Bi, Y. : Zou, Y. : Liu, L. : Zhang, H. : Yuan, Y. : Gong, W. : Zhang, Y.	2022	Impact of fear of hypoglycaemia on self-management in patients with type 2 diabetes mellitus: structural equation modelling	Acta Diabetologica
Li, X. : Huang, L. : Wang, H. : Fennie, K. P. : He, G. : Williams, A. B.	2011	Stigma mediates the relationship between self-efficacy, medication adherence, and quality of life among people living with HIV/AIDS in China	AIDS Patient Care and STDs
Li, Xianhong : Li, Ling : Wang, Honghong : Fennie, Kristopher P. : Chen, Jia : Williams, Ann Bartley	2015	Mediation analysis of health-related quality of life among people living with HIV infection in China	Nursing & Health Sciences
Li, Y. : Guo, Y. : Alicia Hong, Y. : Zeng, C. : Zeng, Y. : Zhang, H. : Zhu, M. : Qiao, J. : Cai, W. : Li, L. : Liu, C.	2021	Mediating effects of stigma and depressive symptoms in a social media-based intervention to improve long-term quality of life among people living with hiv: Secondary analysis of a randomized controlled trial	Journal of Medical Internet Research
Li, Y. : Zhang, X. : Zhang, J. : Zhang, D. : Wang, Y. : Zhu, Y. : Xu, X.	2022	Stigma and unhealthy psychological characteristics in patients with acromegaly: A cross-sectional study and identification of the associated factors	Acta Neurochirurgica
Li, Z. : Hsieh, E. : Morano, J. P. : Sheng, Y.	2016	Exploring HIV-related stigma among HIV-infected men who have sex with men in Beijing, China: a correlation study	AIDS Care - Psychological and Socio-Medical Aspects of AIDS/HIV
Liang, Z. : Huang, Y. T.	2022	Intersecting Stigma and HIV/AIDS Community Participation Among Young Chinese Men Who Have Sex with Men Living with HIV	AIDS Patient Care STDs
Licul, Roberto : Rončević-Gržeta, Ika	2019	Percipirana samostigmatizacija pacijenata hospitaliziranih u Klinici za psihijatriju KBC-a Rijeka = Perceived self-stigmatization of patients hospitalized at Psychiatry Department of Clinical Hospital Centre Rijeka	Socijalna Psihijatrija
Lien, Y. J. : Chang, H. A. : Kao, Y. C. : Tzeng, N. S. : Lu, C. W. : Loh, C. H.	2018	Insight, self-stigma and psychosocial outcomes in Schizophrenia: A structural equation modelling approach	Epidemiology and Psychiatric Sciences
Lien, Y. J. : Chang, H. A. : Kao, Y. C. : Tzeng, N. S. : Lu, C. W. : Loh, C. H.	2018	The impact of cognitive insight, self-stigma, and medication compliance on the quality of life in patients with schizophrenia	Eur. Arch. Psychiatry Clin. Neurosci.
Lien, Yin-Ju : Chang, Hsin-An : Kao, Yu-Chen : Tzeng, Nian-Sheng : Yeh, Chin-Bin : Loh, Ching-Hui : Lien, Yin-Ju : Chang, Hsin-An : Kao, Yu-Chen : Tzeng, Nian-Sheng : Yeh, Chin-Bin : Loh, Ching-Hui	2018	Self-Stigma Mediates the Impact of Insight on Current Suicide Ideation in Suicide Attempters with Schizophrenia: Results of a Moderated Mediation Approach	Suicide & Life-Threatening Behavior
Lien, Y. J. : Kao, Y. C. : Liu, Y. P. : Chang, H. A. : Tzeng, N. S. : Lu, C. W. : Loh, C. H.	2015	Internalized Stigma and Stigma Resistance Among Patients with Mental Illness in Han Chinese Population	Psychiatric Quarterly
Lifson, Alan R. : Workneh, Sale : Hailemichael, Abera : Demissie, Workneh : Slater, Lucy : Shenie, Tibebe	2015	Perceived social support among HIV patients newly enrolled in care in rural Ethiopia	AIDS care
Lifson, A. R. : Workneh, S. : Hailemichael, A. : Demisse, W. : Slater, L. : Shenie, T.	2017	Implementation of a Peer HIV Community Support Worker Program in Rural Ethiopia to Promote Retention in Care	Journal of the International Association of Providers of AIDS Care
Lightner, J. S. : Cabral, H. J. : Flaherty, J. : Silmi, K. P. : Guidry, J. : Kresiberg, A. : Brooks, R. A. : Byrne, T. H. : Rajabiun, S.	2022	Does HIV Stigma Predict Social Networks Over Time: A Latent Growth Curve Analysis	AIDS Behav.
Lightner, J. S. : Rajabiun, S. : Cabral, H. J. : Flaherty, J. : Shank, J. : Brooks, R.	2021	Associations of internalized and anticipated HIV stigma with returning to work for persons living with HIV	PLoS ONE
Lillis, J. : Luoma, J. B. : Levin, M. E. : Hayes, S. C.	2010	Measuring weight self-stigma: The weight self-stigma questionnaire	Obesity
Lim, Leslie : Goh, Justine : Chan, Yiong-Huak	2019	Internalized stigma, disclosure and self-esteem among psychiatric patients in a general hospital outpatient clinic	Australasian Psychiatry
Lim, M. : Li, Z. : Xie, H. : Tan, B. L. : Lee, J.	2019	An Asian study on clinical and psychological factors associated with personal recovery in people with psychosis	BMC Psychiatry
Lim, M. : Li, Z. : Xie, H. : Tan, B. L. : Lee, J.	2021	The Effect of Therapeutic Alliance on Attitudes Toward Psychiatric Medications in Schizophrenia	Journal of clinical psychopharmacology
Lim, M. : Xie, H. : Li, Z. : Tan, B. L. : Lee, J.	2020	Using the CHIME Personal Recovery Framework to Evaluate the Validity of the MHRM-10 in Individuals with Psychosis	Psychiatric Quarterly
Lin, B. : Zhong, G. : Liang, Z. : Huang, J. : Wang, X. : Lin, Y.	2021	Perceived-stigma level of COVID-19 patients in China in the early stage of the epidemic: A cross-sectional research	PLoS ONE
Lin, H. Y. : Hasegawa, H. : Mundil, N. : Samuel, M. : Ashkan, K.	2019	Patients' Expectations and Satisfaction in Subthalamic Nucleus Deep Brain Stimulation for Parkinson Disease: 6-Year Follow-up	World Neurosurgery
Lin, J. : Ou, R. : Wei, Q. : Cao, B. : Li, C. : Hou, Y. : Zhang, L. : Liu, K. : Shang, H.	2022	Self-Stigma in Parkinson's Disease: A 3-Year Prospective Cohort Study	Frontiers in Aging Neuroscience
Lin, M. H. : Ou, H. Y. : Wang, R. H. : Lin, C. H. : Liao, H. Y. : Chen, H. M.	2022	Glycaemic control mediates the relationships of employment status and self-stigma with self-care behaviours in young adults with type 2 diabetes	Journal of clinical nursing
Lin, Yao-Yu : Lin, Mei-Ling : Huang, Yao-Hui : Ma, Wei-Fen : Yen, Wen-Jiuan : Lee, Shih-Kai	2022	Effects of Rehabilitation Models on Self-Stigma among Persons with Mental Illness	Healthcare (Basel, Switzerland)
Lin, Y. C. : Dhaliwal, J. S. : Kong, A. Z. H. : Chan, L. G. : Tan, P. L. L.	2017	HIV-related stigma as perceived by HIV-positive individuals in Singapore	HIV and AIDS Review

Lindayani, Linlin : Ibrahim, Kusman : Wang, Jung-Der : Ko, Nai-Ying	2018	Independent and synergistic effects of self- and public stigmas on quality of life of HIV-infected persons	AIDS Care
Link, B. : Castille, D. M. : Stuber, J.	2008	Stigma and coercion in the context of outpatient treatment for people with mental illnesses	Social Science and Medicine
Lion, Katarzyna Małgorzata : Szcześniak, Dorota : Bulińska, Katarzyna : Evans, Shirley Barbara : Evans, Simon C. : Saibene, Francesca Lea : d'Arma, Alessia : Farina, Elisabetta : Brooker, Dawn June : Chattat, Rabih : Meiland, Franka J. M. : Dröes, Rose-Marie : Rymaszewska, Joanna	2020	Do people with dementia and mild cognitive impairments experience stigma? A cross-cultural investigation between Italy, Poland and the UK	Aging & Mental Health
Lion, Katarzyna Małgorzata : Szcześniak, Dorota : Bulińska, Katarzyna : Mazurek, Justyna : Evans, Shirley B. : Evans, Simon C. : Saibene, Francesca Lea : d'Arma, Alessia : Scorolli, Claudia : Farina, Elisabetta : Brooker, Dawn : Chattat, Rabih : Meiland, Franka J. M. : Dröes, Rose-Marie : Rymaszewska, Joanna	2021	Does the Meeting Centre Support Programme decrease the experience of stigmatisation among people with cognitive deficits?	Aging & Mental Health
Lipira, L. : Williams, E. C. : Huh, D. : Kemp, C. G. : Nevin, P. E. : Greene, P. : Unger, J. M. : Heagerty, P. : French, A. L. : Cohn, S. E. : Turan, J. M. : Mugavero, M. J. : Simoni, J. M. : Andrasik, M. P. : Rao, D.	2019	HIV-Related Stigma and Viral Suppression Among African-American Women: Exploring the Mediating Roles of Depression and ART Nonadherence	AIDS and behavior
Lipira, L. : Williams, E. C. : Nevin, P. E. : Kemp, C. G. : Cohn, S. E. : Turan, J. M. : Simoni, J. M. : Andrasik, M. P. : French, A. L. : Unger, J. M. : Heagerty, P. : Rao, D.	2019	Religiosity, Social Support, and Ethnic Identity: Exploring "resilience Resources" for African-American Women Experiencing HIV-Related Stigma	Journal of Acquired Immune Deficiency Syndromes
Littleton, Tenesha : Choi, Y. Joon : McGarity, Stephen V.	2020	Psychological and social correlates of HIV stigma among people living with HIV	Journal of HIV/AIDS & Social Services
Liu, H. : Xu, Y. : Sun, Y. : Dumenci, L.	2014	Measuring HIV stigma at the family level: Psychometric assessment of the Chinese Courtesy Stigma Scales (CCSSs)	PLoS ONE
Liu, Huan : Zhao, Miaomiao : Ren, Jiaojiao : Qi, Xinye : Sun, Hong : Qu, Lemeng : Yan, Cunling : Zheng, Tong : Wu, Qunhong : Cui, Yu	2018	Identifying factors associated with depression among men living with HIV/AIDS and undergoing antiretroviral therapy: a cross-sectional study in Heilongjiang, China	Health & Quality of Life Outcomes
Liu, K. : Zhang, Y. : Qu, S. : Yang, W. : Guo, L. : Zhang, L.	2021	Prevalence and Correlates of Anxiety and Depressive Symptoms in Patients With and Without Multi-Drug Resistant Pulmonary Tuberculosis in China	Frontiers in Psychiatry
Liu, Nancy F. : Brown, Adam S. : Younge, Michael F. : Guzman, Susan J. : Close, Kelly L. : Wood, Richard	2017	Stigma in People With Type 1 or Type 2 Diabetes	Clinical Diabetes
Liu, Nancy H. : Philbin, Morgan M. : Cham, Heining	2014	Depression and Stigma Among HIV-Positive Injection Drug Users in China: An Exploratory Study	Journal of HIV/AIDS & Social Services
Liu, T. : Geng, Y. : Han, Z. : Qin, W. : Zhou, L. : Ding, Y. : Zhang, Z. : Sun, G.	2022	Self-reported sleep disturbance is significantly associated with depression, anxiety, self-efficacy, and stigma in Chinese patients with rheumatoid arthritis	Psychology, health & medicine
Liu, Xiao Hang : Zhong, Jiu Di : Zhang, Jun E. : Cheng, Yu : Bu, Xiu Qing	2020	Stigma and its correlates in people living with lung cancer: A cross-sectional study from China	Psycho-Oncology
Liu, Y. : Gong, H. : Yang, G. : Yan, J.	2014	Perceived stigma, mental health and unsafe sexual behaviors of people living with HIV/AIDS	Zhong nan da xue xue bao. Yi xue ban = Journal of Central South University. Medical sciences
Livingston, J. D. : Nijdam-Jones, A. : Lapsley, S. : Calderwood, C. : Brink, J.	2013	Supporting Recovery by Improving Patient Engagement in a Forensic Mental Health Hospital: Results From a Demonstration Project	Journal of the American Psychiatric Nurses Association
Livingston, J. D. : Rossiter, K. R. : Verdun-Jones, S. N.	2011	'Forensic' labelling: An empirical assessment of its effects on self-stigma for people with severe mental illness	Psychiatry Res.
Lloyd, C. : Waghorn, G. : Best, M. : Gemmell, S.	2008	Reliability of a composite measure of social inclusion for people with psychiatric disabilities	Australian Occupational Therapy Journal
Lo, S. B. : Huber, C. G. : Meyer, A. : Weinmann, S. : Luethi, R. : Dechent, F. : Borgwardt, S. : Lieb, R. : Lang, U. E. : Moeller, J.	2022	The relationship between psychological characteristics of patients and their utilization of psychiatric inpatient treatment: A cross-sectional study, using machine learning	PLoS ONE
Loganathan, S. : Murthy, S.	2008	Experiences of stigma and discrimination endured by people suffering from schizophrenia	Indian Journal of Psychiatry
Logie, C. H. : Sokolovic, N. : Kazemi, M. : Islam, S. : Frank, P. : Gormley, R. : Kaida, A. : de Pokomandy, A. : Loutfy, M.	2022	Does resource insecurity drive HIV-related stigma? Associations between food and housing insecurity with HIV-related stigma in cohort of women living with HIV in Canada	Journal of the International AIDS Society
Logie, C. H. : Sokolovic, N. : Kazemi, M. : Smith, S. : Islam, S. : Lee, M. : Gormley, R. : Kaida, A. : de Pokomandy, A. : Loutfy, M.	2022	Recent sex work and associations with psychosocial outcomes among women living with HIV: findings from a longitudinal Canadian cohort study	Journal of the International AIDS Society
Logie, Carmen : James, Llana : Tharao, Wangari : Loutfy, Mona	2013	Associations Between HIV-Related Stigma, Racial Discrimination, Gender Discrimination, and Depression Among HIV-Positive African, Caribbean, and Black Women in Ontario, Canada	AIDS Patient Care & STDs
Lolich, M. : Vázquez, G. : Leiderman, E. A.	2010	[First psychotic episode in bipolar disorder: clinical differentiation and functional impact in an Argentinean national sample]	Vertex (Buenos Aires, Argentina)
López, A. : Rafful, C. : Orozco, R. : Contreras-Valdez, J. A. : Jiménez-Rivagorza, L. : Morales, M.	2022	HIV Stigma Mechanisms Scale: Factor Structure, Reliability, and Validity in Mexican Adults	AIDS and behavior
Lopez, Veronica : Sanchez, Katherine : Killian, Michael O. : Eghaneyan, Brittany H.	2018	Depression screening and education: an examination of mental health literacy and stigma in a sample of Hispanic women	BMC public health
Lopez-Soley, E. : Llufriu, S. : Gómez-Ballesteros, R. : Mauriño, J. : Pérez-Miralles, F. : Forero, L. : Sepúlveda, M. : Calles, C. : Gines, M. L. M. : Gonzalez, I. : Boyero, S. : Romero-Pinel, L. : Sempere, Á P. : Meca-Lallana, V. : Querol, L. : Franca, L. C. : Saiz, A. : Meca-Lallana, J. E. : Solana, E.	2022	Cognitive performance and health-related quality of life in patients with neuromyelitis optica spectrum disorder	Neurology
Lotfabadi, M. K. : Hashemi, B. M. : Sarabian, S. : Mohammadi, A. : Salarhaji, A.	2020	The effect of the components of king's spiritual intelligence group training on Stigma in patients with cancer	Nurs. Pract. Today
Low, Hu Liang : Ismail, Mohd Nasir Bin Mohd : Taqvi, Ahsan : Deeb, Jacquie : Fuller, Charlotte : Misbahuddin, Anjum	2019	Comparison of posterior subthalamic area deep brain stimulation for tremor using conventional landmarks versus directly targeting the dentatorubrothalamic tract with tractography	Clinical neurology and neurosurgery
Lu, C. : Winkelman, M. : Wong, S. S.	2016	Tablet-based education to reduce depression-related stigma	Health Education Journal
Lu, M. : Yang, Y. : Wang, G. : Wang, H. : Feng, D.	2022	Effects of Perceived Stigma on Depressive Symptoms and Demoralization in Maintenance Hemodialysis Patients: Self-warmth and Self-coldness as Mediators	Mindfulness
Lu, Q. : Deng, C. : Fu, L. : Wu, R. : Chang, L. : Qi, H. : Wang, K. : Jiang, L. : Yang, X. : Wang, Y. : Li, L. : Zhao, Y.	2019	Reliability and validity of a Chinese version of the Stigma Scale for Chronic Illness (SSCI) in patients with stroke	Topics in Stroke Rehabilitation
Lu, Q. : Wang, D. : Fu, L. : Wang, X. : Li, L. : Jiang, L. : Deng, C. : Zhao, Y.	2022	The effect of stigma on social participation in community-dwelling Chinese patients with stroke sequelae: A cross-sectional study	Clinical rehabilitation
Lu, Y. : Duller, P. : Van Der Valk, P. G. M. : Evers, A. W. M.	2003	Helplessness as predictor of perceived stigmatization in patients with psoriasis and atopic dermatitis	Dermatology and Psychosomatics
Lu, Ying : Wang, Xiaoping	2012	Correlation between insight and internalized stigma in patients with schizophrenia	Shanghai archives of psychiatry
Luciano, M. : Sampogna, G. : Del Vecchio, V. : Giallonardo, V. : Palummo, C. : Andriola, I. : Amore, M. : Rossi, R. : Carmassi, C. : Siracusano, A. : Fiorillo, A. : De Rosa, C. : Giannelli, L. : Malangone, C. : Pocai, B. : Zinno, F. : Sangiuliano, M. : Di Gioia, C. : Calcagno, P. : Serafini, G. : Pacitti, F. : Rossi, A. : Pedrinelli, V. : Cordone, A. : Di Lorenzo, G. : Niolu, C.	2021	The impact of clinical and social factors on the physical health of people with severe mental illness: Results from an Italian multicentre study	Psychiatry Research
Lufianti, A. : Mahanani, S. : Idris, D. N. T.	2022	Stigma and Self-concept of Leprosy Patients	Open Access Macedonian Journal of Medical Sciences
Lundberg, B. : Hansson, L. : Wentz, E. : Björkman, T.	2009	Are stigma experiences among persons with mental illness, [sic] related to perceptions of self-esteem, empowerment and sense of coherence?	Journal of Psychiatric & Mental Health Nursing (Wiley-Blackwell)
Luo, D. : Zhou, M. : Sun, L. : Lin, Z. : Bian, Q. : Liu, M. : Ren, S.	2021	Resilience as a Mediator of the Association Between Perceived Stigma and Quality of Life Among People With Inflammatory Bowel Disease	Frontiers in Psychiatry
Luo, R. : Ji, Y. : Liu, Y. H. : Sun, H. : Tang, S. : Li, X.	2022	Relationships among social support, coping style, self-stigma, and quality of life in patients with diabetic foot ulcer: A multicentre, cross-sectional study	International Wound Journal
Luoma, J. B. : Kulesza, M. : Hayes, S. C. : Kohlenberg, B. : Larimer, M.	2014	Stigma predicts residential treatment length for substance use disorder	Am. J. Drug Alcohol Abuse
Lutova, N. B. : Makarevich, O. V. : Vid, V. D. : Novikova, K. E. : Sorokin, M. Yu	2021	Internal stigma and narcissistic regulation of patients with endogenous psychoses	Russian Psychol. J.
Lv, Xiao-qing : Feng, Yuan : Li, Jing-ru : Zhang, Xin-qiong : Xiang, Ru : Hong, Jing-fang	2022	Validation of a Chinese version of the short-form Cataldo lung cancer stigma scale	Heart & Lung
Lv, Y. : Wolf, A. : Wang, X.	2013	Experienced stigma and self-stigma in Chinese patients with schizophrenia	General Hospital Psychiatry
Lyons, Anthony : Heywood, Wendy : Rozbroj, Tomas	2016	Psychosocial Factors Associated with Resilience in a National Community-Based Cohort of Australian Gay Men Living with HIV	AIDS & Behavior
Lysaker, P. H. : Tunze, C. : Yanos, P. T. : Roe, D. : Ringer, J. : Rand, K.	2012	Relationships between stereotyped beliefs about mental illness, discrimination experiences, and distressed mood over 1 year among persons with schizophrenia enrolled in rehabilitation	Social psychiatry and psychiatric epidemiology
MacDougall, A. G. : Kukan, S. : Price, E. : Glen, S. : Bird, R. : Powe, L. : Wiener, J. C. : Lysaker, P. H. : Anderson, K. K. : Norman, R. M. G.	2020	Participatory video as a novel recovery-oriented intervention in early psychosis: A pilot study	Int. J. Soc. Psychiatry

MacInnes, D. L. : Lewis, M.	2008	The evaluation of a short group programme to reduce self-stigma in people with serious and enduring mental health problems	Journal of Psychiatric & Mental Health Nursing (Wiley-Blackwell)
Macq, J. : Solis, A. : Martinez, G. : Martiny, P.	2008	Tackling tuberculosis patients' internalized social stigma through patient centred care: An intervention study in rural Nicaragua	BMC Public Health
Madawala, S. : Enticott, J. : Sturgiss, E. : Selamoglu, M. : Barton, C.	2022	The impact of smoking status on anticipated stigma and experience of care among smokers and ex-smokers with chronic illness in general practice	Chronic Illness
Magaña, S. M. : Ramírez García, J. I. : Hernández, M. G. : Cortez, R.	2007	Psychological distress among Latino family caregivers of adults with schizophrenia: The roles of burden and stigma	Psychiatric Services
Magidson, J. F. : Rose, A. L. : Regenauer, K. S. : Brooke-Sumner, C. : Anvari, M. S. : Jack, H. E. : Johnson, K. : Belus, J. M. : Joska, J. : Bassett, I. V. : Sibeko, G. : Myers, B.	2022	"It's all about asking from those who have walked the path": Patient and stakeholder perspectives on how peers may shift substance use stigma in HIV care in South Africa	Addiction science & clinical practice
Magidson, Jessica F. : Saal, Wylene : Nel, Adriaan : Remmert, Jocelyn E. : Kagee, Ashraf	2017	Relationship between depressive symptoms, alcohol use, and antiretroviral therapy adherence among HIV-infected, clinic-attending patients in South Africa	Journal of Health Psychology
Magnus, M. : Herwehe, J. : Murtaza-Rossini, M. : Reine, P. : Cuffie, D. : Gruber, D. : Kaiser, M.	2013	Linking and retaining HIV patients in care: The importance of provider attitudes and behaviors	AIDS Patient Care and STDs
Maguire, R. : Lewis, L. : Kotronoulas, G. : McPhelim, J. : Milroy, R. : Cataldo, J.	2019	Lung cancer stigma: A concept with consequences for patients	Cancer Reports
Maha Putra, I. Nyoman Arya : Waluyo, Agung : Yona, Sri	2019	The Correlation between Stigma and Family Acceptance with Religiosity of PLWH MSM in Medan, Indonesia	Asian Pacific Island Nursing Journal
Mahajan, Abhishek : Banerjee, Amitav	2015	Perceived stigma among attendees of psychiatric and nonpsychiatric outpatients department in an industrial township: A comparative study	Industrial psychiatry journal
Mahanani, S. : Idris, D. N. T.	2021	Correlation between disability and stigma on leprosy patient at general public hospital of daha husada kediri	Medico-Legal Update
Mahardita, N. G. P. : Susanto, T. : Siswoyo, : Wuryaningsih, E. W. : Deviantony, F.	2019	Prevalence of disability and drop out from treatment: A cross-sectional study of social stigma and motivation for healing among people affected by leprosy in district of jember, east java province, indonesia	Indian Journal of Leprosy
Maharjan, S. : Panthee, B.	2019	Prevalence of self-stigma and its association with self-esteem among psychiatric patients in a Nepalese teaching hospital: A cross-sectional study	BMC Psychiatry
Mahmoudi, H. : Saffari, M. : Movahedi, M. : Sanaeinasab, H. : Rashidi-Jahan, H. : Pourgholami, M. : Poorebrahim, A. : Barshan, J. : Ghiami, M. : Khoshmanesh, S. : Potenza, M. N. : Lin, C. Y. : Pakpour, A. H.	2021	A mediating role for mental health in associations between COVID-19-related self-stigma, PTSD, quality of life, and insomnia among patients recovered from COVID-19	Brain and Behavior
Maimaiti, Rena : Yuexin, Zhang : Kejun, Pan : Wubili, Maimaitaili : Lalanne, Christophe : Duracinsky, Martin : Andersson, Rune	2017	Assessment of Health-Related Quality of Life among People Living with HIV in Xinjiang, West China	Journal of the International Association of Providers of AIDS Care
Mak, Winnie W. S. : Cheung, Rebecca Y. M.	2012	Psychological distress and subjective burden of caregivers of people with mental illness: the role of affiliate stigma and face concern	Community mental health journal
Makanjuola, V. : Esan, Y. : Oladeji, B. : Kola, L. : Appiah-Poku, J. : Harris, B. : Othieno, C. : Price, L. : Seedat, S. : Gureje, O.	2016	Explanatory model of psychosis: impact on perception of self-stigma by patients in three sub-saharan African cities	Social psychiatry and psychiatric epidemiology
Makoae, L. N. : Portillo, C. J. : Uys, L. R. : Dlamini, P. S. : Greeff, M. : Chirwa, M. : Kohi, T. W. : Naidoo, J. : Mullan, J. : Wantland, D. : Durrheim, K. : Holzemer, W. L.	2009	The impact of taking or not taking ARVs on HIV stigma as reported by persons living with HIV infection in five African countries	AIDS Care - Psychological and Socio-Medical Aspects of AIDS/HIV
Malekmohammadi, N. : Khezri, M. : Rafiee Rad, A. A. : Iranpour, A. : Ghalekhani, N. : Shafiei Bafti, M. : Zolala, F. : Sharifi, H.	2022	Quality of Life and Its Association With HIV-Related Stigma Among People Living With HIV in Kerman, Iran: A Cross-Sectional Study	The Journal of the Association of Nurses in AIDS Care : JANAC
Malekmohammadi, N. : Mirzazadeh, A. : Iranpour, A. : Bafti, M. S. : Zolala, F. : McFarland, W. : Sharifi, H.	2021	Hiv stigma among people living with HIV in southeast Iran	Journal of Kerman University of Medical Sciences
Malik, N. I. : Fatima, R. : Ullah, I. : Atta, M. : Awan, A. : Nashwan, A. J. : Ahmed, S.	2022	Perceived stigma, discrimination and psychological problems among patients with epilepsy	Frontiers in Psychiatry
Malli, M. A. : Forrester-Jones, R.	2022	Stigma and Adults with Tourette's Syndrome: "Never Laugh at Other People's Disabilities, Unless they have Tourette's-Because How Can You Not?"	J. Dev. Phys. Disabil.
Maluccio, J. A. : Wu, F. : Rokon, R. B. : Rawat, R. : Kadiyala, S.	2017	Assessing the Impact of Food Assistance on Stigma Among People Living with HIV in Uganda Using the HIV/AIDS Stigma Instrument-PLWA (HASI-P)	AIDS Behav.
Mao, L. : Wang, K. : Zhang, Q. : Wang, J. : Zhao, Y. : Peng, W. : Ding, J.	2022	Felt Stigma and Its Underlying Contributors in Epilepsy Patients	Front. Public Health
Maragh-Bass, A. C. : Gamble, T. : El-Sadr, W. M. : Hanscom, B. : Tolley, E. E.	2021	Examining stigma, social support, and gender differences in unsuppressed HIV viral load among participants in HPTN 065	J. Behav. Med.
Marbach, J. J. : Lennon, M. C. : Link, B. G. : Dohrenwend, B. P.	1990	Losing face: Sources of stigma as perceived by chronic facial pain patients	J Behav Med
Marbaniang, I. : Borse, R. : Sangle, S. : Kinikar, A. : Chavan, A. : Nimkar, S. : Suryavanshi, N. : Mave, V.	2022	Development of shortened HIV-related stigma scales for young people living with HIV and young people affected by HIV in India	Health and Quality of Life Outcomes
Margetič, B. : Aukst-Margetič, B. : Ivanec, D. : Filipčić, I.	2008	Perception of stigmatization in forensic patients with schizophrenia	International Journal of Social Psychiatry
Margetić, B. A. : Jakovljević, M. : Ivanec, D. : Margetić, B. : Tošić, G.	2010	Relations of internalized stigma with temperament and character in patients with schizophrenia	Comprehensive Psychiatry
Margetic, B. A. : Kukulj, S. : Galic, K. : Zolj, B. S. : Jakšiü, N.	2021	Personality and stigma in lung cancer patients	Psychiatria Danubina
Margolis, S. A. : Gonzalez, J. S. : Faria, C. : Kenney, L. : Grant, A. C. : Nakhutina, L.	2019	Anxiety disorders in predominantly African American and Caribbean American adults with intractable epilepsy: The role of perceived epilepsy stigma	Epilepsy and Behavior
Margolis, S. A. : Nakhutina, L. : Schaffer, S. G. : Grant, A. C. : Gonzalez, J. S.	2018	Perceived epilepsy stigma mediates relationships between personality and social well-being in a diverse epilepsy population	Epilepsy and Behavior
Markiewicz, A. : Hintze, B.	2016	Stigma and social support - Similarities and differences in group of women suffering from chronic diseases	Postepy Psychiatrii i Neurologii
Martiana, Ina : Waluyo, Agung : Yona, Sri	2019	Assessing the relationship between knowledge of antiretroviral therapy and stigma regarding adherence to ART among men who have sex with men	Enfermería Clínica
Martin, S. R. : Cohen, L. L. : Mougianis, I. : Griffin, A. : Sil, S. : Dampier, C.	2018	Stigma and Pain in Adolescents Hospitalized for Sickle Cell Vasoocclusive Pain Episodes	Clinical Journal of Pain
Martinez Marinho, M. : Broseghini Barcelos, L. : Hyczy de Siqueira Tosin, M. : Candeias da Silva, C. : Borges, V. : Ballalai Ferraz, H. : Silva Centeno, R.	2022	Effect of bilateral deep brain stimulation on the subthalamic nucleus on patients with Parkinson's disease: An observational and non-blinded study	Interdisciplinary Neurosurgery: Advanced Techniques and Case Management
Martínez-Fernández, A. : Rueda Vega, M. : Quintas, S. : de Toledo Heras, M. : Díaz de Terán, J. : Latorre González, G. : Trabajos García, O. : Vivancos Mora, J. : Gago-Veiga, A. B.	2020	Psychosocial repercussion of migraine: is it a stigmatized disease?	Neurological Sciences
Martínez-Zambrano, F. : Pizzimenti, M. : Barbeito, S. : Vila-Badia, R. : Comellas, G. : Escandell, M. J. : Hernández, M. J. : Fernández-de Corres, B. : González-Pinto, A. : López-Peña, M. P. : Martínez, M. : Puig, M. : Quilis, J. : Vega, P. : Ochoa, S.	2016	Spanish version of the Link's Perceived Devaluation and Discrimination scale	Psicothema
Maskay, M. H. : Cabral, H. J. : Davila, J. A. : Whitlock Davich, J. A. : Marcus, R. : Quinn, E. K. : Rajabiun, S.	2018	Longitudinal Stigma Reduction in People Living with HIV Experiencing Homelessness or Unstable Housing Diagnosed With Mental Health or Substance Use Disorders: An Intervention Study	American journal of public health
Masnari, O. : Neuhaus, K. : Aegerter, T. : Reynolds, S. : Schiestl, C. M. : Landolt, M. A.	2019	Predictors of Health-related Quality of Life and Psychological Adjustment in Children and Adolescents With Congenital Melanocytic Nevi: Analysis of Parent Reports	Journal of pediatric psychology
Matheu, M. : Sunil, T. : Castro-Peña, A. : Spears, C. E. : Smith, C. J. : Flores, J. M. : Taylor, B. S.	2020	Lack of virologic suppression is associated with lower HIV-related disclosure stigma in people living with HIV	AIDS Care - Psychological and Socio-Medical Aspects of AIDS/HIV
Mattoo, S. K. : Sarkar, S. : Nebhinani, N. : Gupta, S. : Parakh, P. : Basu, D.	2015	How Do Indian Substance Users Perceive Stigma Towards Substance Use Vis-A-Vis Their Family Members?	Journal of Ethnicity in Substance Abuse
Mattoo, S. K. : Sarkar, S. : Gupta, S. : Nebhinani, N. : Parakh, P. : Basu, D.	2015	Stigma towards substance use: Comparing treatment seeking alcohol and opioid dependent men	Int. J. Ment. Health Addict.
Maurino, J. : Martínez-Ginés, M. L. : García-Domínguez, J. M. : Solar, M. D. : Carcelén-Gadea, M. : Ares-Luque, A. : Ballabriga, J. : Navarro-Cantó, L. : Medrano, N. : Honan, C. A.	2020	Workplace difficulties, health-related quality of life, and perception of stigma from the perspective of patients with Multiple Sclerosis	Multiple Sclerosis and Related Disorders
Mazzi, F. : Baccari, F. : Mungai, F. : Ciambellini, M. : Brescancin, L. : Starace, F.	2018	Effectiveness of a social inclusion program in people with non-affective psychosis	BMC Psychiatry
Mbuthia, G. W. : Nyamogoba, H. D. N. : Chiang, S. S. : McGarvey, S. T.	2020	Burden of stigma among tuberculosis patients in a pastoralist community in Kenya: A mixed methods study	PLoS ONE
McAllister, S. : Iosua, E. : Hollingshead, B. : Bruning, J. : Fisher, M. : Olin, R. : Mukakayange, J. : Greenwood, C. : de Gouw, A. : Priest, P.	2022	Quality of life in people living with HIV in Aotearoa New Zealand: an exploratory cross-sectional study	AIDS Care - Psychological and Socio-Medical Aspects of AIDS/HIV
McCoy, Katryna : Lipira, Lauren : Kemp, Christopher G. : Nevin, Paul E. : Huh, David : Turan, Janet M. : Mugavero, Michael J. : Cohn, Susan E. : Bahk, Mieoak : Simoni, Jane M. : Andrasik, Michele P. : Rao, Deepa	2020	Exploring HIV-Related Stigma as a Determinant of Engagement in HIV Care by African American Women	JANAC: Journal of the Association of Nurses in AIDS Care
McDonnell, K. K. : Webb, L. A. : Adams, S. A. : Felder, T. M. : Davis, R. E.	2022	The association between lung cancer stigma and race: A descriptive correlational study	Health expectations : an international journal of public participation in health care and health policy

McEvoy, J. : Gandhi, S. K. : Rizio, A. A. : Maher, S. : Kosinski, M. : Bjorner, J. B. : Carroll, B.	2019	Effect of tardive dyskinesia on quality of life in patients with bipolar disorder, major depressive disorder, and schizophrenia	Quality of Life Research
McKinney, J. : Hickerson, L. : Guffey, D. : Hawkins, J. : Peters, Y. : Levison, J.	2020	Evaluation of human immunodeficiency virus-adapted group prenatal care	American Journal of Obstetrics and Gynecology MFM
McLeod, B. : Meyer, D. : Murray, G. : Foley, F. : Jones, N. : Thomas, N.	2019	Contact with recovered peers: Buffering disempowering service experiences and promoting personal recovery in serious mental illness	BJPsych Open
McManus, I. C. : Stubbings, G. F. : Martin, N.	2006	Stigmatization, physical illness and mental health in primary ciliary dyskinesia	Journal of Health Psychology
Meca-Lallana, J. E. : Prefasi, D. : Miralles, F. P. : Forero, L. : Sepúlveda, M. : Calles, C. : Martínez-Ginés, M. L. : González-Suárez, I. : Boyero, S. : Romeropinel, L. : Sempere, Á P. : Meca-Lallana, V. : Querol, L. : Costafrossard, L. : de Castro-Trapiello, H. : Canal, N. : Maurino, J.	2021	Perception of stigma in patients with neuromyelitis optica spectrum disorder	Patient Preference and Adherence
Medema-Wijnveen, J. S. : Onono, M. : Bukusi, E. A. : Miller, S. : Cohen, C. R. : Turan, J. M.	2012	How Perceptions of HIV-Related Stigma Affect Decision-Making Regarding Childbirth in Rural Kenya	PLoS ONE
Mehra, A. : Avasthi, A. : Grover, S.	2021	The Extent of Stigma Experienced by the Caregivers of Patients with OCD and its Association With Caregiver Burden and Coping	J. Psych. Rehab. Ment. Heal.
Mehta, Kedar : Baxi, Rajendra : Patel, Sangita : Chavda, Paragkumar : Mazumdar, Vihang	2019	Stigma, discrimination, and domestic violence experienced by women living with HIV: A cross-sectional study from western India	Indian Journal of Community Medicine
Meier, Amanda : Csiernik, Rick : Warner, Laura : Forchuk, Cheryl	2015	The Stigma Scale: A Canadian Perspective	Social Work Research
Mejia-Lancheros, Cilia : Lachaud, James : Woodhall-Melnik, Julia : O'Campo, Patricia : Hwang, Stephen W. : Stergiopoulos, Vicky	2021	Longitudinal interrelationships of mental health discrimination and stigma with housing and well-being outcomes in adults with mental illness and recent experience of homelessness	Social Science & Medicine
Mekuria, L. A. : Sprangers, M. A. G. : Prins, J. M. : Yalew, A. W. : Nieuwkerk, P. T.	2015	Health-related quality of life of HIV-infected adults receiving combination antiretroviral therapy in Addis Ababa	AIDS Care - Psychological and Socio-Medical Aspects of AIDS/HIV
Mendes, T. P. : Crespo, C. A. : Austin, J. K.	2017	Family Cohesion, Stigma, and Quality of Life in Dyads of Children With Epilepsy and Their Parents	Journal of pediatric psychology
Mensinger, J. L.	2022	Traumatic stress, body shame, and internalized weight stigma as mediators of change in disordered eating: a single-arm pilot study of the Body Trust® framework	Eating Disord.
Mercado, Amelia E. : Donthula, Deepanjli : Thomas, Jacob E. : Ring, David : Trust, Marc : Crijns, Tom J.	2022	Mediators and moderators of the relationship between body image and community integration among burn survivors	Burns (03054179)
Mezey, Gillian : White, Sarah : Harrison, Isobel : Bousfield, Jennifer : Killaspy, Helen : Lloyd-Evans, Brynmor : Payne, Sarah	2022	'Modelling social exclusion in a diagnostically-mixed sample of people with severe mental illness'	International Journal of Social Psychiatry
Mezey, G. : Youngman, H. : Kretzschmar, I. : White, S.	2016	Stigma and discrimination in mentally disordered offender patients - a comparison with a non-forensic population	Journal of Forensic Psychiatry and Psychology
Milacic Vidojevic, Ivona : Dragojevic, Nada : Toskovic, Oliver	2015	Experienced and anticipated discrimination among people with major depressive disorder in Serbia	The International journal of social psychiatry
Mileva, V. R. : Vázquez, G. H. : Milev, R.	2012	Effects, experiences, and impact of stigma on patients with bipolar disorder	Neuropsychiatric Disease and Treatment
Miller, D. M. : Bethoux, F. : Victorson, D. : Nowinski, C. J. : Buono, S. : Lai, J. S. : Wortman, K. : Burns, J. L. : Moy, C. : Cella, D.	2016	Validating neuro-QoL short forms and targeted scales with people who have multiple sclerosis	Multiple Sclerosis
Minichil, W. : Getinet, W. : Kassew, T.	2021	Prevalence of perceived stigma and associated factors among primary caregivers of children and adolescents with mental illness, Addis Ababa, Ethiopia: Cross-sectional study	PLoS ONE
Minja, A. A. : Larson, E. : Aloyce, Z. : Araya, R. : Kaale, A. : Kaaya, S. F. : Kamala, J. : Kasmani, M. N. : Komba, A. : Mwimba, A. : Ngakongwa, F. : Siril, H. : Smith Fawzi, M. C. : Somba, M. : Sudfeld, C. R. : Figge, C. J.	2022	Burden of HIV-related stigma and associated factors among women living with depression accessing PMTCT services in Dar es Salaam, Tanzania	AIDS Care - Psychological and Socio-Medical Aspects of AIDS/HIV
Mishra, A. : Soni, A. B. : Umate, M. S. : Andrade, C.	2021	An exploratory evaluation of predictors of duration of untreated psychosis in first-episode psychosis in lower income patients	Psychiatry Research
Mitchell, B. D. : Utterback, L. : Hibbeler, P. : Logsdon, A. R. : Smith, P. F. : Harris, L. M. : Castle, B. : Kerr, J. : Crawford, T. N.	2022	Patient-Identified Markers of Quality Care: Improving HIV Service Delivery for Older African Americans	Journal of racial and ethnic health disparities
Mitchell, M. M. : Knowlton, A.	2009	Stigma, disclosure, and depressive symptoms among informal caregivers of people living with HIV/aids	AIDS Patient Care and STDs
Mitzel, Luke D. : Foley, Jacklyn D. : Sweeney, Shannon M. : Park, Aesoon : Vanable, Peter A.	2021	Medication Beliefs, HIV-Related Stigmatization, and Adherence to Antiretroviral Therapy: An Examination of Alternative Models	Behavioral Medicine
Mitzel, Luke : Vanable, Peter : Brown, Jennifer : Bostwick, Rebecca : Sweeney, Shannon : Carey, Michael	2015	Depressive Symptoms Mediate the Effect of HIV-Related Stigmatization on Medication Adherence Among HIV-Infected Men Who Have Sex with Men	AIDS & Behavior
Mizuno, Y. : Hofer, A. : Suzuki, T. : Frajo-Apor, B. : Wartelsteiner, F. : Kemmler, G. : Saruta, J. : Tsukinoki, K. : Mimura, M. : Fleischhacker, W. W. : Uchida, H.	2016	Clinical and biological correlates of resilience in patients with schizophrenia and bipolar disorder: A cross-sectional study	Schizophrenia Research
Mlouki, I. : Zammit, N. : Ghammem, R. : Ben Fredj, S. : Bannour, R. : El Echi, A. : Ladhari, H. : Haddedi, A. : Ghodhbani, M. M. : Maatoug, J. : Ghannem, H.	2022	Validity and reliability of a modified short version of a stigma scale for use among Tunisian COVID-19 patients after quarantine: A cross-sectional study	Health Science Reports
Mo, P. K. H. : Mak, W. W. S.	2009	Intentionality of medication non-adherence among individuals living with HIV/AIDS in Hong Kong	AIDS Care - Psychological and Socio-Medical Aspects of AIDS/HIV
Modelli, A. : Candal Setti, V. P. : van de Bilt, M. T. : Gattaz, W. F. : Loch, A. A. : Rössler, W.	2020	Addressing Mood Disorder Diagnosis' Stigma With an Honest, Open, Proud (HOP)-Based Intervention: A Randomized Controlled Trial	Frontiers in Psychiatry
Modi, L. : Shivji, I. A. : Behere, P. B. : Mishra, K. K. : Patil, P. S. : Goyal, A.	2017	A clinical study of self-stigma among the patients of schizophrenia and alcohol dependence syndrome	J. Datta Meghe Inst. Med. Sci. Univ.
Moges, N. A. : Adesina, O. A. : Okunlola, M. A. : Berhane, Y. : Akinyemi, J. O.	2021	Psychological Distress and Its Correlates among Newly Diagnosed People Living with HIV in Northwest Ethiopia: Ordinal Logistic Regression Analyses	Infectious Diseases: Research and Treatment
Mohammed, Q. Q.	2018	The influence of mental illness stigma on medication adherence in patients with Schizophrenia in Baghdad city	Indian Journal of Public Health Research and Development
Mohammedhussein, M. : Alenko, A. : Tessema, W. : Mamaru, A.	2020	Prevalence and associated factors of depression and anxiety among patients with pulmonary tuberculosis attending treatment at public health facilities in southwest ethiopia	Neuropsychiatric Disease and Treatment
Mohammedhussein, M. : Hajure, M. : Shifa, J. E. : Hassen, T. A.	2020	Perceived stigma among patient with pulmonary tuberculosis at public health facilities in southwest Ethiopia: A cross-sectional study	PLoS ONE
Moideen, S. : Uvais, N. A. : Rajagopal, S. : Maheshwari, V. : Gafoor, T. A. : Sherief, S. H.	2021	COVID-19-Related Stigma Among Inpatients With COVID-19 Infection	Prim. Care Companion CNS Disord.
Moitra, Ethan : Chan, Philip A. : Stein, Michael D.	2015	Open Trial of an Acceptance-Based Behavior Therapy Intervention to Engage Newly Diagnosed HIV Patients in Care	Behavior Modification
Molina, Y. : Choi, S. W. : Cella, D. : Rao, D.	2013	The stigma scale for chronic illnesses 8-item version (SSCI-8): development, validation and use across neurological conditions	International journal of behavioral medicine
Monden, K. R. : Philippus, A. : MacIntyre, B. : Welch, A. : Sevigny, M. : Draganich, C. : Agtarap, S. : Morse, L. R.	2021	The Impact of Stigma on Psychosocial Outcomes Following Spinal Cord Injury: A Cross-Sectional Analysis of Stigma-Mediated Relationships	Rehabil. Psychol.
Moon, Hye-Jin : Seo, Jong-Geun : Park, Sung-Pa	2016	Perceived stress and its predictors in people with epilepsy	Epilepsy & behavior : E&B
Moore, D. : Ayers, S. : Drey, N.	2017	The City MISS: development of a scale to measure stigma of perinatal mental illness	J. Reprod. Infant Psychol.
Moore, O. : Peretz, C. : Giladi, N.	2007	Freezing of gait affects quality of life of peoples with Parkinson's disease beyond its relationships with mobility and gait	Movement Disorders
Moradzadeh, Rahmatollah : Zamanian, Maryam	2021	HIV-Related StigmaAmongPeople Living With HIV in Iran: A Cross-Sectional Study	JANAC: Journal of the Association of Nurses in AIDS Care
Moritz, S. : Schröder, J. : Meyer, B. : Hauschildt, M.	2013	The more it is needed, the less it is wanted: Attitudes toward face-to-face intervention among depressed patients undergoing online treatment	Depression and Anxiety
Morrison, A. P. : Birchwood, M. : Pyle, M. : Flach, C. : Stewart, S. L. K. : Byrne, R. : Patterson, P. : Jones, P. B. : Fowler, D. : Gumley, A. I. : French, P.	2013	Impact of cognitive therapy on internalised stigma in people with at-risk mental states	Br. J. Psychiatry
Morrison, A. P. : Burke, E. : Murphy, E. : Pyle, M. : Bowe, S. : Varese, F. : Dunn, G. : Chapman, N. : Hutton, P. : Welford, M. : Wood, L. J.	2016	Cognitive therapy for internalised stigma in people experiencing psychosis: A pilot randomised controlled trial	Psychiatry Res.
Morton, M. L. : Teagarden, D. L. : Groover, O. A. : Karakis, I.	2018	Disparities in patients with psychogenic non-epileptic seizures and their caregivers	Annals of Neurology
Mosanya, T. J. : Adelufosi, A. O. : Adebowale, O. T. : Ogunwale, A. : Adebayo, O. K.	2014	Self-stigma, quality of life and schizophrenia: An outpatient clinic survey in Nigeria	The International journal of social psychiatry

Moser, E. : Chan, F. : Berven, N. L. : Bezyak, J. : Iwanaga, K. : Umucu, E.	2020	Resilience and life satisfaction in young adults with epilepsy: The role of person-environment contextual factors	Journal of Vocational Rehabilitation
Moses, T.	2014	Determinants of mental illness stigma for adolescents discharged from psychiatric hospitalization	Soc. Sci. Med.
Moses, T.	2015	Coping strategies and self-stigma among adolescents discharged from psychiatric hospitalization: A 6-month follow-up study	Int. J. Soc. Psychiatry
Moura, S. H. L. : Grossi, M. A. F. : Lehman, L. F. : Salgado, S. P. : Almeida, C. A. : Lyon, D. T. : Lyon, S. : Rocha, M. O. C.	2017	Epidemiology and assessment of the physical disabilities and psychosocial disorders in new leprosy patients admitted to a referral hospital in Belo Horizonte, Minas Gerais, Brazil	Lepr. Rev.
Mousley, E. : Deribe, K. : Tamiru, A. : Davey, G.	2013	The impact of podoconiosis on quality of life in Northern Ethiopia	Health and Quality of Life Outcomes
Mroueh, L. : Boumediene, F. : Jost, J. : Ratsimbazafy, V. : Preux, P. M. : Salameh, P. : Al-Hajje, A.	2020	Quality of life and stigma in Lebanese people with epilepsy taking medication	Epilepsy Research
Mulfinger, N. : Müller, S. : Böge, I. : Sakar, V. : Corrigan, P. W. : Evans-Lacko, S. : Nehf, L. : Djamali, J. : Samarelli, A. : Kempter, M. : Ruckes, C. : Libal, G. : Oexle, N. : Noterdaeme, M. : Rüsch, N.	2018	Honest, Open, Proud for adolescents with mental illness: pilot randomized controlled trial	J. Child Psychol. Psychiatry Allied Discip.
Müller, A. D. : Bode, S. : Myer, L. : Stahl, J. : Von Steinbüchel, N.	2011	Predictors of adherence to antiretroviral treatment and therapeutic success among children in South Africa	AIDS Care Psychol. Socio-Med. Asp. AIDS HIV
Münchau, A. : Palmer, J. D. : Dressler, D. : O'Sullivan, J. D. : Tsang, K. L. : Jahanshahi, M. : Quinn, N. P. : Lees, A. J. : Bhatia, K. P. : Bhatia, K. P.	2001	Prospective study of selective peripheral denervation for botulinum-toxin resistant patients with cervical dystonia	Brain
Muñoz, M. : Sanz, M. : Pérez-Santos, E. : Quiroga, M. D. L. A.	2011	Proposal of a socio-cognitive-behavioral structural equation model of internalized stigma in people with severe and persistent mental illness	Psychiatry Res.
Muñoz-Laboy, Miguel : Guidry, John A. : Kreisberg, Alexa	2022	Internalised stigma as durable social determinant of HIV care for transnational patients of Puerto Rican ancestry	Global Public Health
Murphy, P. J. : Garrido-Hernansaiz, H. : Mulcahy, F. : Hevey, D.	2018	HIV-related stigma and optimism as predictors of anxiety and depression among HIV-positive men who have sex with men in the United Kingdom and Ireland	AIDS Care Psychol. Socio-Med. Asp. AIDS HIV
Murri, M. B. : Amore, M. : Calcagno, P. : Respino, M. : Marozzi, V. : Masotti, M. : Bugliani, M. : Innamorati, M. : Pompili, M. : Galderisi, S. : Maj, M.	2016	The "insight Paradox" in schizophrenia: Magnitude, moderators and mediators of the association between insight and depression	Schizophrenia Bulletin
Muslih, M. : Chiu, W. C. : Chuang, Y. H. : Chung, M. H.	2021	Psychometric properties of the Internalized Stigma of Mental Illness (ISMI) Scale in Indonesia	Psychiatric rehabilitation journal
Mutiso, V. N. : Pike, K. : Musyimi, C. W. : Rebello, T. J. : Tele, A. : Gitonga, I. : Thornicroft, G. : Ndetei, D. M.	2019	Feasibility of WHO mhGAP-intervention guide in reducing experienced discrimination in people with mental disorders: A pilot study in a rural Kenyan setting	Epidemiology and Psychiatric Sciences
Muze, R. H.	2009	Relationships between HIV stigma, parenting stress, social support network, physical functioning, and emotional well-being among HIV infected African American women	Journal of the National Society of Allied Health
Mwangala, P. N. : Nasambu, C. : Wagner, R. G. : Newton, C. R. : Abubakar, A.	2022	Prevalence and factors associated with mild depressive and anxiety symptoms in older adults living with HIV from the Kenyan coast	Journal of the International AIDS Society
Myers, A. : Rosen, J. C.	1999	Obesity stigmatization and coping: Relation to mental health symptoms, body image, and self-esteem	International Journal of Obesity
Nabunya, P. : Byansi, W. : Sensoy Bahar, O. : McKay, M. : Ssewamala, F. M. : Damulira, C.	2020	Factors Associated With HIV Disclosure and HIV-Related Stigma Among Adolescents Living With HIV in Southwestern Uganda	Frontiers in Psychiatry
Nabunya, P. : Namuwonge, F.	2022	HIV-Related Shame, Stigma and the Mental Health Functioning of Adolescents Living with HIV: Findings from a Pilot Study in Uganda	Child psychiatry and human development
Nadia, A. B. : Leelavathi, M. : Narul Aida, S. : Diana, M.	2017	Self-Perceived Stigma and its Effect on Quality of Life among Malaysians Living with Human Immunodeficiency Virus	Medicine & Health (Universiti Kebangsaan Malaysia)
Nagarathnam, M. : Vengamma, B. : Shalini, B. : Latheef, S.	2017	Stigma and polytherapy: Predictors of quality of life in patients with epilepsy from South India	Annals of Indian Academy of Neurology
Nakandi, R. M. : Kiconco, P. : Musiimenta, A. : Bwengye, J. J. : Nalugya, S. : Kyomugisa, R. : Obua, C. : Atukunda, E. C.	2022	Understanding patterns of family support and its role on viral load suppression among youth living with HIV aged 15 to 24 years in southwestern Uganda	Health Science Reports
Nambiar, D. : Ramakrishnan, V. : Kumar, P. : Varma, R. : Balaji, N. : Rajendran, J. : Jhona, L. : Chandrasekar, C. : Gere, D.	2011	Knowledge, stigma, and behavioral outcomes among antiretroviral therapy patients exposed to Nalamdana's radio and theater program in Tamil Nadu, India	AIDS Education and Prevention
Nattabi, B. : Li, J. : Thompson, S. C. : Orach, C. G. : Earnest, J.	2011	Factors associated with perceived stigma among peopleliving with hiv/aids in post-conflict northern Uganda	AIDS Education and Prevention
Naushad, N. : Dunn, L. B. : Muñoz, R. F. : Leykin, Y.	2018	Depression increases subjective stigma of chronic pain	Journal of Affective Disorders
Nazar, I. : Kamran, F. : Masood, A.	2021	Psychosocial Predictors of Quality of Life in Patients With Vitiligo	Pak. J. Psychol. Res.
Nazzaro, J. M. : Pahwa, R. : Lyons, K. E.	2012	Long-term benefits in quality of life after unilateral thalamic deep brain stimulation for essential tremor: Clinical article	Journal of Neurosurgery
Negi, B. S. : Joshi, S. K. : Nakazawa, M. : Kotaki, T. : Bastola, A. : Kameoka, M.	2018	Impact of a massive earthquake on adherence to antiretroviral therapy, mental health, and treatment failure among people living with HIV in Nepal	PLoS ONE
Nejatisafa, A. A. : Mozafari, S. : Noorbala, A. A. : Asgarian, F. S. : Earnshaw, V. A. : Sahraian, M. A. : Etesam, F.	2017	Psychometric Evaluation of the Persian Version of the Chronic Illness Anticipated Stigma Scale (CIASS)	International journal of behavioral medicine
Nelson, C. B. : Bowersox, N. : King, J. : Hunt, M. G.	2020	Psychometric examination of care quality measures in VA psychosocial rehabilitation and recovery centers (PRRCs)	Psychol. Serv.
Nestadt, Danielle Friedman : Saisaengjan, Chutima : McKay, Mary McKernan : Bunupuradah, Torsak : Pardo, Gisselle : Lakhonpon, Sudrak : Gopalan, Priya : Leu, Cheng-Shiun : Petdachai, Witaya : Kosalaraksa, Pope : Srirompotong, Ussanee : Ananworanich, Jintanat : Mellins, Claude Ann	2019	CHAMP+ Thailand: Pilot Randomized Control Trial of a Family-Based Psychosocial Intervention for Perinatally HIV-Infected Early Adolescents	AIDS Patient Care & STDs
Nevarez-Flores, Adriana G. : Breslin, Monique : Carr, Vaughan J. : Morgan, Vera A. : Waterreus, Anna : Harvey, Carol : Sanderson, Kristy : Neil, Amanda L.	2022	Health-related quality of life in people with psychotic disorders: The role of loneliness and its contributors	Australian & New Zealand Journal of Psychiatry
Nevarez-Flores, Adriana G. : Morgan, Vera A. : Harvey, Carol : Breslin, Monique : Carr, Vaughan J. : Sanderson, Kristy : Waterreus, Anna : Neil, Amanda L.	2020	Health-related quality of life, functioning and social experiences in people with psychotic disorders	Applied Research in Quality of Life
Ngai, Steven Sek-yum : Cheung, Chau-kiu : Ng, Yuen-hang : Tang, Hon-yin : Ngai, Hui-lam : Wong, Kenix Hok-ching	2020	Development and validation of the chronic illness self-management (CISM) scale: Data from a young patient sample in Hong Kong	Children & Youth Services Review
Ngo, X. M. : Nguyen, T. T. H. : Nguyen, T. T. T. : Doan, L. N. G. : Ngo, N. T. : Lam, T. V. : Nguyen, T. D.	2019	Quality of life among patients with Parkinson's disease: A cross-sectional study in Vietnam	Systematic Reviews in Pharmacy
Ngoc, T. N. : Weiss, B. : Trung, L. T.	2016	Effects of the family schizophrenia psychoeducation program for individuals with recent onset schizophrenia in Viet Nam	Asian journal of psychiatry
Nigussie, K. : Lemma, A. : Sertsu, A. : Asfaw, H. : Kerebih, H. : Abdeta, T.	2021	Depression, anxiety and associated factors among people with epilepsy and attending outpatient treatment at primary public hospitals in northwest Ethiopia: A multicenter cross-sectional study	PLoS ONE
Nilsson, K. K. : Kugathasan, P. : Straarup, K. N.	2016	Characteristics, correlates and outcomes of perceived stigmatization in bipolar disorder patients	Journal of Affective Disorders
Nkambule, B. S. : Lee-Hsieh, J. : Liu, C. Y. : Cheng, S. F.	2019	The relationship between patients' perception of nurse caring behaviors and tuberculosis stigma among patients with drug-resistant tuberculosis in Swaziland	International Journal of Africa Nursing Sciences
Noble, A. J. : McCrone, P. : Seed, P. T. : Goldstein, L. H. : Ridsdale, L.	2014	Clinical- and cost-effectiveness of a nurse led self-management intervention to reduce emergency visits by people with epilepsy	PLoS ONE
Nobre, N. : Pereira, M. : Roine, R. P. : Sutinen, J. : Sintonen, H.	2018	HIV-Related Self-Stigma and Health-Related Quality of Life of People Living With HIV in Finland	The Journal of the Association of Nurses in AIDS Care : JANAC
Nöstlinger, C. : Bakeera-Kitaka, S. : Buyze, J. : Loos, J. : Buvé, A.	2015	Factors influencing social self-disclosure among adolescents living with HIV in Eastern Africa	AIDS Care - Psychological and Socio-Medical Aspects of AIDS/HIV
Nurfalah, Firhan : Yona, Sri : Waluyo, Agung	2019	The relationship between HIV stigma and adherence to antiretroviral (ARV) drug therapy among women with HIV in Lampung, Indonesia	Enfermería Clínica
Nursalam, : Sukartini, T. : Trisusanti, E. H. : Sari, N. A. : Nurhayati, C. : Rustini, S. A. : Huda, N.	2022	Stigma and Anxiety Levels With Adherence on the Treatment Schedule Patient With HIV/AIDS in Indonesia	Malays. J. Med. Health Sci.
Nyamathi, Adeline M. : Ekstrand, Maria : Yadav, Kartik : Ramakrishna, Padma : Heylen, Elsa : Carpenter, Catherine : Wall, Sarah : Oleskowicz, Tanya : Arab, Lenore : Sinha, Sanjeev	2017	Quality of Life Among Women Living With HIV in Rural India	JANAC: Journal of the Association of Nurses in AIDS Care
Nyandiko, W. : Chory, A. : Baum, A. : Aluoch, J. : Ashimosi, C. : Scanlon, M. : Martin, R. : Wachira, J. : Beigon, W. : Munyoro, D. : Apondi, E. : Vreeman, R.	2022	Multi-media teacher training and HIV-related stigma among primary and secondary school teachers in Western Kenya	AIDS Care - Psychological and Socio-Medical Aspects of AIDS/HIV
Nyongesa, M. K. : Mwangi, P. : Koot, H. M. : Cuijpers, P. : Newton, C. R. J. C. : Abubakar, A.	2020	The reliability, validity and factorial structure of the Swahili version of the 7-item generalized anxiety disorder scale (GAD-7) among adults living with HIV from Kilifi, Kenya	Annals of General Psychiatry

Nyongesa, M. K. : Mwangi, P. : Kinuthia, M. : Hassan, A. S. : Koot, H. M. : Cuijpers, P. : Newton, C. R. J. C. : Abubakar, A.	2021	Prevalence, risk and protective indicators of common mental disorders among young people living with HIV compared to their uninfected peers from the Kenyan coast: a cross-sectional study	BMC Psychiatry
Nyongesa, M. K. : Nyongesa, M. K. : Mwangi, P. : Wanjala, S. W. : Mutua, A. M. : Koot, H. M. : Cuijpers, P. : Newton, C. R. J. C. : Newton, C. R. J. C. : Newton, C. R. J. C. : Abubakar, A. : Abubakar, A. : Abubakar, A. : Abubakar, A.	2020	Correlates of health-related quality of life among adults receiving combination antiretroviral therapy in coastal Kenya	Health and Quality of Life Outcomes
O'Brien, J. A. : Hickman, R. L. : Burant, C. : Dolansky, M. : Padrino, S.	2022	Health Literacy, Perceived Stigma, Self-Efficacy, and HRQOL in Sickle Cell Disease	Western journal of nursing research
O'Suilleabhain, P. : Tovar, M. : Shukla, A. W. : Tester, N. J. : Lundervold, D. A. : Turner, T. H. : Howe-Martin, L. : Louis, E. D.	2022	Development of ETStig, a measure for stigma in essential tremor	Parkinsonism and Related Disorders
O'Sullivan, M. : Jones, A. M. : Gage, H. : Jordan, J. : MacPepple, E. : Williams, H. : Verma, S.	2020	ITTREAT (Integrated Community Test - Stage - TREAT) Hepatitis C service for people who use drugs: Real-world outcomes	Liver International
Ober, Allison J. : Watkins, Katherine E. : McCullough, Colleen M. : Setodji, Claude M. : Osilla, Karen : Hunter, Sarah B.	2018	Patient predictors of substance use disorder treatment initiation in primary care	Journal of Substance Abuse Treatment
Öcel, Hatem	2017	Meme kanseri tanısı almış çalışan kadınlarda damgalanma ve bilinçli farkındalık ile psikolojik iyi oluş arasındaki ilişkiler: Psikolojik esnekliğin düzenleyici rolü = The relations between stigmatization and mindfulness with psychological well-being among	Türk Psikoloji Dergisi
Ochoa, S. : Martínez-Zambrano, F. : Garcia-Franco, M. : Vilamala, S. : Ribas, M. : Arenas, O. : Garcia-Morales, E. : Álvarez, I. : Escartin, G. : Villellas, R. : Escandell, M. J. : Martínez-Raves, M. : López-Arias, E. : Cunyat, C. : Haro, J. M.	2015	Development and validation of the Self-Stigma Questionnaire (SSQ) for people with schizophrenia and its relation to social functioning	Comprehensive Psychiatry
Ochoa-Morales, A. : Fresan-Orellana, A. : Hernández-Mojica, T. : Jara-Prado, A. : Corona-Vázquez, T. : Flores-Rivera, J. J. : Rito-García, C. Y. : Rivas-Alonso, V. : Guerrero-Camacho, J. L. : Dávila-Ortiz de Montellano, D. J.	2021	Perceived discrimination in patients with multiple sclerosis and depressive symptomatology	Multiple Sclerosis and Related Disorders
Ociskova, Marie : Prasko, Jan : Kamaradova, Dana : Latalova, Klara : Kurfurst, Pavel : Dostalova, Lenka : Cinculova, Andrea : Kubinek, Radim : Mainerova, Barbora : Vrbova, Kristyna : Tichackova, Anezka	2014	Self-stigma in psychiatric patients--standardization of the ISMI scale	Neuro endocrinology letters
Ociskova, M. : Prasko, J. : Kamaradova, D. : Grambal, A. : Sigmundova, Z.	2015	Individual correlates of self-stigma in patients with anxiety disorders with and without comorbidities	Neuropsychiatric Disease and Treatment
Ociskova, M. : Prasko, J. : Sigmundova, Z. : Grambal, A. : Kamaradova, D. : Latalova, K. : Jelenova, D. : Vrbova, K.	2015	Relationship between internalized stigma and treatment efficacy in mixed neurotic spectrum and depressive disorders	European Psychiatry
Ociskova, M. : Prasko, J. : Kamaradova, D.	2015	Relationship between personality and self-stigma in mixed neurotic spectrum and depressive disorders - cross sectional study	Activitas Nervosa Superior Rediviva
Ociskova, M. : Prasko, J. : Latalova, K. : Kamaradova, D. : Grambal, A.	2016	Psychological factors and treatment effectiveness in resistant anxiety disorders in highly comorbid inpatients	Neuropsychiatric Disease and Treatment
Ociskova, M. : Prasko, J. : Kamaradova, D. : Marackova, M. : Holubova, M.	2016	Evaluation of the psychometric properties of the brief Internalized Stigma of Mental Illness Scale (ISMI-10)	Neuroendocrinology Letters
Ociskova, M. : Prasko, J. : Vrbova, K. : Kasalova, P. : Holubova, M. : Grambal, A. : Machu, K.	2018	Self-stigma and treatment effectiveness in patients with anxiety disorders- a mediation analysis	Neuropsychiatric Disease and Treatment
Ociskova, Marie : Prasko, Jan : Vanek, Jakub : Holubova, Michaela : Hodny, Frantisek : Latalova, Klara : Kantor, Krystof : Nesnidal, Vlastimil	2021	Self-Stigma and Treatment Effectiveness in Patients with SSRI Non-Responsive Obsessive-Compulsive Disorder	Psychology research and behavior management
Oduenyi, Chioma : Ugwa, Emmanuel : Ojukwu, Zimako : Ojukwu-Ajasigwe, Jachike	2019	An Exploratory Study of Stigma and Discrimination among People Living with HIV/ AIDS in Abuja Municipal Area Council, Nigeria	African Journal of Reproductive Health
Oduguwa, T. O. : Akinwotu, O. O. : Adeoye, A. A.	2014	A comparative study of self stigma between HIV/AIDS and schizophrenia patients	African Journal of Psychiatry (South Africa)
Oexle, N. : Müller, M. : Kawohl, W. : Xu, Z. : Viering, S. : Wyss, C. : Vetter, S. : Rüsch, N.	2018	Self-stigma as a barrier to recovery: a longitudinal study	Eur. Arch. Psychiatry Clin. Neurosci.
Ofori-Atta, A. : Reynolds, N. R. : Antwi, S. : Renner, L. : Nichols, J. S. : Lartey, M. : Amissah, K. : Tettey, J. K. : Alhassan, A. : Ofori, I. P. : Catlin, A. C. : Gan, G. : Kyriakides, T. C. : Paintsil, E.	2019	Prevalence and correlates of depression among caregivers of children living with HIV in Ghana: findings from the Sankofa pediatric disclosure study	AIDS Care - Psychological and Socio-Medical Aspects of AIDS/HIV
Ogawa, M. : Fujikawa, M. : Jin, K. : Kakisaka, Y. : Ueno, T. : Nakasato, N.	2021	Acceptance of disability predicts quality of life in patients with epilepsy	Epilepsy and Behavior
Ogawa, M. : Fujikawa, M. : Tasaki, K. : Jin, K. : Kakisaka, Y. : Nakasato, N.	2022	Development and validation of the Japanese version of the Epilepsy Stigma Scale in adults with epilepsy	Epilepsy and Behavior
Ograczyk, A. : Malec, J. : Miniszewska, J. : Zalewska-Janowska, A.	2012	Psychological aspects of atopic dermatitis and contact dermatitis: Stress coping strategies and stigmatization	Postepy Dermatologii i Alergologii
Oh, C. : Li, H. : Lee, W. : Tey, H.	2015	Biopsychosocial factors associated with prurigo nodularis in endogenous eczema	Indian Journal of Dermatology
Ohlsson-Nevo, Emma : Ahlgren, Johan : Karlsson, Jan	2020	Impact of health-related stigma on psychosocial functioning in cancer patients: Construct validity of the stigma-related social problems scale	European journal of cancer care
Oka, A. : Tanikawa, C. : Isogai, Y. : Mihara, K. : Yamashiro, T.	2022	Evaluation of Facial Appearance-Related Quality of Life in Young Japanese Patients With Cleft Lip and/or Palate	Cleft Palate-Craniofacial Journal
Oka, A. : Tanikawa, C. : Ohara, H. : Yamashiro, T.	2022	Relationship Between Stigma Experience and Self-Perception Related to Facial Appearance in Young Japanese Patients with Cleft lip and/or Palate	Cleft Palate-Craniofacial Journal
Oke, O. O. : Akinboro, A. O. : Olanrewaju, F. O. : Oke, O. A. : Omololu, A. S.	2019	Assessment of HIV-related stigma and determinants among people living with HIV/AIDS in Abeokuta, Nigeria: A cross-sectional study	SAGE Open Medicine
Okhakhume, Aide Sylvester	2012	Influence of psychological factors on self and perceived stigma and the efficacy of cognitive behaviour therapy in symptoms reduction among mentally ill patients	IFE Psychologia: An International Journal
Olagundoye, O. : Akhuemokhan, V. : Alugo, M.	2017	Towards caring for caregivers: Assessing the burden of care and experience of associative stigma among caregivers of patients with chronic mental illnesses at a mental health care facility in Lagos Metropolis, Nigeria	Family Medicine and Primary Care Review
Olçun, Z. : Şahin Altun, Ö	2017	The Correlation Between Schizophrenic Patients' Level of Internalized Stigma and Their Level of Hope	Archives of psychiatric nursing
Oleniuk, A. : Duncan, C. R. : Tempier, R.	2013	The impact of stigma of mental illness in a Canadian community: a survey of patients experiences	Community mental health journal
Oliveira, M. C. : Lima, E. M. : de Paiva, M. L. N. : Valente, K. D. R.	2022	Factors associated with caregiver burden of adults with epilepsy in a middle-income country	Seizure
Oliveira, S. E. H. : Carvalho, H. : Esteves, F.	2016	Internalized stigma and quality of life domains among people with mental illness: The mediating role of self-esteem	Journal of Mental Health
Oliver, K. N. : Free, M. L. : Bok, C. : McCoy, K. S. : Lemanek, K. L. : Emery, C. F.	2014	Stigma and optimism in adolescents and young adults with cystic fibrosis	J. Cyst. Fibrosis
Olley, B. O.	2004	Psychosocial and seizure factors related to depression and neurotic-disorders among patients with chronic epilepsy in Nigeria	African journal of medicine and medical sciences
Olley, B. O. : Ogunde, M. J. : Oso, P. O. : Ishola, A.	2016	HIV-related stigma and self-disclosure: the mediating and moderating role of anticipated discrimination among people living with HIV/AIDS in Akure Nigeria	AIDS Care
Omiya, Tomoko : Yamazaki, Yoshihiko : Shimada, Megumi : Ikeda, Kazuko : Ishiuchi-Ishitani, Seiko : Tsuno, Yoko Sumikawa : Ohira, Katsumi	2014	Mental health of patients with human immunodeficiency virus in Japan: a comparative analysis of employed and unemployed patients	AIDS care
Ongenae, K. : Dierckxsens, L. : Brochez, L. : Van Geel, N. : Naeyaert, J. M.	2005	Quality of life and stigmatization profile in a cohort of vitiligo patients and effect of the use of camouflage	Dermatology
Onono, Maricianah : Owuor, Kevin : Turan, Janet : Bukusi, Elizabeth A. : Gray, Glenda E. : Cohen, Craig R.	2015	The Role of Maternal, Health System, and Psychosocial Factors in Prevention of Mother-to-Child Transmission Failure in the Era of Programmatic Scale Up in Western Kenya: A Case Control Study	AIDS Patient Care & STDs
Onu, D. U.	2021	Treatment adherence mediates the relationship between HIV-related stigma and health-related quality of life	AIDS Care - Psychological and Socio-Medical Aspects of AIDS/HIV
Onyebuchi-Iwudibia, O. : Brown, A.	2014	HIV and depression in Eastern Nigeria: The role of HIV-related stigma	AIDS Care - Psychological and Socio-Medical Aspects of AIDS/HIV
Orsi, Jose Alberto : Malinowski, Fernando Rocha Loures : Kagan, Simao : Weingarten, Richard : Villares, Cecilia Cruz : Bressan, Rodrigo Affonseca : de Oliveira, Walter Ferreira : Andrade, Mario Cesar Rezende : Gadelha, Ary	2021	Evaluation of Ongoing Participation of People with Schizophrenia in a Mutual Support Group as a Complementary Intervention to Outpatient Psychiatric Treatment	The Psychiatric quarterly
Osborn, L. : Ronen, K. : Larsen, A. M. : Richardson, B. : Khasimwa, B. : Chohan, B. : Matemo, D. : Unger, J. : Drake, A. L. : Kinuthia, J. : John-Stewart, G.	2022	Antenatal depressive symptoms in Kenyan women living with HIV: contributions of recent HIV diagnosis, stigma, and partner violence	AIDS Care - Psychological and Socio-Medical Aspects of AIDS/HIV
Ostroff, J. S. : Riley, K. E. : Shen, M. J. : Atkinson, T. M. : Williamson, T. J. : Hamann, H. A.	2019	Lung cancer stigma and depression: Validation of the Lung Cancer Stigma Inventory	Psycho-Oncology
Ouimette, Paige : Vogt, Dawne : Wade, Michael : Tirone, Vanessa : Greenbaum, Mark A. : Kimerling, Rachel : Laffaye, Charlene : Fitt, Julie E. : Rosen, Craig S.	2011	Perceived barriers to care among veterans health administration patients with posttraumatic stress disorder	Psychological Services
Overstreet, N. M. : Earnshaw, V. A. : Kalichman, S. C. : Quinn, D. M.	2013	Internalized stigma and HIV status disclosure among HIV-positive black men who have sex with men	AIDS Care - Psychological and Socio-Medical Aspects of AIDS/HIV

Ow, C. Y. : Lee, B. O.	2015	Relationships between perceived stigma, coping orientations, self-esteem, and quality of life in patients with schizophrenia	Asia-Pacific journal of public health / Asia-Pacific Academic Consortium for Public Health
Owczarzak, J. : Fuller, S. : Coyle, C. : Davey-Rothwell, M. : Kiriazova, T. : Tobin, K.	2022	The Relationship Between Intersectional Drug Use and HIV Stigma and HIV Care Engagement Among Women Living with HIV in Ukraine	AIDS and behavior
Öz, B. : Yüksel, T. : Nasiroğlu, S.	2020	Depression-anxiety symptoms and stigma perception in mothers of children with autism spectrum disorder	Noropsikiyatri Arsivi
Özkesici Kurt, B. : İnan Doğan, E. : Özdemir, M. : Alpsoy, E.	2021	Internalized stigma: One of the main factors responsible for the psychosocial burden of melasma?	Journal of Cosmetic Dermatology
Ozturk, A. : Akin, S. : Kundakci, N.	2022	Self-esteem mediates the relationship perceived stigma with self-efficacy for diabetes management in individuals with type 2 diabetes mellitus	Saudi medical journal
Öztürk, Zeynep : Şahin Altun, Özlem	2021	The effect of nursing interventions to instill hope on the internalized stigma, hope, and quality of life levels in patients with schizophrenia	Perspectives in Psychiatric Care
Padilla, A. H. : Palmer, P. M. : Rodríguez, B. L.	2019	The Relationship Between Culture, Quality of Life, and Stigma in Hispanic New Mexicans With Dysphagia: A Preliminary Investigation Using Quantitative and Qualitative Analysis	American journal of speech-language pathology
Pahwa, R. : Stacy, M. A. : Factor, S. A. : Lyons, K. E. : Stocchi, F. : Hersh, B. P. : Elmer, L. W. : Truong, D. D. : Earl, N. L.	2007	Ropinirole 24-hour prolonged release: Randomized, controlled study in advanced Parkinson disease	Neurology
Paintsil, E. : Renner, L. : Antwi, S. : Dame, J. : Enimil, A. : Ofori-Atta, A. : Alhassan, A. : Ofori, I. P. : Cong, X. : Kyriakides, T. : Reynolds, N. R.	2015	HIV knowledge, stigma, and illness beliefs among pediatric caregivers in Ghana who have not disclosed their child's HIV status	AIDS Care - Psychological and Socio-Medical Aspects of AIDS/HIV
Pakdewong, P. : Kasemkitwatana, S. : Puncharoenworakul, K. : Miles, M. S. : Kaemkate, W.	2006	A structural model of maternal role attainment in Thai HIV sero-positive mothers	Thai Journal of Nursing Research
Pakhale, S. : Armstrong, M. : Holly, C. : Edjoc, R. : Gaudet, E. : Aaron, S. : Tasca, G. : Cameron, W. : Balfour, L.	2014	Assessment of stigma in patients with cystic fibrosis	BMC Pulmonary Medicine
Pal, A. : Sharan, P. : Chadda, R. K.	2017	Internalized stigma and its impact in Indian outpatients with bipolar disorder	Psychiatry Research
Palar, Kartika : Sheira, Lila A. : Weiser, Sheri D. : Metsch, Lisa : Tien, Phyllis C. : Turan, Janet M. : Frongillo, Edward A. : Escobar, Jessica : Wilson, Tracey E. : Adedimeji, Adebola : Merenstein, Daniel : Cohen, Mardge H. : Wentz, Eryka L. : Adimora, Adaora A. : Ofotokun, Ighovwerha	2018	Food Insecurity, Internalized Stigma, and Depressive Symptoms Among Women Living with HIV in the United States	AIDS & Behavior
Palmeira, L. : Pinto-Gouveia, J. : Cunha, M.	2017	Exploring the efficacy of an acceptance, mindfulness & compassionate-based group intervention for women struggling with their weight (Kg-Free): A randomized controlled trial	Appetite
Paltun, Birsen : Bölükbaş, Nurgül	2020	Turkish version of the cancer stigma scale: Validity and reliability study	Psychology, Health & Medicine
Pampalia, N. : Waluyo, A. : Yona, S.	2021	Knowledge, stigma and health-seeking behavior of patients co-infected with HIV and tuberculosis in Jakarta	Enferm. Clin.
Pantelic, M. : Boyes, M. : Cluver, L. : Meinck, F.	2017	HIV, violence, blame and shame: Pathways of risk to internalized HIV stigma among South African adolescents living with HIV: Pathways	Journal of the International AIDS Society
Papathanasiou, I. : MacDonald, L. : Whurr, R. : Jahanshahi, M.	2001	Perceived stigma in spasmodic torticollis	Mov. Disord.
Paraskevoulakou, Alexia : Vrettou, Kassiani : Pikouli, Katerina : Triantafillou, Evgenia : Lykou, Anastasia : Economou, Marina	2017	Mental Illness Related Internalized Stigma: Psychometric Properties of the Brief ISMI Scale in Greece	Materia socio-medica
Parcesepe, A. M. : Filiatreau, L. M. : Ebasone, P. V. : Dzudie, A. : Pence, B. W. : Wainberg, M. : Yotebieng, M. : Anastos, K. : Pefura-Yone, E. : Nsame, D. : Ajeh, R. : Nash, D.	2022	Psychiatric comorbidity and psychosocial stressors among people initiating HIV care in Cameroon	PLoS ONE
Parcesepe, A. M. : Nash, D. : Tymejczyk, O. : Reidy, W. : Kulkarni, S. G. : Elul, B.	2020	Gender, HIV-Related Stigma, and Health-Related Quality of Life Among Adults Enrolling in HIV Care in Tanzania	AIDS Behav.
Parcesepe, A. : Tymejczyk, O. : Remien, R. : Gadisa, T. : Kulkarni, S. G. : Hoffman, S. : Melaku, Z. : Elul, B. : Nash, D.	2018	HIV-related stigma, social support, and psychological distress among individuals initiating ART in Ethiopia	AIDS Behav.
Park, H. J. : Sohng, K. Y. : Kim, S.	2014	Validation of the Korean version of the 39-Item Parkinson's disease questionnaire (PDQ-39)	Asian Nursing Research
Park, H. Y. : Jung, J. : Park, H. Y. : Lee, S. H. : Kim, E. S. : Kim, H. B. : Song, K. H.	2020	Psychological Consequences of Survivors of COVID-19 Pneumonia 1 Month after Discharge	Journal of Korean medical science
Park, S. G. : Bennett, M. E. : Couture, S. M. : Blanchard, J. J.	2013	Internalized stigma in schizophrenia: Relations with dysfunctional attitudes, symptoms, and quality of life	Psychiatry Res.
Parker, J. E. : Martinez, A. : Deutsch, G. K. : Prabhakar, V. : Lising, M. : Kapphahn, K. I. : Anidi, C. M. : Neuville, R. : Coburn, M. : Shah, N. : Bronte-Stewart, H. M.	2020	Safety of Plasma Infusions in Parkinson's Disease	Mov. Disord.
Parmar, Arpit : Gupta, Prashant : Bhad, Roshan	2022	An exploratory study of clinical profile, stigma and pathways to care among primary cannabis use disorder patients in India	Journal of Substance Use
Pascual-Sánchez, A. : Jenaro, C. : Montes, J. M.	2020	Performance of a measure to assess distress in bipolar disorder	Revista de Psiquiatria y Salud Mental
Patel, E. U. : Solomon, S. S. : Lucas, G. M. : McFall, A. M. : Tomori, C. : Srikrishnan, A. K. : Kumar, M. S. : Laeyendecker, O. : Celentano, D. D. : Thomas, D. L. : Quinn, T. C. : Mehta, S. H.	2021	Drug use stigma and its association with active hepatitis C virus infection and injection drug use behaviors among community-based people who inject drugs in India	Int. J. Drug Policy
Patra, B. N. : Patil, V. : Balhara, Y. P. S. : Khandelwal, S. K.	2022	Self-stigma in patients with major depressive disorder: An exploratory study from India	The International journal of social psychiatry
Patrick, D. L. : Edwards, T. C. : Skalicky, A. M. : Schick, B. : Topolski, T. D. : Kushalnagar, P. : Leng, M. : O'Neill-Kemp, A. M. : Sie, K.	2011	Validation of a quality-of-life measure for deaf or hard of hearing youth	Otolaryngol. Head Neck Surg.
Pawar, A. A. : Peters, A. : Rathod, J.	2014	Stigma of mental illness: A study in the Indian Armed Forces	Medical Journal Armed Forces India
Pearl, Rebecca L. : Forgeard, Marie J. C. : Rifkin, Lara : Beard, Courtney : Björgvinsson, Thröstur	2017	Internalized stigma of mental illness: Changes and associations with treatment outcomes	Stigma and Health
Pearl, R. L. : Wadden, T. A. : Hopkins, C. M. : Shaw, J. A. : Hayes, M. R. : Bakizada, Z. M. : Alfaris, N. : Chao, A. M. : Pinkasavage, E. : Berkowitz, R. I. : Alamuddin, N.	2017	Association between weight bias internalization and metabolic syndrome among treatment-seeking individuals with obesity	Obesity
Pearl, Rebecca L. : Wadden, Thomas A. : Chao, Ariana M. : Walsh, Olivia : Alamuddin, Naji : Berkowitz, Robert I. : Tronieri, Jena Shaw	2019	Weight Bias Internalization and Long-Term Weight Loss in Patients With Obesity	Annals of behavioral medicine : a publication of the Society of Behavioral Medicine
Pearson, C. A. : Johnson, M. O. : Neilands, T. B. : Dilworth, S. E. : Sauceda, J. A. : Mugavero, M. J. : Crane, H. M. : Fredericksen, R. J. : Mathews, W. C. : Moore, R. D. : Napravnik, S. : Mayer, K. H. : Christopoulos, K. A.	2021	Internalized HIV Stigma Predicts Suboptimal Retention in Care among People Living with HIV in the United States	AIDS Patient Care and STDs
Pearson, C. R. : Micek, M. A. : Pfeiffer, J. : Montoya, P. : Matediane, E. : Jonasse, T. : Cunguara, A. : Rao, D. : Gloyd, S. S.	2009	One year after ART initiation: Psychosocial factors associated with stigma among HIV-positive mozambicans	AIDS and Behavior
Peitzmeier, S. M. : Grosso, A. : Bowes, A. : Ceesay, N. : Baral, S. D.	2015	Associations of stigma with negative health outcomes for people living with HIV in the Gambia: Implications for key populations	Journal of Acquired Immune Deficiency Syndromes
Pellet, J. : Golay, P. : Nguyen, A. : Suter, C. : Ismailaj, A. : Bonsack, C. : Favrod, J.	2019	The relationship between self-stigma and depression among people with schizophrenia-spectrum disorders: A longitudinal study	Psychiatry Research
Peltzer, K. : Pengpid, S.	2019	Prevalence and associated factors of enacted, internalized and anticipated stigma among people living with HIV in South Africa: Results of the first national survey	HIV/AIDS - Research and Palliative Care
Peltzer, K. : Ramlagan, S.	2011	Perceived stigma among patients receiving antiretroviral therapy: a prospective study in KwaZulu-Natal, South Africa	AIDS Care
Peltzer, K. : Shikwane, M. E.	2011	Prevalence of postnatal depression and associated factors among HIV-positive women in primary care in Nkangala District, South Africa	Southern African Journal of HIV Medicine
Peng, Li : Dai, Yi	2020	Effect of metronidazole combined with autolytic debridement for the management of malignant wound malodor	The Journal of international medical research
Peng, W. : Liu, H. : Chen, J. : Zheng, Y. : Xu, X. : Tang, H. : Liu, Q.	2021	Development and validation of psychological status questionnaire for parents of infantile hemangiomas	Translational Pediatrics
Penn, T. M. : Overstreet, D. S. : Aroke, E. N. : Rumble, D. D. : Sims, A. M. : Kehrer, C. V. : Michl, A. N. : Hasan, F. N. : Quinn, T. L. : Long, D. L. : Trost, Z. : Morris, M. C. : Goodin, B. R.	2020	Perceived injustice helps explain the association between chronic pain stigma and movement-evoked pain in adults with nonspecific chronic low back pain	Pain Med.
Penwell-Waines, Lauren : Lewis, Kimberly : Valvano, Abbey : Smith, Suzanne : Rahn, Rebecca : Stepleman, Lara	2017	Testing the health promotion model for adherence and quality of life in individuals with multiple sclerosis	Psychology, Health & Medicine
Pérez-Miralles, F. : Prefasi, D. : García-Merino, A. : Ara, J. R. : Izquierdo, G. : Meca-Lallana, V. : Gascón, F. : Martínez-Ginés, M. L. : Ramió-Torrenta, L. : Costa-Frossard, L. : Fernández, Ó S. : Moreno-García, S. : Maurino, J. : Casanova-Estruch, B.	2019	Perception of stigma in patients with primary progressive multiple sclerosis	Multiple Sclerosis Journal - Experimental, Translational and Clinical
Pérez-Miralles, F. : Prefasi, D. : García-Merino, A. : Ara, J. R. : Izquierdo, G. : Meca-Lallana, V. : Gascón-Giménez, F. : Martínez-Ginés, M. L. : Ramió-Torrentà, L. : Costa-Frossard, L. : Fernández, Ó : Moreno-García, S. : Medrano, N. : Maurino, J. : Casanova, B.	2021	Short-term data on disease activity, cognition, mood, stigma and employment outcomes in a cohort of patients with primary progressive multiple sclerosis (UPPMS study)	Multiple Sclerosis and Related Disorders

Perlick, D. A. : Miklowitz, D. J. : Link, B. G. : Struening, E. : Kaczynski, R. : Gonzalez, J. : Manning, L. N. : Wolff, N. : Rosenheck, R. A.	2007	Perceived stigma and depression among caregivers of patients with bipolar disorder	Br. J. Psychiatry
Perlick, D. A. : Nelson, A. H. : Mattias, K. : Selzer, J. : Kalvin, C. : Wilber, C. H. : Huntington, B. : Holman, C. S. : Corrigan, P. W.	2011	In our own voice-family companion: Reducing self-stigma of family members of persons with serious mental illness	Psychiatric Services
Perlick, D. A. : Rosenheck, R. A. : Clarkin, J. F. : Sirey, J. A. : Salahi, J. : Struening, E. L. : Link, B. G.	2001	Adverse effects of perceived stigma on social adaptation of persons diagnosed with bipolar affective disorder	Psychiatric Services
Perlick, D. A. : Rosenheck, R. A. : Miklowitz, D. J. : Kaczynski, R. : Link, B. : Ketter, T. : Wisniewski, S. : Wolff, N. : Sachs, G.	2008	Caregiver burden and health in bipolar disorder: A cluster analytic approach	Journal of Nervous and Mental Disease
Perrott, Stephen B. : Murray, Alexander H. : Lowe, Janet : Mathieson, Cynthia M.	2000	The psychosocial impact of psoriasis: Physical severity, quality of life, and stigmatization	Physiology & Behavior
Pham, N. T. : Lee, J. J. : Pham, N. H. : Phan, T. D. Q. : Tran, K. : Dang, H. B. : Teo, I. : Malhotra, C. : Finkelstein, E. A. : Ozdemir, S.	2021	The prevalence of perceived stigma and self-blame and their associations with depression, emotional well-being and social well-being among advanced cancer patients: evidence from the APPROACH cross-sectional study in Vietnam	BMC Palliative Care
Phelan, S. M. : Bangerter, L. R. : Friedemann-Sanchez, G. : Lackore, K. A. : Morris, M. A. : Van Houtven, C. H. : Carlson, K. F. : van Ryn, M. : Harden, K. J. : Griffin, J. M.	2018	The Impact of Stigma on Community Reintegration of Veterans With Traumatic Brain Injury and the Well-Being of Their Caregivers	Archives of Physical Medicine and Rehabilitation
Phelan, S. M. : Bauer, K. W. : Bradley, D. : Bradley, S. M. : Haller, I. V. : Mundi, M. S. : Finney Rutten, L. J. : Schroeder, D. R. : Fischer, K. : Croghan, I.	2022	A model of weight-based stigma in health care and utilization outcomes: Evidence from the learning health systems network	Obesity Science and Practice
Picco, L. : Lau, Y. W. : Pang, S. : Abdin, E. : Vaingankar, J. A. : Chong, S. A. : Subramaniam, M.	2017	Mediating effects of self-stigma on the relationship between perceived stigma and psychosocial outcomes among psychiatric outpatients: Findings from a cross-sectional survey in Singapore	BMJ Open
Picco, L. : Pang, S. : Lau, Y. W. : Jeyagurunathan, A. : Satghare, P. : Abdin, E. : Vaingankar, J. A. : Lim, S. : Poh, C. L. : Chong, S. A. : Subramaniam, M.	2016	Internalized stigma among psychiatric outpatients: Associations with quality of life, functioning, hope and self-esteem	Psychiatry Research
Pichon, L. C. : Rossi, K. R. : Ogg, S. A. : Krull, L. J. : Griffin, D. Y.	2015	Social support, stigma and disclosure: Examining the relationship with HIV medication adherence among ryan white program clients in the mid-south USA	International Journal of Environmental Research and Public Health
Pięta, M. : Rzeszutek, M.	2022	Posttraumatic growth and posttraumatic depreciation among people living with HIV: the role of resilience and HIV/AIDS stigma in the person-centered approach	AIDS Care - Psychological and Socio-Medical Aspects of AIDS/HIV
Pijnenborg, G. H. M. : de Vos, A. E. : Timmerman, M. E. : Van der Gaag, M. : Sportel, B. E. : Arends, J. : Koopmans, E. M. : Van der Meer, L. : Aleman, A.	2019	Social cognitive group treatment for impaired insight in psychosis: A multicenter randomized controlled trial	Schizophrenia Research
Pirola, W. E. : Paiva, B. S. R. : Barroso, E. M. : Kissane, D. W. : Serrano, C. V. M. P. : Paiva, C. E.	2017	Translation and cultural adaptation of the Shame and Stigma Scale (SSS) into Portuguese (Brazil) to evaluate patients with head and neck cancer	Brazilian Journal of Otorhinolaryngology
Pirola, W. E. : Paiva, B. S. R. : de Oliveira, C. Z. : Lucchetti, G. : Lucchetti, A. L. G. : Kissane, D. : Paiva, C. E.	2020	Validation of the Brazilian version of the Shame and Stigma Scale (SSS-Br) for patients with head and neck cancers	Palliative & supportive care
Piskulic, D. : Addington, J. : Auther, A. : Cornblatt, B. A.	2011	Using the global functioning social and role scales in a first-episode sample	Early Intervent. Psychiatry
Pitchaimani, Govindharaj : Joydeepa, Darlong : John, A. S. : Suresh, Mani	2016	Children and adolescents' attitude towards having leprosy in a high endemic district of India	Leprosy Review
Pittet, V. : Vaucher, C. : Froehlich, F. : Burnand, B. : Michetti, P. : Maillard, M. H. : Anderegg, C. : Bauerfeind, P. : Beglinger, C. : Begré, S. : Belli, D. : Bengoa, J. M. : Biedermann, L. : Bigler, B. : Binek, J. : Blattmann, M. : Boehm, S. : Borovicka, J. : Braegger, C. P. : Brunner, N. : Bühr, P. : Burri, E. : Buyse, S. : Cremer, M. : Criblez, D. H. : De Saussure, P. : Degen, L. : Delarive, J. : Doerig, C. : Dora, B. : Dorta, G. : Egger, M. : Ehmann, T. : El-Wafa, A. : Engelmann, M. : Ezri, J. : Felley, C. : Fliegner, M. : Fournier, N. : Fraga, M. : Frei, P. : Frei, R. : Fried, M. : Funk, C. : Furlano, R. I. : Gallot-Lavallée, S. : Geyer, M. : Girardin, M. : Golay, D. : Grandinetti, T. : Gysi, B. : Haack, H. : Haarer, J. : Helbling, B. : Hengstler, P. : Herzog, D. : Hess, C. : Heyland, K. : Hinterleitner, T. : Hiroz, P. : Hirschi, C. : Hruz, P. : Iwata, R. : Jost, R. : Juillerat, P. : Brondolo, V. K. : Knellwolf, C. : Knoblauch, C. : Köhler, H. : Koller, R. : Krieger-Grübel, C. : Kullak-Ublick, G. : Künzler, P. : Landolt, M. : Lange, R. : Lehmann, F. S. : Macpherson, A. : Maerten, P. : Manser, C. : Manz, M. : Marbet, U. : Marx, G. : Matter, C. : McLin, V. : Meier, R. : Mendanova, M. : Meyenberger, C. : Misselwitz, B. : Moradpour, D. : Morell, B. : Mosler, P. : Mottet, C. : Müller, C. : Müller, P. : Müllhaupt, B. : Münger-Beyeler, C. : Musso, L. : Nagy, A. : Neagu, M. : Nichita, C. : Niess, J. : Noël, N. : Nydegger, A. : Obialo, N. : Oneta, C. : Oropesa, C. : Peter, U. : Peternac, D. : Petit, L. M. : Piccoli-Gfeller, F. : Pilz, J. B. : Raschle, N. : Rentsch, R. : Restellini, S. : Richterich, J. P. : Rihs, S. : Ritz, M. A. : Roduit, J. : Rogler, D. : Rogler, G. : Rossel, J. B. : Rueger, V. : Sagmeister, M. : Saner, G. : Sauter, B. : Sawatzki, M. : Schäppi, M. : Scharl, M. : Scharl, S. : Schelling, M. : Schibli, S. : Schlauri, H. : Uebelhart, S. S. : Schnegg, J. F. : Schoepfer, A. : Seibold, F. : Seirafi, M. : Semadeni, G. M. : Semela, D. : Senning, A. : Sidler, M. : Sokollik, C. : Spalinger, J. : Spangenberger, H. : Stadler, P. : Steuerwald, M. : Straumann, A. : Straumann-Funk, B. : Sulz, M. : Suter, A. : Thorens, J. : Tiedemann, S. : Tutuian, R. : Vavricka, S. : Viani, F. : Vögtlin, J. : Von Känel, R. : Vonlaufen, A. : Vouillamoz, D. : Vulliamy, R. : Wermuth, J. : Werner, H. : Wiesel, P. : Wiest, R. : Wylie, T. : Zeitz, J. : Zimmermann, D.	2017	Patient self-reported concerns in inflammatory bowel diseases: A genderspecific subjective quality-of-life indicator	PLoS ONE
Pollack, T. M. : Duong, H. T. : Nhat Vinh, D. T. : Phuong, D. T. : Thuy, D. H. : Nhung, V. T. T. : Uyen, N. K. : Linh, V. T. : Van Truong, N. : Le Ai, K. A. : Ninh, N. T. : Nguyen, A. : Canh, H. D. : Cosimi, L. A.	2022	A pretest-posttest design to assess the effectiveness of an intervention to reduce HIV-related stigma and discrimination in healthcare settings in Vietnam	Journal of the International AIDS Society
Polonsky, W. H. : Fisher, L. : Hessler, D. : Desai, U. : King, S. B. : Perez-Nieves, M.	2022	Toward a more comprehensive understanding of the emotional side of type 2 diabetes: A re-envisioning of the assessment of diabetes distress	Journal of Diabetes and its Complications
Poorkaveh, A. : Modabbernia, A. : Ashrafi, M. : Taslimi, S. : Karami, M. : Dalir, M. : Estakhri, A. : Malekzadeh, R. : Sharifi, H. P. : Poustchi, H.	2012	Validity, reliability and factor structure of hepatitis B quality of life questionnaire version 1.0: Findings in a large sample of 320 patients	Archives of Iranian Medicine
Porter, Kristen E. : Brennan-Ing, Mark : Burr, Jeffrey A. : Dugan, Elizabeth : Karpiak, Stephen E.	2017	Stigma and Psychological Well-being Among Older Adults With HIV: The Impact of Spirituality and Integrative Health Approaches	Gerontologist
Post, F. : Buchta, M. : Kemmler, G. : Pardeller, S. : Frajo-Apor, B. : Hofer, A.	2021	Resilience Predicts Self-Stigma and Stigma Resistance in Stabilized Patients With Bipolar I Disorder	Frontiers in Psychiatry
Post, F. : Pardeller, S. : Frajo-Apor, B. : Kemmler, G. : Sondermann, C. : Hausmann, A. : Fleischhacker, W. W. : Mizuno, Y. : Uchida, H. : Hofer, A.	2018	Quality of life in stabilized outpatients with bipolar I disorder: Associations with resilience, internalized stigma, and residual symptoms	Journal of Affective Disorders
Potter, L. P. : Mathias, S. D. : Raut, M. : Kianifard, F. : Landsman, A. : Tavakkol, A.	2007	The impact of aggressive debridement used as an adjunct therapy with terbinafine on perceptions of patients undergoing treatment for toenail onychomycosis	Journal of Dermatological Treatment
Pourmarzi, D. : Khoramirad, A. : Gaeeni, M.	2017	Perceived stigma in people living with HIV in Qom	Journal of Family and Reproductive Health
Power, J. : Amir, S. : Lea, T. : Brown, G. : Lyons, A. : Carman, M. : Rule, J. : Bourne, A.	2021	Bisexual Men Living with HIV: Wellbeing, Connectedness and the Impact of Stigma	AIDS Behav.
Pradhan, A. : Koirala, P. : Bhandari, S. S. : Dutta, S. : García-Grau, P. : Sampath, H. : Sharma, I.	2022	Internalized and Perceived Stigma and Depression in Pulmonary Tuberculosis: Do They Explain the Relationship Between Drug Sensitivity Status and Adherence?	Frontiers in Psychiatry
Prasko, J. : Ociskova, M. : Grambal, A. : Sigmundova, Z. : Kasalova, P. : Marackova, M. : Holubova, M. : Vrbova, K. : Latalova, K. : Slepecky, M.	2016	Personality features, dissociation, self-stigma, hope, and the complex treatment of depressive disorder	Neuropsychiatric Disease and Treatment
Price, Sarah N. : Shen, Megan : Rigney, Maureen : Ostroff, Jamie S. : Hamann, Heidi A.	2022	Identifying Barriers to Advocacy Among Patients With Lung Cancer: The Role of Stigma-Related Interpersonal Constraint	Oncology Nursing Forum
Prince, J. D. : Oyo, A. : Mora, O. : Wyka, K. : Schonebaum, A. D.	2018	Loneliness among Persons with Severe Mental Illness	J. Nerv. Ment. Dis.
Prior, N. : Remor, E. : Pérez-Fernández, E. : Caminoa, M. : Gómez-Traseira, C. : Gayá, F. : Aabom, A. : Aberer, W. : Betschel, S. : Boccon-Gibod, I. : Bouillet, L. : Bygum, A. : Csuka, D. : Farkas, H. : Gomide, M. : Grumach, A. : Leibovich, I. : Malbran, A. : Moldovan, D. : Mihaly, E. : Obtulowicz, K. : Perpén, C. : Peveling-Oberhag, A. : Porebski, G. : Chavannes, C. R. : Reshef, A. : Staubach, P. : Wiednig, M. : Caballero, T.	2016	Psychometric Field Study of Hereditary Angioedema Quality of Life Questionnaire for Adults: HAE-QoL	Journal of Allergy and Clinical Immunology: In Practice
Prouteau, Antoinette : Roux, Solenne : Destaillats, Jean-Marc : Bergua, Valérie	2017	Profiles of relationships between subjective and objective cognition in schizophrenia: Associations with quality of life, stigmatization, and mood factors	Journal of Cognitive Education and Psychology
Prus, N. : Grant, A. C.	2010	Patient beliefs about epilepsy and brain surgery in a multicultural urban population	Epilepsy and Behavior
Pruß, L. : Wiedl, K. H. : Waldorf, M.	2012	Stigma as a predictor of insight in schizophrenia	Psychiatry Research

Puia, I. C. : Fadgyas Stanculete, M. : Hopulele-Petri, A. : Muresan, D. : Puia, A.	2017	Patients' perception of Weight-Related stigma in a Romanian sample	J. Evid.-Based Psychother.
Pungrassami, P. : Kipp, A. M. : Stewart, P. W. : Chongsuvivatwong, V. : Strauss, R. P. : Van Rie, A.	2010	Tuberculosis and AIDS stigma among patients who delay seeking care for tuberculosis symptoms	International Journal of Tuberculosis and Lung Disease
Putera, A. M. : Irwanto, : Maramis, M. M.	2020	Quality-of-life (Qol) of indonesian children living with hiv: The role of caregiver stigma, burden of care, and coping	HIV/AIDS - Research and Palliative Care
Pyne, Jeffrey M. : Bean, Donna : Greer, Sullivan : Pyne, J. M. : Bean, D. : Sullivan, G.	2001	Characteristics of patients with schizophrenia who do not believe they are mentally ill	Journal of Nervous & Mental Disease
Qin, F. : Ye, X. : Wei, H. : Wen, Y. : Shi, L. : Zhen, L. : Zhi, M. : Zhang, L.	2019	Sexual Experience and Stigma Among Chinese Patients With an Enterostomy: A Cross-sectional, Descriptive Study	Wound management & prevention
Qin, Fang : Zhen, Li : Ye, Xinmei : Wei, Huiqiang : Zhu, Mulan : Chen, Jiali : Shi, Lei	2020	Stigma and Its Influence on Patients With Temporary Ostomy: A Cross-sectional Survey	Journal of wound, ostomy, and continence nursing : official publication of The Wound, Ostomy and Continence Nurses Society
Qin, Shouxue : Tan, Yanping : Lu, Bingyan : Cheng, Yuqing : Nong, Yanli	2018	Survey and analysis for impact factors of psychological distress in HIV-infected pregnant women who continue pregnancy	Journal of Maternal-Fetal & Neonatal Medicine
Qin, Y. : Dai, M. : Chen, L. : Zhang, T. : Zhou, N. : Chen, X.	2022	The relationship between ecological executive function and stigma among patients with epilepsy: The mediating effect of social support	Epilepsy Research
Qiu, L. : Tong, Y. : Lu, Z. : Gong, Y. : Yin, X.	2019	Depressive symptoms mediate the associations of stigma with medication adherence and quality of life in tuberculosis patients in China	American Journal of Tropical Medicine and Hygiene
Qiu, L. : Tong, Y. : Yang, Q. : Sun, N. : Gong, Y. : Yin, X.	2020	Reliability and validity of a smart quality of life scale for patients with tuberculosis	Journal of Public Health (Germany)
Quattrocchi, Annalisa : Barchitta, Martina : Nobile, Carmelo G. A. : Prato, Rosa : Sotgiu, Giovanni : Casuccio, Alessandra : Vitale, Francesco : Agodi, Antonella : Ccm Tb network	2018	Determinants of patient and health system delay among Italian and foreign-born patients with pulmonary tuberculosis: a multicentre cross-sectional study	BMJ open
Quenneville, A. F. : Badoud, D. : Nicastro, R. : Jermann, F. : Favre, S. : Kung, A. L. : Euler, S. : Perroud, N. : Richard-Lepouriel, H.	2020	Internalized stigmatization in borderline personality disorder and attention deficit hyperactivity disorder in comparison to bipolar disorder	Journal of Affective Disorders
Rabin, C. : O'Leary, A. : Neighbors, C. : Whitmore, K.	2000	Pain and depression experienced by women with interstitial cystitis	Women & Health
Radcliffe, Sheldon : Neaigus, Alan : Bernard, Marie Antoinette : Shepard, Colin	2015	HIV-related stigma in a New York City sample of adults in outpatient care for HIV infection: a short report	AIDS Care
Rael, C. T. : Davis, A.	2017	Depression and key associated factors in female sex workers and women living with HIV/AIDS in the Dominican Republic	International Journal of STD and AIDS
Rael, Christine Tagliaferri : Hampanda, Karen	2016	Understanding internalized HIV/AIDS-related stigmas in the Dominican Republic: a short report	AIDS Care
Rafael, F. : Houinato, D. : Nubukpo, P. : Dubreuil, C. M. : Si Tran, D. : Odermatt, P. : Clément, J. P. : Weiss, M. G. : Preux, P. M.	2010	Sociocultural and psychological features of perceived stigma reported by people with epilepsy in Benin	Epilepsia
Raguram, R. : Raghu, T. M. : Vounatsou, P. : Weiss, M. G.	2004	Schizophrenia and the cultural epidemiology of stigma in Bangalore, India	Journal of Nervous and Mental Disease
Raguram, R. : Weiss, M. G. : Channabasavanna, S. M. : Devins, G. M.	1996	Stigma, depression, and somatization in South India	American Journal of Psychiatry
Rajiah, K. : Maharajan, M. K. : Yeen, S. J. : Lew, S.	2017	Quality of Life and Caregivers' Burden of Parkinson's Disease	Neuroepidemiology
Ramos, J. V. : Mmbaga, B. T. : Turner, E. L. : Rugalabamu, L. L. : Luhanga, S. : Cunningham, C. K. : Dow, D. E.	2018	Modality of Primary HIV Disclosure and Association with Mental Health, Stigma, and Antiretroviral Therapy Adherence in Tanzanian Youth Living with HIV	AIDS Patient Care and STDs
Ranjan, L. K. : Gupta, P. R. : Srivastava, M.	2022	Perceived Stigma and Its Association with Stress, Anxiety, and Depression among Patients with Epilepsy	Journal of Nervous and Mental Disease
Rao, D. : Desmond, M. : Andrasik, M. : Rasberry, T. : Lambert, N. : Cohn, S. E. : Simoni, J.	2012	Feasibility, acceptability, and preliminary efficacy of the unity workshop: An internalized stigma reduction intervention for african american women living with hiv	AIDS Patient Care STDs
Rao, D. : Feldman, B. J. : Fredericksen, R. J. : Crane, P. K. : Simoni, J. M. : Kitahata, M. M. : Crane, H. M.	2012	A Structural equation model of HIV-related stigma, depressive symptoms, and medication adherence	AIDS Behav.
Rao, P. S. S. : Raju, M. S. : Barkataki, A. : Nanda, N. K. : Kumar, S.	2008	Extent and correlates of leprosy stigma in Rural India	Indian Journal of Leprosy
Rasoolinajad, M. : Abedinia, N. : Noorbala, A. A. : Mohraz, M. : Badie, B. M. : Hamad, A. : Sahebi, L.	2018	Relationship Among HIV-Related Stigma, Mental Health and Quality of life for HIV-Positive Patients in Tehran	AIDS and behavior
Rätsepp, Marju : Õun, Andre : Haldre, Sulev : Kaasik, Ain-Elmar	2000	Felt stigma and impact of epilepsy on employment status among Estonian people: Exploratory study	Seizure
Raves, Danielle M. : Brewis, Alexandra : Trainer, Sarah : Han, Seung-Yong : Wutich, Amber	2016	Bariatric Surgery Patients' Perceptions of Weight-Related Stigma in Healthcare Settings Impair Post-surgery Dietary Adherence	Frontiers in psychology
Raya, N. A. J. : Nilmanat, K.	2021	Experience and management of stigma among persons living with HIV in Bali, Indonesia: A descriptive study	Jpn. J. Nurs. Sci.
Rayan, Ahmad : Aldaieflih, Mo'tasem	2019	Public stigma toward mental illness and its correlates among patients diagnosed with schizophrenia	Contemporary Nurse: A Journal for the Australian Nursing Profession
Rayan, A. : Mahroum, M. H. : Khasawneh, A.	2018	The correlates of stigma toward mental illness among Jordanian patients with major depressive disorder	Perspectives in psychiatric care
Rayanakorn, A. : Ong-Artborirak, P. : Ademi, Z. : Chariyalertsak, S.	2022	Predictors of Stigma and Health-Related Quality of Life Among People Living with HIV in Northern Thailand	AIDS Patient Care and STDs
Razali, S. M. : Hussein, S. : Ismail, T. A. T.	2010	Perceived stigma and self-esteem among patients with schizophrenia	International Medical Journal
Razali, S. M. : Hariani, I.	2015	Caregiving experience and social support in patients with Schizophrenia	International Medical Journal
Regenauer, K. S. : Kleinman, M. B. : Belus, J. M. : Myers, B. : Joska, J. A. : Magidson, J. F.	2022	Effects of intersecting internalized stigmas and avoidance on HIV and alcohol-related outcomes among people living with HIV in South Africa	Drug Alcohol Depend.
Reginold, W. : Duff-Canning, S. : Meaney, C. : Armstrong, M. J. : Fox, S. : Rothberg, B. : Zadikoff, C. : Kennedy, N. : Gill, D. : Eslinger, P. : Marshall, F. : Mapstone, M. : Chou, K. L. : Persad, C. : Litvan, I. : Mast, B. : Tang-Wai, D. : Lang, A. E. : Marras, C.	2013	Impact of mild cognitive impairment on health-related quality of life in Parkinson's disease	Dementia and Geriatric Cognitive Disorders
Reid, K. : Herbert, A. : Baker, G. A.	2004	Epilepsy surgery: Patient-perceived long-term costs and benefits	Epilepsy and Behavior
Reif, S. : Cooper, H. : Wilson, E. : Brown, G. : Beckwith, N. : Ward, D.	2021	HIV stigma reduction through peer-led advocacy training	AIDS Education and Prevention
Reif, S. : Wilson, E. : McAllaster, C. : Pence, B.	2019	The Relationship of HIV-related Stigma and Health Care Outcomes in the US Deep South	AIDS Behav.
Reinius, M. : Rao, D. : Manhart, L. E. : Wiklander, M. : Svedhem, V. : Pryor, J. : Mayer, R. : Gaddist, B. : Kumar, S. : Mohanraj, R. : Jeyaseelan, L. : Wettergren, L. : Eriksson, L. E.	2018	Differential item functioning for items in Berger's HIV Stigma Scale: an analysis of cohorts from the Indian, Swedish, and US contexts	Quality of Life Research
Reinius, M. : Wettergren, L. : Wiklander, M. : Svedhem, V. : Ekström, A. M. : Eriksson, L. E.	2017	Development of a 12-item short version of the HIV stigma scale	Health and Quality of Life Outcomes
Relf, M. V. : Rollins, K. V.	2015	HIV-related Stigma Among an Urban Sample of Persons Living With HIV at Risk for Dropping Out of HIV-oriented Primary Medical Care	J. Assoc. Nurses AIDS Care
Remmert, J. E. : Convertino, A. D. : Roberts, S. R. : Godfrey, K. M. : Butryn, M. L.	2019	Stigmatizing weight experiences in health care: Associations with BMI and eating behaviours	Obesity Science and Practice
Remmert, Jocelyn E. : Mosery, Nzwakie : Goodman, Georgia : Bangsberg, David R. : Safren, Steven A. : Smit, Jennifer A. : Psaros, Christina	2020	Breastfeeding Practices Among Women Living with HIV in KwaZulu-Natal, South Africa: An Observational Study	Maternal & Child Health Journal
Ren, Z. : Wang, H. : Feng, B. : Gu, C. : Ma, Y. : Chen, H. : Li, B. : Liu, L.	2016	An exploratory cross-sectional study on the impact of education on perception of stigma by Chinese patients with schizophrenia	BMC health services research
Rendina, H. J. : Weaver, L. : Millar, B. M. : López-Matos, J. : Parsons, J. T.	2019	Psychosocial Well-Being and HIV-Related Immune Health Outcomes among HIV-Positive Older Adults: Support for a Biopsychosocial Model of HIV Stigma and Health	Journal of the International Association of Providers of AIDS Care
Reneses, B. : Sevilla Llewellyn-Jones, J. : Vila-Badia, R. : Palomo, T. : Lopez-Micó, C. : Pereira, M. : José Regatero, M. : Ochoa, S.	2020	The relationships between sociodemographic, psychosocial and clinical variables with personal-stigma in patients diagnosed with schizophrenia	Actas espanolas de psiquiatria
Restek-Petrović, B. : Bogović, A. : Grah, M. : Filipčić, I. : Ivezić, E.	2015	Personality characteristics of psychotic patients as possible motivating factors for participating in group psychotherapy	Psychiatria Danubina
Reynoso, S. F. : Dávalos, R. M. : García, R. R.	2011	Study of translation to Spanish and psychometric evaluation of a scale to measure the internalized stigma in patients with serious mental illness	Salud Ment.
Rice, W. S. : Crockett, K. B. : Mugavero, M. J. : Raper, J. L. : Atkins, G. C. : Turan, B.	2017	Association between internalized HIV-related stigma and HIV care visit adherence	Journal of Acquired Immune Deficiency Syndromes
Richards, H. L. : Fortune, D. G. : Griffiths, C. E. M. : Main, C. J.	2001	The contribution of perceptions of stigmatisation to disability in patients with psoriasis	J. Psychosom. Res.
Ridsdale, L. : McCrone, P. : Morgan, M. : Goldstein, L. : Seed, P. : Noble, A.	2013	Can an epilepsy nurse specialist-led self-management intervention reduce attendance at emergency departments and promote well-being for people with severe epilepsy? A non-randomised trial with a nested qualitative phase	
Ridsdale, L. : Wojewodka, G. : Robinson, E. J. : Noble, A. J. : Morgan, M. : Taylor, S. J. C. : McCrone, P. : Richardson, M. P. : Baker, G. : Landau, S. : Goldstein, L. H.	2018	The effectiveness of a group self-management education course for adults with poorly controlled epilepsy, SMILE (UK): A randomized controlled trial	Epilepsia
Rigney, M. : Rapsomaniki, E. : Carter-Harris, L. : King, J. C.	2021	A 10-Year Cross-Sectional Analysis of Public, Oncologist, and Patient Attitudes About Lung Cancer and Associated Stigma	Journal of Thoracic Oncology

Rinehart, Rachel : Rao, Deep : Amico, Rivet K. : Ruiz, Eduardo : Brandes, Peter : Correa, Cecilia : Pasalar, Siavash : Lama, Javier R. : Duerr, Ann : Molina, Yamile	2019	Experienced HIV-Related Stigma and Psychological Distress in Peruvian Sexual and Gender Minorities: A Longitudinal Study to Explore Mediating Roles of Internalized HIV-Related Stigma and Coping Styles	AIDS & Behavior
Ritsher, J. B. : Otilingam, P. G. : Grajales, M.	2003	Internalized stigma of mental illness: Psychometric properties of a new measure	Psychiatry Research
Ritsher, J. B. : Phelan, J. C.	2004	Internalized stigma predicts erosion of morale among psychiatric outpatients	Psychiatry Research
Robson, C. : Myers, L. : Pretorius, C. : Lian, O. S. : Reuber, M.	2018	Health related quality of life of people with non-epileptic seizures: The role of socio-demographic characteristics and stigma	Seizure
Rodak, J. : Witusik, A. : Nowakowska-Domagała, K. : Pietras, T. : Mokros, Ł	2018	Psychopathological profile and antipsychotic treatment may be linked to internalised stigma in schizophrenia - A cross-sectional study	Postepy Psychiatrii i Neurologii
Rodriguez, V. J. : Cook, R. R. : Peltzer, K. : Jones, D. L.	2017	Prevalence and psychosocial correlates of suicidal ideation among pregnant women living with HIV in Mpumalanga Province, South Africa	AIDS Care - Psychological and Socio-Medical Aspects of AIDS/HIV
Rodriguez, V. J. : Sued, O. : Cecchini, D. : Mandell, L. N. : Bofill, L. M. : Weiss, S. M. : Cassetti, I. : Cahn, P. : Jones, D. L.	2018	Suicidality among nonadherent patients living with HIV in Buenos Aires, Argentina: Prevalence and correlates	AIDS Care - Psychological and Socio-Medical Aspects of AIDS/HIV
Rodríguez-Márquez, I. : Montes, F. : Upegui-Arango, L. D. : Montoya, N. : Vargas, N. E. : Rojas, A. : Valencia, G. C. : Álvarez, C. M. : Marceló-Díaz, C. : Ochoa, J.	2022	Measurement of stigma and associated characteristics in people with tuberculosis in Medellín, Colombia: A cross-sectional study	Transactions of the Royal Society of Tropical Medicine and Hygiene
Rolston, A. M. : Gardner, M. : Vilain, E. : Sandberg, D. E.	2015	Parental reports of stigma associated with child's disorder of sex development	Intl. J. Endocrinol.
Rongkavilit, C. : Wright, K. : Chen, X. : Naar-King, S. : Chuenyam, T. : Phanuphak, P.	2010	HIV stigma, disclosure and psychosocial distress among Thai youth living with HIV	International Journal of STD and AIDS
Rood, J. E. : Schultz, J. R. : Rausch, J. R. : Modi, A. C.	2014	Examining perceived stigma of children with newly-diagnosed epilepsy and their caregivers over a two-year period	Epilepsy Behav.
Roosenschoon, B. J. : van Weeghel, J. : Deen, M. L. : van Esveld, E. W. : Kamperman, A. M. : Mulder, C. L.	2021	Effects of Illness Management and Recovery: A Multicenter Randomized Controlled Trial	Frontiers in Psychiatry
Rose, Shiho : Boyes, Allison : Kelly, Brian : Cox, Martine : Palazzi, Kerrin : Paul, Christine	2018	Help-seeking behaviour in newly diagnosed lung cancer patients: Assessing the role of perceived stigma	Psycho-Oncology
Rose, S. : Boyes, A. : Kelly, B. : Cox, M. : Palazzi, K. : Paul, C.	2021	Lung cancer stigma is a predictor for psychological distress: A longitudinal study. Lung cancer stigma is a predictor for psychological distress	Psycho-Oncology
Rose, Shiho : Kelly, Brian : Boyes, Allison : Cox, Martine : Palazzi, Kerrin : Paul, Christine	2018	Impact of Perceived Stigma in People Newly Diagnosed With Lung Cancer: A Cross-Sectional Analysis	Oncology Nursing Forum
Rosen, D. : Greenberg, D. : Schmeidler, J. : Shefler, G.	2008	Stigma of mental illness, religious change, and explanatory models of mental illness among Jewish patients at a mental-health clinic in North Jerusalem	Ment. Health Relig. Cult.
Rosen, J. G. : Phiri, L. : Chibuye, M. : Namukonda, E. S. : Mbizvo, M. T. : Kayeyi, N.	2021	Integrated psychosocial, economic strengthening, and clinical service-delivery to improve health and resilience of adolescents living with HIV and their caregivers: Findings from a prospective cohort study in Zambia	PLoS ONE
Rossi, A. A. : Manzoni, G. M. : Pietrabissa, G. : Di Pauli, D. : Mannarini, S. : Castelnuovo, G.	2022	Weight stigma in patients with overweight and obesity: validation of the Italian Weight Self-Stigma Questionnaire (WSSQ)	Eating and Weight Disorders
Rossi, A. : Galderisi, S. : Rocca, P. : Bertolino, A. : Mucci, A. : Rucci, P. : Gibertoni, D. : Aguglia, E. : Amore, M. : Andriola, I. : Bellomo, A. : Biondi, M. : Callista, G. : Comparelli, A. : Dell'Osso, L. : Di Giannantonio, M. : Fagiolini, A. : Marchesi, C. : Monteleone, P. : Montemagni, C. : Niolu, C. : Piegari, G. : Pinna, F. : Roncone, R. : Stratta, P. : Tenconi, E. : Vita, A. : Zeppegno, P. : Maj, M. : Mancini, M. : Nettis, M. A. : Rizzo, G. : Porcelli, S. : Deste, G. : Galluzzo, A. : Gheda, L. : Carpiniello, B. : Ghiani, A. : Lai, A. : Cannavò, D. : Minutolo, G. : Signorelli, M. S. : Acciavatti, T. : Alessandrini, M. : Vellante, F. : Cantisani, A. : Altamura, M. : Padalino, F. A. : Pagano, T. : Belvedere Murri, M. : Calcagno, P. : Corso, A. : D'Onofrio, S. : Marucci, C. : Santarelli, V. : Bianchini, V. : Giusti, L. : Malavolta, M. : Bucci, P. : Chieffi, M. : De Simone, S. : Merlotti, E. : Rocco, M. : Vignapiano, A. : Tomasetti, C. : Feggi, A. : Gattoni, E. : Gramaglia, C. : Cremonese, C. : Collantoni, E. : Gallicchio, D. : De Panfilis, C. : Ossola, P. : Tonna, M. : Carmassi, C. : Gesi, C. : Rutigliano, G. : Brugnoli, R. : Corigliano, V. : De Carolis, A. : Di Fabio, F. : Mirigliani Torti, A. M. C. : Di Lorenzo, G. : Siracusano, A. : Troisi, A. : Bartoli, L. : Corrivetti, G. : Diasco, F. : Bolognesi, S. : Borghini, E. : Goracci, A. : Frieri, T. : Mingrone, C. : Sigaudo, M. : Italian Network for Research on, Psychoses	2017	The relationships of personal resources with symptom severity and psychosocial functioning in persons with schizophrenia: results from the Italian Network for Research on Psychoses study	Eur. Arch. Psychiatry Clin. Neurosci.
Rossi, S. L. : Sereda, Y. : Luoma, J. B. : Pavlov, N. : Toussova, O. : Vasileva, J. : Abramova, K. : Bendiks, S. : Kiriazova, T. : Vetrova, M. : Blokhina, E. : Krupitsky, E. : Lioznov, D. : Lodi, S. : Lunze, K.	2021	Addressing intersectional stigma as a care barrier for HIV-positive people who inject drugs: Design of an RCT in St. Petersburg, Russia	Contemporary Clinical Trials Communications
Rubtsova, Anna A. : Wingood, Gina : Ofotokun, Ighovwerha : Mehta, C. Christina : Gustafson, Deborah : Vance, David E. : Sharma, Anjali : Adimora, Adaora A. : Holstad, Marcia	2021	Psychosocial Mechanisms of Self-rated Successful Aging with HIV: A Structural Equation Model	AIDS & Behavior
Rukundo, G. Z. : Mpango, R. S. : Ssembajjwe, W. : Gadow, K. D. : Patel, V. : Kinyanda, E.	2020	Prevalence and risk factors for youth suicidality among perinatally infected youths living with HIV/AIDS in Uganda: the CHAKA study	Child and Adolescent Psychiatry and Mental Health
Rusch, L. C. : Kanter, J. W. : Manos, R. C. : Weeks, C. E.	2008	Depression stigma in a predominantly low income african american sample with elevated depressive symptoms	J. Nerv. Ment. Dis.
Rüsch, N. : Nordt, C. : Kawohl, W. : Brantschen, E. : Bärtsch, B. : Müller, M. : Corrigan, P. W. : Rössler, W.	2014	Work-related discrimination and change in self-stigma among people with mental illness during supported employment	Psychiatr. Serv.
Rüsch, N. : Schiel, S. : Corrigan, P. W. : Leihener, F. : Jacob, G. A. : Olschewski, M. : Lieb, K. : Bohus, M.	2008	Predictors of dropout from inpatient dialectical behavior therapy among women with borderline personality disorder	J. Behav. Ther. Exp. Psychiatry
Russinova, Z. : Rogers, E. S. : Gagne, C. : Bloch, P. : Drake, K. M. : Mueser, K. T.	2014	A randomized controlled trial of a peer-run antistigma photovoice intervention	Psychiatr. Serv.
Rydström, L. L. : Wiklander, M. : Ygge, B. M. : Navér, L. : Eriksson, L. E.	2015	Legal guardians understand how children with the human immunodeficiency virus perceive quality of life and stigma	Acta Paediatrica, International Journal of Paediatrics
Rydström, L. L. : Wiklander, M. : Navér, L. : Ygge, B. M. : Eriksson, L. E.	2016	HIV-related stigma and health-related quality of life among children living with HIV in Sweden	AIDS Care - Psychological and Socio-Medical Aspects of AIDS/HIV
Rzhevskaya, N. K. : Ruzhenkov, V. A. : Ruzhenkova, V. V. : Retyunsky, K. Y. : Minakova, J. S.	2020	Attitude to diseases and social distance of persons with mental disorders	Intern. J. Early Child. Spec. Educ.
Sabanciogullari, S. : Dogan, S.	2016	Internalized stigma among inpatients with mental illness in Turkey and factors affecting it	Kuwait Medical Journal
Sadeghian, E. : Rostami, P. : Shamsaei, F. : Tapak, L.	2019	The effect of counseling on stigma in psychiatric patients receiving electroconvulsive therapy: A clinical trial study	Neuropsychiatric Disease and Treatment
Sadighi, G. : Khodaei, M. R. : Fadai, F. : Mirabzadeh, A. : Sadighi, A.	2015	Self stigma among people with Bipolar-I disorder in Iran	Iran. Rehabil. J.
Saffari, M. : Koenig, H. G. : O'Garo, K. N. : Pakpour, A. H.	2018	Mediating effect of spiritual coping strategies and family stigma stress on caregiving burden and mental health in caregivers of persons with dementia	Dementia
Saffari, Mohsen : Lin, Chung-Ying : Koenig, Harold G. : O'Garo, Keisha-Gaye N. : Broström, Anders : Pakpour, Amir H.	2019	A Persian version of the Affiliate Stigma Scale in caregivers of people with dementia	Health Promotion Perspectives
Sahu, Ajaykumar C. : Akhade, Kiran S.	2022	A cross-sectional study on stigma and discrimination confronted by HIV positive patients in the economic capital of India	Journal of family medicine and primary care
Saine, M. E. : Szymczak, J. E. : Moore, T. M. : Bamford, L. P. : Barg, F. K. : Schnittker, J. : Holmes, J. H. : Mitra, N. : Lo Re, V.	2020	Determinants of stigma among patients with hepatitis C virus infection	Journal of Viral Hepatitis
Saine, M. E. : Szymczak, J. E. : Moore, T. M. : Bamford, L. P. : Barg, F. K. : Forde, K. A. : Schnittker, J. : Holmes, J. H. : Mitra, N. : Lo Re, V.	2021	The impact of disease-related knowledge on perceptions of stigma among patients with Hepatitis C Virus (HCV) infection	PLoS ONE
Sajatovic, M. : Tatsuoka, C. : Welter, E. : Perzynski, A. T. : Colon-Zimmermann, K. : Van Doren, J. R. : Bukach, A. : Lawless, M. E. : Ryan, E. R. : Sturniolo, K. : Lhatoo, S.	2016	Targeted Self-Management of Epilepsy and Mental Illness for individuals with epilepsy and psychiatric comorbidity	Epilepsy Behav.
Saleem, S. : Tariq, S. : Tariq, S. : Irfan, S. : Javed, F.	2023	Factors leading to delayed and challenging presentation of benign breast lumps in young females	Pakistan Journal of Medical Sciences
Salgado, P. C. B. : Fernandes, P. T. : Noronha, A. L. A. : Barbosa, F. D. : Souza, E. A. P. : Li, L. M.	2005	The second step in the construction of a stigma scale of epilepsy	Arquivos de Neuro-Psiquiatria
Salih, M. H. : Mekonnen, H. : Derseh, L. : Lindgren, H. : Erlandsson, K.	2022	Anticipated stigma and associated factors among chronic illness patients in Amhara Region Referral Hospitals, Ethiopia: A multicenter cross-sectional study	PLoS ONE

Salih, M. H. : Wettergren, L. : Lindgren, H. : Erlandsson, K. : Mekonen, H. : Derseh, L.	2022	Translation and psychometric evaluation of chronic illness anticipated stigma scale (CIASS) among patients in Ethiopia	PLoS ONE
Salter, K. A. : Prior, K. N. : Bond, M. J.	2017	Predicting wellbeing among people with epilepsy using illness cognitions	Epilepsy Behav.
Salters, K. A. : Irick, M. : Anema, A. : Zhang, W. : Parashar, S. : Patterson, T. L. : Chen, Y. : Somers, J. : Montaner, J. S. G. : Hogg, R. S.	2017	Harder-to-reach people living with HIV experiencing high prevalence of all-type mental health disorder diagnosis	AIDS Care - Psychological and Socio-Medical Aspects of AIDS/HIV
Sánchez, J. : Sung, C. : Phillips, B. N. : Tschopp, M. K. : Muller, V. : Lee, H. L. : Chan, F.	2019	Predictors of perceived social effectiveness of individuals with serious mental illness	Psychiatric rehabilitation journal
Sanchez, K. : Killian, M. O. : Eghaneyan, B. H. : Cabassa, L. J. : Trivedi, M. H.	2019	Culturally adapted depression education and engagement in treatment among Hispanics in primary care: Outcomes from a pilot feasibility study	BMC Fam. Pract.
Sangaramoorthy, T. : Jamison, A. M. : Dyer, T. V.	2017	HIV Stigma, Retention in Care, and Adherence Among Older Black Women Living With HIV	J. Assoc. Nurses AIDS Care
Sarfraz, M. : Waqas, H. : Ahmed, S. : Rurush-Asencio, R. : Mushtaque, I.	2022	Cancer-Related Stigmatization, Quality of Life, and Fear of Death Among Newly Diagnosed Cancer Patients	Omega
Sarısoy, Gökhan : Kaçar, Ömer Faruk : Pazvantoğlu, Ozan : Korkmaz, Işil Zabun : Öztürk, Arif : Akkaya, Derya : Yılmaz, Sercan : Böke, Ömer : Sahin, Ahmet Rifat	2013	Internalized stigma and intimate relations in bipolar and schizophrenic patients: A comparative study	Comprehensive Psychiatry
Sarkar, S. : Balhara, Y. P. S. : Kumar, S. : Saini, V. : Kamran, A. : Patil, V. : Singh, S. : Gyawali, S.	2019	Internalized stigma among patients with substance use disorders at a tertiary care center in India	Journal of Ethnicity in Substance Abuse
Saurabh, Yakhmi : Sidhu, B. S. : Baldeep, Kaur : Dalla, E. K.	2014	Study of HIV related stigma in people living with HIV/AIDS (PLHA): role of gender differences	Indian Journal of Scientific Research
Sayed, T. A. : Ali, M. M. : Hadad, S.	2021	Risk factors and impact of stigma on psychiatric patients in Sohag	Egyptian Journal of Neurology, Psychiatry and Neurosurgery
Sayles, J. N. : Hays, R. D. : Sarkisian, C. A. : Mahajan, A. P. : Spritzer, K. L. : Cunningham, W. E.	2008	Development and psychometric assessment of a multidimensional measure of internalized HIV stigma in a sample of HIV-positive adults	AIDS Behav.
Sayles, J. N. : Wong, M. D. : Kinsler, J. J. : Martins, D. : Cunningham, W. E.	2009	The association of stigma with self-reported access to medical care and antiretroviral therapy adherence in persons living with HIV/AIDS	J. Gen. Intern. Med.
Schäfer, A. : Scheurlen, M. : Felten, M. : Kraus, M. R.	2005	Physician-patient relationship and disclosure behaviour in chronic hepatitis C in a group of German outpatients	European Journal of Gastroenterology and Hepatology
Scheerder, G. : Van Den Eynde, S. : Reyntiens, P. : Koeck, R. : Deblonde, J. : Ddungu, C. : Florence, E. : Joosten, C. : Van Wijngaerden, E. : Dewaele, A.	2021	Quality of life in people living with HIV: An exploratory cross-sectional survey in Belgium	AIDS Education and Prevention
Schensul, Stephen L. : Ha, Toan : Schensul, Jean J. : Grady, James : Burleson, Joseph A. : Gaikwad, Sushma : Joshi, Kavita : Malye, Rupal : Sarna, Avina	2021	Multilevel and Multifactorial Interventions to Reduce Alcohol Consumption and Improve ART Adherence and Related Factors Among HIV Positive Men in Mumbai, India	AIDS & Behavior
Schick, B. : Skalicky, A. : Edwards, T. : Kushalnagar, P. : Topolski, T. : Patrick, D.	2013	School placement and perceived quality of life in youth who are deaf or hard of hearing	Journal of Deaf Studies and Deaf Education
Schlüter, D. K. : Tennant, A. : Mills, R. : Diggle, P. J. : Young, C. A.	2018	Risk factors for social withdrawal in amyotrophic lateral sclerosis/motor neurone disease	Amyotrophic Lateral Sclerosis and Frontotemporal Degeneration
Schmid-Ott, G. : Jaeger, B. : Kuensebeck, H. W. : Ott, R. : Lamprecht, F.	1996	Dimensions of stigmatization in patients with psoriasis in a 'Questionnaire on Experience with Skin Complaints'	Dermatology
Schmid-Ott, G. : Kuensebeck, H. W. : Jaeger, B. : Werfel, T. : Frahm, K. : Ruitman, J. : Kapp, A. : Lamprecht, F.	1999	Validity study for the stigmatization experience in atopic dermatitis and psoriatic patients	Acta Derm.-Venereol.
Schmid-Ott, G. : Künsebeck, H. W. : Jäger, B. : Sittig, U. : Hofste, N. : Ott, R. : Malewski, P. : Lamprecht, F.	2005	Significance of the stigmatization experience of psoriasis patients: A 1-year follow-up of the illness and its psychosocial consequences in men and women	Acta Derm.-Venereol.
Schmid-Ott, G. : Künsebeck, H. W. : Jecht, E. : Shimshoni, R. : Lazaroff, I. : Schallmayer, S. : Calliess, I. T. : Malewski, P. : Lamprecht, F. : Götz, A.	2007	Stigmatization experience, coping and sense of coherence in vitiligo patients	Journal of the European Academy of Dermatology and Venereology
Schneider, L. : Stevens, J. : Husain, A. M. : Ito, D. : Macfadden, W. : Fuller, D. S. : Zee, P.	2022	Impairment in Functioning and Quality of Life in Patients With Idiopathic Hypersomnia: the Real World Idiopathic Hypersomnia Outcomes Study	Neurology
Schnell, T. : Kehring, A. : Moritz, S. : Morgenroth, O.	2021	Patients responses to diagnoses of mental disorders: Development and validation of a reliable self-report measure	International journal of methods in psychiatric research
Schofield, Casey A. : Ponzini, Gabriella T.	2020	The Skidmore Anxiety Stigma Scale (SASS): A covert and brief self-report measure	Journal of anxiety disorders
Schomerus, G. : Corrigan, P. W. : Klauer, T. : Kuwert, P. : Freyberger, H. J. : Lucht, M.	2011	Self-stigma in alcohol dependence: Consequences for drinking-refusal self-efficacy	Drug and Alcohol Dependence
Schrag, A. : Hovris, A. : Morley, D. : Quinn, N. : Jahanshahi, M.	2003	Young- versus older-onset Parkinson's disease: Impact of disease and psychosocial consequences	Movement Disorders
Schroyen, S. : Missotten, P. : Jerusalem, G. : Van den Akker, M. : Buntinx, F. : Adam, S.	2017	Association between self-perception of aging, view of cancer and health of older patients in oncology: A one-year longitudinal study	BMC Cancer
Scott, W. : Yu, L. : Patel, S. : McCracken, L. M.	2019	Measuring Stigma in Chronic Pain: Preliminary Investigation of Instrument Psychometrics, Correlates, and Magnitude of Change in a Prospective Cohort Attending Interdisciplinary Treatment	J. Pain
Seb-Akahomen, Omonefe Joy : Lawani, Ambrose Onivefu : James, Bawo Onesirosan	2019	Stigma and suicidality among people living with HIV attending a secondary healthcare facility in Nigeria	Perspectives in Psychiatric Care
Sebring, K. : Shattuck, J. : Berk, J. : Boersma, I. : Sillau, S. : Kluger, B. M.	2018	Assessing the validity of proxy caregiver reporting for potential palliative care outcome measures in Parkinson's disease	Palliative Medicine
Sedić, B. : Štrkalj Ivezić, S. : Petrak, O. : Ilić, B.	2021	Differences in Resilience, Self-Stigma and Mental Health Recovery between Patients with Schizophrenia and Depression	Psychiatria Danubina
Sedláčková, Z. : Kamarádová, D. : Praško, J. : Látalová, K. : Ocisková, M. : Cinculová, A. : Kubínek, R. : Mainerová, B. : Ticháčková, A. : Vrbová, K.	2015	Treatment adherence and self-stigma in patients with depressive disorder in remission - A cross-sectional study	Neuroendocrinology Letters
Segalovich, J. : Doron, A. : Behrbalk, P. : Kurs, R. : Romem, P. : Stolovi, T.	2015	Internalization of stigma and self-esteem as it affects the capacity for intimacy among patients with schizophrenia, comparison between Jews and Arabs	European Psychiatry
Sehlo, M. G. : Bahlas, S. M.	2013	Perceived illness stigma is associated with depression in female patients with systemic lupus erythematosus	Journal of Psychosomatic Research
Seid, J. : Mebrahtu, K.	2022	Prevalence and associated factors of depression among people with epilepsy in Ethiopia: a cross-sectional study	Egypt. J. Neurol., Psychiatr. Neurosurg.
Seid, S. : Abdu, O. : Mitiku, M. : Tamirat, K. S.	2020	Prevalence of depression and associated factors among HIV/AIDS patients attending antiretroviral therapy clinic at Dessie referral hospital, South Wollo, Ethiopia	International Journal of Mental Health Systems
Selohilwe, O. : Bhana, A. : Garman, E. C. : Petersen, I.	2019	Evaluating the role of levels of exposure to a task shared depression counselling intervention led by behavioural health counsellors: Outcome and process evaluation	Int. J. Ment. Health Syst.
Sematlane, N. P. : Knight, L. : Masquillier, C. : Wouters, E.	2022	A cross-cultural adaptation and validation of a scale to assess illness identity in adults living with a chronic illness in South Africa: a case of HIV	AIDS Research and Therapy
Sen, M. S. : Nehra, R. : Grover, S.	2020	Social cognition in patients with first episode of psychosis in remission	Indian Journal of Psychiatry
Seng, E. K. : Shapiro, R. E. : Buse, D. C. : Robbins, M. S. : Lipton, R. B. : Parker, A.	2022	The unique role of stigma in migraine-related disability and quality of life	Headache
Seo, J. G. : Kim, J. M. : Park, S. P.	2015	Perceived stigma is a critical factor for interictal aggression in people with epilepsy	Seizure
Seo, K. : Song, Y.	2021	Development and validation of the self-stigma scale in people with diabetes	Nursing open
Sereda, Y. : Kiriazova, T. : Makarenko, O. : Carroll, J. J. : Rybak, N. : Chybisov, A. : Bendiks, S. : Idrisov, B. : Dutta, A. : Gillani, F. S. : Samet, J. H. : Flanigan, T. : Lunze, K.	2020	Stigma and quality of co-located care for HIV-positive people in addiction treatment in Ukraine: a cross-sectional study	Journal of the International AIDS Society
Servin, A. E. : Muñoz, F. A. : Strathdee, S. A. : Kozo, J. : Zúñiga, M. L.	2012	Choosing sides: HIV health care practices among shared populations of HIV-positive latinos living near the US-Mexico Border	J. Int. Assoc. Phys. AIDS
Sevenoaks, T. : Fouche, J. P. : Phillips, N. : Heany, S. : Myer, L. : Zar, H. J. : Stein, D. J. : Hoare, J.	2022	Childhood Trauma and Mental Health in the Cape Town Adolescent Antiretroviral Cohort	J. Child Adolesc. Trauma
Shacham, Enbal : Rosenburg, Neal : Onen, Nur F. : Donovan, Michael F. : Overton, E. Turner	2015	Persistent HIV-related stigma among an outpatient US clinic population	International journal of STD & AIDS
Shah, B. B. : Nieweglowski, K. : Corrigan, P. W.	2022	Perceptions of difference and disdain on the self-stigma of mental illness	J. Ment. Health
Shah, I. : Khalily, M. T. : Ahmad, I. : Hallahan, B.	2019	Impact of Conventional Beliefs and Social Stigma on Attitude Towards Access to Mental Health Services in Pakistan	Community mental health journal
Shah, K. : McMahon, J. M. : Trabold, N. : Aidala, A. A. : Chen, M. : Pouget, E. R. : Simmons, J. : Klostermann, K.	2015	Determinants of physical and global functioning in adult HIV-positive heterosexual men	AIDS Care - Psychological and Socio-Medical Aspects of AIDS/HIV
Shakibazadeh, E. : Bartholomew, L. K. : Rashidian, A. : Larijani, B.	2016	Persian Diabetes Self-Management Education (PDSME) program: evaluation of effectiveness in Iran	Health promotion international
Shamsaei, F. : Holtforth, M. G.	2020	Development and Psychometric Testing of the Stigma Assessment Tool for Family Caregivers of People with Mental Illness	East Asian Archives of Psychiatry
Shamsalinia, A. : Masoudi, R. : Rad, R. E. : Ghaffari, F.	2019	Development and psychometric evaluation of the Perceived Social Stigma Questionnaire (PSSQ-for adults with epilepsy): A mixed method study	Epilepsy and Behavior

Sharaf, Amira Y. : Ossman, Laila H. : Lachine, Ola A.	2012	A cross-sectional study of the relationships between illness insight, internalized stigma, and suicide risk in individuals with schizophrenia	International Journal of Nursing Studies
Shen, M. J. : Hamann, H. A. : Thomas, A. J. : Ostroff, J. S.	2016	Association between patient-provider communication and lung cancer stigma	Supportive Care in Cancer
Sher, I. : McGinn, L. : Sirey, J. A. : Meyers, B.	2005	Effects of caregivers' perceived stigma and causal beliefs on patients' adherence to antidepressant treatment	Psychiatric Services
Shi-Jie, F. : Hong-Mei, G. : Li, W. : Bin-Hong, W. : Yi-Ru, F. : Gang, W. : Tian-Mei, S.	2017	Perceptions of stigma and its correlates among patients with major depressive disorder: A multicenter survey from China	Asia-Pacific Psychiatry
Shim, S. : Kang, D. : Bae, K. R. : Lee, W. Y. : Nam, S. J. : Sohn, T. S. : Jeong, B. C. : Sinn, D. H. : Kweon, S. S. : Shim, Y. M. : Cho, J.	2021	Association between cancer stigma and job loss among cancer survivors	Psycho-Oncology
Shimotsu, S. : Horikawa, N. : Emura, R. : Ishikawa, S. I. : Nagao, A. : Ogata, A. : Hiejima, S. : Hosomi, J.	2014	Effectiveness of group cognitive-behavioral therapy in reducing self-stigma in Japanese psychiatric patients	Asian Journal of Psychiatry
Shimotsu, S. : Horikawa, N.	2016	Self-stigma in depressive patients: Association of cognitive schemata, depression, and self-esteem	Asian Journal of Psychiatry
Shin, Sanghyuk S. : Yadav, Kartik : Nyamathi, Adeline M. : Carpenter, Catherine L. : Shah, Saanchi V. : Ekstrand, Maria L. : Ramakrishnan, Padma : Sinha, Sanjeev : Pamujula, Suresh	2018	Household Food Insecurity as Mediator of the Association Between Internalized Stigma and Opportunistic Infections	AIDS & Behavior
Shin, Y. J. : Joo, Y. H. : Kim, J. H.	2016	Self-perceived cognitive deficits and their relationship with internalized stigma and quality of life in patients with schizophrenia	Neuropsychiatric Disease and Treatment
Shin, Y. J. : Oh, E. G.	2021	Factors Influencing Resilience among Korean adolescents and young adult survivors of childhood cancer	European journal of oncology nursing : the official journal of European Oncology Nursing Society
Shittu, R. O. : Issa, B. A. : Olanrewaju, G. T. : Odeigah, L. O. : Sule, A. G. : Sanni, M. A. : Aderibigbe, S. A.	2014	Adherence to highly active antiretroviral therapy, in depressed peoples living with HIV/AIDS in Nigeria, West Africa	Journal of Antivirals and Antiretrovirals
Shokoohi, Mostafa : Bauer, Greta R. : Kaida, Angela : Logie, Carmen H. : Lacombe-Duncan, Ashley : Milloy, M. -J : Lloyd-Smith, Elisa : Carter, Allison : Loutfy, Mona	2019	Patterns of social determinants of health associated with drug use among women living with HIV in Canada: a latent class analysis	Addiction
Shrestha, R. : Altice, F. L. : Copenhaver, M. M.	2019	HIV-Related stigma, motivation to adhere to antiretroviral therapy, and medication adherence among HIV-positive methadone-maintained patients	Journal of Acquired Immune Deficiency Syndromes
Shrestha, S.	2019	Internalized Stigma, Coping and Social Support with Mental Illness in Manipal Teaching Hospital, Pokhara, Nepal	Journal of Nepal Health Research Council
Shrestha, S. : Shibanuma, A. : Poudel, K. C. : Nanishi, K. : Koyama Abe, M. : Shakya, S. K. : Jimba, M.	2019	Perceived social support, coping, and stigma on the quality of life of people living with HIV in Nepal: a moderated mediation analysis	AIDS Care - Psychological and Socio-Medical Aspects of AIDS/HIV
Shumet, S. : Wondie, M. : Ayano, G. : Asfaw, H. : Kassew, T. : Mesafint, G.	2022	Antiepileptic Drug Adherence and Its Associated Factors among Epilepsy Patients on Follow-ups at Amanuel Mental Specialized Hospital, Ethiopia	Ethiopian journal of health sciences
Shumet, S. : W/michele, B. : Angaw, D. : Ergete, T. : Alemnew, N.	2021	Magnitude of internalised stigma and associated factors among people with bipolar disorder at Amanuel Mental Specialized Hospital, Addis Ababa, Ethiopia: A cross-sectional study	BMJ Open
Sianturi, Elfride I. : Perwitasari, Dyah A. : Soltief, Sitti N. : Atiqul Islam, Md : Geboers, Bas : Taxis, Katja	2021	Health literacy of people living with HIV in a rural area in Indonesia: A cross-sectional study	Health & Social Care in the Community
Sibitz, I. : Amering, M. : Unger, A. : Seyringer, M. E. : Bachmann, A. : Schrank, B. : Benesch, T. : Schulze, B. : Woppmann, A.	2011	The impact of the social network, stigma and empowerment on the quality of life in patients with schizophrenia	Eur. Psychiatry
Sibitz, I. : Provaznikova, K. : Lipp, M. : Lakeman, R. : Amering, M.	2013	The impact of recovery-oriented day clinic treatment on internalized stigma: Preliminary report	Psychiatry Research
Sibitz, I. : Unger, A. : Woppmann, A. : Zidek, T. : Amering, M.	2011	Stigma resistance in patients with schizophrenia	Schizophrenia Bulletin
Siderowf, A. : Jaggi, J. L. : Xie, S. X. : Loveland-Jones, C. : Leng, L. : Hurtig, H. : Colcher, A. : Stern, M. : Chou, K. L. : Liang, G. : Maccarone, H. : Simuni, T. : Baltuch, G.	2006	Long-term effects of bilateral subthalamic nucleus stimulation on health-related quality of life in advanced Parkinson's disease	Movement Disorders
Siefried, Krista J. : Mao, Limin : Kerr, Stephen : Cysique, Lucette A. : Gates, Thomas M. : McAllister, John : Maynard, Anthony : de Wit, John : Carr, Andrew : Paart study investigators	2017	Socioeconomic factors explain suboptimal adherence to antiretroviral therapy among HIV-infected Australian adults with viral suppression	PloS one
Silva, C. : Soares, M. J. : Madeira, N. : Rosendo, I. : Miranda, A. F. : Pereira, A. T. : Araújo, A. : Cabaços, C. : Macedo, A.	2022	Portuguese Version of the Stigma Scale: Preliminary Psychometric Characteristics	Acta Medica Portuguesa
Silverman, Michael J.	2013	Effects of music therapy on self- and experienced stigma in patients on an acute care psychiatric unit: a randomized three group effectiveness study	Archives of psychiatric nursing
Singer, E.	1976	Sociopsychological factors influencing response to levodopa therapy for Parkinson's disease	Archives of Physical Medicine and Rehabilitation
Singh, A. : Mattoo, S. K. : Grover, S.	2016	Stigma and its correlates in patients with schizophrenia attending a general hospital psychiatric unit	Indian Journal of Psychiatry
Singh, R. J. : Sarna, A. : Schensul, J. J. : Mahapatra, B. : Ha, T. : Schensul, S. L.	2020	A multilevel intervention to reduce stigma among alcohol consuming men living with HIV receiving antiretroviral therapy: findings from a randomized control trial in India	AIDS (London, England)
Singh, Swarndeep : Kumar, Saurabh : Sarkar, Siddharth : Balhara, Yatan Pal Singh	2018	Quality of Life and its Relationship with Perceived Stigma among Opioid Use Disorder Patients: An Exploratory Study	Indian journal of psychological medicine
Singla, N. : Avasthi, A. : Grover, S.	2020	Recovery and its correlates in patients with schizophrenia	Asian Journal of Psychiatry
Sinha, A. : Kushwaha, A. S. : Kotwal, A. : Sanghi, S. : Verma, A. K.	2010	Stigma in leprosy: Miles to go!	Indian Journal of Leprosy
Sirey, J. A. : Bruce, M. L. : Alexopoulos, G. S. : Perlick, D. A. : Friedman, S. J. : Meyers, B. S.	2001	Perceived stigma and patient-rated severity of illness as predictors of antidepressant drug adherence	Psychiatric Services
Sirey, J. A. : Bruce, M. L. : Alexopoulos, G. S. : Perlick, D. A. : Raue, P. : Friedman, S. J. : Meyers, B. S.	2001	Perceived stigma as a predictor of treatment discontinuation in young and older outpatients with depression	American Journal of Psychiatry
Sivakumar, T. : Basavarajappa, C. : Philip, M. : Kumar, C. N. : Thirthalli, J. : Parthasarathy, R.	2022	Impact of incentivizing ASHAs on the outcome of persons with severe mental illness in a rural South Indian community amidst the COVID-19 pandemic	Asian journal of psychiatry
Slater, Larry Z. : Moneyham, Linda : Vance, David E. : Raper, James L. : Mugavero, Michael J. : Childs, Gwendolyn	2015	The Multiple Stigma Experience and Quality of Life in Older Gay Men With HIV	JANAC: Journal of the Association of Nurses in AIDS Care
Small, L. A. : Huynh, A. K. : Parchment, T. M.	2022	The association between self-Esteem, stigma, and mental health among South African youth living with HIV: the need for integrated HIV care services	AIDS Care - Psychological and Socio-Medical Aspects of AIDS/HIV
Smilowitz, S. : Aftab, A. : Aebi, M. : Levin, J. : Tatsuoka, C. : Sajatovic, M.	2020	Age-Related Differences in Medication Adherence, Symptoms, and Stigma in Poorly Adherent Adults With Bipolar Disorder	J. Geriatr. Psychiatry Neurol.
Smith, Dylan M. : Loewenstein, George : Rozin, Paul : Sherriff, Ryan L. : Ubel, Peter A.	2007	Sensitivity to disgust, stigma, and adjustment to life with a colostomy	Journal of Research in Personality
Smith Fawzi, Mary C. : Eustache, Eddy : Oswald, Catherine : Louis, Ermaze : Surkan, Pamela J. : Scanlan, Fiona : Hook, Sarah : Mancuso, Anna : Mukherjee, Joia S.	2012	Psychosocial support intervention for HIV-affected families in Haiti: Implications for programs and policies for orphans and vulnerable children	Social Science & Medicine
Smith, J. : Ayre, J. : Jansen, J. : Cvejic, E. : McCaffery, K. J. : Doust, J. : Copp, T.	2021	Impact of diagnostic labels and causal explanations for weight gain on diet intentions, cognitions and emotions: An experimental online study	Appetite
Smith, Meghan L. : Yang, Lawrence H. : Huang, Debbie : Pike, Kathleen M. : Yuan, Chengmei : Wang, Zhen	2018	Measuring internalized stigma of mental illness among Chinese outpatients with mood disorders	International Journal of Culture & Mental Health
Snyder, S. : Kroll, J. L. : Chen, A. B. : Antonoff, M. B. : Yang, C. C. : Milbury, K.	2022	Moderators of the association between stigma and psychological and cancer-related symptoms in women with non-small cell lung cancer	Psycho-Oncology
So, H. S. : Chae, M. J. : Kim, H. Y.	2017	Reliability and Validity of the Korean Version of the Cancer Stigma Scale	Journal of Korean Academy of Nursing
Sokrab, M. : Sokrab, A. : Elzubeir, M.	2012	Quality of life in people with epilepsy in Sudan: An example of underserved communities in developing countries	Qatar Med. J.
Soliman, M. M.	2020	Feeling of stigmatization and satisfaction with life among Arabic psoriatic patients	Saudi Pharmaceutical Journal
Soltan, E. M. : Salama, H. M. : Aboelmagd, M. A.	2018	Assessment of stigma among patients infected with hepatitis C virus in Suez City, Egypt	Journal of Public Health (Germany)
Sommer, R. : Augustin, M. : Hilbring, C. : Ständer, S. : Hubo, M. : Hutt, H. J. : von Stülpnagel, C. C. : da Silva, N.	2021	Significance of chronic pruritus for intrapersonal burden and interpersonal experiences of stigmatization and sexuality in patients with psoriasis	J. Eur. Acad. Dermatol. Venereol.
Song, B. : Yan, C. : Lin, Y. : Wang, F. : Wang, L.	2016	Health-related quality of life in HIV-infected men who have sex with men in china: A cross-sectional study	Med. Sci. Monit.
Song, Hayeon : Shin, Hochang : Kim, Youngmi	2015	Perceived stigma of alcohol dependency: comparative influence on patients and family members	Journal of Substance Use
Song, W. : Guo, X. : Chen, K. : Chen, X. : Cao, B. : Wei, Q. : Huang, R. : Zhao, B. : Wu, Y. : Shang, H. F.	2014	The impact of non-motor symptoms on the Health-Related Quality of Life of Parkinson's disease patients from Southwest China	Parkinsonism and Related Disorders
Sorsdahl, K. R. : Mall, S. : Stein, D. J. : Joska, J. A.	2011	The prevalence and predictors of stigma amongst people living with HIV/AIDS in the Western Province	AIDS Care - Psychological and Socio-Medical Aspects of AIDS/HIV
Spencer, Lauren A. : Silverman, Arielle M. : Cook, Jonathan E.	2019	Adapting to Multiple Sclerosis Stigma Across the Life Span	International Journal of MS Care

Spiegel, B. M. R. : Bolus, R. : Han, S. : Tong, M. : Esrailian, E. : Talley, J. : Tran, T. : Smith, J. : Karsan, H. A. : Durazo, F. : Bacon, B. : Martin, P. : Younossi, Z. : Hwa-Ong, S. : Kanwal, F.	2007	Development and validation of a disease-targeted quality of life instrument in chronic hepatitis B: The hepatitis B quality of life instrument, version 1.0	Hepatology
Spielman, K. L. : Soler-Hampejsek, E. : Muula, A. S. : Tenthani, L. : Hewett, P. C.	2021	Depressive symptoms, HIV-related stigma and ART adherence among caregivers of children in vulnerable households in rural southern Malawi	PLoS ONE
Spooner, Catherine : Jayasinghe, Upali W. : Faruqi, Nighat : Stocks, Nigel : Harris, Mark F.	2018	Predictors of weight stigma experienced by middle-older aged, general-practice patients with obesity in disadvantaged areas of Australia: a cross-sectional study	BMC Public Health
Sprague, Laurel : Simon, Sara : Sprague, Courtenay	2011	Employment discrimination and HIV stigma: survey results from civil society organisations and people living with HIV in Africa	African Journal of AIDS Research (AJAR)
Squiers, Linda : Siddiqui, Mariam : Kataria, Ishu : Dhillon, Preet K. : Aggarwal, Aastha : Bann, Carla : Lynch, Molly : Nyblade, Laura	2021	Perceived, Experienced, and Internalized Cancer Stigma: Perspectives of Cancer Patients and Caregivers in India	RTI Press
Sripad, P. : Arnoff, E. : Warren, C. : Tripathi, V.	2021	Measuring barriers to fistula care: investigating composite measures for targeted fistula programming in Nigeria and Uganda	BMC Women's Health
Stangl, A. L. : Lilleston, P. : Mathema, H. : Pliakas, T. : Krishnaratne, S. : Sievwright, K. : Bell-Mandla, N. : Vermaak, R. : Mainga, T. : Steinhaus, M. : Donnell, D. : Schaap, A. : Bock, P. : Ayles, H. : Hayes, R. : Hoddinott, G. : Bond, V. : Hargreaves, J. R.	2019	Development of parallel measures to assess HIV stigma and discrimination among people living with HIV, community members and health workers in the HPTN 071 (PopART) trial in Zambia and South Africa	Journal of the International AIDS Society
Stangl, A. L. : Mwale, M. : Sebany, M. : Mackworth-Young, C. R. S. : Chiiya, C. : Chonta, M. : Clay, S. : Sievwright, K. : Bond, V.	2021	Feasibility, Acceptability and Preliminary Efficacy of Tikambisane ('Let's Talk to Each Other'): A Pilot Support Group Intervention for Adolescent Girls Living With HIV in Zambia	Journal of the International Association of Providers of AIDS Care
Staring, A. B. P. : Ter Huurne, M. A. B. : Van Der Gaag, M.	2013	Cognitive Behavioral Therapy for negative symptoms (CBT-n) in psychotic disorders: A pilot study	J. Behav. Ther. Exp. Psychiatry
Staring, A. B. P. : Van der Gaag, M. : Van den Berge, M. : Duivenvoorden, H. J. : Mulder, C. L.	2009	Stigma moderates the associations of insight with depressed mood, low self-esteem, and low quality of life in patients with schizophrenia spectrum disorders	Schizophrenia Research
Stepleman, L. M. : Floyd, R. M. : Valvano-Kelley, A. : Penwell-Waines, L. : Wonn, S. : Crethers, D. : Rahn, R. : Smith, S.	2017	Developing a measure to assess identity reconstruction in patients with multiple sclerosis	Rehabilitation Psychology
Stevens, Serena D. : Thompson, Nicolas R. : Sullivan, Amy B.	2019	Prevalence and Correlates of Body Image Dissatisfaction in Patients with Multiple Sclerosis...Annual Meeting of the Consortium of Multiple Sclerosis Centers (CMSC); May 30-June 2, 2018, Nashville, TN, USA	International Journal of MS Care
Stewart, C. J. : Lysaker, P. H. : Davis, L. W.	2013	Relationships of social-sexual function with stigma and narrative quality among persons with schizophrenia spectrum disorders over one year	Am. J. Psychiatr. Rehabil.
Stip, E. : Caron, J. : Tousignant, M. : Lecomte, Y.	2017	Suicidal Ideation and Schizophrenia: Contribution of Appraisal, Stigmatization, and Cognition	Canadian Journal of Psychiatry
Stjernman, H. : Tysk, C. : Almer, S. : Ström, M. : Hjortswang, H.	2010	Worries and concerns in a large unselected cohort of patients with Crohn's disease	Scandinavian Journal of Gastroenterology
Stockton, M. A. : Mughal, A. Y. : Bui, Q. : Greene, M. C. : Pence, B. W. : Go, V. : Gaynes, B. N.	2021	Psychometric performance of the perceived stigma of substance abuse scale (PSAS) among patients on methadone maintenance therapy in Vietnam	Drug and Alcohol Dependence
Stolzenburg, S. : Freitag, S. : Evans-Lacko, S. : Muehlan, H. : Schmidt, S. : Schomerus, G.	2017	The stigma of mental illness as a barrier to self labeling as having a mental illness	J. Nerv. Ment. Dis.
Stolzenburg, S. : Freitag, S. : Schmidt, S. : Schomerus, G.	2018	Associations between causal attributions and personal stigmatizing attitudes in untreated persons with current mental health problems	Psychiatry Res.
Stolzenburg, Susanne : Tessmer, Claudia : Corrigan, Patrick W. : Böttge, Matthias : Freitag, Simone : Schäfer, Ingo : Freyberger, Harald J. : Schomerus, Georg	2018	Childhood trauma and self-stigma of alcohol dependence: Applying the progressive model of self-stigma	Stigma and Health
Storholm, Erik David : Halkitis, Perry N. : Kupprat, Sandra A. : Hampton, Melvin C. : Palamar, Joseph J. : Brennan-Ing, Mark : Karpiak, Stephen	2013	HIV-Related Stigma as a Mediator of the Relation Between Multiple-Minority Status and Mental Health Burden in an Aging HIV-Positive Population	Journal of HIV/AIDS & Social Services
Straits-Tröster, K. : Fields, J. A. : Wilkinson, S. B. : Pahwa, R. : Lyons, K. E. : Koller, W. C. : Tröster, A. I.	2000	Health-related quality of life in Parkinson's disease after pallidotomy and deep brain stimulation	Brain and Cognition
Strother, P. J. : Tipayamongkholgul, M. : Kosaisevee, V. : Suwannapong, N.	2022	Effects of psychosocial factors on nonadherence to ART in Ganta, Nimba county, Liberia	AIDS Research and Therapy
Subedi, B. : Timilsina, B. D. : Tamrakar, N.	2019	Perceived stigma among people living with HIV/AIDS in Pokhara, Nepal	HIV/AIDS - Research and Palliative Care
Subedi, S. : Shyangwa, P. M. : Shakya, D. R. : Pandey, A. K. : Sapkota, N.	2014	Knowledge, attitude and stigma in epilepsy: a study from a tertiary care centre in Nepal	Health Renaissance
Subramanian, T. : Gupte, M. D. : Dorairaj, V. S. : Periannan, V. : Mathai, A. K.	2009	Psycho-social impact and quality of life of people living with HIV/AIDS in South India	AIDS Care - Psychological and Socio-Medical Aspects of AIDS/HIV
Suhail, Kausar : Chaudhry, Haroon R.	2004	Impact of perceived fears and stigma on psychosocial problems in patients with epilepsy	Pakistan Journal of Social and Clinical Psychology
Sulaiman, A. A. : Bushara, S. O. E. : Elmadhoun, W. M. Y. : Noor, S. K. M.	2013	Characteristics and perspectives of newly diagnosed sputum smear positive tuberculous patients under DOTS strategy in River Nile State - Sudan	Sudanese Journal of Public Health
Sum, M. Y. : Chan, S. K. W. : Tse, S. : Bola, J. R. : Chen, E. Y. H.	2021	Internalized stigma as an independent predictor of employment status in patients with schizophrenia	Psychiatric rehabilitation journal
Sum, M. Y. : Chan, S. K. W. : Tse, S. : Bola, J. R. : Ng, R. M. K. : Hui, C. L. M. : Lee, E. H. M. : Chang, W. C. : Chen, E. Y. H.	2022	Elucidating the relationship between internalized stigma, cognitive insight, illness severity, and functioning in patients with schizophrenia using a path analysis approach	J. Ment. Health
Suman, Aarzoo : Nehra, Ritu : Sahoo, Swapnajeet : Grover, Sandeep	2022	Prevalence of loneliness and its correlates among patients with schizophrenia	The International journal of social psychiatry
Sumari-de Boer, I. M. : Sprangers, M. A. : Prins, J. M. : Nieuwkerk, P. T.	2012	HIV stigma and depressive symptoms are related to adherence and virological response to antiretroviral treatment among immigrant and indigenous HIV infected patients	AIDS and behavior
Sumari-de Boer, M. : Nellen, J. F. B. : Sprangers, M. A. G. : Prins, J. M. : Nieuwkerk, P. T.	2012	Personalized Stigma and Disclosure Concerns Among HIV-Infected Immigrant and Indigenous HIV-Infected Persons in the Netherlands	J. HIV/AIDS Soc. Serv.
Sun, L. : Liu, X. : Weng, X. : Deng, H. : Li, Q. : Liu, J. : Luan, X.	2022	Narrative therapy to relieve stigma in oral cancer patients: A randomized controlled trial	International journal of nursing practice
Sun, Y. : Chen, G. : Wang, L. : Li, N. : Srisurapanont, M. : Hong, J. P. : Hatim, A. : Chen, C. H. : Udomratn, P. : Bae, J. N. : Fang, Y. R. : Chua, H. C. : Liu, S. I. : George, T. : Bautista, D. : Chan, E. : John Rush, A. : Yang, H. : Su, Y. A. : Si, T. M.	2019	Perception of stigma and its associated factors among patients with major depressive disorder: A multicenter survey from an Asian population	Frontiers in Psychiatry
Sung, C. : Chan, F. : Ditchman, N. : Chan, C.	2020	Evaluating the World Health Organization's International Classification of Functioning, Disability, and Health (ICF) framework as an employment model for people with epilepsy	Journal of Vocational Rehabilitation
Surmann, M. : Falke, S. : von Gruchalla, L. : Maisch, B. : Uhlmann, C. : Arolt, V. : Lencer, R.	2021	Understanding the multidimensional phenomenon of medication adherence attitudes in psychosis	Psychiatry Res.
Sutar, R. : Lahiri, A. : Singh, G. : Chaudhary, S.	2022	Development and Validation of Structured COVID Perception Interview Guide (COPING) for Assessing the Acute Impact of COVID-19 Diagnosis	Journal of Neurosciences in Rural Practice
Suvirya, S. : Shukla, M. : Pathania, S. : Banerjee, G. : Kumar, A. : Tripathi, A.	2018	Stigma associated with sexually transmitted infections among patients attending suraksha clinic at a tertiary care hospital in northern India	Indian Journal of Dermatology
Suzukamo, Y. : Ohbu, S. : Kondo, T. : Kohmoto, J. : Fukuhara, S.	2006	Psychological adjustment has a greater effect on health-related quality of life than on severity of disease in Parkinson's disease	Movement Disorders
Swanson, Robert M. : Spitzer, Stephen P.	1970	Stigma and the psychiatric patient career	Journal of Health and Social Behavior
Swendeman, D. : Fehrenbacher, A. E. : Roy, S. : Das, R. : Ray, P. : Sumstine, S. : Ghose, T. : Jana, S.	2018	Gender disparities in depression severity and coping among people living with HIV/AIDS in Kolkata, India	PLoS ONE
Świtaj, P. : Anczewska, M. : Chrostek, A. : Grygiel, P. : Prot-Klinger, K. : Choma, M. : Wciórka, J.	2013	The sense of coherence as a predictor of the scope and impact of stigma experiences among people with mental illness	Psychiatry Res.
Świtaj, P. : Grygiel, P. : Wciórka, J. : Humenny, G. : Anczewska, M.	2013	The Stigma subscale of the Consumer Experiences of Stigma Questionnaire (CESQ): A psychometric evaluation in Polish psychiatric patients	Comprehensive Psychiatry
Świtaj, Piotr : Grygiel, Paweł : Anczewska, Marta : Wciórka, Jacek	2014	Loneliness mediates the relationship between internalised stigma and depression among patients with psychotic disorders	International Journal of Social Psychiatry
Świtaj, Piotr : Grygiel, Paweł : Chrostek, Anna : Nowak, Izabela : Wciórka, Jacek : Anczewska, Marta : Świtaj, Piotr : Grygiel, Paweł : Wciórka, Jacek	2017	The relationship between internalized stigma and quality of life among people with mental illness: are self-esteem and sense of coherence sequential mediators?	Quality of Life Research
Świtaj, P. : Wciórka, J. : Smolarska-Świtaj, J. : Grygiel, P.	2009	Extent and predictors of stigma experienced by patients with schizophrenia	European Psychiatry
Sylla, M. : Vogel, A. C. : Bah, A. K. : Tassiou, N. R. : Barry, S. D. : Djibo, B. A. : Toure, M. L. : Foksona, S. : Konate, M. : Cisse, F. A. : Mateen, F. J.	2020	Prevalence, severity, and associations of depression in people with epilepsy in Guinea: A single-center study	Epilepsy and Behavior
Szcześniak, D. : Kobyłko, A. : Wojciechowska, I. : Kłapciński, M. : Rymaszewska, J.	2018	Internalized stigma and its correlates among patients with severe mental illness	Neuropsychiatric Disease and Treatment

Szczesniak, Dorota : Kobylko, Agnieszka : Lenart, Marta : Karczewski, Maciej : Cyran, Agnieszka : Musial, Piotr : Rymaszewska, Joanna	2021	Personality Factors Crucial in Internalized Stigma Understanding in Psychiatry	Healthcare (Basel, Switzerland)
Taft, T. H. : Ballou, S. : Keefer, L.	2013	A preliminary evaluation of internalized stigma and stigma resistance in inflammatory bowel disease	Journal of health psychology
Taft, T. H. : Keefer, L. : Leonhard, C. : Nealon-Woods, M.	2009	Impact of perceived stigma on inflammatory bowel disease patient outcomes	Inflammatory Bowel Diseases
Taft, T. H. : Keefer, L. : Artz, C. : Bratten, J. : Jones, M. P.	2011	Perceptions of illness stigma in patients with inflammatory bowel disease and irritable bowel syndrome	Quality of life research : an international journal of quality of life aspects of treatment, care and rehabilitation
Taft, T. H. : Riehl, M. E. : Dowjotas, K. L. : Keefer, L.	2014	Moving beyond perceptions: Internalized stigma in the irritable bowel syndrome	Neurogastroenterology and Motility
Takada, S. : Ettner, S. L. : Harawa, N. T. : Garland, W. H. : Shoptaw, S. J. : Cunningham, W. E.	2020	Life Chaos is Associated with Reduced HIV Testing, Engagement in Care, and ART Adherence Among Cisgender Men and Transgender Women upon Entry into Jail	AIDS Behav.
Takada, S. : Nyakato, V. : Nishi, A. : O'Malley, A. J. : Kakuhikire, B. : Perkins, J. M. : Bangsberg, D. R. : Christakis, N. A. : Tsai, A. C.	2019	The social network context of HIV stigma: Population-based, sociocentric network study in rural Uganda	Social Science and Medicine
Tan, Chuxia : Zhong, Chenxi : Mei, Ranran : Yang, Ronghong : Wang, Dangdang : Deng, Xianjiao : Chen, Shihao : Ye, Man	2022	Stigma and related influencing factors in postoperative oral cancer patients in China: a cross-sectional study	Supportive care in cancer : official journal of the Multinational Association of Supportive Care in Cancer
Tan, M. M. J. : Lim, E. C. : Nadkarni, N. V. : Lye, W. K. : Tan, E. K. : Kumar, M. P.	2019	The characteristics of patients associated with high caregiver burden in Parkinson's disease in Singapore	Front. Neurol.
Tan, Sharon Ch : Yeoh, Ai Lean : Choo, Irene Bk : Huang, Adeline Ph : Ong, Seng Hong : Ismail, Hafizah : Ang, Pei Pei : Chan, Yiong Huak	2012	Burden and coping strategies experienced by caregivers of persons with schizophrenia in the community	Journal of Clinical Nursing (John Wiley & Sons, Inc.)
Tanaka, N. : Hamamoto, Y. : Kurotobi, Y. : Yamazaki, Y. : Nakatani, S. : Matsubara, M. : Haraguchi, T. : Yamaguchi, Y. : Izumi, K. : Fujita, Y. : Kuwata, H. : Hyo, T. : Yanase, M. : Matsuda, M. : Negoro, S. : Higashiyama, H. : Yamada, Y. : Kurose, T. : Seino, Y.	2022	Stigma evaluation for diabetes and other chronic non-communicable disease patients: Development, validation and clinical use of stigma scale - The Kanden Institute Stigma Scale	Journal of Diabetes Investigation
Taneja, N. : Chellaiyan, V. G. : Daral, S. : Adhikary, M. : Das, T. K.	2017	Home based care as an approach to improve the efficiency of treatment for MDR tuberculosis: A quasi-experimental pilot study	Journal of Clinical and Diagnostic Research
Tang, I. C. : Wu, H. C.	2012	Quality of life and self-stigma in individuals with schizophrenia	Psychiatric Quarterly
Tang, Q. : Yang, S. : Liu, C. : Li, L. : Chen, X. : Wu, F. : Huang, X.	2021	Effects of Mindfulness-Based Cognitive Therapy on Stigma in Female Patients With Schizophrenia	Frontiers in Psychiatry
Tanney, M. R. : Naar-King, S. : MacDonnel, K.	2012	Depression and Stigma in High-Risk Youth Living With HIV: A Multi-Site Study	J. Pediatr. Health Care
Tanriverdi, D. : Kaplan, V. : Bilgin, S. : Demir, H.	2020	The comparison of internalized stigmatization levels of patients with different mental disorders	Journal of Substance Use
Tantik Pak, A. : Sengul, H. S. : Sengul, Y. : Mail Gurkan, Z.	2021	Social phobia and its relationship with perceived epilepsy-associated stigma IN patients with epilepsy	Epilepsy and Behavior
Tareke, M. : Belete, T. : Ergetie, T. : Tadesse, M. : Menberu, M. : Ketemaw, A.	2022	Triple Burden of Neurocognitive Impairment and Co-occurring Depression and Anxiety Among People Living With HIV in Bahir Dar, Ethiopia: A Multicenter Study	Frontiers in Psychiatry
Tedrus, G. M. A. S. : Sterca, G. S. : Pereira, R. B.	2017	Physical activity, stigma, and quality of life in patients with epilepsy	Epilepsy and Behavior
Tegegne, M. T. : Mossie, T. B. : Awoke, A. A. : Assaye, A. M. : Gebrie, B. T. : Eshetu, D. A.	2015	Depression and anxiety disorder among epileptic people at Amanuel Specialized Mental Hospital, Addis Ababa, Ethiopia	BMC Psychiatry
Temel, A. B. : Bozkurt, S. : Senol, Y. : Alpsoy, E.	2019	Internalized stigma in patients with Acne Vulgaris, Vitiligo, and alopecia areata	Turkish Journal of Dermatology
Temesgen, W. A. : Chien, W. T. : Valimaki, M. A. : Bressington, D.	2020	Predictors of subjective recovery from recent-onset psychosis in a developing country: a mixed-methods study	Social psychiatry and psychiatric epidemiology
Teo, I. : Ozdemir, S. : Malhotra, C. : Yang, G. M. : Ocampo, R. R. : Bhatnagar, S. : Hapuarachchi, T. : Joad, A. K. : Mariam, L. : Palat, G. : Rahman, R. : Finkelstein, E. A.	2021	High anxiety and depression scores and mental health service use among South Asian advanced cancer patients: A multi-country study	Journal of Pain and Symptom Management
Tesfaw, G. : Ayano, G. : Awoke, T. : Assefa, D. : Birhanu, Z. : Miheretie, G. : Abebe, G.	2016	Prevalence and correlates of depression and anxiety among patients with HIV on-follow up at Alert Hospital, Addis Ababa, Ethiopia	BMC Psychiatry
Tesfay, A. : Gebremariam, A. : Gerbaba, M. : Abrha, H.	2015	Gender differences in health related quality of life among people living with HIV on highly active antiretroviral therapy in mekelle town, northern ethiopia	BioMed Res. Int.
Tesfaye, Elias : Kassaw, Chalachew : Agenagnew, Liyew	2020	Stigma Resistance and Its Associated Factors among Patients with Mood Disorder at St. Paul's Hospital and Millennium Medical College, Addis Ababa, Ethiopia, 2019	Psychiatry journal
Tesfaye, Elias : Kassaw, Chalachew : Agenagnew, Liyew	2021	Functional Disability in Patients with Mood Disorders at St Paul's Hospital Psychiatry Clinic, Addis Ababa, Ethiopia, 2019	Patient related outcome measures
Tesfaye, E. : Worku, B. : Girma, E. : Agenagnew, L.	2020	Internalized stigma among patients with mood disorders in Ethiopia: A cross-sectional facility-based study	International Journal of Mental Health Systems
Thai, T. T. : Tran, V. B. : Nguyen, N. B. T. : Bui, H. H. T.	2022	HIV-related stigma, symptoms of depression and their association with suicidal ideation among people living with HIV in Ho Chi Minh City, Vietnam	Psychology, health & medicine
Thakoor, J. P. D. : Dong, H. : Zhang, X. : Wang, G. : Huang, H. : Xiang, Y. : Hao, W.	2016	Duration of untreated psychosis in Chinese and mauritian: Impact of clinical characteristics and patients' and families' perspectives on psychosis	PLoS ONE
Than, P. Q. T. : Tran, B. X. : Nguyen, C. T. : Truong, N. T. : Thai, T. P. T. : Latkin, C. A. : Ho, C. S. H. : Ho, R. C. M.	2019	Stigma against patients with HIV/AIDS in the rapid expansion of antiretroviral treatment in large drug injection-driven HIV epidemics of Vietnam	Harm Reduction Journal
Theano, P. : Periklis, P. : Vasilis, P. : Elli, K. : Dimitrios, P.	2021	Sarscov-2 psychosomatic effects and fear of stigma on the discharge day of infected individuals: Sapfo study	Psychiatr. Danub.
Thomé, E. S. : Dargél, A. A. : Migliavacca, F. M. : Potter, W. A. : Jappur, D. M. C. : Kapczinski, F. : Cerésér, K. M.	2012	Stigma experiences in bipolar patients: the impact upon functioning	Journal of Psychiatric & Mental Health Nursing (John Wiley & Sons, Inc.)
Tian, Xu : Liao, Zhongli : Yi, Lijuan : Tang, Ling : Chen, Guihua : Jimenez Herrera, Maria F.	2023	Efficacy and mechanisms of 4-week MBSR on psychological distress in lung cancer patients: A single-center, single-blind, longitudinal, randomized controlled trial	Asia-Pacific journal of oncology nursing
Tilahun, Hibret A. : Mariam, Damen Haile : Tsui, Amy O.	2012	Effect of Perceived Stigma on Adherence to Highly Active Antiretroviral Therapy and Self-Confidence to Take Medication Correctly in Addis Ababa, Ethiopia	Journal of HIV/AIDS & Social Services
Todor, I. : Marincu, I.	2010	An investigation of the perceived stigma: Among people with HIV infection in Romania	Int. J. Interdiscip. Soc. Sci.
Todorova, A. : Milanov, I. : Maslarov, D.	2008	Investigating quality of life in Bulgarian patients with Parkinson's disease	International Journal of Neuroprotection and Neuroregeneration
Tombini, M. : Assenza, G. : Quintiliani, L. : Ricci, L. : Lanzone, J. : De Mojà, R. : Ulivi, M. : Di Lazzaro, V.	2019	Epilepsy-associated stigma from the perspective of people with epilepsy and the community in Italy	Epilepsy and Behavior
Tombini, M. : Assenza, G. : Quintiliani, L. : Ricci, L. : Lanzone, J. : Di Lazzaro, V.	2020	Alexithymia and emotion dysregulation in adult patients with epilepsy	Epilepsy and Behavior
Tombini, Mario : Assenza, Giovanni : Quintiliani, Livia : Ricci, Lorenzo : Lanzone, Jacopo : Ulivi, Martina : Di Lazzaro, Vincenzo	2020	Depressive symptoms and difficulties in emotion regulation in adult patients with epilepsy: Association with quality of life and stigma	Epilepsy & behavior : E&B
Tombini, M. : Assenza, G. : Quintiliani, L. : Ricci, L. : Lanzone, J. : Di Lazzaro, V.	2021	Epilepsy and quality of life: what does really matter?	Neurological Sciences
Tong, Ping : Bu, Ping : Yang, Yang : Dong, Liping : Sun, Ting : Shi, Yuanhong	2020	Group cognitive behavioural therapy can reduce stigma and improve treatment compliance in major depressive disorder patients	Early intervention in psychiatry
Tora, A. : Franklin, H. : Deribe, K. : Reda, A. A. : Davey, G.	2013	Podoconiosis-related stigma in wolaita zone, southern Ethiopia: A cross-sectional study	American Journal of Tropical Medicine and Hygiene
Toth, Steven : A York, Jill : DePinto, Nicholas	2016	HIV stigma: perceptions from HIV-positive and HIV-negative patients in a community dental clinic	Journal of dental research, dental clinics, dental prospects
Townley, Greg : Brusilovskiy, Eugene : Salzer, Mark S.	2017	Urban and non-urban differences in community living and participation among individuals with serious mental illnesses	Social Science & Medicine
Traino, Katherine A. : Roberts, Caroline M. : Fisher, Rachel S. : Delozier, Alexandria M. : Austin, Paul F. : Baskin, Laurence S. : Chan, Yee-Ming : Cheng, Earl Y. : Diamond, David A. : Fried, Allyson J. : Kropp, Bradley : Lakshmanan, Yegappan : Meyer, Sabrina Z. : Meyer, Theresa : Buchanan, Cindy : Palmer, Blake W. : Paradis, Alethea : Reyes, Kristy J. : Tishelman, Amy : Williot, Pierre	2022	Stigma, Intrusiveness, and Distress in Parents of Children with a Disorder/Difference of Sex Development	Journal of Developmental & Behavioral Pediatrics
Tran, B. X. : Than, P. Q. T. : Tran, T. T. : Nguyen, C. T. : Latkin, C. A.	2019	Changing sources of stigma against patients with HIV/AIDS in the rapid expansion of antiretroviral treatment services in Vietnam	BioMed Research International
Tran, T. N. : Ha, U. N. L. : Nguyen, T. M. : Nguyen, T. D. : Vo, K. N. C. : Dang, T. H. : Trinh, P. M. P. : Truong, D.	2021	The effect of Non-Motor symptoms on Health-Related quality of life in patients with young onset Parkinson's Disease: A single center Vietnamese Cross-Sectional study	Clinical Parkinsonism and Related Disorders
Trani, J. F. : Bakhshi, P. : Kuhlberg, J. : Narayanan, S. S. : Venkataraman, H. : Mishra, N. N. : Groce, N. E. : Jadhav, S. : Deshpande, S.	2015	Mental illness, poverty and stigma in India: A case-control study	BMJ Open
Trejos-Herrera, A. M. : Vinaccia, S. : Bahamón, M. J. : Alarcón-Vásquez, Y. : Giraldo, M. R. : García, G. G. : Lozano, K. F. : Earnshaw, V. A.	2021	Translation and validation of the Spanish version of the Chronic Illness Anticipated Stigma Scale (CIASS) in Colombian patients diagnosed with chronic illnesses	Adv. Ment. Health

Tsai, A. C. : Bangsberg, D. R. : Emenyonu, N. : Senkungu, J. K. : Martin, J. N. : Weiser, S. D.	2011	The social context of food insecurity among persons living with HIV/AIDS in rural Uganda	Social Science and Medicine
Tsai, J. : Lysaker, P. H. : Vohs, J. L.	2010	Negative symptoms and concomitant attention deficits in schizophrenia: Associations with prospective assessments of anxiety, social dysfunction, and avoidant coping	Journal of Mental Health
Tsai, W. : Lu, Q.	2019	Ambivalence over emotional expression and intrusive thoughts as moderators of the link between self-stigma and depressive symptoms among Chinese American breast cancer survivors	Journal of behavioral medicine
Tsang, H. W. H. : Fung, K. M. T. : Corrigan, P. W.	2006	Psychosocial treatment compliance scale for people with psychotic disorders	Aust. New Zealand J. Psychiatry
Tsegabrhan, H. : Negash, A. : Tesfay, K. : Abera, M.	2014	Co-morbidity of depression and epilepsy in jimma university specialized hospital, southwest ethiopia	Neurology India
Tseng, W. T. : Lee, Y. : Hung, C. F. : Lin, P. Y. : Chien, C. Y. : Chuang, H. C. : Fang, F. M. : Li, S. H. : Huang, T. L. : Chong, M. Y. : Wang, L. J.	2019	Validation of the chinese version of the shame and stigma scale in patients with head and neck cancer	Cancer Management and Research
Tseng, W. T. : Lee, Y. : Hung, C. F. : Lin, P. Y. : Chien, C. Y. : Chuang, H. C. : Fang, F. M. : Li, S. H. : Huang, T. L. : Chong, M. Y. : Wang, L. J.	2022	Stigma, depression, and anxiety among patients with head and neck cancer	Supportive Care in Cancer
Tsutsumi, A. : Izutsu, T. : Islam, M. D. A. : Amed, J. U. : Nakahara, S. : Takagi, F. : Wakai, S.	2004	Depressive status of leprosy patients in Bangladesh: Association with self-perception of stigma	Leprosy Review
Tsutsumi, A. : Izutsu, T. : Islam, A. M. : Maksuda, A. N. : Kato, H. : Wakai, S.	2007	The quality of life, mental health, and perceived stigma of leprosy patients in Bangladesh	Social Science & Medicine
Tu, Chao-Ying : Liu, Wei-Shih : Chen, Yen-Fu : Huang, Wei-Lieh	2022	Patients who complain of autonomic dysregulation: A cross-sectional study of patients with somatic symptom disorder	International Journal of Social Psychiatry
Tun, M. M. M. : Mongkolchati, A. : Aung, M. N. : Aung, M. Y. : Laosee, O.	2019	Determinants of quality of life among people living with HIV in the hilly region of Myanmar	J. HIV/AIDS Soc. Serv.
Tun, W. : Apicella, L. : Casalini, C. : Bikaru, D. : Mbita, G. : Jeremiah, K. : Makyao, N. : Koppenhaver, T. : Mlanga, E. : Vu, L.	2019	Community-Based Antiretroviral Therapy (ART) Delivery for Female Sex Workers in Tanzania: 6-Month ART Initiation and Adherence	AIDS Behav.
Turan, Bulent : Browning, Wesley : Budhwani, Henna : Turan, Janet : Fazeli, Pariya : Raper, James : Mugavero, Michael	2017	How Does Stigma Affect People Living with HIV? The Mediating Roles of Internalized and Anticipated HIV Stigma in the Effects of Perceived Community Stigma on Health and Psychosocial Outcomes	AIDS & Behavior
Turan, B. : Crockett, K. B. : Buyukcan-Tetik, A. : Kempf, M. C. : Konkle-Parker, D. : Wilson, T. E. : Tien, P. C. : Wingood, G. : Neilands, T. B. : Johnson, M. O. : Weiser, S. D. : Turan, J. M.	2019	Buffering Internalization of HIV Stigma: Implications for Treatment Adherence and Depression	J. Acquired Immune Defic. Syndr.
Turan, B. : Rice, W. S. : Crockett, K. B. : Johnson, M. : Neilands, T. B. : Ross, S. N. : Kempf, M. C. : Konkle-Parker, D. : Wingood, G. : Tien, P. C. : Cohen, M. : Wilson, T. E. : Logie, C. H. : Sosanya, O. : Plankey, M. : Golub, E. : Adimora, A. A. : Parish, C. : Weiser, S. D. : Turan, J. M.	2019	Longitudinal association between internalized HIV stigma and antiretroviral therapy adherence for women living with HIV: The mediating role of depression	AIDS
Turan, B. : Stringer, K. L. : Onono, M. : Bukusi, E. A. : Weiser, S. D. : Cohen, C. R. : Turan, J. M.	2014	Linkage to HIV care, postpartum depression, and HIV-related stigma in newly diagnosed pregnant women living with HIV in Kenya: A longitudinal observational study	BMC Pregnancy Childbirth
Turan, G. B. : Özer, Z. : Özden, B.	2022	The Effects of Perceived Stigma on the Concealment of Disease and Satisfaction with Life in Patients with Epilepsy: An Example in Eastern Turkey	International journal of clinical practice
Tymejczyk, O. : Vo, Q. : Kulkarni, S. G. : Antelman, G. : Boshe, J. : Reidy, W. : Parcesepe, A. : Nash, D. : Elul, B.	2021	Tracing-corrected estimates of disengagement from HIV care and mortality among patients enrolling in HIV care without overt immunosuppression in Tanzania	AIDS Care - Psychological and Socio-Medical Aspects of AIDS/HIV
Ucok, Alp : Karadayi, Gulsah : Emiroglu, Birgul : Sartorius, Norman	2013	Anticipated discrimination is related to symptom severity, functionality and quality of life in schizophrenia	Psychiatry research
Uhlmann, C. : Kaehler, J. : Harris, M. S. H. : Unser, J. : Arolt, V. : Lencer, R.	2014	Negative impact of self-stigmatization on attitude toward medication adherence in patients with psychosis	Journal of Psychiatric Practice
Umar, E. : Levy, J. A. : Bailey, R. C. : Donenberg, G. : Hershow, R. C. : Mackesy-Amiti, M. E.	2019	Virological Non-suppression and Its Correlates Among Adolescents and Young People Living with HIV in Southern Malawi	AIDS Behav.
Unal, Gulin Ozdamar : Atay, Inci Meltem	2022	Complementary and alternative medicine use and its association with clinical features and functioning in patients with bipolar I disorder: A cross-sectional study	Dusunen Adam: Journal of Psychiatry & Neurological Sciences
Ustundag, Mehmet Fatih : Kesebir, Sermin	2013	[Internalized stigmatization in bipolar patients: relationship with clinical properties, quality of life and treatment compliance]	Iki uclu bozukluklta icsellestirilmis damgalanma: klinik ozellikler, yasam kalitesi ve tedaviye uyum ile iliskisi.
Uys, L. : Chirwa, M. : Kohi, T. : Greeff, M. : Naidoo, J. : Makoae, L. : Dlamini, P. : Durrheim, K. : Cuca, Y. : Holzemer, W. L.	2009	Evaluation of a health setting-based stigma intervention in five African countries	AIDS Patient Care & STDs
Valenzuela, C. : Ugarte-Gil, C. : Paz, J. : Echevarria, J. : Gotuzzo, E. : Vermund, S. H. : Kipp, A. M.	2015	HIV stigma as a barrier to retention in HIV care at a general hospital in Lima, Peru: a case-control study	AIDS and behavior
Valenzuela, C. V. : Kipp, A. : Ugarte, C. : Paz, J. : Echevarria, J. : Gotuzzo, E.	2012	Hiv stigma as a barrier to receiving hiv care at a general hospital in lima, Peru: A case-control study	American Journal of Tropical Medicine and Hygiene
Vallabh, P. K. : Rashiq, S. : Verrier, M. J. : Baker, G. : Sanderman, B. : Dick, B. D.	2015	The effect of a cognitive-behavioral therapy chronic pain management program on perceived stigma: a clinical controlled trial	Journal of pain management
van Brakel, W. H. : Sihombing, B. : Djarir, H. : Beise, K. : Kusumawardhani, L. : Yulihane, R. : Kurniasari, I. : Kasim, M. : Kesumaningsih, K. I. : Wilder-Smith, A.	2012	Disability in people affected by leprosy: the role of impairment, activity, social participation, stigma and discrimination	Global health action
van den Berg, Jacob J. : Neilands, Torsten B. : Johnson, Mallory O. : Chen, Bing : Saberi, Parya	2016	Using Path Analysis to Evaluate the Healthcare Empowerment Model Among Persons Living with HIV for Antiretroviral Therapy Adherence	AIDS Patient Care & STDs
van der Beek, K. M. : Bos, I. : Middel, B. : Wynia, K.	2013	Experienced stigmatization reduced quality of life of patients with a neuromuscular disease: a cross-sectional study	Clinical rehabilitation
Van Der Kooij, Y. L. : Kupková, A. : Den Daas, C. : Van Den Berk, G. E. L. : Kleene, M. J. T. : Jansen, H. S. E. : Elsenburg, L. J. M. : Schenk, L. G. : Verboon, P. : Brinkman, K. : Bos, A. E. R. : Stutterheim, S. E.	2021	Role of Self-Stigma in Pathways from HIV-Related Stigma to Quality of Life among People Living with HIV	AIDS Patient Care and STDs
Van Rie, A. : Sengupta, S. : Pungrassami, P. : Balthip, Q. : Choonuan, S. : Kasetjaroen, Y. : Strauss, R. P. : Chongsuvivatwong, V.	2008	Measuring stigma associated with tuberculosis and HIV/AIDS in southern Thailand: Exploratory and confirmatory factor analyses of two new scales	Tropical Medicine and International Health
Van Tam, V. : Larsson, M. : Pharris, A. : Diedrichs, B. : Nguyen, H. P. : Nguyen, C. T. K. : Ho, P. D. : Marrone, G. : Thorson, A.	2012	Peer support and improved quality of life among persons living with HIV on antiretroviral treatment: A randomised controlled trial from north-eastern Vietnam	Health and Quality of Life Outcomes
Van Veen, M. G. : Trienekens, S. C. M. : Heijman, T. : Gotz, H. M. : Zaheri, S. : Ladbury, G. : De Wit, J. : Fennema, J. S. A. : De Wolf, F. : Van Der Sande, M. A. B.	2015	Delayed linkage to care in one-third of HIV-positive individuals in the Netherlands	Sexually Transmitted Infections
Van Voorhees, B. W. : Gollan, J. : Fogel, J.	2012	Pilot study of internet-based early intervention for combat-related mental distress	J. Rehabil. Res. Dev.
Vanable, P. A. : Carey, M. P. : Blair, D. C. : Littlewood, R. A.	2006	Impact of HIV-related stigma on health behaviors and psychological adjustment among HIV-positive men and women	AIDS & Behavior
Vanbellingen, T. : Nyffeler, T. : Nef, T. : Kwakkel, G. : Bohlhalter, S. : van Wegen, E. E. H.	2016	Reliability and validity of a new dexterity questionnaire (DextQ-24) in Parkinson's disease	Parkinsonism and Related Disorders
Vancampfort, D. : Byansi, P. : Ward, P. B. : Mugisha, J.	2021	Correlates of missed HIV appointments in low-resource settings: a study from Uganda	African Journal of AIDS Research
Vancampfort, D. : Byansi, P. : Kinyanda, E. : Bbosa, R. S. : Mugisha, J.	2021	Internalised HIV-related stigma associated with physical inactivity in people with HIV and AIDS: A cross-sectional study from Uganda	African Journal of AIDS Research
Varagur, K. : Skolnick, G. B. : Naidoo, S. D. : Smyth, M. D. : Patel, K. B.	2022	School-age outcomes in patients with unilateral coronal synostosis: comparison of fronto-orbital advancement and endoscopic strip craniectomy	Journal of Neurosurgery: Pediatrics
Vass, V. : Morrison, A. P. : Law, H. : Dudley, J. : Taylor, P. : Bennett, K. M. : Bentall, R. P.	2015	How stigma impacts on people with psychosis: The mediating effect of self-esteem and hopelessness on subjective recovery and psychotic experiences	Psychiatry Res.
Vaughn-Sandler, V. : Sherman, C. : Aronsohn, A. : Volk, M. L.	2014	Consequences of perceived stigma among patients with cirrhosis	Digestive Diseases and Sciences
Vauth, R. : Kleim, B. : Wirtz, M. : Corrigan, P. W.	2007	Self-efficacy and empowerment as outcomes of self-stigmatizing and coping in schizophrenia	Psychiatry Research
Vayshenker, B. : Mulay, A. L. : Gonzales, L. : West, M. L. : Brown, I. : Yanos, P. T.	2016	Participation in peer support services and outcomes related to recovery	Psychiatric rehabilitation journal
Vázquez, G. H. : Kapczinski, F. : Magalhaes, P. V. : Córdoba, R. : Lopez Jaramillo, C. : Rosa, A. R. : Sanchez De Carmona, M. : Tohen, M.	2011	Stigma and functioning in patients with bipolar disorder	Journal of Affective Disorders
Vega, W. A. : Rodriguez, M. A. : Ang, A.	2010	Addressing stigma of depression in Latino primary care patients	General Hospital Psychiatry
Veld, D. H. : Pengpid, S. : Colebunders, R. : Skaal, L. : Peltzer, K.	2017	High-risk alcohol use and associated socio-demographic, health and psychosocial factors in patients with HIV infection in three primary health care clinics in South Africa	International Journal of STD and AIDS
Verdoux, H. : Quiles, C. : Bon, L. : Chéreau-Boudet, I. : Dubreucq, J. : Legros-Lafarge, E. : Guillard-Bouhet, N. : Massoubre, C. : Plasse, J. : Franck, N.	2021	Characteristics associated with self-reported medication adherence in persons with psychosis referred to psychosocial rehabilitation centers	Eur. Arch. Psychiatry Clin. Neurosci.

Verhaak, A. M. S. : Ferrand, J. : Puhl, R. M. : Tishler, D. S. : Papasavas, P. K. : Umashanker, D.	2022	Experienced weight stigma, internalized weight bias, and clinical attrition in a medical weight loss patient sample	International Journal of Obesity
Verhaeghe, Mieke : Bracke, Piet	2011	Stigma and Trust Among Mental Health Service Users	Archives of Psychiatric Nursing
Verma, M. : Arora, A. : Malviya, S. : Nehra, A. : Sagar, R. : Tripathi, M.	2015	Do expressed emotions result in stigma? A potentially modifiable factor in persons with epilepsy in India	Epilepsy and Behavior
Vetrova, Marina V. : Cheng, Debbie M. : Bendiks, Sally : Gnatienko, Natalia : Lloyd-Travaglini, Christine : Jiang, Wenqing : Luoma, Jason : Blokhina, Elena : Krupitsky, Evgeny : Lioznov, Dmitry : Ekstrand, Maria L. : Raj, Anita : Samet, Jeffrey H. : Lunze, Karsten	2021	HIV and Substance Use Stigma, Intersectional Stigma and Healthcare Among HIV-Positive PWID in Russia	AIDS & Behavior
Victorson, D. : Cavazos, J. E. : Holmes, G. L. : Reder, A. T. : Wojna, V. : Nowinski, C. : Miller, D. : Buono, S. : Mueller, A. : Moy, C. : Cella, D.	2014	Validity of the Neurology Quality-of-Life (Neuro-QoL) measurement system in adult epilepsy	Epilepsy and Behavior
Victoryna, Fallon : Yona, Sri : Waluyo, Agung	2019	The relationship between stigma, family acceptance, peer support and stress level among HIV-positive men who have sex with men (MSM) in Medan, North Sumatera, Indonesia	Enfermería Clínica
Vidojević, I. M. : Jocić, D. E. : Tosković, O.	2012	Comparative study of experienced and anticipated stigma in Serbia and the world	The International journal of social psychiatry
Vidović, D. : Brečić, P. : Vilibić, M. : Jukić, V.	2016	Insight and self-stigma in patients with schizophrenia	Acta Clinica Croatica
Vilhena, Estela : Pais-Ribeiro, José : Silva, Isabel : Cardoso, Helena : Mendonça, Denisa	2014	Predictors of Quality of Life in Portuguese Obese Patients: A Structural Equation Modeling Application	Journal of Obesity
Villotti, Patrizia : Corbière, Marc : Dewa, Carolyn S. : Fraccaroli, Franco : Sultan-Taïeb, Hélène : Zaniboni, Sara : Lecomte, Tania	2018	A serial mediation model of workplace social support on work productivity: the role of self-stigma and job tenure self-efficacy in people with severe mental disorders	Disability & Rehabilitation
Visser, M. J. : Kershaw, T. : Makin, J. D. : Forsyth, B. W. C.	2008	Development of parallel scales to measure HIV-related stigma	AIDS Behav.
Viteva, E.	2013	Impact of stigma on the quality of life of patients with refractory epilepsy	Seizure
Viteva, E. I.	2014	Quality of life predictors in patients with epilepsy and cognitive disabilities	International Journal of Epilepsy
Viteva, E. : Semerdjieva, M.	2015	Enacted stigma among patients with epilepsy and intellectual impairment	Epilepsy and Behavior
von Hippel, C. : Brener, L. : Horwitz, R.	2018	Implicit and explicit internalized stigma: Relationship with risky behaviors, psychosocial functioning and healthcare access among people who inject drugs	Addict. Behav.
Von Mach, T. : Rodriguez, K. : Mojtabai, R. : Spivak, S. : Eaton, W. W. : Cullen, B. A.	2022	The relationship between social and environmental factors and symptom severity in the seriously mentally ill population	Int. J. Soc. Psychiatry
Vos, J. : Van Asperen, C. J. : Oosterwijk, J. C. : Menko, F. H. : Collee, M. J. : Garcia, E. G. : Tibben, A.	2013	The counselees' self-reported request for psychological help in genetic counseling for hereditary breast/ovarian cancer: Not only psychopathology matters	Psycho-Oncology
Vrbová, K. : Kamarádová, D. : Látalová, K. : Ocisková, M. : Praško, J. : Mainerová, B. : Cinculová, A. : Kubínek, R. : Ticháčková, A.	2014	Self-stigma and adherence to medication in patients with psychotic disorders - Cross-sectional study	Neuroendocrinology Letters
Vrbova, K. : Prasko, J. : Holubova, M. : Kamaradova, D. : Ociskova, M. : Marackova, M. : Latalova, K. : Grambal, A. : Slepecky, M. : Zatkova, M.	2016	Self-stigma and schizophrenia: A cross-sectional study	Neuropsychiatric Disease and Treatment
Vrbova, K. : Prasko, J. : Ociskova, M. : Holubova, M.	2017	Comorbidity of schizophrenia and social phobia - impact on quality of life, hope, and personality traits: A cross sectional study	Neuropsychiatric Disease and Treatment
Vrbova, K. : Prasko, J. : Ociskova, M. : Kamaradova, D. : Marackova, M. : Holubova, M. : Grambal, A. : Slepecky, M. : Latalova, K.	2017	Quality of life, self-stigma, and hope in schizophrenia spectrum disorders: A cross-sectional study	Neuropsychiatric Disease and Treatment
Vrbova, K. : Prasko, J. : Ociskova, M. : Holubova, M. : Kantor, K. : Kolek, A. : Grambal, A. : Slepecky, M.	2018	Suicidality, self-stigma, social anxiety and personality traits in stabilized schizophrenia patients - A cross-sectional study	Neuropsychiatric Disease and Treatment
Vreeman, R. C. : Scanlon, M. L. : Marete, I. : Mwangi, A. : Inui, T. S. : McAteer, C. I. : Nyandiko, W. M.	2015	Characteristics of HIV-infected adolescents enrolled in a disclosure intervention trial in western Kenya	AIDS Care Psychol. Socio-Med. Asp. AIDS HIV
Vreeman, R. C. : Scanlon, M. L. : Tu, W. : Slaven, J. : McAteer, C. : Aluoch, J. : Ayaya, S. : Nyandiko, W. M.	2019	Validation of an HIV/AIDS Stigma Measure for Children Living with HIV and Their Families	Journal of the International Association of Providers of AIDS Care
Vyavaharkar, Medha : Moneyham, Linda : Murdaugh, Carolyn : Tavakoli, Abbas	2012	Factors Associated with Quality of Life Among Rural Women with HIV Disease	AIDS & Behavior
Wadle, N. E. : Schwab, C. : Seifart, C. : von Podewils, F. : Knake, S. : Willems, L. M. : Menzler, K. : Schulz, J. : Conradi, N. : Rosenow, F. : Strzelczyk, A.	2022	Prospective, longitudinal, multicenter study on the provision of information regarding sudden unexpected death in epilepsy to adults with epilepsy	Epilepsia
Wadley, A. : Kamerman, P. : Pincus, T. : Evangeli, M. : Chinaka, T. : Francois Venter, W. D. : Akpomiemie, G. : Moorhouse, M. : Parker, R.	2022	Slow and Steady But Not Related to HIV Stigma: Physical Activity in South Africans Living with HIV and Chronic Pain	AIDS and behavior
Wagner, G. J. : Gwokyalya, V. : Akena, D. : Nakigudde, J. : McBain, R. : Faherty, L. : Ngo, V. : Nakku, J. : Kyohangirwe, L. : Banegura, A. : Beyeza-Kashesya, J. : Wanyenze, R. K.	2022	Stressors and Maladaptive Coping Mechanisms Associated with Elevated Perinatal Depressive Symptoms and Suicidality Among Women Living with HIV in Uganda	International journal of behavioral medicine
Wagner, Glenn J. : Ngo, Victoria K. : Nakasujja, Noeline : Akena, Dickens : Aunon, Frances : Musisi, Seggane	2014	Impact of antidepressant therapy on cognitive aspects of work, condom use, and psychosocial well-being among HIV clients in Uganda	International journal of psychiatry in medicine
Wagner, G. : Linnemayr, S. : Kityo, C. : Mugyenyi, P.	2012	Factors associated with intention to conceive and its communication to providers among HIV clients in Uganda	Maternal and child health journal
Waljee, J. F. : Hu, E. S. : Ubel, P. A. : Smith, D. M. : Newman, L. A. : Alderman, A. K.	2008	Effect of esthetic outcome after breast-conserving surgery on psychosocial functioning and quality of life	Journal of Clinical Oncology
Waljee, J. F. : Ubel, P. A. : Atisha, D. M. : Hu, E. S. : Alderman, A. K.	2011	The choice for breast cancer surgery: Can women accurately predict postoperative quality of life and disease-related stigma?	Annals of Surgical Oncology
Wan, M. T. : Pearl, R. L. : Fuxench, Z. C. C. : Takeshita, J. : Gelfand, J. M.	2020	Anticipated and Perceived Stigma Among Patients With Psoriasis	Journal of Psoriasis and Psoriatic Arthritis
Wan, X. J. : Li, J. J. : Wang, X. J. : Zhang, Y. : Liu, Y. : Wu, C. : Wang, K. F.	2014	The bothersomeness of female urinary incontinence and its influencing factors: Study from a Chinese city	International Journal of Nursing Sciences
Wan, X. : Wang, C. : Xu, D. : Guan, X. : Sun, T. : Wang, K.	2014	Disease stigma and its mediating effect on the relationship between symptom severity and quality of life among community-dwelling women with stress urinary incontinence: a study from a Chinese city	Journal of clinical nursing
Wang, D. F. : Zhou, Y. N. : Liu, Y. H. : Hao, Y. Z. : Zhang, J. H. : Liu, T. Q. : Ma, Y. J.	2022	Social support and depressive symptoms: exploring stigma and self-efficacy in a moderated mediation model	BMC Psychiatry
Wang, L. : Chen, Y. : Hu, C. : Qin, H.	2021	Influence of Family Dynamics on Stigma Experienced by Patients With Schizophrenia: Mediating Effect of Quality of Life	Frontiers in Psychiatry
Wang, L. : Dowdy, D. W. : Comins, C. A. : Young, K. : McIngana, M. : Mulumba, N. : Mhlophe, H. : Chen, C. : Hausler, H. : Schwartz, S. R. : Baral, S. : Mishra, S.	2021	Health-related quality of life of female sex workers living with HIV in South Africa: a cross-sectional study	medRxiv
Wang, L. : Wu, D. : Wu, S. : Liu, Y. : Tan, X. : Liu, Y. : Wu, Z. : Wang, Q. : He, X.	2022	The Effect of Narrative Nursing Intervention on Shame in Elderly Patients with Bladder Cancer after Ileal Bladder Replacement: A Cohort Study	Comp. Math. Methods Med.
Wang, P. : Gao, J. Y. : Zhou, H. J. : Wu, J. : Wang, Y.	2022	Health-related quality of life of Chinese AIDS patients: a multi-region study	Quality of Life Research
Wang, Qian : Fong, Vivian W. I. : Qin, Qinghua : Yao, Hui : Zheng, Jiarui : Wang, Xiaoyan : Wang, Ailing : Gao, Qun : Mo, Phoenix K. H.	2022	Trends in the psychosocial and mental health of HIV-positive women in China from 2015 to 2020: Results from two cross-sectional surveys	Health Expectations
Wang, Ruey-Hsia : Lin, Chia-Chin : Chen, Shi-Yu : Hsu, Hui-Chun : Huang, Chiu-Ling	2021	The Impact of Self-Stigma, Role Strain, and Diabetes Distress on Quality of Life and Glycemic Control in Women With Diabetes: A 6-Month Prospective Study	Biological Research for Nursing
Wang, W. : Yang, N. : Li, X. : Xiao, H. : Gao, M. : Yan, H. : Li, S.	2019	A pathway analysis of exploring how HIV-related stigma affects social capital among people living with HIV/AIDS in China	Psychology, health & medicine
Wang, X. Q. : Petrini, M. : Morisky, D. E.	2016	Comparison of the Quality of Life, Perceived Stigma and Medication Adherence of Chinese with Schizophrenia: A Follow-Up Study	Archives of psychiatric nursing
Wang, X. Q. : Petrini, M. A. : Morisky, D. E.	2017	Predictors of quality of life among Chinese people with schizophrenia	Nursing & health sciences
Wang, Y. : Li, S. : Gong, J. : Cao, L. : Xu, D. : Yu, Q. : Wang, X. : Chen, Y.	2022	Perceived Stigma and Self-Efficacy of Patients With Inflammatory Bowel Disease-Related Stoma in China: A Cross-Sectional Study	Frontiers in Medicine
Wang, Y. Y. : Wang, T. : Yan, H. : D'Amato, R. C. : Wang, W. : Li, S. Y.	2019	Evaluating the relationship between adherence to Highly Active Antiretroviral Therapy (HAART) and social and clinical characteristics in Chinese patients with HIV	AIDS Care - Psychological and Socio-Medical Aspects of AIDS/HIV
Wanjala, S. W. : Nyongesa, M. K. : Mwangi, P. : Mutua, A. M. : Luchters, S. : Newton, C. R. J. C. : Abubakar, A.	2022	Measurement characteristics and correlates of HIV-related stigma among adults living with HIV: a cross-sectional study from coastal Kenya	BMJ Open
Wardell, J. D. : Shuper, P. A. : Rourke, S. B. : Hendershot, C. S.	2018	Stigma, Coping, and Alcohol Use Severity Among People Living With HIV: A Prospective Analysis of Bidirectional and Mediated Associations	Annals of behavioral medicine : a publication of the Society of Behavioral Medicine

Warren, A. M. : Khetan, R. : Bennett, M. : Pogue, J. : Waddimba, A. C. : Powers, M. B. : Sanchez, K.	2022	The Relationship Between Stigma and Mental Health in a Population of Individuals With COVID-19	Rehabilitation Psychology
Watson, Jack D. : Perrin, Paul B.	2022	Relations among Stigma, Quality of Life, Resilience, and Life Satisfaction in Individuals with Burn Injuries	European Burn Journal (EBJ)
Watt, M. H. : Minja, L. : Knettel, B. A. : Mwamba, R. N. : Osaki, H. : Ngocho, J. S. : Kisigo, G. A. : Renju, J. : Vissoci, J. R. N. : Sao, S. S. : Mmbaga, B. T.	2021	Pilot Outcomes of Maisha: An HIV Stigma Reduction Intervention Developed for Antenatal Care in Tanzania	AIDS Behav.
Waynor, William R. : Eissenstat, SunHee J. : Yanos, Phillip T. : Reinhardt-Wood, Dawn : Taylor, Ellen : Karyczak, Sean : Lu, Weili	2020	The Role of Illness Identity in Assertive Community Treatment	Rehabilitation Counseling Bulletin
Waynor, William R. : Karyczak, Sean : Taylor, Ellen : Eissenstat, Sunhee J. : Reinhardt-Wood, Dawn	2020	A Mediation Model: Fighting Internalized Stigma to Create Hope for People Living with Serious Mental Illness	Journal of Rehabilitation
Wciórka, J. : Świtaj, P. : Anczewska, M.	2015	The sense of empowerment in the early stage of recovery from psychosis	Psychosis
Wedajo, S. : Degu, G. : Deribew, A. : Ambaw, F.	2022	Social support, perceived stigma, and depression among PLHIV on second-line antiretroviral therapy using structural equation modeling in a multicenter study in Northeast Ethiopia	International Journal of Mental Health Systems
Wee, Christina C. : Davis, Roger B. : Chiodi, Sarah : Huskey, Karen W. : Hamel, Mary B.	2015	Sex, race, and the adverse effects of social stigma vs. other quality of life factors among primary care patients with moderate to severe obesity	Journal of general internal medicine
Wei, L. : Yan, H. : Guo, M. : Tian, J. : Jiang, Q. : Zhai, M. : Zhu, B. : Yin, X. : Liao, Y. : Yu, B.	2022	Perceived HIV Stigma, Depressive Symptoms, Self-esteem, and Suicidal Ideation Among People Living with HIV/AIDS in China: a Moderated Mediation Modeling Analysis	Journal of racial and ethnic health disparities
Wei, Z. : Ren, L. : Yang, L. : Liu, C. : Cao, M. : Yang, Q. : Liu, X. : Liu, Y. : Deng, Y.	2021	The relationship between social anxiety and felt stigma in patients with epilepsy: A network analysis	Seizure
Weineland, S. : Lillis, J. : Dahl, J.	2013	Measuring experiential avoidance in a bariatric surgery population - Psychometric properties of AAQ-W	Obes. Res. Clin. Pract.
Weiss, M. G. : Jadhav, S. : Raguram, R. : Vounatsou, P. : Littlewood, R.	2001	Psychiatric stigma across cultures: Local validation in Bangalore and London	Anthropology and Medicine
Wells, J. : Flowers, L. : Mehta, C. C. : Chandler, R. : Knott, R. : McDonnell Holstad, M. : Watkins Bruner, D.	2022	Follow-Up to High-Resolution Anoscopy after Abnormal Anal Cytology in People Living with HIV	AIDS Patient Care STDs
Wen, Y. : Shi, Y. : Jiang, C. : Detels, R. : Wu, D.	2013	HIV/AIDS patients' medical and psychosocial needs in the era of HAART: a cross-sectional study among HIV/AIDS patients receiving HAART in Yunnan, China	AIDS care
Werner, P. : Aviv, A. : Barak, Y.	2008	Self-stigma, self-esteem and age in persons with schizophrenia	International Psychogeriatrics
West, M. L. : Vayshenker, B. : Rotter, M. : Yanos, P. T.	2015	The Influence of Mental Illness and Criminality Self-Stigmas and Racial Self-Concept on Outcomes in a Forensic Psychiatric Sample	Psychiatr. Rehab. J.
Whatley, A. D. : Dilorio, C. K. : Yeager, K.	2010	Examining the relationships of depressive symptoms, stigma, social support and regimen-specific support on quality of life in adult patients with epilepsy	Health Education Research
Wickersham, K. E. : Sereika, S. M. : Kang, H. J. : Tamres, L. K. : Erlen, J. A.	2018	Use of a Self-Report Medication Adherence Scale for Measuring Adherence to Antiretroviral Therapy in Patients With HIV/AIDS	Journal of nursing measurement
Wight, R. G. : Aneshensel, C. S. : Murphy, D. A. : Miller-Martinez, D. : Beals, K. P.	2006	Perceived HIV stigma in AIDS caregiving dyads	Social Science & Medicine
Wiginton, J. M. : Maksut, J. L. : Murray, S. M. : Augustinavicius, J. L. : Kall, M. : Delpech, V. : Baral, S. D.	2021	Brief report: HIV-related healthcare stigma/discrimination and unmet needs among persons living with HIV in England and Wales	Preventive Medicine Reports
Willemse, H. : Geenen, R. : Van Loey, N. E.	2021	Reliability and structural validity of the Dutch version of Perceived Stigmatization Questionnaire in adults with burns	Burns
Willemse, H. : Geenen, R. : Egberts, M. R. : Engelhard, I. M. : Van Loey, N. E.	2021	Perceived stigmatization and fear of negative evaluation: Two distinct pathways to body image dissatisfaction and self-esteem in burn survivors	Psychology & health
Williams, Ann B. : Wang, Honghong : Li, Xianhong : Chen, Jia : Li, Ling : Fennie, Kristopher	2014	Efficacy of an evidence-based ARV adherence intervention in China	AIDS patient care and STDs
Williams, Leslie D. : Friedman, S. R. : Downing, M. J. : Morgan, E. : Korobchuk, A. : Smyrnov, P. : Pavlitina, E. : Nikolopoulos, G. K. : Hadjikou, A. : Skaathun, B. : Schneider, J. : Kostaki, E. G. : Paraskevis, D. : Hatzakis, A. : Vasylyeva, T. I. : Psichogiou, M.	2019	Experiences of Stigma and Support Reported by Participants in a Network Intervention to Reduce HIV Transmission in Athens, Greece; Odessa, Ukraine; and Chicago, Illinois	AIDS & Behavior
Williams, R. : Brumback, B. : Cook, R. : Cook, C. : Ezenwa, M. : Lucero, R.	2022	Prevalence of HIV-related stigma in adults living with HIV & disability in Florida, 2015-2016	AIDS Care - Psychological and Socio-Medical Aspects of AIDS/HIV
Williamson, Heidi : Hamlet, Claire : White, Paul : Marques, Elsa M. R. : Paling, Thomas : Cadogan, Julia : Perera, Rohan : Rumsey, Nichola : Hayward, Leighton : Harcourt, Diana	2019	A Web-Based Self-Help Psychosocial Intervention for Adolescents Distressed by Appearance-Affecting Conditions and Injuries (Young Persons' Face IT): Feasibility Study for a Parallel Randomized Controlled Trial	JMIR mental health
Williamson, T. J. : Choi, A. K. : Kim, J. C. : Garon, E. B. : Shapiro, J. R. : Irwin, M. R. : Goldman, J. W. : Bornyazan, K. : Carroll, J. M. : Stanton, A. L.	2018	A Longitudinal Investigation of Internalized Stigma, Constrained Disclosure, and Quality of Life Across 12 Weeks in Lung Cancer Patients on Active Oncologic Treatment	Journal of Thoracic Oncology
Williamson, T. J. : Kwon, D. M. : Riley, K. E. : Shen, M. J. : Hamann, H. A. : Ostroff, J. S.	2020	Lung Cancer Stigma: Does Smoking History Matter?	Annals of behavioral medicine : a publication of the Society of Behavioral Medicine
Williamson, Timothy J. : Ostroff, Jamie S. : Haque, Noshin : Martin, Chloe M. : Hamann, Heidi A. : Banerjee, Smita C. : Shen, Megan J.	2020	Dispositional shame and guilt as predictors of depressive symptoms and anxiety among adults with lung cancer: The mediational role of internalized stigma	Stigma and health
Williamson, Timothy J. : Ostroff, Jamie S. : Martin, Chloé M. : Banerjee, Smita C. : Bylund, Carma L. : Hamann, Heidi A. : Shen, Megan Johnson	2021	Evaluating relationships between lung cancer stigma, anxiety, and depressive symptoms and the absence of empathic opportunities presented during routine clinical consultations	Patient Education & Counseling
Willis, N. : Milanzi, A. : Mawodzeke, M. : Dziwa, C. : Armstrong, A. : Yekeye, I. : Mtshali, P. : James, V.	2019	Effectiveness of community adolescent treatment supporters (CATS) interventions in improving linkage and retention in care, adherence to ART and psychosocial well-being: a randomised trial among adolescents living with HIV in rural Zimbabwe	BMC public health
Wo, M. C. M. : Lim, K. S. : Choo, W. Y. : Tan, C. T.	2016	Factors affecting the employability in people with epilepsy	Epilepsy Research
Wohl, A. R. : Galvan, F. H. : Carlos, J. A. : Myers, H. F. : Garland, W. : Witt, M. D. : Cadden, J. : Operskalski, E. : Jordan, W. : George, S.	2013	A comparison of MSM stigma, HIV stigma and depression in HIV-positive latino and African American men who have sex with men (MSM)	AIDS Behav.
Woith, W. M. : Larson, J. L.	2008	Delay in seeking treatment and adherence to tuberculosis medications in Russia: A survey of patients from two clinics	International Journal of Nursing Studies
Wong, M. : Myer, L. : Zerbe, A. : Phillips, T. : Petro, G. : Mellins, C. A. : Remien, R. H. : Shiau, S. : Brittain, K. : Abrams, E. J.	2017	Depression, alcohol use, and stigma in younger versus older HIV-infected pregnant women initiating antiretroviral therapy in Cape Town, South Africa	Archives of Women's Mental Health
Wong, Y. L. I. : Kong, D. : Tu, L. : Frasso, R.	2018	"My bitterness is deeper than the ocean": Understanding internalized stigma from the perspectives of persons with schizophrenia and their family caregivers	International Journal of Mental Health Systems
Wong-Anuchit, C. : Mills, A. C. : Schneider, J. K. : Rujkorakarn, D. : Kerdpongbunchote, C. : Panyayong, B.	2016	Internalized Stigma of Mental Illness Scale - Thai Version: Translation and Assessment of Psychometric Properties Among Psychiatric Outpatients in Central Thailand	Archives of psychiatric nursing
Wood, Andrew : Barden, Sejal : Terk, Mitchell : Cesaretti, Jamie	2019	Prostate cancer: the influence of stigma on quality of life and relationship satisfaction for survivors and their partners	Journal of Psychosocial Oncology
Wood, L. : Byrne, R. : Enache, G. : Morrison, A. P.	2018	A brief cognitive therapy intervention for internalised stigma in acute inpatients who experience psychosis: A feasibility randomised controlled trial	Psychiatry Research
Woon, L. : Khoo, S. : Baharudin, A. : Midin, M.	2020	Association between insight and internalized stigma and other clinical factors among patients with depression: A cross-sectional study	Indian Journal of Psychiatry
Wouters, E. : Masquillier, C. : le Roux Booysen, F.	2016	The Importance of the Family: A Longitudinal Study of the Predictors of Depression in HIV Patients in South Africa	AIDS and behavior
Wright, K. : Naar-King, S. : Lam, P. : Templin, T. : Frey, M.	2007	Stigma scale revised: reliability and validity of a brief measure of stigma for HIV+ youth	Journal of Adolescent Health
Wu, D. Y. : Munoz, M. : Espiritu, B. : Zeladita, J. : Sanchez, E. : Callacna, M. : Rojas, C. : Arevalo, J. : Caldas, A. : Shin, S.	2008	Burden of depression among impoverished HIV-Positive women in Peru	Journal of Acquired Immune Deficiency Syndromes
Wu, T. H. : Chang, C. C. : Chen, C. Y. : Wang, J. D. : Lin, C. Y.	2015	Further psychometric evaluation of the Self-Stigma Scale-Short: Measurement invariance across mental illness and gender	PLoS ONE
Wu, X. : Hu, Y. : Hu, A.	2022	Living with stigma and low self-esteem among individuals with burn injuries: a cross-sectional study	Journal of burn care & research : official publication of the American Burn Association
Wu, Y. : Guo, X. Y. : Wei, Q. Q. : Song, W. : Chen, K. : Cao, B. : Ou, R. W. : Zhao, B. : Shang, H. F.	2014	Determinants of the quality of life in Parkinson's disease: Results of a cohort study from Southwest China	Journal of the Neurological Sciences
Wu, Y. P. : Follansbee-Junger, K. : Rausch, J. : Modi, A.	2014	Parent and family stress factors predict health-related quality in pediatric patients with new-onset epilepsy	Epilepsia
Xiao, X. : Yang, X. : Zheng, W. : Wang, B. : Fu, L. : Luo, D. : Hu, Y. : Ju, N. : Xu, H. : Fang, Y. : Fong Chan, P. S. : Xu, Z. : Chen, P. : He, J. : Zhu, H. : Tang, H. : Huang, D. : Hong, Z. : Hao, Y. : Cai, L. : Ye, S. : Yuan, J. : Xiao, F. : Yang, J. : Wang, Z. : Zou, H.	2022	Depression, anxiety and post-traumatic growth among COVID-19 survivors six-month after discharge	European journal of psychotraumatology

Xiaohua, Wu : Jia, Chen : Huigen, Huang : Ziping, Liu : Xianhong, Li : Honghong, Wang	2015	Perceived stigma, medical social support and quality of life among people living with HIV/AIDS in Hunan, China	Applied Nursing Research
Xu, M. : Markström, U. : Lyu, J. : Xu, L.	2017	Survey on tuberculosis patients in rural areas in China: Tracing the role of stigma in psychological distress	International Journal of Environmental Research and Public Health
Xu, Xiaohua : Sheng, Yu : Khoshnood, Kaveh : Clark, Kirsty	2017	Factors Predicting Internalized Stigma Among Men Who Have Sex with Men Living with HIV in Beijing, China	JANAC: Journal of the Association of Nurses in AIDS Care
Yan, H. : Li, X. : Li, J. : Wang, W. : Yang, Y. : Yao, X. : Yang, N. : Li, S.	2019	Association between perceived HIV stigma, social support, resilience, self-esteem, and depressive symptoms among HIV-positive men who have sex with men (MSM) in Nanjing, China	AIDS Care - Psychological and Socio-Medical Aspects of AIDS/HIV
Yan, M. H. : Fan, Y. Y. : Zhang, J. E.	2022	Stigma, self-efficacy and late toxicities among Chinese nasopharyngeal carcinoma survivors	European journal of cancer care
Yan, X. J. : Luo, Q. Q. : Qiu, H. Y. : Ji, C. F. : Chen, S. L.	2021	The impact of stigma on medication adherence in patients with functional dyspepsia	Neurogastroenterology and Motility
Yang, L. H. : Grivel, M. M. : Anderson, B. : Bailey, G. L. : Opler, M. : Wong, L. Y. : Stein, M. D.	2019	A new brief opioid stigma scale to assess perceived public attitudes and internalized stigma: Evidence for construct validity	Journal of Substance Abuse Treatment
Yang, Lawrence H. : Ho-Foster, Ari R. : Becker, Timothy D. : Misra, Supriya : Rampa, Shathani : Poku, Ohemaa B. : Entaile, Patlo : Goodman, Melody : Blank, Michael B.	2021	Psychometric Validation of a Scale to Assess Culturally-Salient Aspects of HIV Stigma Among Women Living with HIV in Botswana: Engaging "What Matters Most" to Resist Stigma	AIDS & Behavior
Yang, N. : Xiao, H. : Wang, W. : Li, S. : Yan, H. : Wang, Y.	2018	Effects of doctors' empathy abilities on the cellular immunity of patients with advanced prostate cancer treated by orchiectomy: The mediating role of patients' stigma, self-efficacy, and anxiety	Patient Preference and Adherence
Yang, Q. Q. : Liu, H. X. : Yang, C. L. : Ji, S. Y. : Li, L.	2014	Reliability and validity of chinese version of cataldo lung cancer stigma scale	International Journal of Nursing Sciences
Yang, X. : Mak, W. W. S. : Ho, C. Y. Y. : Chidgey, A.	2017	Self-in-love versus self-in-stigma: implications of relationship quality and love attitudes on self-stigma and mental health among HIV-positive men having sex with men	AIDS Care - Psychological and Socio-Medical Aspects of AIDS/HIV
Yanos, P. T. : Lysaker, P. H. : Silverstein, S. M. : Vayshenker, B. : Gonzales, L. : West, M. L. : Roe, D.	2019	A randomized-controlled trial of treatment for self-stigma among persons diagnosed with schizophrenia-spectrum disorders	Social psychiatry and psychiatric epidemiology
Yanos, P. T. : West, M. L. : Gonzales, L. : Smith, S. M. : Roe, D. : Lysaker, P. H.	2012	Change in internalized stigma and social functioning among persons diagnosed with severe mental illness	Psychiatry Res.
Yates, T. : Yates, S. : Rushing, J. : Schafer, K. R.	2018	Effects of Religious Involvement on HIV Management Outcomes among HIV-Positive Adults in Central North Carolina	Southern Medical Journal
Yator, O. : Mathai, M. : Vander Stoep, A. : Rao, D. : Kumar, M.	2016	Risk factors for postpartum depression in women living with HIV attending prevention of mother-to-child transmission clinic at Kenyatta National Hospital, Nairobi	AIDS Care - Psychological and Socio-Medical Aspects of AIDS/HIV
Yator, O. : Mathai, M. : Albert, T. : Kumar, M.	2020	Burden of HIV-Related Stigma and Post-Partum Depression: A Cross-Sectional Study of Patients Attending Prevention of Mother-to-Child Transmission Clinic at Kenyatta National Hospital in Nairobi	Frontiers in Psychiatry
Yazla, E. : Inanç, L. : Bilici, M.	2012	Relationship of sociodemographic features, clinical symptoms and functioning level among bipolar patients with manic episode and difference between these variables according to gender	Dusunen Adam
Yen, C. F. : Chen, C. C. : Lee, Y. : Tang, T. C. : Ko, C. H. : Yen, J. Y.	2005	Insight and correlates among outpatients with depressive disorders	Comprehensive Psychiatry
Yen, C. F. : Chen, C. C. : Lee, Y. : Tang, T. C. : Yen, J. Y. : Ko, C. H.	2005	Self-stigma and its correlates among outpatients with depressive disorders	Psychiatric Services
Yeni, K. : Tulek, Z. : Bebek, N. : Dede, O. : Gurses, C. : Baykan, B. : Gokyigit, A.	2016	Attitudes towards epilepsy among a sample of Turkish patients with epilepsy	Epilepsy and Behavior
Yeni, K. : Tulek, Z. : Bebek, N.	2016	Factors associated with perceived stigma among patients with epilepsy in Turkey	Epilepsy and Behavior
Yeni, K. : Tulek, Z. : Simsek, O. F. : Bebek, N.	2018	Relationships between knowledge, attitudes, stigma, anxiety and depression, and quality of life in epilepsy: A structural equation modeling	Epilepsy and Behavior
Yi, M. : Li, J. : Liu, G. : Ou, Z. : Liu, Y. : Li, J. : Chen, Y. : Guo, Y. : Wang, Y. : Zhang, W. : Zeng, J. : Dang, C.	2022	Mental health and quality of life in patients with craniofacial movement disorders: A cross-sectional study	Front. Neurol.
Yi, S. : Ngin, C. : Tuot, S. : Chhoun, P. : Fleming, T. : Brody, C.	2017	Utilization of traditional, complementary and alternative medicine and mental health among patients with chronic diseases in primary health care settings in Cambodia	International Journal of Mental Health Systems
Yigit, I. : Bayramoglu, Y. : Weiser, S. D. : Johnson, M. O. : Mugavero, M. J. : Turan, J. M. : Turan, B.	2020	Changes in Internalized Stigma and HIV Health Outcomes in Individuals New to HIV Care: The Mediating Roles of Depression and Treatment Self-Efficacy	AIDS Patient Care and STDs
Yigit, I. : Bayramoglu, Y. : Weiser, S. D. : Johnson, M. O. : Mugavero, M. J. : Turan, J. M. : Turan, B.	2021	Disclosure Concerns and Viral Suppression in People Newly Initiating HIV Care: The Role of Internalized HIV Stigma	Journal of acquired immune deficiency syndromes (1999)
Yildirim, Arzu : Hacihasanoglu Asilar, Rabia : Camcioglu, Tuba Hale : Erdiman, Sezgin : Karaagac, Ebru	2015	Effect of Psychosocial Skills Training on Disease Symptoms, Insight, Internalized Stigmatization, and Social Functioning in Patients with Schizophrenia	Rehabilitation nursing : the official journal of the Association of Rehabilitation Nurses
Yildirim, T. : Kavak Budak, F.	2020	The relationship between internalized stigma and loneliness in patients with schizophrenia	Perspectives in psychiatric care
Yildiz, M. İ : Başar, K. : Karadağ Çaman, Ö : İnkaya, A. Ç	2021	The reliability and validity of the Turkish form of berger HIV stigma scale	Noropsikiyatri Arsivi
Yilmaz, A. : Dedeli, O.	2016	Assessment of anxiety, depression, loneliness and stigmatization in patients with tuberculosis	Acta Paul. Enferm.
Yilmaz, Medine : Dissiz, Gulcin : Demir, Filiz : Iriz, Sibel : Alacacioglu, Ahmet	2017	Reliability and Validity Study of a Tool to Measure Cancer Stigma: Patient Version	Asia-Pacific journal of oncology nursing
Yimer, S. : Girma, Z. : Mengistu, N. : Shumye, S.	2021	Determinants of common mental disorders among people living with hiv/aids attending art service in southern Ethiopia	HIV/AIDS - Research and Palliative Care
Yimer Tawiye, N. : Mekonnen Assefa, Z. : Gizeyatu Zengye, A.	2021	Patient satisfaction and associated factors among adults attending ART clinic at Dessie refferal Hospital, Amhara Region, Ethiopia	International Journal of Africa Nursing Sciences
Yin, Xiaoxv : Yan, Shijiao : Tong, Yeqing : Peng, Xin : Yang, Tingting : Lu, Zuxun : Gong, Yanhong	2018	Status of tuberculosis-related stigma and associated factors: a cross-sectional study in central China	Tropical medicine & international health : TM & IH
Yin, Yi : Zhang, Weijun : Hu, Zhenyu : Jia, Fujun : Li, Yafang : Xu, Huiwen : Zhao, Shuliang : Guo, Jing : Tian, Donghua : Qu, Zhiyong	2014	Experiences of stigma and discrimination among caregivers of persons with schizophrenia in China: a field survey	PloS one
Yip, C. C. H. : Chan, K. K. S.	2022	Longitudinal impact of public stigma and courtesy stigma on parents of children with autism spectrum disorder: The moderating role of trait mindfulness	Res. Dev. Disabil.
Yıldırım, Z. : Ertem, D. H. : Ceyhan Dirican, A. : Baybaş, S.	2018	Stigma accounts for depression in patients with epilepsy	Epilepsy and Behavior
Yıldız, K. : Koç, Z.	2021	Stigmatization, discrimination and illness perception among oncology patients: A cross-sectional and correlational study	European journal of oncology nursing : the official journal of European Oncology Nursing Society
Yıldız, M. : Demir, Y. : Kırcalı, A. : Incedere, A.	2021	Caregiver Burden in Schizophrenia and Autism Spectrum Disorders: A Comparative Study	Psychiatry Investigation
Yıldız, M. : İncedere, A. : Kiras, F. : Abut, F. B. : Kırcalı, A. : İpçi, K.	2019	Development of Self-Stigma Inventory for Families of the patients with schizophrenia (SSI-F): validity and reliability study	Psychiatry and Clinical Psychopharmacology
Yıldız, M. : Kiras, F. : İncedere, A. : Abut, F. B.	2019	Development of self-stigma inventory for patients with schizophrenia (SSI-P): reliability and validity study	Psychiatry and Clinical Psychopharmacology
Yılmaz, E. : Kavak, F.	2020	Effects of Mindfulness-Based Psychoeducation on the Internalized Stigmatization Level of Patients With Schizophrenia	Clinical nursing research
Yoo, S. H. : Kim, S. R. : So, H. S. : Chung, H. I. C. : Chae, D. H. : Kim, M. K. : Kim, B. C. : Park, M. S. : Lee, S. H. : Nam, T. S. : Correia, H. : Cella, D.	2017	The Validity and Reliability of the Korean Version of the Stigma Scale for Chronic Illness 8-Items (SSCI-8) in Patients with Neurological Disorders	International journal of behavioral medicine
Yoo, T. : Kim, S. W. : Kim, S. Y. : Lee, J. Y. : Kang, H. J. : Bae, K. Y. : Kim, J. M. : Shin, I. S. : Yoon, J. S.	2015	Relationship between suicidality and low self-esteem in patients with schizophrenia	Clinical Psychopharmacology and Neuroscience
Yoshii, H. : Mandai, N. : Saito, H. : Akazawa, K.	2015	Reliability and validity of the workplace social distance scale	Global journal of health science
Young, W. B. : Park, J. E. : Tian, I. X. : Kempner, J.	2013	The Stigma of Migraine	PLoS ONE
Yousaf, A. : Adil, A. : Niazi, S. : Ghayas, S. : Khan, A. : Rafique, N.	2021	Mediating role of social isolation between stigma and depression among hepatitis patients	Rawal Medical Journal
Yousuf, A. : Musa, R. : Isa, M. L. M. : Arifin, S. R. M.	2020	Anxiety and depression among women living with hiv: Prevalence and correlations	Clinical Practice and Epidemiology in Mental Health
Yow, T. S. : Mehta, K.	2010	Perceived stigma and coping strategies among Asians with schizophrenia: the Singapore case	International Social Work
Yozgat, A. : Can, G. : Can, H. : Ekmen, N. : Akyol, T. : Kasapoglu, B. : Kekilli, M.	2021	Social stigmatization in Turkish patients with chronic hepatitis B and C	Gastroenterologia y Hepatologia
Yu, Chia-Hui : Huang, Chu-Yu : Lee, Yuan-Ti : Cheng, Su-Fen	2019	Development of an 18-item abbreviated Chinese version of Berger's HIV Stigma Scale	International Journal of Nursing Practice (John Wiley & Sons, Inc.)
Yu, E. : Adams-Clark, A. : Riehm, A. : Franke, C. : Susukida, R. : Pinto, M. : Arenberg, S. : Tosi, D. : Hughes, A. : Montague, A. : Kumar, A. : Jamison, K. : Kaplin, A.	2021	Perspectives on illness-related stigma and electronically sharing psychiatric health information by people with multiple sclerosis	Journal of Affective Disorders
Yuan, Jing Min : Zhang, Jun E. : Zheng, Mei Chun : Bu, Xiu Qing	2018	Stigma and its influencing factors among Chinese patients with stoma	Psycho-oncology
Yuan, L. : Pan, B. : Wang, W. : Wang, L. : Zhang, X. : Gao, Y.	2020	Prevalence and predictors of anxiety and depressive symptoms among patients diagnosed with oral cancer in China: a cross-sectional study	BMC Psychiatry
Yuksel, B. : Genc, F. : Yaman, A. : Goksu, E. O. : Ak, P. D. : Gomceli, Y. B.	2019	Evaluation of stigmatization in hemifacial spasm and quality of life before and after botulinum toxin treatment	Acta Neurologica Belgica
Yun, Y. S. : Syed Jaapar, S. Z. : Fadzil, N. A. : Cheng, K. Y.	2018	Validation of the malay version of the affiliate stigma scale among caregivers of patients with mental illness	Malaysian Journal of Medical Sciences

Zacks, S. : Beavers, K. : Theodore, D. : Dougherty, K. : Batey, B. : Shumaker, J. : Galanko, J. : Shrestha, R. : Fried, M. W.	2006	Social stigmatization and hepatitis C virus infection	Journal of Clinical Gastroenterology
Zafra-Tanaka, J. H. : Ticona-Chavez, E.	2016	Stigma related to HIV/AIDS associated with adherence to antiretroviral therapy in patients of a public hospital in Lima, Peru 2014	Revista Peruana de Medicina Experimental y Salud Publica
Zamanian, Hadi : Amini-Tehrani, Mohammadali : Jalali, Zahra : Daryaafzoon, Mona : Ramezani, Fatemeh : Malek, Negin : Adabimohazab, Maede : Hozouri, Roghayeh : Rafiei Taghanaky, Fereshteh	2022	Stigma and Quality of Life in Women With Breast Cancer: Mediation and Moderation Model of Social Support, Sense of Coherence, and Coping Strategies	Frontiers in psychology
Zang, C. : Guida, J. : Sun, Y. : Liu, H.	2014	Collectivism culture, HIV stigma and social network support in Anhui, China: A path analytic model	AIDS Patient Care STDs
Zarei, M. : Roohafza, H.	2018	Examining the effect of cognitive-behavioral family therapy on social stigma in family with children suffering from sickle cells in manujan in 2016	Electronic Journal of General Medicine
Zarei, N. : Joulaei, H.	2018	The Impact of perceived stigma, quality of life, and spiritual beliefs on suicidal ideations among HIV-positive patients	AIDS Research and Treatment
Zäske, H. : Linden, M. : Degner, D. : Jockers-Scherübl, M. : Klingberg, S. : Klosterkötter, J. : Maier, W. : Möller, H. J. : Sauer, H. : Schmitt, A. : Gaebel, W.	2019	Stigma experiences and perceived stigma in patients with first-episode schizophrenia in the course of 1 year after their first in-patient treatment	European Archives of Psychiatry and Clinical Neuroscience
Zelaya, Carla E. : Sivaram, Sudha : Johnson, Sethulakshmi C. : Srikrishnan, A. K. : Suniti, Solomon : Celentano, David D.	2012	Measurement of self, experienced, and perceived HIV/AIDS stigma using parallel scales in Chennai, India	AIDS Care
Zeller, M. H. : Ingerski, L. M. : Wilson, L. : Modi, A. C.	2010	Factors contributing to weight misperception in obese children presenting for intervention	Clinical Pediatrics
Zewude, S. B. : Ajebe, T. M.	2022	Magnitude of optimal adherence and predictors for a low level of adherence among HIV/AIDS-infected adults in South Gondar zone, Northwest Ethiopia: a multifacility cross-sectional study	BMJ Open
Zhang, C. : Li, X. : Liu, Y. : Qiao, S. : Zhou, Y. : Shen, Z. : Chen, Y.	2016	Substance use and psychosocial status among people living with HIV/AIDS who encountered HIV stigma in China: Stratified analyses by socio-economic status	PLoS ONE
Zhang, Chen : Li, Xiaoming : Liu, Yu : Qiao, Shan : Zhang, Liying : Zhou, Yuejiao : Shen, Zhiyong : Tang, Zhengzhu	2016	Emotional, physical and financial burdens of stigma against people living with HIV/AIDS in China	AIDS Care
Zhang, H. : Zhong, R. : Chen, Q. : Guo, X. : Han, Y. : Zhang, X. : Lin, W.	2021	Depression severity mediates the impact of perceived stigma on quality of life in patients with epilepsy	Epilepsy and Behavior
Zhang, L. J. : Shannon, K. : Tibashoboka, D. : Ogilvie, G. : Pick, N. : Kestler, M. : Logie, C. : Udall, B. : Braschel, M. : Deering, K. N.	2021	Prevalence and correlates of having sexual and reproductive health priorities met by HIV providers among women living with HIV in a Canadian setting	Sexual and Reproductive Healthcare
Zhang, Liying : Li, Xiaoming : Qiao, Shan : Zhou, Yuejiao : Shen, Zhiyong : Tang, Zhenzhu : Shah, Iqbal : Stanton, Bonita	2015	The mediating role of individual resilience resources in stigma-health relationship among people living with HIV in Guangxi, China	AIDS Care
Zhang, N. : Lai, F. : Guo, Y. : Wang, L.	2022	Status of and Factors Influencing the Stigma of Chinese Young and Middle-Aged Maintenance Hemodialysis Patients: A Preliminary Study	Front. Psychol.
Zhang, T. M. : Wong, I. Y. L. : Yu, Y. H. : Ni, S. G. : He, X. S. : Bacon-Shone, J. : Gong, K. : Huang, C. H. : Hu, Y. : Tang, M. M. : Cao, W. : Chan, C. L. W. : Ran, M. S.	2019	An integrative model of internalized stigma and recovery-related outcomes among people diagnosed with schizophrenia in rural China	Social psychiatry and psychiatric epidemiology
Zhang, Xiaomei : Li, Yanqing : Zhong, Yueping : Wang, Ziheng	2022	Variables Associated With Body Image Concerns in Acromegaly Patients: A Cross-Sectional Study	Frontiers in psychology
Zhang, Y. B. : Yang, Z. : Zhang, H. J. : Xu, C. Q. : Liu, T.	2022	The role of resilience in diabetes stigma among young and middle-aged patients with type 2 diabetes	Nursing open
Zhang, Y. : Cui, C. : Wang, Y. : Wang, L.	2020	Effects of stigma, hope and social support on quality of life among Chinese patients diagnosed with oral cancer: A cross-sectional study	Health and Quality of Life Outcomes
Zhang, Y. : Guo, X. : Guo, J. : Wang, L. : Zhao, H. : Wang, Y. : Wang, J. : Sun, X. : Jiang, W. : Liu, G. : Xiao, Z. : Cong, N. : Qi, J. : Han, S. : Wang, Z. : Gao, L. : Bao, X. : Feng, M. : Yao, Y. : Deng, K. : Lian, W. : Xing, B.	2020	Sleep quality in acromegaly and changes after transsphenoidal surgery: a prospective longitudinal study	Sleep Medicine
Zhang, Y. : Guo, X. : Wang, L. : Guo, J. : Zhao, H. : Sun, S. : Sun, Y. : Xu, D. : Wang, Z. : Gao, L. : Feng, M. : Xing, B.	2020	Pre- And Postoperative Health Status of Patients with Nonfunctioning and Secretory Pituitary Adenomas and an Analysis of Related Factors	International Journal of Endocrinology
Zhang, Yu-Jing : Fan, Yin-Guang : Dai, Se-Ying : Li, Bao-Zhu : Xu, Wang-Dong : Hu, Lin-Feng : Liu, Juan : Su, Hong : Ye, Dong-Qing	2015	HIV/ AIDS stigma among older PLWHA in south rural China	International Journal of Nursing Practice (John Wiley & Sons, Inc.)
Zhang, Y. : Subramaniam, M. : Lee, S. P. : Abdin, E. : Sagayadevan, V. : Jeyagurunathan, A. : Chang, S. : Shafie, S. B. : Abdul Rahman, R. F. : Vaingankar, J. A. : Chong, S. A.	2018	Affiliate stigma and its association with quality of life among caregivers of relatives with mental illness in Singapore	Psychiatry research
Zhang, Y. : Zhao, J. : Jiang, N. : Liu, Y. : Wang, T. : Yu, X. : Wang, J. : Yu, J.	2022	The association between comorbidities and stigma among breast cancer survivors	Scientific reports
Zhang, Y. : Zhao, J. : Jiang, N. : Wang, T. : Cao, H. : Wang, Q. : Wei, X. : Wang, J. : Yu, J.	2022	Effects of Stigma on the Relationship Between Perceived Social Support and Sexual Satisfaction Among Breast Cancer Survivors	Journal of Sexual Medicine
Zhang, Z. : Azman, N. : Eyu, H. T. : Nik Jaafar, N. R. : Mohd Salleh Sahimi, H. : Mohamad Yunus, M. R. : Mohd Shariff, N. : Hami, R. : Mansor, N. S. : Lu, P. : Leong Bin Abdullah, M. F. I.	2022	Validation of the Malay Version of the Shame and Stigma Scale among Cancer Patients in Malaysia	International Journal of Environmental Research and Public Health
Zhao, M. : Liu, B. : Zheng, T. : Xu, J. : Hao, Y. : Wang, J. : Zhang, X. : Nie, W. : Wang, C. : Wang, F. : Jiao, M. : Wu, Q. : Liang, L.	2019	Factors associated with hostility among people living with HIV/AIDS in Northeast China: a cross-sectional study	BMC public health
Zhao, Y. : Liu, X. : Xiao, Z.	2021	Effects of perceived stigma, unemployment and depression on suicidal risk in people with epilepsy	Seizure
Zheng, L. : Jin, Q.	2022	Roy adaptation model-based nursing diagnosis and implementation reduces the sense of shame and enhances nursing outcomes in female patients with breast cancer	American Journal of Translational Research
Zhu, B. : Kohn, R. : Patel, A. : Koo, B. B. : Louis, E. D. : De Figueiredo, J. M.	2021	Demoralization and Quality of Life of Patients with Parkinson Disease	Psychotherapy and Psychosomatics
Zhu, L. : Yao, J. : Wu, L. : Wang, J. : Qiu, M. : Zhang, C. : Zhang, H. : Xie, J. : Liu, A. : Ranchor, A. : Schroevers, M.	2019	Psychometric properties of the Depression Stigma Scale (DSS) in Chinese cancer patients: a cross-sectional study	BMJ open
Zhu, M. : Cai, W. : Li, L. : Guo, Y. : Monroe-Wise, A. : Li, Y. : Zeng, C. : Qiao, J. : Xu, Z. : Zhang, H. : Zeng, Y. : Liu, C.	2019	Mediators of Intervention Effects on Depressive Symptoms Among People Living With HIV: Secondary Analysis of a Mobile Health Randomized Controlled Trial Using Latent Growth Curve Modeling	JMIR mHealth and uHealth
Zhu, M. : Zhou, H. : Zhang, W. : Deng, Y. : Wang, X. : Zhang, X. : Yang, L. : Li, M. : Bai, X. : Lin, Z.	2019	Stigma experienced by Chinese patients with stroke during inpatient rehabilitation and its correlated factors: a cross-sectional study	Topics in Stroke Rehabilitation
Zhu, R. : Zhang, L. : Zheng, Y. H. : Zhang, Z. H. : Zhang, L. M. : Yang, H. L. : Yue, Y. : Xiong, X.	2022	Association between Stigma and Pain in Patients with Temporomandibular Disorders	International Journal of Clinical Practice
Zhuang, H. : Wang, L. : Yu, X. : Chan, S. W. C. : Gao, Y. : Li, X. : Gao, S. : Zhu, J.	2022	Effects of decisional conflict, decision regret and self-stigma on quality of life for breast cancer survivors: A cross-sectional, multisite study in China	Journal of advanced nursing
Ziegler, S. : Bednasch, K. : Baldofski, S. : Rummel-Kluge, C.	2021	Long durations from symptom onset to diagnosis and from diagnosis to treatment in obsessive-compulsive disorder: A retrospective self-report study	PLoS ONE
Zou, X. : Hong, Z. : Chen, J. : Zhou, D.	2014	Is antiepileptic drug withdrawal status related to quality of life in seizure-free adult patients with epilepsy?	Epilepsy and Behavior
Zulkarnain, Z. : Tuapattinaja, J. M. R. : Yurliani, R. : Iskandar, R.	2020	Psychological well-being of housewives living with HIV/AIDS: Stigma and forgiveness	HIV and AIDS Review
	2018	Impact of Stigma on Compliance to Medication in Functional Dyspepsia	
	2023	Assessment of stigma among caregivers of persons with mental illness in armed forces population	Indian Journal of Psychiatry
Abate, A. W. : Menberu, M. : Belete, H. : Ergetie, T. : Teshome, A. A. : Chekol, A. T. : Aschale, M. : Desalegn, W.	2023	Perceived compassionate care and associated factors among patients with mental illness at Tibebe Ghion specialized and Felege Hiwot comprehensive specialized hospital, Northwest Ethiopia	BMC health services research
Abbas, Q. : Nisa, M. : Khan, M. U. : Anwar, N. : Aljhani, S. : Ramzan, Z. : Shahzadi, M.	2023	Brief cognitive behavior therapy for stigmatization, depression, quality of life, social support and adherence to treatment among patients with HIV/AIDS: a randomized control trial	BMC Psychiatry
Abdollahi, F. : Ariyan, E. : Rastegar, F. : Sepasi, R. R. : Motalebi, S. A.	2024	Relationships Between Perceived Stigma and Coping Strategies in Patients with Multiple Sclerosis	Jundishapur. J. Chronic. Dis. Care.
Abdul Taib, N. I. : Nik Jaafar, N. R. : Azman, N. : Leong Bin Abdullah, M. F. I. : Mohamad Kamal, N. A. : Baharudin, A. : Bin Abdullah, M. N. : Chidambaram, S. K. : Adlan, A. : Tan, L. H. : Tamilselvam, S. : Mohamed Said, M. S. : Abd Samad, A. : Binti Dollah, S. N.	2023	Stigma, Sociodemographic Factors, and Clinical Factors Associated with Psychological Distress among COVID-19 Survivors during the Convalescence Period: A Multi-Centre Study in Malaysia	International Journal of Environmental Research and Public Health
Adhikari, Kaushik : Dutt, Debashis : Pal, Dipak : Hazra, Suprakas	2022	Stigma and its associated factors among people living with HIV/AIDS (PLHA) attending art centre in a tertiary care institute in Kolkata	MAMC Journal of Medical Sciences
Aduloju-Ajijola, N. : Oyerinde, I. A. : Gaul, Z. J. : Gaskins, S. : Sutton, M. Y. : Cody, S. L. : Johnson, K. : Mugoya, G. C. T. : Payne-Foster, P.	2024	HIV Knowledge among African Americans Living with HIV in the Rural South: Implications for Improving HIV Prevention and Care Outcomes	Journal of racial and ethnic health disparities

Afriyie-Adjimi, H. : Nkyi, A. K.	2024	Impact of body image and perceived stigmatization on the psychological wellbeing of obese women in Kumasi metropolis, Ghana	PLoS ONE
Afzal, M. I. : Jamshaid, S. : Wang, L. : Lo-Ngoen, N. : Olorundare, A. : Iqbal, M. : Amin, R. : Younas, R. : Naz, S.	2023	Stigmatization, panic disorder, and death anxiety among patients of Covid-19: Fourth wave of pandemic in Pakistan	Acta psychologica
Agu, K. M. : Eze, J. N. : Muoneke, U. V. : Uwaezuoke, S. N.	2023	Prevalence and determinants of psychosocial disorders in children and adolescents with asthma in Enugu, Nigeria: a comparative cross-sectional study	Journal of Asthma
Ahmad, M. A. : Mohamad Nor, A. : Abd Hamid, H. S.	2024	HIV Stigma, Sexual Identity Stigma and Online Coping Strategy of Gay, Bisexual and Queer People Living with HIV: A Moderated Mediation Study	Current HIV research
Ahmed, Saba : Mendez, Rosario Yslado : Naveed, Shaheryar : Akhter, Shoaib : Mushtaque, Iqra : Malik, Mareen A. : Ahmad, Waqar : Figueroa, Roger Norabuena : Younas, Ammar	2023	Assessment of hepatitis-related knowledge, attitudes, and practices on quality of life with the moderating role of internalized stigma among hepatitis B-positive patients in Pakistan	Health psychology and behavioral medicine
Ahmed-Chowdhury, S. : Ahmad, S. : Sarker, M. M. R. : Ismail, N. E.	2024	Assessment of depression and internalized-stigma among adult asthma patients in Bangladesh: a cross-sectional study	Discov. Social Sci. Health
Ahn, S. H. : Kim, J. L. : Lee, S. H. : Park, H. Y. : Lee, J. J. : Lee, H.	2022	Associations of health-related quality of life with depression and stigma in MERS-CoV survivors during the recovery period	Medicine
Akoko, B. : Regan, S. : Idigbe, I. : Ezechi, O. : Pierce, L. J. : Musa, Z. : Okonkwo, P. : Freedberg, K. A. : Ahonkhai, A. A.	2024	HIV-related stigma and psychological distress in a cohort of patients receiving antiretroviral therapy in Nigeria	AIDS Care - Psychological and Socio-Medical Aspects of AIDS/HIV
Al-Shdaifat, A. : Al Tarifi, A. : Al-Shudifat, A. E. : Colton, B. : Taha, H.	2023	Prevalence of Acute Post-Traumatic Stress Symptoms and Perceived Stigma among COVID-19 Patients in Jordan	Jordan Medical Journal
Albalawi, W. F. : Albaraki, J. : Alharbi, S. : Ababtain, N. : Aloteibi, R. E. : Alsudais, A. S. : Jamjoom, J. : Alaqeel, M.	2023	Distribution of perceived weight stigma and its psychological impact on obese people in Saudi Arabia	Saudi Pharmaceutical Journal
Albarraq, Rahaf Hamood : Alhujaili, Naseem Abdulmohi : Alshehri, Ziyad Ibrahim : Alqarni, Abdullah Mohammed : Bawareth, Rime Mohammed	2024	Anticipated Stigma among Patients with Multiple Sclerosis in Saudi Arabia	Saudi journal of medicine & medical sciences
Albayrak, Eda : Asi Karakas, Sibel	2022	An investigation of internalized stigma and recovery levels of patients registered in a Community Mental Health Center	Perspectives in psychiatric care
Alblowi, Yassmeen Hmoud : Alsaati, Ahmed A. : Alzubaidi, Amirah Saleh : Alsifri, Sahar Saud : AlHarthi, Yousef : Alotaibi, Moteb Khalaf	2024	Prevalence and Causes of Stigmatization Among Patients With Chronic Skin Diseases in Saudi Arabia	Cureus
AlFattani, A. : Bilal, L. : Saad, S. Y. : Naseem, M. T. : Hyder, S. : Alhabib, A. : Alsubaie, A. : Altwaijri, Y.	2023	Effect of perceived stigma on work and social roles among individuals with mental health disorders in Saudi Arabia: findings from a national survey	Annals of General Psychiatry
Alford, K. : Banerjee, S. : Daley, S. : Hamlyn, E. : Trotman, D. : Vera, J. H.	2023	Health-Related Quality of Life in People Living With HIV With Cognitive Symptoms: Assessing Relevant Domains and Associations	Journal of the International Association of Providers of AIDS Care
AlHamawi, R. : Khader, Y. : Abaza, H. : Satyanarayana, S. : Wilson, N. : Saleh Abu Rumman, A. : Okkah, K.	2024	Tuberculosis-related knowledge, practices, perceived stigma and discrimination among patients with tuberculosis: a cross-sectional study in Jordan	Infect. Dis.
Aliche, C. J. : Ifeagwazi, C. M. : Ozor, C. C.	2022	Psychological flexibility as a moderator of the relationship between HIV-related stigma and resilience among HIV/AIDS patients	Afr. J. AIDS Res.
Alinaitwe, R. : Seggane, M. : Turiho, A. : Bird, V. : Priebe, S. : Sewankambo, N.	2024	Family Psycho-Social Involvement Intervention for severe mental illness in Uganda	S. Afr. J. Psychiatry
Almeida, A. : Ogbonnaya, I. N. : Wanyenze, R. K. : Crockett, K. S. : Ediau, M. : Naigino, R. : Lin, C. D. : Kiene, S. M.	2023	A Psychometric Evaluation and a Framework Test of the HIV Stigma Mechanisms Scale Among a Population-Based Sample of Men and Women Living with HIV in Central Uganda	AIDS Behav.
Alqahtani, S. A. : Alswat, K. : Mawardi, M. : Sanai, F. M. : Abaakhail, F. : Alghamdi, S. : Al-Hamoudi, W. K. : Nader, F. : Stepanova, M. : Younossi, Z. M.	2023	STIGMA IN STEATOTIC LIVER DISEASE: A SURVEY OF PATIENTS FROM SAUDI ARABIA	Saudi Journal of Gastroenterology
Altun, Ozlem Sahin : Ozer, Duygu : Gungor, Zeynep : Sahin, Fatih	2023	Investigation of the Relationship Between Internalized Stigma and Happiness Levels of Individuals Diagnosed with Schizophrenia	Florence Nightingale journal of nursing
Amal, A. I. : Sukartini, T. : Kurniawati, N. D. : Sulistyaningsih, D. R. : Suyanto, S. : Wahyuningsih, I. S.	2024	Analysis of Coping Strategies and Self-Stigma Among People Living with HIV (PLHIV): A Cross Sectional Study	Open Public Health Journal
Anderson, Madeleine : McCracken, Lance M. : Scott, Whitney	2024	An investigation of the associations between stigma, self-compassion, and pain outcomes during treatment based on Acceptance and Commitment Therapy for chronic pain	Frontiers in psychology
Anjuga Elavarasi, E. : Smitha, M. C. : Manasa, K. : Kruthika, B. N.	2023	Depression and stigma experience among patients with tuberculosis in urban and rural settings	Indian Journal of Tuberculosis
Anu Pargavi, J. : Kirubakaran, : Senthil Kumar, R. S. : Rajamanickam, S. : Appanabhotla, P.	2023	A HOSPITAL BASED CROSS-SECTIONAL STUDY ON ASSESSMENT OF STIGMA AMONG TUBERCULOSIS PATIENTS IN RURAL TAMIL NADU	International Journal of Medicine and Public Health
Armstrong, A. W. : Bohannan, B. : Mburu, S. : Coates, L. C. : Ogdie, A. : Alarcon, I. : Kasparek, T. : Frade, S. : Barrio, S. F. : Augustin, M.	2023	Patient Perspectives on Psoriatic Disease Burden: Results from the Global Psoriasis and Beyond Survey	Dermatology
Arora, A. K. : Vicente, S. : Engler, K. : Lessard, D. : Huerta, E. : Ishak, J. : Kronfli, N. : Routy, J. P. : Cox, J. : Lemire, B. : Klein, M. : de Pokomandy, A. : Del Balso, L. : Sebastiani, G. : Vedel, I. : Quesnel-Vallée, A. : Lebouché, B.	2024	Patient-reported outcomes and experiences of migrants enrolled in a multidisciplinary HIV clinic with rapid, free, and onsite treatment dispensation: the 'ASAP' study	AIDS Research and Therapy
Arrab, M. M. : Sabola, N. E. : ElSalamoni, M. A. E. : Shahin, M. A. : Shafik, S. : Doma, N. I.	2024	Psychological Aspects, Coping Strategies and Perceived Social Stigma associated with COVID-19 Pandemic	African Journal of Biological Sciences (South Africa)
Ashager, K. : Feleke, M. G. : Degefu, S. : Elfios, E. : Getnet, A. : Ezo, E. : Sintayehu, M.	2023	Psychological distress and associated factors among asthmatic patients in Southern, Ethiopia, 2021	Asthma Research and Practice
Aslan, E. Ö : Toygar, İ : Feyizoğlu, G. : Polat, S. : Eti Aslan, F.	2023	Relationship between the insulin use and stigma in type 2 diabetes mellitus	Primary Care Diabetes
Assefa, S. : Boru, B. : Gebeyehu, D. A. : Terefe, B.	2023	Depression, anxiety and their associated factors among patients with tuberculosis attending in Gondar city health facilities, North West Ethiopia	BMC Psychiatry
Atan, G. : OruÇ, F. G. : Atan, K.	2024	Assessment of stigmatization and self-esteem in patients with epilepsy	Epilepsy and Behavior
Aung, S. : Hardy, N. : Hogan, J. : DeLong, A. : Kyaw, A. : Tun, M. S. : Aung, K. W. : Kantor, R.	2023	Characterization of HIV-Related Stigma in Myanmar	AIDS Behav.
Azman, N. : Nik Jaafar, N. R. : Leong Bin Abdullah, M. F. I. : Abdul Taib, N. I. : Mohamad Kamal, N. A. : Abdullah, M. N. : Dollah, S. N. : Mohamed Said, M. S.	2023	Stigma and posttraumatic growth among COVID-19 survivors during the first wave of the COVID-19 pandemic in Malaysia: a multicenter cross-sectional study	Frontiers in Psychiatry
Badru, Oluwaseun Abdulganiyu : Babalola, Oluwatobi Emmanuel	2023	Significant Others and Not Family or Friend Support Mediate Between Stigma and Discrimination Among People Living With HIV in Lagos State, Nigeria: A Cross-sectional Study	JANAC: Journal of the Association of Nurses in AIDS Care
Bailey, C. : Agrawal, N. : Cope, S. : Proctor, B. : Mildon, B. : Butler, M. : Holt, K. : Edwards, M. : Poole, N. : Nicholson, T. R.	2024	Illness perceptions, experiences of stigma and engagement in functional neurological disorder (FND): Exploring the role of multidisciplinary group education sessions	BMJ Neurology Open
Bal, N. B. : Karslioğlu, E. H. : Ata, P. E. : Çayköylü, A.	2023	Internalized Stigma and Resistance Against Stigma as Risk Factors for Suicide in Individuals with Schizophrenia: A Cross-Sectional Study	Noropsikiyatri Arsivi
Bann, C. M. : Okoniewski, K. C. : Clarke, L. : Wilson-Costello, D. : Merhar, S. : DeMauro, S. : Lorch, S. : Ambalavanan, N. : Peralta-Carcelen, M. : Limperopoulos, C. : Poindexter, B. : Davis, J. M. : Walsh, M. : Newman, J.	2024	Psychological distress among postpartum women who took opioids during pregnancy: the role of perceived stigma in healthcare settings	Archives of Women's Mental Health
Barré, T. : Ramier, C. : Antwerpes, S. : Costa, M. : Bureau, M. : Maradan, G. : Di Beo, V. : Cutarella, C. : Leloutre, J. : Riccobono-Soulier, O. : Hedoire, S. : Frot, E. : Vernier, F. : Vassas-Goyard, S. : Dufort, S. : Protopopescu, C. : Marcellin, F. : Casanova, D. : Coste, M. : Carrieri, P.	2023	A novel community-based therapeutic education program for reducing alcohol-related harms and stigma in people with alcohol use disorders: A quasi-experimental study (ETHER study)	Drug Alcohol Rev.
Barreras, Joanna L. : Bogart, Laura M. : MacCarthy, Sarah : Klein, David J. : Pantalone, David W.	2023	Discrimination and adherence in a cross-sectional study of Latino sexual minority men with HIV: Coping with discrimination as a mediator and coping self-efficacy as a moderator	Journal of Behavioral Medicine
Barrington, Clare : Goldenberg, Tamar : Donastorg, Yeycy : Gomez, Hoisex : Perez, Martha : Kerrigan, Deanna	2023	Stigma and HIV Treatment Outcomes Among Transgender Women Sex Workers in the Dominican Republic	AIDS & Behavior
Batchelder, A. W. : Heo, M. : Foley, J. D. : Sullivan, M. C. : Lum, P. : Pericot Valverde, I. : Taylor, L. E. : Mehta, S. H. : Kim, A. Y. : Norton, B. : Tsui, J. I. : Feinberg, J. : Page, K. : Litwin, A. H.	2023	Shame and stigma in association with the HCV cascade to cure among people who inject drugs	Drug and Alcohol Dependence
Beainy, C. : Haddad, C. : Fekih-Romdhane, F. : Hallit, S. : Haddad, G.	2023	Decreased insight, but not self-stigma or belief about medicine, is associated with greater severity of delusions in a sample of long-stay patients with schizophrenia: a cross-sectional study	BMC Psychiatry
Bebell, L. M. : Kembabazi, A. : Musinguzi, N. : Martin, J. N. : Hunt, P. W. : Boum, Y. : O'Laughlin, K. N. : Muzoora, C. : Haberer, J. E. : Bwana, M. B. : Bangsberg, D. R. : Siedner, M. J. : Tsai, A. C.	2021	Internalized stigma, depressive symptoms, and the modifying role of antiretroviral therapy: A cohort study in rural Uganda	SSM - Mental Health
Ben-Zeev, D. : Larsen, A. : Attah, D. A. : Obeng, K. : Beaulieu, A. : Asafo, S. M. : Gavi, J. K. : Kadakia, A. : Sottie, E. Q. : Ohene, S. : Kola, L. : Hallgren, K. : Snyder, J. : Collins, P. Y. : Ofori-Atta, A.	2024	Combining mHealth Technology and Pharmacotherapy to Improve Mental Health Outcomes and Reduce Human Rights Abuses in West Africa: Intervention Field Trial	JMIR Mental Health

Bernard, C. : Font, H. : Ziadeh, S. : Tine, J. M. : Diaw, A. : Ndiaye, I. : Samba, O. : Bottai, T. : Jacquesy, L. : Verdeli, H. : Ngom, N. F. : Dabis, F. : Seydi, M. : De Rekeneire, N.	2023	Management of depression in people living with HIV/AIDS in Senegal: Acceptability, feasibility and benefits of group interpersonal therapy	Global Mental Health
Bhatt, Jem : Kohl, Gianna : Scior, Katrina : Charlesworth, Georgina : Muller, Majon : Dröes, Rose-Marie	2023	Comparing the stigma experiences and comfort with disclosure in Dutch and English populations of people living with dementia	Dementia (14713012)
Bibi, F. : Majeed, S. : Nikhet, S. : Makhdoom, B. J. : Mustafa, A. B.	2024	Disease-Related Stigma, Emotional Regulation and Depression in patients with HIV	Pakistan Journal of Medical and Health Sciences
Bień, B. : Krajewski, P. K. : Szepietowski, J. C.	2024	Acne influences life-changing decisions among young adults	Ital. J. Dermatol. Venereol.
Bilgic, A. : Fettahlloglu Karaman, B. : Demirseren, D. D. : Clnar, L. : Kacar, N. : Türel Ermertcan, A. : Bulbul Baskan, E. : Güven, M. : Yazici, S. : Özkök Akbulut, T. : Ucmak, D. : Ataseven, A. : Temiz, S. A. : Engin, B. : Şikar Aktürk, A. : Sarlcaoğlu, H. : Güler Özden, M. : Özdemir, H. : Mammadli, K. : Durmaz, K. : Gülbaşaran, F. : Klllnc, F. : Kaya Özden, H. : Gönülal, M. : Didar Balcl, D. : Çevirgen Cemil, B. : Sarlkaya Solak, S. : Baykal Selçuk, L. : Özaydln Yavuz, G. : Yavuz, Ä H. : Dönmez, L. : Alpsoy, E.	2023	Internalized Stigma in Hidradenitis Suppurativa: A Multicenter Cross-Sectional Study	Dermatology
Bilgin Koçak, M. : Öztürk Atkaya, N.	2024	The Relationship Between Internalized Stigma with Self-reported Cognitive Dysfunction and Insight in Schizophrenia	Psychiatry and Clinical Psychopharmacology
Billian, J. : Imfeld, L. : Roth, C. B. : Moeller, J. : Lang, U. E. : Huber, C. G.	2024	Patient-reported experience measures (PREMs) in outpatient psychiatry - is there an association to perceived discrimination and devaluation?	Frontiers in Psychiatry
Billian, J. : Imfeld, L. : Roth, C. B. : Moeller, J. : Lang, U. E. : Huber, C. G.	2024	Treatment-seeking threshold and accessibility of psychiatric outpatient services in Switzerland: the relationship with stigma and self-esteem	Frontiers in Psychiatry
Bin Ahmad, Mohd Zulfikry : Md Yasin, Mazapuspavina : Mat Nasir, Nafiza : Mohamad, Mariam	2024	The association between HIV-related stigma, HIV knowledge and HIV late presenters among people living with HIV (PLHIV) attending public primary care clinic settings in Selangor	PloS one
Bint-e-Saif, S. : Shahzad, S.	2023	Importance of perceived social support for HIV/AIDS patients in Pakistan: a collectivist society	Journal of Substance Use
Bobevski, I. : Kissane, D. W. : Desroches, J. : De Simone, A. : Henry, M.	2023	Validation of the Canadian Version of the Shame and Stigma Scale for Head and Neck Cancer Patients	Current Oncology
Boersma-van Dam, E. : Spronk, I. : Hofland, H. W. C. : Van Loey, N. E. E.	2024	Gender differences in relation to stigmatization and depressive symptoms after burns	Burns
Borhade, S. R. : Mishra, K. K. : John, S.	2024	A Comparative Study of Internalized Stigma in Patients with Schizophrenia and Bipolar Affective Disorder in Remission	Indian J. Psychol. Med.
Boyle, M. P. : Cheyne, M. R.	2024	Major discrimination due to stuttering and its association with quality of life	J. Fluency Disord.
Brittain, Kirsty : Brown, Karryn : Phillips, Tamsin : Zerbe, Allison : Pellowski, Jennifer : Remien, Robert H. : Mellins, Claude A. : Abrams, Elaine J. : Myer, Landon	2023	Why do Integrated Maternal HIV and Infant Healthcare Services work? A Secondary Analysis of a Randomised Controlled Trial in South Africa	AIDS & Behavior
Brown, Monique J. : Amoatika, Daniel : Kaur, Amandeep : Addo, Prince Nii Ossah : Yoo-Jeong, Moka	2023	Psychosocial Protective and Risk Factors of Quality of Life Outcomes Among Older Adults Living With HIV	AIDS & Behavior
Bryant, J. V. : Carcioppolo, N. : Lun, D. : Potter, J.	2023	Entertainment-education to reduce internalized stigma, increase intimate partner status disclosure intentions, and increase antiretroviral medical adherence intentions: A randomized controlled trial targeting black women living with HIV in the Southern U.	Soc. Sci. Med.
Bu, L. : Chen, X. : Zheng, S. : Fan, G.	2023	Construction of the structural equation model of stigma, self-disclosure, social support, and quality of life of breast cancer patients after surgery-a multicenter study	Frontiers in Oncology
Buonsenso, Danilo : Camporesi, Anna : Morello, Rosa : De Rose, Cristina : Fracasso, Matteo : Chieffo, Daniela Pia Rosaria : Valentini, Piero	2023	Social Stigma in Children with Long COVID	Children
Buse, D. C. : Cady, R. : Starling, A. J. : Buzby, M. : Spinale, C. : Steinberg, K. : Lenaburg, K. : Kymes, S.	2024	Headache/migraine-related stigma, quality of life, disability, and most bothersome symptom in adults with current versus previous high-frequency headache/migraine and medication overuse: results of the Migraine Report Card survey	BMC Neurology
Butt, M. : Chinchilli, V. M. : Leslie, D. L. : Khesroh, E. : Helm, M. F. : Flamm, A. : Kirby, J. S. : Rigby, A.	2022	Internalized skin bias: validation study to explore the impact of the internalization of social stigma on those with hidradenitis suppurativa	Journal of the European Academy of Dermatology and Venereology : JEADV
Calugi, Simona : Segattini, Barbara : Cattaneo, Gianmatteo : Chimini, Mirko : Dalle Grave, Anna : Dametti, Laura : Molgora, Manuela : Dalle Grave, Riccardo	2023	Weight Bias Internalization and Eating Disorder Psychopathology in Treatment-Seeking Patients with Obesity	Nutrients
	2022	Need for psychological support and ethnicity in people diagnosed with Schizophrenia: The importance of the subjective component in addressing internalized stigma	
ÇElİKkanat, Şirin : ÖZgÜÇ, Safiye : EmİNoĞLu, Ayşe	2023	Analysis of the Anxiety Sensitivity and Self-Management in Chronic Illness: Descriptive Research (Correlation Studies)	Turkiye Klinikleri Journal of Nursing Sciences
Cenit-Garcia, Judit : Buendia-Gilabert, Carolina : Contreras-Molina, Carmen : Puente-Fernandez, Daniel : Fernandez-Castillo, Rafael : Garcia-Caro, Maria Paz	2024	Development and Psychometric Validation of the Breast Cancer Stigma Assessment Scale for Women with Breast Cancer and Its Survivors	Healthcare (Basel, Switzerland)
Chaballout, B. H. : Chang, E. M. : Shaverdian, N. : Lee, P. P. : Beron, P. J. : Steinberg, M. L. : Raldow, A. C.	2023	The Patient's perspective on radiation therapy for anal cancer: Evaluation of expectations and stigma	Cancer Reports
Chan, K. K. S. : Tsui, J. K. C.	2023	Longitudinal impact of experienced discrimination on mental health among people with mental disorders	Psychiatry Res.
Chansky, K. : Rigney, M. : King, J. C.	2024	Real-world analysis of the relationships between smoking, lung cancer stigma, and emotional functioning	Cancer Medicine
Chen, Wei-Ti : Huang, Feifei : Shiu, Cheng-Shi : Lin, Sai Htun : Tun, Min San : Nwe, Thet Wai : Oo, Yin Thet Nu : Oo, Htun Nyunt	2024	Can social support mediate stigma and perceived stress in people live with human immunodeficiency virus?	AIDS Care
Chen, W. T. : Huang, F. : Sun, W. : Zhang, L.	2023	Fear of COVID and Physical Health Among People Living with HIV in China: Mediation Effects of HIV Stigma, Social Support, and Substance Use	AIDS and behavior
Chen, X. : Dai, Z. : Fu, J. : Si, M. : Jing, S. : Wu, Y. : Wang, H. : Huang, Y. : Cui, D. : Qu, Y. : Su, X.	2023	Suicidal ideation and associated risk factors among COVID-19 patients who recovered from the first wave of the pandemic in Wuhan, China	QJM: An International Journal of Medicine
Chen, X. : Fan, Y.	2024	Analysis of Factors Influencing the Stigma Associated with Postoperative Cervical Cancer Patients and the Impact of Integrated Nursing Interventions on Stigma	Alternative therapies in health and medicine
Chernyshov, P. V. : Sampogna, F. : Raimondi, G. : Zouboulis, C. C. : Boffa, M. J. : Marron, S. E. : Manolache, L. : Pustišek, N. : Bettoli, V. : Koumaki, D. : Bewley, A. P. : Dreno, B. : Tomas-Aragones, L.	2024	Development of the acne-specific quality of life questionnaire Quality of Life Relevance-Acne	JAAD International
Chiang, S. N. : Skolnick, G. B. : Naidoo, S. D. : Smyth, M. D. : Patel, K. B.	2023	Outcomes after Endoscope-Assisted Strip Craniectomy and Orthotic Therapy for Syndromic Craniosynostosis	Plastic and reconstructive surgery
Choi, H. : Wetmore, J. B. : Camarillo, I. A. : Misiewicz, S. : Siegel, K. : Chung, W. K. : Leu, C. S. : Phelan, J. C. : Yang, L. H. : Ottman, R.	2023	Association of antiseizure medication adherence with illness perceptions in adults with epilepsy	Epilepsy and Behavior
Chou, Pei-Chien : Lee, Yu : Chang, Yung-Yee : Hung, Chi-Fa : Chen, Ying-Fa : Lin, Tsu-Kung : Shih, Fu-Yuan : Chen, Wu-Fu : Lin, Pao-Yen : Chong, Mian-Yoon : Wang, Liang-Jen	2024	The Interrelationship of Benefit Finding, Demoralization, and Stigma among Patients with Parkinson's Disease and Their Caregivers	Healthcare (2227-9032)
Chu, R. S. T. : Ng, C. M. : Chu, S. C. : Lui, T. T. : Lau, F. C. : Chan, S. K. W. : Lee, E. H. M. : Hui, C. L. M. : Chen, E. Y. H. : Lui, S. S. Y. : Chang, W. C.	2023	Rate and correlates of self-stigma in adult patients with early psychosis	Frontiers in Psychiatry
Chunfeng, Cai : Liping, Yu	2017	Quality of Life in Patients With Schizophrenia in China	Journal of Psychosocial Nursing & Mental Health Services
Clemmesen, Maria Elise Ross : Gren, Susanne Thiesen : Frostrup, Anne Grete : Thomsen, Simon Francis : Egeberg, Alexander : Thein, David	2024	Psychosocial and mental impact of alopecia areata: Analysis of the Danish Skin Cohort	Journal of the European Academy of Dermatology and Venereology : JEADV
Crerand, C. E. : Conrad, A. L. : Bellucci, C. C. : Albert, M. : Heppner, C. E. : Sheikh, F. : Woodard, S. : Udaipuria, S. : Kapp-Simon, K. A.	2023	Psychosocial Outcomes in Children with Cleft Lip and/or Palate: Associations of Demographic, Cleft Morphologic, and Treatment-Related Variables	Cleft Palate Craniofacial Journal
Crompvoets, P. I. : Nieboer, A. P. : van Rossum, E. F. C. : Cramm, J. M.	2024	Perceived weight stigma in healthcare settings among adults living with obesity: A cross-sectional investigation of the relationship with patient characteristics and person-centred care	Health Expect.
Cuauro, S. E. : Santos, N. : Andrade, E. : Dani, A. W. : Sanchious, S. N. : Hooper, S. C. : Becker, C. B.	2023	Internalized Weight Stigma and Weight Discrimination: Associations with Quality of Life and Psychosocial Impairment in a Sample Living with Food Insecurity	Int. J. Environ. Res. Public Health
Dai, J. : Sun, D. : Li, B. : Zhang, Y. : Wen, M. : Wang, H. : Bi, H.	2024	Mixed-Mode Mindfulness-based cognitive therapy for psychological resilience, Self Esteem and Stigma of patients with schizophrenia: a randomized controlled trial	BMC Psychiatry
Dar, S. A. : Wani, Z. A. : Baba, N. F. : Nabi, J. : Khanam, A.	2020	A cross-sectional study to assess disability and internalized stigma among treatment-seeking individuals with opioid use disorders	Indian J. Soc. Psychiatry

Davis, C. H. : Twohig, M. P. : Levin, M. E.	2024	Examining processes of change for acceptance and commitment therapy and cognitive behavioral therapy self-help books with depressed college students	Cognitive behaviour therapy
Davis, S. : Gupta, N. : Samudra, M. : Dhamija, S. : Chaudhury, S. : Saldanha, D.	2023	Evaluation of stigma among patients reporting to a tertiary care psychiatric center	Med. J. Dr, D.Y. Patil. Vidyapeeth.
Davtyan, M. : Kacanek, D. : Lee, J. : Berman, C. : Chadwick, E. G. : Smith, R. : Salomon, L. : Frederick, T.	2023	The role of internalised HIV stigma in disclosure of maternal HIV serostatus to children perinatally HIV-exposed but uninfected: a prospective study in the United States	Journal of the International AIDS Society
Davtyan, Mariam : Kacanek, Deborah : Lee, Jessica : Berman, Claire : Chadwick, Ellen G. : Smith, Renee : Salomon, Liz : Frederick, Toinette	2024	Factors associated with internalized HIV-related stigma among biological mothers living with HIV enrolled in a US cohort study	AIDS Care
Davtyan, Mariam : Nguyen, Annie L. : Taylor, Jeff : Christensen, Chris : Brown, Brandon J.	2023	HIV-related Stigma, Personal Mastery, Mindfulness, and Social Support in Older Adults Living with HIV in Coachella Valley, California	Ageing International
Davtyan, M. : Uraga, S. : Wilson, M. L. : Frederick, T.	2023	Internalized HIV-related stigma in women of color obtaining care at an HIV specialty center in Los Angeles County, California	AIDS Care - Psychological and Socio-Medical Aspects of AIDS/HIV
Dawe, J. : Cassano, D. : Keane, R. : Ruth, S. : Wilkinson, A. L. : Elsum, I. : Gunn, J. : Brown, G. : West, M. : Hoy, J. : Power, J. : Stoové, M.	2023	Quality of life among people living with HIV aged 50 years and over in Australia: Identifying opportunities to support better ageing	HIV Medicine
de Rooy, E. C. : Toner, B. B. : Maunder, R. G. : Greenberg, G. R. : Baron, D. : Steinhart, A. H. : McLeod, R. : Cohen, Z.	2001	Concerns of patients with inflammatory bowel disease: results from a clinical population	The American journal of gastroenterology
Değirmenci, T.	2022	Hemifacial spasm and psychiatric comorbidities	Pamukkale. Med. J.
Della Morte, S. : Berti, E. : Lalli, C. : Modugno, N. : Morgante, F. : Schrag, A. : Makovac, E. : Ricciardi, L.	2024	Compassionate mind training for people with Parkinson's disease: A pilot study and predictors of response	European Journal of Neurology
Dellar, R. : Ali, O. : Kinfe, M. : Mengiste, A. : Davey, G. : Bremner, S. : Semrau, M. : Fekadu, A.	2022	Effect of a Community-Based Holistic Care Package on Physical and Psychosocial Outcomes in People with Lower Limb Disorder Caused by Lymphatic Filariasis, Podoconiosis, and Leprosy in Ethiopia: Results from the EnDPoINT Pilot Cohort Study	Am. J. Trop. Med. Hyg.
Delle Donne, V. : Massaroni, V. : Lombardi, F. : Dusina, A. : Salvo, P. F. : Borghetti, A. : Ciccullo, A. : Visconti, E. : Di Giambenedetto, S.	2024	The association between stigma and wellbeing in an Italian cohort of PLWH: The role of social support and personal factors	International Journal of STD and AIDS
Demirkiran, Bedriye Cansu : Kiyak, Emine	2022	Evaluation of Internalized Stigma and Quality of Life of Patients with Psoriasis	Makara Journal of Health Research
Demiryurek, E. : Demiryurek, B. E.	2023	Factors influencing the level of stigma in Parkinson's disease in western Turkey	Ideggyogyaszati Szemle
Deng, Zi-ru : Bernot, Ausma : Davies, Sara E.	2023	Perceived stigma of COVID-19 patients in Shanghai, China, in the third year of the pandemic: a cross-sectional social impact survey	BMC Public Health
Dilsiz, N. B. : Arslan, S.	2023	Investigation of the relationship between weight self-stigma, emotional eating, and diet satisfaction in obese individuals	European Research Journal
Ding, J. L. : Lv, N. : Wu, Y. F. : Chen, I. H. : Yan, W. J.	2024	The hidden curves of risk: a nonlinear model of cumulative risk and school bullying victimization among adolescents with autism spectrum disorder	Child Adolesc. Psychiatry Ment. Health
Drumright, L. N. : Johnson, M. O. : Mayer, K. H. : Christopoulos, K. : Cachay, E. : Crawford, T. N. : Whitney, B. M. : Dai, M. : Ruderman, S. A. : Mixson, L. S. : Keruly, J. C. : Chander, G. : Saag, M. S. : Kitahata, M. M. : Moore, R. D. : Willig, A. L. : Eron, J. J. : Napravnik, S. : Nance, R. M. : Hahn, A. : Ma, J. : Bamford, L. : Fredericksen, R. J. : Delaney, J. A. C. : Crane, H. M.	2024	Differences in internalized HIV stigma across subpopulations of people with HIV in care across the United States	AIDS
Dussault, Josee M. : Akiba, Christopher : Zimba, Chifundo : Malava, Jullita : Akello, Harriet : Stockton, Melissa : Mbota, MacDonald : Matewere, Maureen : Masiye, Jones : Udedi, Michael : Gaynes, Bradley N. : Go, Vivian F. : Hosseinipour, Mina C. : Pence, Brian W.	2023	Evaluating the validity of depression-related stigma measurement among diabetes and hypertension patients receiving depression care in Malawi: A mixed-methods analysis	PLOS global public health
Dussault, J. M. : Zimba, C. : Akello, H. : Stockton, M. : Hill, S. : Aiello, A. E. : Keil, A. : Gaynes, B. N. : Udedi, M. : Pence, B. W.	2023	Estimating the effect of anticipated depression treatment-related stigma on depression remission among people with noncommunicable diseases and depressive symptoms in Malawi	PLoS ONE
Edet, B. E. : Essien, E. A. : Olose, E. O. : Okafor, C. J. : Ogbodum, M. U. : Daniel, F. M.	2024	Pattern and causes of missed appointments in a Nigerian Psychiatric Hospital A cross-sectional study	Medicine (United States)
Eitel, K. B. : Roberts, A. J. : D'agostino, R. : Barrett, C. E. : Bell, R. A. : Bellatorre, A. : Cristello, A. : Dabelea, D. : Dolan, L. M. : Jensen, E. T. : Liese, A. D. : Mayer-Davis, E. J. : Reynolds, K. : Marcovina, S. M. : Pihoker, C.	2023	Diabetes Stigma and Clinical Outcomes in Adolescents and Young Adults: The SEARCH for Diabetes in Youth Study	Diabetes Care
Ekmen, A. : Doulazmi, M. : Méneret, A. : Jegatheesan, P. : Hervé, A. : Damier, P. : Gras, D. : Roubertie, A. : Piard, J. : Mutez, E. : Tarrano, C. : Welniarz, Q. : Vidailhet, M. : Worbe, Y. : Gallea, C. : Roze, E.	2023	Non-Motor Symptoms and Quality of Life in Patients with PRRT2-Related Paroxysmal Kinesigenic Dyskinesia	Mov. Disord. Clin. Pract.
Eliaçık, Sinan : Aykaç, Serdar : Karakaşlı, Alp : Tan, Funda Uysal : Özalp, Elvan	2023	Hitit University Epilepsy Outpatient Clinic Experience	Archives of Epilepsy
Elkalla, I. H. R. : El-Gilany, A. H. : Baklola, M. : Terra, M. : Aboeldahab, M. : Sayed, S. E. : ElWasify, M.	2023	Assessing self-stigma levels and associated factors among substance use disorder patients at two selected psychiatric hospitals in Egypt: a cross-sectional study	BMC Psychiatry
Ellin, Mohammed Rasheedan : Ramoo, Vimala : Yahaya, Nor Aziyan : Tan Maw, Pin	2023	Association between Affiliate Stigma and Psychological Well-being among Caregivers of Patients with Dementia: A Quantitative Report	Malaysian Journal of Nursing (MJN)
English, S. : Vallis, M.	2023	Moving beyond eat less, move more using willpower: Reframing obesity as a chronic disease impact of the 2020 Canadian obesity guidelines reframed narrative on perceptions of self and the patient-provider relationship	Clinical Obesity
Er, D. : AktaŞ, B.	2023	An investigation of stigma and self-management in individuals diagnosed with epilepsy	Epilepsy and Behavior
Erdoğan Kaya, A. : Aydinoğlu, Ü	2023	A correlational and cross-sectional study on the relationship between internalized stigma and religious coping in patients with schizophrenia	Medicine (United States)
Erdogan Kaya, Ayse : Erdogan Akturk, Beyza	2023	The Relationship Between Religious Coping and Internalized Stigma Among Patients With Bipolar Disorder	Cureus
Evangeli, M. : Foster, C. : Musiime, V. : Fidler, S. : Seeley, J. : Frize, G. : Uwizera, A. : Price, J.	2024	Cultural Adaption, Translation, Preliminary Reliability and Validity of Key Psychological and Behavioural Measures for 18 to 25 Year-Olds Living with HIV in Uganda: A Multi-Stage Approach	AIDS Behav.
Eweida, R. S. : Abdelwahab Khedr, M. : Hussein, R. M.	2024	A comparative study of old versus novel psychoactive substances on craving, perceived stigma and suicidal risk among rural-dwelling patients with substance abuse	Journal of psychiatric and mental health nursing
Fakih, A. : Tannous, R. : Lajnef, M. : Seneschal, J. : Andreu, N. : Tran, V. T. : Ezzedine, K.	2024	Stigma in vitiligo: associated factors and severity strata of the Patient Unique Stigmatization Holistic tool in Dermatology (PUSH-D) score	British Journal of Dermatology
Faller, Y. N. : Peynenburg, V. : Tessier, E. : Thiessen, D. : Hadjistavropoulos, H. D.	2023	Efficacy of an Online Workplace Mental Health Accommodations Psychoeducational Course: A Randomized Controlled Trial	Int. J. Environ. Res. Public Health
Fan, W. : Ma, K. K. : Yang, C. X. : Guo, Y. L.	2023	The mediating effect of stigma between self-perceived burden and loneliness in stroke patients	Frontiers in Psychiatry
Fan, Y. : Yu, B. : Liu, H. : Ma, H. : Ma, C. : Li, Y. : Feng, C. : Jia, P. : Yang, S.	2024	Network analysis of illness perception, stigma, and resilience with cognition in old people living with HIV	Journal of Psychosomatic Research
Fang, X. : Ren, K. : Li, Y. : Meng, Q. : Li, M. : Miao, M. : Zhan, J. : Wang, X. : Wu, F. : Zhang, M.	2024	Prevalence and influencing factors of social alienation among elderly patients undergoing radical prostatectomy for prostate cancer	Current Urology
Faraade, M. H. : Jeffree, M. S. : Avoi, R. : Fiidow, O. A.	2023	Tuberculosis Stigma Intervention, Hargeisa Hospital Somalia - A Randomized, Controlled Trial	Malays. J. Med. Health Sci.
Fekete, L. : Iantovics, L. B. : Fekete, G. L.	2024	Validation of the DLQI questionnaire in assessing the disease burden and principal aspects related to life quality of vitiligo patients	Front. Psychol.
Fekih-Romdhane, F. : Hajje, R. : Haddad, C. : Hallit, S. : Azar, J.	2023	Exploring negative symptoms heterogeneity in patients diagnosed with schizophrenia and schizoaffective disorder using cluster analysis	BMC Psychiatry
Fhager, Johan : Svensson, Ake : Ormon, Karin : Fischer, Tobias W. : Sjostrom, Karin	2023	The Hairdex quality of life instrument-A translation and psychometric validation in patients with alopecia areata	Skin health and disease
Filiatreau, L. M. : Ebasone, P. V. : Dzudie, A. : Wainberg, M. : Yotebieng, M. : Anastos, K. : Parcesepe, A. M.	2024	Intersectional HIV- and Depression-Related Stigma Among People with HIV Entering HIV Care in Cameroon	AIDS and behavior
Filiz, İ : Didem, B. : Aslan, M. : Yilmaz, E. : KamiLoğlu, O.	2024	The effect of childhood trauma on sleep quality and functionality in patients with bipolar disorder-I	Journal of Experimental and Clinical Medicine (Turkey)
Fitrianur, Widya Lita : Widiyawati, Wiwik : Suminar, Ervi	2022	SELF-STIGMA AS THE TRIGGER OF DEPRESSION FACTOR IN MULTIDRUG - RESISTANT TUBERCULOSIS (MDR-TB) PATIENTS AT A PUBLIC HOSPITAL IN GRESIK	Indonesian Nursing Journal of Education & Clinic (INJEC)
Forney, Derrick J. : Sheehan, Diana M. : Dale, Sannisha K. : Li, Tan : De La Rosa, Mario : Spencer, Emma C. : Sanchez, Mariana	2024	The Impact of HIV-Related Stigma on Racial/Ethnic Disparities in Retention in HIV Care Among Adults Living with HIV in Florida	Journal of Racial & Ethnic Health Disparities

Fotouhi, A. R. : Patel, K. B. : Skolnick, G. B. : Merrill, C. M. : Hofmann, K. M. : Mantilla-Rivas, E. : Collett, B. R. : Allhusen, V. D. : Naidoo, S. D. : Rogers, G. F. : Keating, R. F. : Smyth, M. D. : Magge, S. N.	2023	School-age anthropometric and patient-reported outcomes after open or endoscopic repair of sagittal craniosynostosis	J. Neursurg. Pediatr.
Fu, Po Chun : Hsu, Chia Ling : Huang, Min Hsi : Yang, Chiu Yueh	2024	Effect of an Anti-Stigma Program on Self-Stigma for Chinese Individuals With Schizophrenia: A Pilot Study With a Quasi-Experimental Design	Journal of Psychosocial Nursing & Mental Health Services
Fuady, A. : Arifin, B. : Yunita, F. : Rauf, S. : Fitriangga, A. : Sugiharto, A. : Yani, F. F. : Nasution, H. S. : Putra, I. G. A. E. : Mansyur, M. : Wingfield, T.	2023	Stigma towards people with tuberculosis: a cross-cultural adaptation and validation of a scale in Indonesia	BMC psychology
Fuady, Ahmad : Arifin, Bustanul : Yunita, Ferdiana : Rauf, Saidah : Fitriangga, Agus : Sugiharto, Agus : Yani, Finny Fitry : Nasution, Helmi Suryani : Putra, I. Wayan Gede Artawan Eka : Mansyur, Muchtaruddin : Wingfield, Tom	2024	Stigma, depression, quality of life, and the need for psychosocial support among people with tuberculosis in Indonesia: A multi-site cross-sectional study	PLOS global public health
Gamassa, Editruda : Steven, Ester : Mtei, Rachel : Kaaya, Sylvia	2023	Prevalence of Depression and Suicidal Ideation and Associated Risk Factors in Adolescents Receiving Care and Treatment for Hiv/Aids at a Tertiary Health Facility in Kilimanjaro Region, Tanzania	Research square
Gamwell, K. L. : Roberts, C. M. : Kraft, J. D. : Edwards, C. S. : Baudino, M. N. : Grunow, J. E. : Jacobs, N. J. : Tung, J. : Mullins, L. L. : Chaney, J. M.	2023	Factor analysis of the stigma scale-child in pediatric inflammatory bowel disease	Journal of Psychosomatic Research
Garg, R. : Kaur, H.	2020	Quality of life among patients with depression: Impact of self-stigma	Indian J. Soc. Psychiatry
Garg, R. : Singla, A. : Raj, R.	2023	Health-related quality of life and stigma in opioid dependence: Comparison between buprenorphine users and non-users	Journal of Neurosciences in Rural Practice
Gastón-Panthaki, A. : Serrano, A. : Virani, N. : Sylvestre, J. : Crisafulli, B. F. : Becker, C. B.	2023	Food insecurity, weight-based discrimination, weight self-stigma, and mental health in post-bariatric surgery patients	Body image
Gerdes, S. : Ostendorf, R. : Süß, A. : Schadeck, T. : Taut, F. : Makuc, J. : Scharfenberger, L. : Jacobsen, S. : Trenkler, N. : Behrens, J. : Joks, G. : Tabori, S. : Mortazawi, D.	2024	Effectiveness, safety and impact of guselkumab on sexuality and perceived stigmatization in patients with psoriasis in routine clinical practice: Week 28 results from the prospective German multicentre G-EPOSS study	Journal of the European Academy of Dermatology and Venereology
Geta, Edosa Tesfaye : Guteta, Temesgen Oluma : Tiruneh, Gemechu	2024	Impairment of health-related quality of life and its determinants among patients with podoconiosis in East Wollega Zone, Oromia Regional State, Ethiopia: institutional-based cross-sectional study	BMJ open
Ghajarzadeh, M. : Fitzgerald, K. C. : Mowry, E. M. : Nourbakhsh, B.	2024	Association between demographics, socioeconomics, and disease-related factors and the perception of stigma in people with MS	Multiple Sclerosis Journal
Ghearing, Gena R. : Tyrrell, Maegan : Black, Jessica : Krehel-Montgomery, Jacqueline : Yala, Joy : Adeniyi, Clara : Briggs, Farren : Sajatovic, Martha	2024	Clinical correlates of negative health events and disparities among adults with epilepsy enrolled in a self-management clinical trial	Epilepsy research
Gjesfjeld, Christopher D. : Kahn, Jeffrey H.	2024	Self-disclosure of mental illness in the college classroom: the role of stigma and avoidance	Social Work in Mental Health
Gnanasekaran, Vijayalakshmi : Perumal, Vanamail : Periyasamy, Mahendiran : Ramanathan, Meena : de Britto, Lourduraj	2023	Stigma and stigma-induced stress in filarial lymphoedema patients in Puducherry, India	Journal of Health Sciences
Gökşin, Ş : İmren, I. G.	2024	Internalized stigma, disease severity, quality of life, anxiety and depression in axillary hyperhidrosis	Annales de Dermatologie et de Venereologie
Goyal, A. K. : Bakshi, J. : Panda, N. K. : Kapoor, R. : Vir, D. : Kumar, K. : Aneja, P.	2023	Accuracy of a self-reported Measure in Psychological Assessment when the Instrument is self-administered by the Patient or when Administrated by the Clinician	Indian J. Otolaryngol. Head Neck Surg.
Goyal, A. K. : Bakshi, J. : Panda, N. K. : Kapoor, R. : Vir, D. : Kumar, K. : Aneja, P.	2023	Shame and Stigma Over Long-Term Survival in Postoperative Cases of Head and Neck Cancer	J. Maxillofac. Oral Surg.
Graboyes, E. M. : Kistner-Griffin, E. : Hill, E. G. : Maurer, S. : Balliet, W. : Williams, A. M. : Padgett, L. : Yan, F. : Rush, A. : Johnson, B. : McLeod, T. : Dahne, J. : Ruggiero, K. J. : Sterba, K. R.	2023	Efficacy of a brief cognitive behavioral therapy for head and neck cancer survivors with body image distress: secondary outcomes from the BRIGHT pilot randomized clinical trial	Journal of cancer survivorship : research and practice
Grady, K. L. : Kallen, M. A. : Cella, D. : Allen, L. A. : Lindenfeld, J. : McIlvennan, C. K. : Beiser, D. G. : Walsh, M. N. : Denfeld, Q. E. : Lee, C. S. : Ruo, B. : Murks, C. : Stehlik, J. : Kirklin, J. K. : Teuteberg, J. : Adler, E. : Kiernan, M. : Rich, J. : Bedjeti, K. : Hahn, E. A.	2024	Efficient measurement of multiple ventricular assist device patient-reported outcomes: Creation of a 20-item profile from the MCS A-QOL study	Journal of Heart and Lung Transplantation
Graff, N. R. : Hong, C. : Guthrie, B. : Micheni, M. : Chirro, O. : Wahome, E. : van der Elst, E. : Sanders, E. J. : Simoni, J. M. : Graham, S. M.	2023	The Effect of the Shikamana Peer-and-Provider Intervention on Depressive Symptoms, Alcohol Use, and Other Drug Use Among Gay, Bisexual, and Other Men Who Have Sex with Men in Kenya	AIDS and behavior
Gruszczyńska, E. : Rzeszutek, M.	2024	Daily stigma and daily emotional well-being among people living with HIV: Testing a buffering hypothesis of social support during the COVID-19 pandemic	Applied psychology. Health and well-being
Gu, Zhi Hui : Wang, Jia Yi : Yang, Chen Xin : Wu, Hui	2024	Study on the Profiles of Sleep Disorders, Associated Factors, and Pathways Among Gynecological Cancer Patients - A Latent Profile Analysis	Nature and science of sleep
Gudeta, D. B. : Leta, K. : Alemu, B. : Kandula, U. R.	2023	Medication adherence and associated factors among psychiatry patients at Asella Referral and Teaching Hospital in Oromia, Ethiopia: Institution based cross sectional study	PLoS ONE
Gudzune, K. A. : Kaplan, L. M. : Kahan, S. : Kumar, R. B. : Dunn, J. P. : Ahmad, N. N. : Poon, J. L. : Sims, T. J. : Mackie, D. S. : Jauregui, A. K. : Balkaran, B. L. : Kan, H. : Ard, J.	2024	Weight-Reduction Preferences Among OBSERVE Study Participants With Obesity or Overweight: Opportunities for Shared Decision-Making	Endocrine Practice
Gull, M. : Javaid, Z. K. : Khan, K. : Chaudhry, H. A.	2023	Improving healthcare for substance users: the moderating role of psychological flexibility on stigma, mental health, and quality of life	International Journal of Human Rights in Healthcare
Gupta, N. : Samudra, M. : Dhamija, S. : Chaudhury, S. : Saldanha, D.	2023	Perceived stigma among caregivers of psychiatric disorders as compared to chronic dermatological disorders	Med. J. Dr, D.Y. Patil. Vidyapeeth.
Gutin, S. A. : Ruark, A. : Darbes, L. A. : Neilands, T. B. : Mkandawire, J. : Conroy, A. A.	2023	Supportive couple relationships buffer against the harms of HIV stigma on HIV treatment adherence	BMC Public Health
Ha, T. : Shi, H. : Gaikwad, S. S. : Joshi, K. : Padiyar, R. : Schensul, S. L.	2024	Longitudinal trajectories of depressive symptoms among alcohol consuming men with HIV in India	Journal of Affective Disorders
Hamano, S. : Onishi, Y. : Yoshida, Y. : Takao, T. : Tahara, T. : Kikuchi, T. : Kobori, T. : Kubota, T. : Iwamoto, M. : Kasuga, M.	2023	Association of self-stigma with glycated hemoglobin: A single-center, cross-sectional study of adults with type 1 diabetes in Japan	Journal of Diabetes Investigation
Hamidi, Samira : Ebrahimi, Hossein : Vahidi, Maryam : Areshtanab, Hossein Namdar	2023	Internalized Stigma and its Association with Hope, Self-Esteem, Self-Efficacy, and Treatment Adherence among Outpatients with Severe Mental Illness: A Cross-Sectional Survey	Iranian journal of nursing and midwifery research
Han, Zi-Yin : Chen, Yong : Chen, You-Di : Sun, Guo-Min : Dai, Xiao-Ying : Yin, Yue-Qin : Geng, Ya-Qin	2023	Latent characteristics and influencing factors of stigma in rheumatoid arthritis: A latent class analysis	Medicine
Hanson, Olivia R. : Weglarz, Anya J. : Barabara, Mariam L. : Cohen, Susanna R. : Minja, Linda M. : Mlay, Pendo S. : Stephens, Maya J. : Olomi, Gaudensia A. : Mlay, Janeth : Mmbaga, Blandina T. : Watt, Melissa H.	2024	HIV-related Shame among Women Giving Birth in Tanzania: A Mixed Methods Study	AIDS & Behavior
Harrison, Abigail : Mtukushe, Bulelwa : Kuo, Caroline : Wilson-Barthes, Marta : Davidson, Bianca : Sher, Rebecca : Galarraga, Omar : Hoare, Jacqueline	2023	Better Together: acceptability, feasibility and preliminary impact of chronic illness peer support groups for South African adolescents and young adults	Journal of the International AIDS Society
He, D. : He, L. : Yuan, Y. : Huang, L. : Xiao, Q. : Ye, X. : Zhang, J. E.	2023	Stigma and its correlates among patients with Crohn's disease: A cross-sectional study in China	International Journal of Nursing Sciences
He, H. : Huang, X. : Yu, B. : Liu, Y. : Mai, S. : Ouyang, L. : Zhang, Q. : Yan, X.	2024	Stigma in Elderly Females with Stress Urinary Incontinence: A Latent Profile Analysis	Clinical and Experimental Obstetrics and Gynecology
Hill, S. E. : Zhang, C. : Remera, E. : Ingabire, C. : Umwiza, F. : Munyaneza, A. : Muhoza, B. : Rwibasira, G. : Yotebieng, M. : Anastos, K. : et al.,	2024	Association Between Clinical Encounter Frequency and HIV-Related Stigma Among Newly-Diagnosed People Living with HIV in Rwanda	AIDS and behavior
Hoang, V. T. H. : Pham, H. T. : Nguyen, L. T. P. : Tran, N. A. : Le-Thi, V. Q. T.	2024	The relationship between HIV-related stigma and quality of life among HIV infected outpatients: A cross-sectional study in Vietnam	J. Public Health Res.
Hofmann, M. : Jermann, F. : Baggio, S. : Küng, A. L. : Favre, S. : Dayer, A. : Aubry, J. M. : Richard-Lepouriel, H.	2023	Childhood trauma and self-stigma in mood disorders	Psychiatry Research Communications
Hohmann, L. : Bien, C. G. : Holtkamp, M. : Grewe, P.	2024	German questionnaires assessing quality of life and psycho-social status in people with epilepsy: Reliable change and intercorrelations	Epilepsy and Behavior
Hong, Cheng-lin : Ochoa, Ayako Miyashita : Wilson, Bianca D. M. : Wu, Elizabeth S. C. : Thomas, Damone : Holloway, Ian W.	2023	The associations between HIV stigma and mental health symptoms, life satisfaction, and quality of life among black sexual minority men with HIV	Quality of Life Research
Horsakulchai, W. : Sermprasartkul, T. : Sumetchoengprachya, P. : Chummaneekul, P. : Rungruang, N. : Uthis, P. : Sripan, P. : Srithanaviboonchi, K.	2024	Factors associated with internalized HIV-related stigma among people living with HIV in Thailand	AIDS Care - Psychological and Socio-Medical Aspects of AIDS/HIV
Hosseini, S. : Ranjbaran, F. : Shahmoradi, Z. : Omidi, F. : Rezaie, M. : Mohamadi, Z. : Tajerian, A.	2024	The Role of Internalized Stigma in Modulating Hope, Self-Esteem, and Self-Efficacy among Outpatients with Mental Illness	Iranian Journal of Psychiatry and Behavioral Sciences
Howard, L. : Flach, C. : Leese, M. : Byford, S. : Killaspy, H. : Cole, L. : Lawlor, C. : Betts, J. : Sharac, J. : Cutting, P. : McNicholas, S. : Johnson, S.	2010	Effectiveness and cost-effectiveness of admissions to women's crisis houses compared with traditional psychiatric wards: pilot patient-preference randomised controlled trial	The British journal of psychiatry. Supplement
Hsiao, Chiu-Yueh : Hsieh, Ming-Hong : Chung, Feng-Chin : Chiu, Shu-Chuan : Chang, Chi-Wen : Tsai, Yun-Fang	2023	Changes in family functioning among primary family caregivers of patients with schizophrenia	Journal of nursing scholarship : an official publication of Sigma Theta Tau International Honor Society of Nursing

Hu, C. : Zhao, Y. : Xiao, Z.	2024	Effects of stigma on the quality of life in patients with epilepsy	Acta Epileptologica
Huang, Haitao : Zhang, Liao : Dong, Wanglin : Tu, Ling : Tang, Haishan : Liu, Shejuan : Chen, Hong : Xie, Naze : Chen, Chaoran	2024	Stigma and loneliness among young and middle-aged stroke survivors: A moderated mediation model of interpersonal sensitivity and resilience	Journal of Psychiatric & Mental Health Nursing (John Wiley & Sons, Inc.)
Huang, Li-Ting : Liu, Chieh-Yu : Yang, Chiu-Yueh	2023	Narrative enhancement and cognitive therapy for perceived stigma of chronic schizophrenia: A multicenter randomized controlled trial study	Archives of Psychiatric Nursing
Huang, L. T. : Liu, Y. L. : Pao, C. H. : Chang, Y. H. : Chu, R. Y. : Hsu, H. M. : Wei, D. R. : Yang, C. Y.	2024	The association of social support and hope with self-stigma and perceived recovery among people with schizophrenia: The serial mediation effect	Journal of advanced nursing
Hyesun, Kim : Kawoun, Seo	2022	The Mediating Effect of Acceptance Action in the Relationship between Diabetes Distress and Self-stigma among Old Adults with Diabetes in South Korea	Journal of Korean Academy of Community Health Nursing / Jiyeog Sahoe Ganho Hakoeji
İlter, Z. Ç : Çetin, S. B. : Özkorumak, E. : Tiryaki, A. : Ak, İ	2023	The relationship between internalized stigma and coping strategies in bipolar disorder	Klin. Psikiyatr. Derg.
İzci, F. : Fidan, Y. : Çallı, S. : Aslan, M.	2024	Stigma, Perception of Social Support, and Functionality Levels of Schizophrenia Patients with and without Legal Guardianship	Psychiatry and Clinical Psychopharmacology
Jankowiak, B. : Krajewska-Kułak, E. : Jakoniuk, M. : Khvorik, D. F.	2023	Stigmatization among Patients with Plaque Psoriasis	J. Clin. Med.
Janocko, N. J. : Morton, M. L. : Groover, O. : Teagarden, D. L. : Villarreal, H. K. : Merchant, S. : Ahuruonye, N. : Rodriguez-Ruiz, A. A. : Drane, D. L. : Karakis, I.	2022	Translating Phenomenology of Psychogenic Nonepileptic Seizures Into Nosology: Insights From Patients' and Caregivers' Profiles	Neurologist
Janssens, K. M. E. : Joosen, M. C. W. : Henderson, C. : Bakker, M. : den Hollander, W. : van Weeghel, J. : Brouwers, E. P. M.	2024	Effectiveness of a Stigma Awareness Intervention on Reemployment of People with Mental Health Issues/Mental Illness: A Cluster Randomised Controlled Trial	Journal of Occupational Rehabilitation
Jegede, O. : Muvvala, S. : Katehis, E. : Paul, S. : Soipe, A. : Jolayemi, A.	2021	Perceived barriers to access care, anticipated discrimination and structural vulnerability among African Americans with substance use disorders	The International journal of social psychiatry
Ji, Q. : Zhang, L. : Xu, J. : Ji, P. : Song, M. : Chen, Y. : Guo, L.	2024	The relationship between stigma and quality of life in hospitalized middle-aged and elderly patients with chronic diseases: the mediating role of depression and the moderating role of psychological resilience	Frontiers in Psychiatry
Jiang, N. : Jin, W. : Fu, Z. : Cao, H. : Zheng, H. : Wang, Q. : Ju, K. : Wang, J. : Zhang, Q.	2024	Effects of Social Support on Medication Adherence Among Patients with Schizophrenia: Serial Multiple Mediation Model	Patient Preference and Adherence
Jiménez-Rivagorza, L. : Orozco, R. : Medina-Mora, M. E. : Rafful, C.	2024	HIV-Related Stigma and Treatment Adherence Among Gay, Bisexual, and Other Men Who Have Sex with Men Who Use Crystal Meth in the Metropolitan Area of Mexico City	Arch. Sex. Behav.
Kaggwa, M. M. : Najjuka, S. M. : Mamun, M. A. : Griffiths, M. D. : Nyemara, N. : Ashaba, S.	2023	Involvement and burden of informal caregivers of patients with mental illness: the mediating role of affiliated stigma	BMC Psychiatry
Kane, J. C. : Mauro, P. M. : Hahn, J. A. : Chander, G. : Tobin, K. E. : Martins, S. S. : Paniagua-Avila, A. : Latkin, C. A.	2023	HIV stigma and disclosure of alcohol use to physicians: examining the mediating role of depression among persons living with HIV	AIDS Care - Psychological and Socio-Medical Aspects of AIDS/HIV
Karram, S. : Sanger, C. : Convery, C. : Brantley, A.	2024	Social Determinants of Health Among Persons Living with HIV Impact Important Health Outcomes in Michigan	AIDS & Behavior
Katcher, J. G. : Klassen, A. C. : Hann, H. W. : Chang, M. : Juon, H. S.	2024	Racial discrimination, knowledge, and health outcomes: The mediating role of hepatitis B-related stigma among patients with chronic hepatitis B	Journal of Viral Hepatitis
Kato, A. : Yoshiuchi, K. : Hashimoto, H. : Suzuki, R. : Yamauchi, T. : Kadowaki, T.	2023	Feasibility, acceptability, and effects of a self-stigma reduction pilot program for Japanese individuals with type 2 diabetes	PEC. Innov.
Katusiime, B. : Cassidy, R. : Krska, J. : Corlett, S. A.	2024	Medicine burden experiences of people living with HIV and association with stigma	AIDS Care - Psychological and Socio-Medical Aspects of AIDS/HIV
Kaur, K. : Sharma, G. : Dwivedi, R. : Nehra, A. : Parajuli, N. : Upadhyay, A. D. : Deepak, K. K. : Jat, M. S. : Ramanujam, B. : Sagar, R. : Mohanty, S. : Tripathi, M.	2023	Effectiveness of Yoga Intervention in Reducing Felt Stigma in Adults with Epilepsy: A Randomized Controlled Trial	Neurology
Kaur, Ramandeep : Garg, Rohit : Raj, Rajnish	2023	Quality of life among patients with obsessive compulsive disorder: Impact of stigma, severity of illness, insight, and beliefs	Industrial psychiatry journal
Kawoun, Seo	2024	Validity and Reliability of the Korean Version of the Diabetes Acceptance and Action Scale (DAAS-K)	Research in Community & Public Health Nursing (RCPHN)
Kayaoğlu, K. : Ay, E.	2024	Examination of internalized stigma, quality of life, and happiness in patients with schizophrenia	Journal of Mental Health
Kazemi, F. : Omidi, F. : Shahmoradi, Z. : Ranjbaran, F. : Tajerian, A.	2024	Internalized Stigma and Adherence to Treatment Among Outpatients with Mental Illness	Iranian Journal of Psychiatry and Behavioral Sciences
Kechine, Temesgen : Ali, Tilahun : Worku, Teshager : Abdisa, Lemesa : Assebe Yadeta, Tesfaye	2022	Anxiety and Associated Factors Among Clients on Highly Active Antiretroviral Therapy (HAART) in Public Hospitals of Southern Ethiopia: A Multi-Center Cross-Sectional Study	Psychology research and behavior management
Kendie, Mikiyas Tesera : Worku, Lelisa : Abebaw, Ermias : Solomon, Damtew : Luke, Amana Ogeto : Hayilu, Sisay : Bogale, Eyob Ketema	2023	Predictors of suicidal ideation, attempts among adults living with HIV attending Art follow-ups at tirunesh Beijing general hospital, Addis Ababa, Ethiopia: a cross-sectional study	BMJ Open
Kerr, A. M. : Lin, S. : Sisk, B. A.	2023	Mental and physical health of adult patients affected by complex vascular anomalies	Patient Education and Counseling
Kerrigan, D. : Barrington, C. : Donastorg, Y. : Perez, M. : Gomez, H. : Davis, W. : Beckham, S. W. : Karver, T. S. : Mantsios, A. : Galai, N.	2024	Individual and Collective Forms of Stigma Resistance: Pathways Between HIV and Sex Work Stigma and Viral Suppression Among Female Sex Workers in the Dominican Republic	AIDS Behav.
Khalaf, O. O. : Fathy, H. : Ebrahim, H. A. M. : Samie, M. A.	2023	Self-stigma and coping in youth with schizophrenia and bipolar disorder: a comparative study	Middle East Curr. Psychiatry
Kilapilo, M. S. : Mosha, I. H. : Bwire, G. M. : Sambayi, G. L. : Sangeda, R. Z. : Killewo, J.	2023	Factors Associated with Viral Load Suppression and Indicators of Stigma among People Living with HIV in Dar es Salaam Tertiary Hospitals, Tanzania	Microbiology Research
KiliÇLi, Ayşegül : Akbulut, Şahide : Damar ÇAkirca, Tuba	2022	Stigma, Hopelessness, Depression and Associated Factors in People Living with HIV	Journal of Harran University Medical Faculty / Harran Üniversitesi Tıp Fakültesi Dergisi
Kim, J. H. : Kim, J. M. : Ye, M. : Lee, J. I. : Na, S. : Lee, Y. : Short, D. : Choi, J. Y.	2022	Implementation of a Nurse-Delivered Cognitive Behavioral Therapy for Adherence and Depression of People Living with HIV in Korea	Infection and Chemotherapy
Kim, K. : Jang, S. : Rim, H. D. : Kim, S. W. : Chang, H. H. : Woo, J.	2023	Attachment Insecurity and Stigma as Predictors of Depression and Anxiety in People Living With HIV	Psychiatry Investig.
Kim, Naru : Kang, Danbee : Shin, Sang Hyun : Heo, Jin Seok : Shim, Sungkeun : Lim, Jihyun : Cho, Juhee : Han, In Woong	2023	Effects of cancer stigma on quality of life of patients with hepatobiliary and pancreatic cancer	Annals of hepato-biliary-pancreatic surgery
Kimmel, S. D. : Samet, J. H. : Cheng, D. M. : Vetrova, M. : Idrisov, B. : Rossi, S. L. : Rateau, L. : Astone, K. : Michals, A. : Sisson, E. : Blokhina, E. : Milet-Carty, N. : Bovell-Ammon, B. J. : Gnatienko, N. : Truong, V. : Krupitsky, E. : Lunze, K.	2024	Stigma and other correlates of sharing injection equipment among people with HIV in St. Petersburg, Russia	Global Public Health
Kiratli Nalbant, Esra : Imren, Isil Gogem : Tas Dolek, Gamze	2024	Internalized Stigma and Its Relationship With Quality of Life and Perceived Health Status in Rosacea and Acne Vulgaris: A Comparative Cross-Sectional Study	Cureus
Kizilpinar, S. C. : Demir, B. K.	2024	Investigation of self-stigmatization and perceptions towards delinquency in inpatient individuals diagnosed with schizophrenia in high-security forensic psychiatry settings in Turkiye	Klin. Psikiyatr. Derg.
Knettel, B. : Minja, L. : Msoka, E. : Tarimo, C. : Katiti, V. : Pan, W. : Mwobobia, J. : Juhlin, E. : Knippler, E. : Watt, M. : Suneja, G. : Kimani, S. : Abouelella, D. : Mmbaga, B. : Osazuwa-Peters, N.	2024	Culturally-informed adaptation and psychometric properties of the Cataldo Cancer Stigma Scale in Northern Tanzania	Journal of Psychosocial Oncology
Kolek, A. : Prasko, J. : Ociskova, M. : Holubova, M. : Vanek, J. : Grambal, A. : Slepecky, M.	2019	Severity of panic disorder, adverse events in childhood, dissociation, self-stigma and comorbid personality disorders Part 2: Therapeutic effectiveness of a combined cognitive behavioural therapy and pharmacotherapy in treatment-resistant inpatients	Neuroendocrinology Letters
Komatsu, H. : Ono, T. : Maita, Y. : Ishida, Y. : Kikuchi, T. : Maki, T. : Hase, S. : Sakurai, H. : Oba, A. : Teshirogi, O. : Suzuki, A. : Mori, Y. : Shoji, C. : Fujita, A. : Takahashi, S. : Ebina, T. : Ozaki, S. : Honma, R. : Tomita, H. : Kakuto, Y.	2020	Association between autistic symptoms and self-stigma in patients with schizophrenia spectrum disorders	Neuropsychiatric Disease and Treatment
Kore, S. : Bhide, A. : Bhide, V. : Shinde, A.	2023	A Cross Sectional Epidemiological Study to Find the Prevalence of Depression and Anxiety in Patients of Leprosy and It's Correlation to Stigma Related with Leprosy	International Journal of Pharmaceutical and Clinical Research
Kota, K. K. : Luo, Q. : Beer, L. : Dasgupta, S. : McCree, D. H.	2023	Stigma, Discrimination, and Mental Health Outcomes Among Transgender Women With Diagnosed HIV Infection in the United States, 2015-2018	Public Health Rep.
Kuramochi, I. : Iwayama, T. : Brandt, C. : Yoshimasu, H. : Bien, C. G. : Hagemann, A.	2023	Assessment of self-stigma in epilepsy: Validation of the German version Epilepsy Self-Stigma Scale (ESSS-G)	Epilepsia Open
Kuramochi, I. : Iwayama, T. : Oga, K. : Shiganami, T. : Umemura, T. : Kobayashi, S. : Yasuda, T. : Yoshimasu, H.	2023	An online survey on differences in knowledge and stigma about epilepsy among the Tokyo metropolitan area and non-urban areas in Japan, a post-hoc study	Epilepsy and Behavior
Lacroix, A. : Puybaret, V. : Villéger, P. : Zattoni-Leroy, J. : Cantaloube, S. : Chevalier, C. : Nubukpo, P.	2024	Predictive factors for acceptance of a long-acting opiate substitution treatment studied through social representations and internalized stigma	Therapies

Lai, J. S. : Nowinski, C. : Rangel, S. M. : Batra, S. T. : Mueller, K. : Chamlin, S. : Ustsinovich, V. : Cella, D. : Mansolf, M. : Paller, A. S.	2024	Development of the PROMIS pediatric stigma and extension to the PROMIS pediatric stigma: skin item banks	Qual. Life Res.
Lalatović, S. : Smiljanić, I. : Ristić, A. J. : Cvorović, D. : Golubović, V. : Parojčić, A. : Baščarević, V. : Krstić, N. : Milovanović, M.	2023	Psychometric evaluation of the Serbian version of the Stigma Scale of Epilepsy (SSE)	Epilepsy Behav.
Lasalvia, A. : Pillan, S. : Marzocco, G. : Ambrosini, A. : Veltro, F. : Pozzan, T. : D'Astore, C. : Cristofalo, D. : Ruggeri, M. : Bonetto, C.	2023	Development and validation of a new standardized measure for assessing experiences of discrimination within mental health services. A participatory research project	Epidemiol. Psychiatr. Sci.
Lavefjord, A. : Sundström, F. T. A. : Buhrman, M. : McCracken, L. M.	2024	The role of stigma in health and functioning in chronic pain: Not just catastrophizing	Eur. J. Pain
Laxmi, R. : Sahoo, S. : Grover, S. : Nehra, R.	2023	Psychological/Personal recovery and its correlates in patients with first episode psychosis	The International journal of social psychiatry
Li, B. : Lin, X. : Chen, S. : Qian, Z. : Wu, H. : Liao, G. : Chen, H. : Kang, Z. : Peng, J. : Liang, G.	2024	The association between fear of progression and medical coping strategies among people living with HIV: a cross-sectional study	BMC public health
Li, J. M. : Su, X. Q. : Xu, X. P. : Xue, P. : Guo, Y. J.	2023	Influencing factors analysis of adaptability of cancer patients to return-to-work	Supportive Care Cancer
Li, Qian : Yang, Xue : Wang, Xin : Zhang, Han : Ding, Ningning : Zhao, Wenqian : Tian, Wenwen : He, Jiankang : Du, Mingxuan : Hu, Haiyan : Zhang, Guohua	2023	COVID-19 symptoms, internet information seeking, and stigma influence post-lockdown health anxiety	Frontiers in psychology
Li, S. : Jiang, Y. : Yuan, B. : Wang, M. : Zeng, Y. : Knobf, M. T. : Wu, J. : Ye, Z.	2024	The interplay between stigma and sleep quality in breast cancer: A cross-sectional network analysis	European journal of oncology nursing : the official journal of European Oncology Nursing Society
Li, Shuhan : Wang, Xinqin : Wang, Minyi : Jiang, Yingting : Mai, Qingxin : Wu, Jiahua : Ye, Zengjie	2023	Association between stigma and sleep quality in patients with breast cancer: A latent profile and mediation analysis	European journal of oncology nursing : the official journal of European Oncology Nursing Society
Li, X. H. : Deng, S. Y. : Zhang, T. M. : Wang, Y. Z. : Wong, I. Y. L. : Ran, M. S.	2024	Medication non-adherence and its influencing factors in persons with schizophrenia in rural China	J. Ment. Health
Li, X. : Wu, L. : Yun, J. : Sun, Q.	2023	The status of stigma in patients with type 2 diabetes mellitus and its association with medication adherence and quality of life in China: A cross-sectional study	Medicine (United States)
Li, Y. : Qiu, D. : Wu, Q. : Ni, A. : Tang, Z. : Xiao, S.	2023	Family caregivers' abusive behaviour and its association with internalized stigma of people living with schizophrenia in China	Schizophr.
Li, Y. : Zhang, J. : Hu, J. : Chen, X. : Yang, X. : Zhu, Y. : Fan, Y. : Zhang, X. : Xu, X.	2024	Stigma and related influencing factors in brain cancer patients: a cross-sectional study and parallel mediation analysis	Supportive Care in Cancer
Lifson, A. R. : Hailemichael, A. : Workneh, S. : MacLehose, R. F. : Horvath, K. J. : Hilk, R. : Sites, A. : Shenie, T.	2023	Impact of Community Support Workers in Rural Ethiopia on Emotional and Psychosocial Health of Persons Living with HIV: Results of a Three-Year Randomized Community Trial	AIDS and behavior
Lin, C. W. : Chang, Y. P. : Yen, C. F.	2023	Predictors of Motivation to Receive a COVID-19 Vaccination and the Number of COVID-19 Vaccine Doses Received in Patients with Schizophrenia	Vaccines
Lin, P. Y. : Chou, W. J. : Hsiao, R. C. : Liu, T. L. : Yen, C. F.	2023	Association of Affiliate Stigma with Parenting Stress and Its Moderators among Caregivers of Children with Attention-Deficit/Hyperactivity Disorder	International Journal of Environmental Research and Public Health
Lin, Z. : Cheng, L. : Han, X. : Wang, H. : Liao, Y. : Guo, L. : Shi, J. : Fan, B. : Teopiz, K. M. : Jawad, M. Y. : Zhang, H. : Chen, Y. : Lu, C. : McIntyre, R. S.	2023	The Effect of Internet-Based Cognitive Behavioral Therapy on Major Depressive Disorder: Randomized Controlled Trial	Journal of Medical Internet Research
Ling, Mao-Sheng : Wang, Chao-Ping : Hsieh, Yu-Ling : Lin, Yi-Ping : Lee, Pi-Chung : Hu, Sophia : Hung, Fang-Ming	2023	Emotional disturbance and risk factors among COVID-19 confirmed cases in isolation hotels	International Journal of Mental Health Nursing
Liu, F. : Deng, H. : Hu, N. : Huang, W. : Wang, H. : Liu, L. : Chai, J. : Li, Y.	2024	The relationship between self-stigma and quality of life in long-term hospitalized patients with schizophrenia: a cross-sectional study	Frontiers in Psychiatry
Liu, Heng-Yu : Liu, Qun-Hong : Li, Zhuo-Ran : Deng, Cheng-Song : Zhang, Xiao-Pei : Wan, Li-Hong	2024	The cognitive appraisal path of stroke knowledge, coping traits, family functioning and stigma among stroke patients: A moderated parallel mediation model	Journal of clinical nursing
Liu, Qun-Hong : Tan, Ju-Xiang : Hu, Cai-Xia : Zhang, Xiao-Pei : Liu, Shu-Ying : Wan, Li-Hong	2023	Relationship of family function and pre-hospital delay among Chinese patients with recurrent ischaemic stroke and the mediation effect of stigma	European Journal of Cardiovascular Nursing
Liu, Xiang-min : Lan, Hui-zhen : Bai, Xin-yu : Li, Qi-an : Wen, Yan : Feng, Mei : Tang, Xiang-dong	2023	Sleep quality and its associated factors among patients with tuberculosis: a cross-sectional study	Frontiers in Public Health
Liu, Z. J. : Feng, L. S. : Li, F. : Yang, L. R. : Wang, W. Q. : He, Y. : Meng, Z. T. : Wang, Y. F.	2023	Development and validation of the thyroid cancer self-perceived discrimination scale to identify patients at high risk for psychological problems	Frontiers in Oncology
Lo Hog Tian, J. M. : Watson, J. R. : McFarland, A. : Parsons, J. A. : Maunder, R. G. : McGee, A. : Boni, A. R. : Cioppa, L. : Ajiboye, M. E. : Rourke, S. B.	2023	The cost of anticipating stigma: a longitudinal examination of HIV stigma and health	AIDS Care - Psychological and Socio-Medical Aspects of AIDS/HIV
Lodi, Sara : Rossi, Sarah L. : Bendiks, Sally : Gnatienko, Natalia : Lloyd-Travaglini, Christine : Vetrova, Marina : Toussova, Olga : Bushara, Natalia : Blokhina, Elena : Krupitsky, Evgeny : Ekstrand, Maria L. : Lioznov, Dmitry : Samet, Jeffrey H. : Lunze, Karsten	2023	Correlates of Intersectional HIV and Substance Use Stigma Affecting People with HIV and Substance Use in St. Petersburg, Russia	AIDS & Behavior
Lu, Weili : Caldwell, Barbara : Gao, Ni : Oursler, Janice : Wang, Ke : Beninato, John : Srijeyanthan, Jeganee : Kumi, Cindy : Sawyer, Jeremy : Giacobbe, Giovanna : Chen, Yubi : Lin, Karen Wei-Ru : Mueser, Kim T.	2024	Healing Trauma While Staying at Home: Using Telehealth to Conduct a Brief Treatment Program for Posttraumatic Stress Disorder	Journal of Psychosocial Nursing & Mental Health Services
Lv, M. : Feng-Fang, Y. : Wang, Y. : Zhen-Xu, H.	2023	Factors contributing to emotional distress when caring for children with imperforate anus: a multisite cross-sectional study in China	Frontiers in Medicine
Ma, M. : Ju, P. : Xia, Q. : Pan, Z. : Gao, J. : Zhang, L. : Gao, H. : Yan, J. : Zhang, J. : Wang, K. : Li, C. : Xie, W. : Zhu, C.	2023	Automatic Thoughts, Self-Stigma, and Resilience Among Schizophrenia Patients with Metabolic Syndrome: A Cross-Sectional Study	Neuropsychiatric Disease and Treatment
Maas, I. L. : Bohlken, M. M. : Gangadin, S. S. : Rosema, B. S. : Veling, W. : Boonstra, N. : de Haan, L. : Begemann, M. J. H. : Koops, S.	2024	Personal recovery in first-episode psychosis: Beyond clinical and functional recovery	Schizophrenia Research
Mahfoud, D. : Fekih-Romdhane, F. : Abou Zeid, J. : Rustom, L. : Mouez, C. : Haddad, G. : Hallit, S.	2023	Functionality appreciation is inversely associated with positive psychotic symptoms in overweight/obese patients with schizophrenia	BMC Psychiatry
Malama, K. : Logie, C. H. : Sokolovic, N. : Skeritt, L. : O'Brien, N. : Cardinal, C. : Gagnier, B. : Loutfy, M. : Kaida, A. : De Pokomandy, A.	2023	Pathways From HIV-Related Stigma, Racial Discrimination, and Gender Discrimination to HIV Treatment Outcomes Among Women Living With HIV in Canada: Longitudinal Cohort Findings	Journal of Acquired Immune Deficiency Syndromes
Malika, Nipher : Bogart, Laura M. : Mutchler, Matt G. : Goggin, Kathy : Klein, David J. : Lawrence, Sean J. : Wagner, Glenn J.	2024	Loneliness Among Black/African American Adults Living with HIV: Sociodemographic and Psychosocial Correlates and Implications for Adherence	Journal of Racial & Ethnic Health Disparities
Mangla, P. M. : Barot, C. K.	2023	Stigma and Depression in Patients Suffering from HIV/AIDS	International Journal of Pharmaceutical and Clinical Research
Marsidi, N. : Ottevanger, R. : Demir, Y. E. : van Beugen, S. : Goeman, J. J. : Genders, R. E.	2024	Patient-reported outcome measurements in facial skin surgery and a comparison between Mohs micrographic surgery and conventional excisions	Journal of the European Academy of Dermatology and Venereology
Martiningsih, W. : Mulyadi, A. : Winarni, S. : Retnaningtyas, E.	2024	Development of Self Stigma Assessment Tool for People Living With HIV/AIDS (PLWHA)	Natl. J. Community. Med.
Masa, Rainier : Zimba, Mathias : Zimba, Gilbert : Zulu, Graham : Zulu, Joseph : Operario, Don	2024	The Association of Emotional Support, HIV Stigma, and Home Environment With Disclosure Efficacy and Perceived Disclosure Outcomes in Young People Living With HIV in Zambia: A Cross-Sectional Study	JANAC: Journal of the Association of Nurses in AIDS Care
Massaroni, V. : Iannone, V. : Donne, V. D. : D'Angelillo, A. : Baldin, G. : Passerotto, R. : Sangiorgi, F. : Steiner, R. J. : Ciccullo, A. : Borghetti, A. : Visconti, E. : Giambenedetto, S. D.	2024	HIV and vicarious stigma in a cohort of people living with HIV in Italy: What happens when the stigma is fueled by healthcare providers?	AIDS Care - Psychological and Socio-Medical Aspects of AIDS/HIV
McCree, Donna Hubbard : Beer, Linda : Crim, Stacy M. : Kota, Krishna Kiran : Baugher, Amy : Jeffries Iv, William L. : Patel, Deesha : Marcus, Ruthanne : Yuan, Xin Anne : Luke Shouse, R.	2023	Intersectional Discrimination in HIV Healthcare Settings Among Persons with Diagnosed HIV in the United States, Medical Monitoring Project, 2018-2019	AIDS & Behavior
McKinney-Prupis, E. : Chiu, Y. C. J. : Grov, C. : Tsui, E. K. : Duke, S. I.	2023	Psychosocial and Health-Related Behavioral Outcomes of a Work Readiness HIV Peer Worker Training Program	International Journal of Environmental Research and Public Health
McLaren, T. : Peter, L. J. : Tomczyk, S. : Muehlan, H. : Schomerus, G. : Schmidt, S.	2023	The Seeking Mental Health Care model: prediction of help-seeking for depressive symptoms by stigma and mental illness representations	BMC Public Health
Mehel Tutuk, S. P. : Budak, F.	2023	The effect of mindfulness-based psychoeducation on internalized stigma and substance abuse proclivity in individuals with substance use disorder	Journal of Substance Use
Mehra, A. : Kumar, A. : Grover, S. : Chakrabarti, S. : Avasthi, A.	2020	Relationship of stigma with burden and coping among caregivers of patients with severe mental disorders	Indian J. Soc. Psychiatry
Mehta, H. : Narang, T. : Singh, S.	2024	Task sharing for the management of leprosy by nurses in a tertiary healthcare setting of Northern India	Trans. R. Soc. Trop. Med. Hyg.
Mei, Yu-jin : Yang, Xue : Gui, Jiao-feng : Li, Yu-qing : Zhang, Xiao-yun : Wang, Ying : Chen, Wen-yue : Chen, Ming-jia : Liu, Chang-jun : Zhang, Lin	2023	The relationship between psychological resilience and quality of life among the Chinese diabetes patients: the mediating role of stigma and the moderating role of empowerment	BMC Public Health

Mei, Y. : Yang, X. : Gui, J. : Li, Y. : Zhang, X. : Wang, Y. : Chen, W. : Chen, M. : Liu, C. : Zhang, L.	2023	The relationship between psychological resilience and depression among the diabetes patients under the background of "dynamic zero COVID-19": the mediating role of stigma and the moderating role of medication burden	Frontiers in public health
Mei, Y. : Yang, X. : Liu, C. : Li, Y. : Gui, J. : Zhang, L.	2023	The impact of psychological resilience on chronic patients' depression during the dynamic Zero-COVID policy: the mediating role of stigma and the moderating role of sleep quality	BMC psychology
Mejri, I. : Ouali, U. : Gronholm, P. C. : Zgueb, Y. : Ouertani, A. : Nacef, F.	2023	"To fast or not to fast?" Ramadan and religiosity through the eyes of people with bipolar disorder: an exploratory study	Frontiers in Psychiatry
Meng, D. : Jin, Z. : Gao, L. : Wang, Y. : Wang, R. : Fang, J. : Qi, L. : Su, Y. : Liu, A. : Fang, B.	2022	The quality of life in patients with Parkinson's disease: Focus on gender difference	Brain Behav.
Mesías-Gazmuri, J. : Folch, C. : Palacio-Vieira, J. : Bruguera, A. : Egea-Cortés, L. : Forero, C. G. : Hernández, J. : Miró, J. M. : Navarro, J. : Riera, M. : Peraire, J. : Alonso-García, L. : Díaz, Y. : Casabona, J. : Reyes-Urueña, J.	2023	Syndemic conditions and quality of life in the PISCIS Cohort of people living with HIV in Catalonia and the Balearic Islands: a cross sectional study	Health and Quality of Life Outcomes
Mesinkovska, N. : Craiglow, B. : Ball, S. G. : Morrow, P. : Smith, S. G. : Pierce, E. : Shapiro, J.	2023	The Invisible Impact of a Visible Disease: Psychosocial Impact of Alopecia Areata	Dermatology and Therapy
Meyers, A. : Jin, A. : Kwiecien, G. J. : Gatherwright, J. : Khetarpal, S. : Zins, J. E.	2023	Platelet-Rich Plasma for Treatment of Hair Loss Improves Patient-Reported Quality of Life	Aesthetic plastic surgery
Mgbako, Ofole : Loughran, Claire : Mathu, Rachel : Castor, Delivette : McLean, Jacob : Sobieszczyk, Magdalena E. : Olender, Susan : Gordon, Peter : Lopez-Rios, Javier : Remien, Robert H.	2023	Rapid or Immediate ART, HIV Stigma, Medical Mistrust, and Retention in Care: An Exploratory Mixed Methods Pilot Study	AIDS & Behavior
Minneci, P. C. : Gil, L. A. : Cooper, J. N. : Asti, L. : Nishimura, L. : Lutz, C. M. : Deans, K. J.	2024	Laser Epilation as an Adjunct to Standard Care in Reducing Pilonidal Disease Recurrence in Adolescents and Young Adults: A Randomized Clinical Trial	JAMA Surgery
Mlunde, L. B. : Hirschhorn, L. R. : Nyblade, L. : Rothrock, N. E. : Mbugi, E. V. : Moskowitz, J. T. : Kaaya, S. : Hawkins, C. : Leyna, G. : Mbwambo, J. K.	2023	Translation and cultural adaptation of drug use stigma and HIV stigma measures among people who use drugs in Tanzania	PLoS ONE
Modi, L. : Gedam, S. R. : Shivji, I. A. : Babar, V. : Patil, P. S.	2018	Comparison of total self-stigma between schizophrenia and alcohol dependence patients	Int. J. High Risk Behav. Addict.
Mohammedhussein, M. : Dule, A. : Tessema, W. : Mamaru, A. : Alenko, A.	2023	Perceived stress and its psychosocial and clinical correlates among patients with pulmonary tuberculosis: A cross-sectional study	Indian J. Psychiatry
Mohamud, A. K. : Ahmed, O. A. : Mohamud, A. A. : Dirie, N. I.	2023	Prevalence of and factors associated with depression among adult patients living with HIV/AIDs undergoing ART unit in Banadir hospital, Mogadishu Somalia	BMC Psychiatry
Moraleda, Á : Galán-Casado, D. : Cangas, A. J.	2019	Reducing self-stigma in people with severe mental illness participating in a regular football league: An exploratory study	International Journal of Environmental Research and Public Health
Moring, J. C. : Peterson, A. L. : Straud, C. L. : Ortman, J. : Mintz, J. : Young-McCaughan, S. : McGeary, C. A. : McGeary, D. D. : Litz, B. T. : Macdonald, A. : Roache, J. D. : Resick, P. A. : For The Strong Star, Consortium	2023	The interactions between patient preferences, expectancies, and stigma contribute to posttraumatic stress disorder treatment outcomes	Journal of traumatic stress
Mugo, C. : Kohler, P. : Kumar, M. : Badia, J. : Kibugi, J. : Wamalwa, D. C. : Kapogiannis, B. : Agot, K. : John-Stewart, G. C.	2023	Effect of HIV stigma on depressive symptoms, treatment adherence, and viral suppression among youth with HIV	AIDS
Munie, B. M. : Guangul, M. M. : Mamaru, A. : Asnakew, S. : Amha, H. : Tedla, A.	2024	Quality of life among caregivers of patients with severe mental illness in northwest Ethiopia, 2022: an institutional-based cross-sectional study	Frontiers in Psychiatry
Murshidi, R. : Hammouri, M. : Al-Ani, A. : Kitaneh, R. : Al-Soleiti, M. : Al Ta'ani, Z. : Sweis, S. : Halasa, Z. : Fashho, E. : Arafah, M. : Almaani, N. : Abdallat, M. : Al-Dar'awi, F. : Kittaneh, E. : Jaber, B. : Almudallal, F. : Smadi, Z.	2024	Investigating the prevalence of body dysmorphic disorder among Jordanian adults with dermatologic and cosmetic concerns: a case-control study	Scientific reports
Murugan, Yogesh : Patel, Nirmalkumar : Kumar, Vinay : Gandhi, Rohankumar	2024	Mental Health Impacts of Multidrug-Resistant Tuberculosis in Patients and Household Contacts: A Mixed Methods Study	Cureus
Mutamba, B. B. : Rukundo, G. Z. : Sembajjwe, W. : Nakasujja, N. : Birabwa-Oketcho, H. : Mpango, R. S. : Kinyanda, E.	2024	A 'hidden problem': Nature, prevalence and factors associated with sexual dysfunction in persons living with HIV/AIDS in Uganda	PLoS ONE
Nabunya, P. : Ssewamala, F. M. : Kizito, S. : Mugisha, J. : Brathwaite, R. : Neilands, T. B. : Migadde, H. : Namuwonge, F. : Ssentumbwe, V. : Najjuuko, C. : et al.,	2024	Preliminary Impact of Group-Based Interventions on Stigma, Mental Health and Treatment Adherence Among Adolescents Living with HIV in Uganda	Journal of pediatrics
Naftaly, J. P. : Feldman, E. C. H. : Greenley, R. N.	2024	Perceived Stigma in Patients with Autoimmune Hepatitis	Journal of Clinical Psychology in Medical Settings
Narita, Z. : Hazumi, M. : Kataoka, M. : Usuda, K. : Nishi, D.	2024	Association between discrimination and subsequent psychotic experiences in patients with COVID-19: A cohort study	Schizophrenia Research
Nelson, A. K. : Denavit, C. : Muñoz, M. : Wong, M. : Saldaña, O. : Santa Cruz, J. : Rodriguez, C. A. : Caldas, A. : Castro, A. : Shin, S.	2023	The Dynamics of Intimate Partner Violence and Its Impact on HIV Care: A Cross-Sectional Study of People of Mixed Gender and Sexual Preference in Lima, Peru	Journal of interpersonal violence
Nguyen, H. B. : Vo, L. N. Q. : Forse, R. J. : Wiemers, A. M. C. : Huynh, H. B. : Dong, T. T. T. : Phan, Y. T. H. : Creswell, J. : Dang, T. M. H. : Nguyen, L. H. : Shedrawy, J. : Lönnroth, K. : Nguyen, T. D. : Dinh, L. V. : Annerstedt, K. S. : Codlin, A. J.	2024	Is convenience really king? Comparative evaluation of catastrophic costs due to tuberculosis in the public and private healthcare sectors of Viet Nam: a longitudinal patient cost study	Infectious Diseases of Poverty
Nguyen, H. L. T. : Bui, T. M. : Dam, V. A. T. : Nguyen, T. T. : Nguyen, H. T. : Zeng, G. M. : Bradley, D. : Nguyen, Q. N. : Van Ngo, T. : Latkin, C. A. : Ho, R. C. M. : Ho, C. S. H.	2023	Avoidance of healthcare service use and correlates among HIV-positive patients in Vietnam: a cross-sectional study	BMJ Open
Nogueira, Andreza Soares : Garcia, Monique Allana Chagas : Silva, Moises Batista da : Costa, Patricia Fagundes da : Frade, Marco Andrey Cipriani : Salgado, Claudio Guedes : Barreto, Josafa Goncalves	2024	Clofazimine-induced cutaneous hyperpigmentation as a source of stigma in the treatment of leprosy: A cross-sectional study	Tropical medicine & international health : TM & IH
Norcini Pala, A. : Turan, B.	2024	A Bayesian network analysis to examine the effects of HIV stigma processes on self-concept and depressive symptoms among persons living with HIV	Journal of personality
Nurye, Y. : Tareke, M. : Tadesse, M. : Shegaw, M. : Mekonen, T.	2023	Depression among people with chronic skin disease at Boru Meda Hospital in Northeast Ethiopia	PLoS ONE
Ochoa-Morales, A. : Fresan-Orellana, A. : Ramírez-García, M. Á : Márquez-González, H. : Martínez-Juárez, I. E. : López-Uribe, M. : Zuniga-García, C. : Jara-Prado, A. : Luis Guerrero-Camacho, J. : Dávila-Ortiz de Montellano, D. J.	2023	Low quality of life, increased number of anti-seizure drugs, and the lack of caregiver support are associated with internalized stigma in adult Mexican patients with epilepsy	Epilepsy and Behavior
Østberg, N. : Jacobsen, B. G. : Lauridsen, M. M. : Ladegaard Grønkjær, L.	2023	Mental Health, Quality of Life, and Stigmatization in Danish Patients with Liver Disease	International Journal of Environmental Research and Public Health
Ownsworth, Tamara : Mols, Helen : O'Loghlen, Jessica : Xie, Yanfei : Kendall, Melissa : Nielsen, Mandy : Mitchell, Jessie : Jones, Rachel : Geraghty, Timothy	2024	Stigma following acquired brain injury and spinal cord injury: relationship to psychological distress and community integration in the first-year post-discharge	Disability & Rehabilitation
Pal, A.	2020	A cross-sectional study of internalized stigma in euthymic patients of bipolar disorder across its predominant polarity	Indian J. Soc. Psychiatry
Paller, A. S. : Rangel, S. M. : Chamlin, S. L. : Hajek, A. : Phan, S. : Hogeling, M. : Castelo-Soccio, L. : Lara-Corrales, I. : Arkin, L. : Lawley, L. P. : Funk, T. : Castro Porto Silva Lopes, F. : Antaya, R. J. : Ramien, M. L. : Vivar, K. L. : Teng, J. : Coughlin, C. C. : Rehmus, W. : Gupta, D. : Bercovitch, L. : Stein, S. L. : Boull, C. : Tom, W. L. : Liang, M. G. : Hunt, R. : Luu, M. : Holland, K. E. : Schoch, J. J. : Cella, D. : Lai, J. S. : Griffith, J. W. : Afifi, L. : Bahl, A. : Bice, D. : Bruckner, A. : Canter, T. : Cheng, C. : Diaz, L. : Dupuy, E. : Drolet, B. : Fadzeyeva, H. : Fernandez, E. : Glick, S. : Harfmann, K. : Hawryluk, E. B. : Hughes, M. : Humphrey, S. : Huynh, T. : Jones, M. : Kamili, N. : Koh, E. : Kruse, L. : Lam, J. : Lancaster, J. : Lee, M. : Levy, M. L. : Lor, M. : Martin, K. : Martinez-Cabriales, S. : Nguyen, A. : O'Haver, J. : Olamiju, B. : Park, H. : Patel, V. : Rangu, S. : Razi, R. : Rosenblatt, A. : Flores, X. S. : Siegel, D. : Silverberg, N. : Tollefson, M. : Tran, J.	2024	Stigmatization and Mental Health Impact of Chronic Pediatric Skin Disorders	JAMA Dermatology
Pan, Deyu : Qin, Sang : Brown, Wilson J. : Sánchez, Jennifer	2024	Psychometric Evaluation of the Symptom-Checklist-K-9 Among U.S. Working-Age Adults With Psychiatric Disabilities	Rehabilitation Psychology
Pan, Shucheng : Wang, Lijuan : Zheng, Li : Luo, Jie : Mao, Jinjiao : Qiao, Wenbo : Zhu, Binbin : Wang, Wei	2023	Effects of stigma, anxiety and depression, and uncertainty in illness on quality of life in patients with prostate cancer: a cross-sectional analysis	BMC psychology
Parekh, U. : Bardolia, D. D. : Gandhi, R. : Nemlekar, S.	2024	Internalized Stigma and Quality of Life in Patients With Alcohol Use Disorder: A Cross-Sectional Study From Gujarat, India	Current Psychiatry Research and Reviews
Park, D. : Park, S.	2024	Role of Stigma in Moderating the Effects of Loneliness on Mental Health Problems Among Patients With COVID-19 in South Korea	Psychiatry Investigation
Pascual-Sanchez, A. : Jenaro, C. : Montes, J. M.	2020	Understanding social withdrawal in euthymic bipolar patients: The role of stigma	Psychiatry Research

Patel, R. S. : Makwana, D. : Shukla, R. : Pandey, V.	2023	Comparative Study of Health-Related Quality of Life, Psychological Well-Being, Impact of Illness, and Stigma in Epilepsy and Migraine: A Comprehensive Analysis	Journal of Cardiovascular Disease Research
Patterson, Sophie : Nicholson, Valerie : Gormley, Rebecca : Carter, Allison : Logie, Carmen H. : Closson, Kalysha : Ding, Erin : Trigg, Jason : Li, Jenny : Hogg, Robert : de Pokomandy, Alexandra : Loutfy, Mona : Kaida, Angela	2022	Impact of Canadian human immunodeficiency virus non-disclosure case law on experiences of violence from sexual partners among women living with human immunodeficiency virus in Canada: Implications for sexual rights	Women's Health (17455057)
Peng, Hsi-Ling : Chen, Yen-Hui : Lee, Han-Yen : Tsai, Wen-Ying : Chang, Ya-Lan : Lai, Yeur-Hur : Chen, Shu-Ching	2024	Factors associated with shame and stigma among head and neck cancer patients: a cross-sectional study	Supportive care in cancer : official journal of the Multinational Association of Supportive Care in Cancer
Peralta, V. : García de Jalón, E. : Moreno-Izco, L. : Peralta, D. : Janda, L. : Sánchez-Torres, A. M. : Cuesta, M. J.	2024	The association of adverse childhood experiences with long-term outcomes of psychosis: a 21-year prospective cohort study after a first episode of psychosis	Psychological medicine
Piro, L. : Luo, H. : Jones, K. : Lazorick, S. : Cummings, D. M. : Saeed, S. A.	2023	Racial and Ethnic Differences Among Active-Duty Service Members in Use of Mental Health Care and Perceived Mental Health Stigma: Results From the 2018 Health Related Behaviors Survey	Prev. Chronic Dis.
Pitanupong, J. : Aunjitsakul, W.	2024	Personal and perceived stigma in relation to diverse domains of quality of life among patients with major depressive disorder having residual symptoms: a hospital-based cross-sectional study in Thailand	Qual. Life Res.
Pitanupong, J. : Sammathit, J.	2023	Knowledge and attitudes on medication adherence and residual symptoms in individuals with depression: a survey at a University Hospital	BMC Psychiatry
Polonijo, A. N. : Nguyen, A. L. : Greene, K. Y. : Lopez, J. L. : Yoo-Jeong, M. : Ruiz, E. L. : Christensen, C. : Galea, J. T. : Brown, B.	2024	Brief virtual intervention associated with increased social engagement and decreased negative affect among people aging with HIV	AIDS Care - Psychological and Socio-Medical Aspects of AIDS/HIV
	2022	Chilean Validation of the Spanish Version of the Recovery Assessment Scale (RAS) in Patients Diagnosed with Schizophrenia	
Pos, K. : Franke, N. : Smit, F. : Wijnen, B. F. M. : Staring, A. B. P. : Van Der Gaag, M. : Meijer, C. : De Haan, L. : Velthorst, E. : Schirmbeck, F.	2019	Cognitive behavioral therapy for social activation in recent-onset psychosis: Randomized controlled trial	Journal of Consulting and Clinical Psychology
Priasmoro, D. P. : Dradjat, R. S. : Zuhriyah, L. : Lestari, R. : Subagiyono,	2023	A model of acceptance for family caregivers in the management of severe mental disorders	Medical Journal of Malaysia
Price, J. K. : Gerber, L. H. : Stepanova, M. : de Avila, L. : Weinstein, A. A. : Pham, H. : Nader, F. : Afendy, M. : Terra, K. : Austin, P. : Keo, W. : Racila, A. : Estep, J. M. : Gerber, S. : Verma, M. : Golabi, P. : Lam, B. P. : Younossi, Z.	2023	Post-Acute SARS-CoV-2 Symptoms are Fewer, Less Intense Over Time in People Treated with Mono-Clonal Antibodies for Acute Infection	International Journal of General Medicine
Price, Sarah N. : Neil, Jordan M. : Flores, Melissa : Ponzani, Colin : Muzikansky, Alona : Ballini, Lauren : Ostroff, Jamie S. : Park, Elyse R.	2023	Patient-Reported Receipt of Oncology Clinician-Delivered Brief Tobacco Treatment (5As) Six Months following Cancer Diagnosis	Oncology
Prikhodkina, M. : Melnikov, S.	2024	Factors that influence medication adherence in women with fibromyalgia: A path analysis	Journal of clinical nursing
Pulerwitz, J. : Gottert, A. : Tun, W. : Eromhonsele, A. F. : Oladimeji, P. L. : Shoyemi, E. : Akoro, M. : Ndeloa, C. : Adedimeji, A.	2024	Reducing stigma and promoting HIV wellness/mental health of sexual and gender minorities: RCT results from a group-based programme in Nigeria	Journal of the International AIDS Society
Pullano, A. : Melmed, K. R. : Lord, A. : Olivera, A. : Frontera, J. : Brush, B. : Ishida, K. : Torres, J. : Zhang, C. : Dickstein, L. : Kahn, E. : Zhou, T. : Lewis, A.	2024	Negative disease-related stigma 3-months after hemorrhagic stroke is related to functional outcome and female sex	Journal of Stroke and Cerebrovascular Diseases
Quiles-Tsimaratos, N. : Gherardi, A. : Crochard, A. : Hueber, M. : Pain, E. : Vives, A. : Villani, A. P.	2024	The patient's perspective on the burden of psoriasis: findings based on the ROCQ, an online survey	European Journal of Dermatology
Rafferty, M. R. : Achler, S. : Su, H. : Kocherginsky, M. : Bega, D. : Heinemann, A. W. : Johnson, K.	2023	Financial hardship is associated with employment challenges and reduced quality of life in early Parkinson's disease	Clinical Parkinsonism and Related Disorders
Rajendran, Arunima : Gurushanthappa, Yashwant : Akkineni, Shilpa	2022	Life satisfaction in the caregivers of schizophrenia and factors associated with it: a cross sectional study from South India	EUREKA Health Sciences
Ramos-Vera, C. : Basauri-Delgado, M. : Diaz Peña, M. : Tinoco Alberto, J. : Perez Arroyo, K. : Herrera Mamani, B. : Sánchez-Villena, A. : Saintila, J.	2023	Bifactor SEM and MIRT Structure of a 12-Item Human Immunodeficiency Virus Stigma Scale in Peruvian Adults	Journal of primary care & community health
Razali, Salleh Mohd : Hussein, Suria : Ismail, Tg Alina Tg	2010	PSYCHIATRY. Perceived Stigma and Self-Esteem among Patients with Schizophrenia	International Medical Journal
Reinius, M. : Svedhem, V. : Bruchfeld, J. : Larm, H. H. : Nygren-Bonnier, M. : Eriksson, L. E.	2023	COVID-19-related stigma among infected people in Sweden; psychometric properties and levels of stigma in two cohorts as measured by a COVID-19 stigma scale	PLoS ONE
Blanca Reneses; Julia Sevilla-Llewellyn-Jones; Regina Vila-Badia; Tomas Palomo; Cristina Lopez-Micó; Manuel Pereira;Maria José Regatero; Susana Ochoa	2020	Relación entre variables sociodemográficas, psicosociales y clínicas y el estigma personal en pacientes con diagnóstico de esquizofrenia	Actas Esp Psiquiatr
Rimal, S. : Kumari, Y. P.	2023	Stigma Perceived by the Family Members of Psychiatric Patient	International Medical Journal
Rinawati, S. A. W. : Puspitasari, S.	2023	FAMILY SUPPORT AND STIGMATIZATION ON MOTIVATION TO CURE DRUG-RESISTANT TUBERCULOSIS IN WOMEN	Community Pract.
Robinson, K. M. : Scherer, A. M. : Nishimura, T. E. : Laroche, H. H.	2023	Value of cognitive interviewing in the development of the weight stigma in healthcare inventory	Patient Educ. Couns.
Rodríguez-Otero, L.M.	2023	Risky sexual behaviors and stigma in seropositive Mexican patients	Cuadernos de Trabajo Social
Rzeszutek, M. : Gruszczyńska, E.	2023	Depression during the COVID-19 pandemic among people living with HIV: Are low HIV/AIDS stigma and high perceived emotional support protective resources?	Stress and Health
Sabola, N. E. : Hussien, S. : Kamel, A. : Elsalamony, M. A. : Shahin, M. A. : Ali, L. A. : Loulah, S. A.	2023	Implementing of Nursing Intervention on Knowledge, Perceived Stigma and Health related Outcomes among Patients with Hepatitis B Virus	International Journal of Pharmaceutical Quality Assurance
Sadashiv, M. : Kakunje, A. : Karkal, R. : Ganganna, S.	2020	Twin-center study comparing stigma among males and females with alcohol dependence	Soc. Health. Behav.
Saffari, M. : Chang, K. C. : Chen, J. S. : Potenza, M. N. : Yen, C. F. : Chang, C. W. : Huang, P. C. : Tsai, H. C. : Lin, C. Y.	2023	Sleep Quality and Self-Stigma Mediate the Association Between Problematic Use of Social Media and Quality of Life Among People With Schizophrenia in Taiwan: A Longitudinal Study	Psychiatry Investigation
Schlachter, S. : Sommer, R. : Augustin, M. : Tsianakas, A. : Westphal, L.	2023	A Comparative Analysis of the Predictors, Extent and Impacts of Self-stigma in Patients with Psoriasis and Atopic Dermatitis	Acta Dermato-Venereologica
Schmalzle, S. A. : Grant, M. : Lovelace, S. : Jung, J. : Choate, C. : Guerin, J. : Weinstein, W. : Taylor, G.	2024	Survey of pain and stigma experiences in people diagnosed with mpox in Baltimore, Maryland during 2022 global outbreak	PLoS ONE
Schmidt, S. : Fischer, T. W. : Chren, M. M. : Strauss, B. M. : Elsner, P.	2001	Strategies of coping and quality of life in women with alopecia	The British journal of dermatology
Secinti, E. : Snyder, S. : Wu, W. : Mosher, C. E.	2023	Preliminary Validation of the Injustice Experience Questionnaire in Patients With Advanced Cancer	Journal of Pain and Symptom Management
Sedney, C. : Cowher, A. : Turiano, N. A. : Cox, S. : Dekeseredy, P. : Haggerty, T.	2023	Understanding the Intersectional Relationship of Pain Stigma, Weight Bias Internalization, and Clinical Indicators in a Rural Population with Back Pain: A Survey-Based Study	World Neurosurgery
Seekles, M. L. : Kadima, J. K. : Ding, Y. : Bulambo, C. B. : Kim, J. J. : Kukola, J. K. : Omumbu, P. O. L. : Mulamba, R. M. : Nganda, M. : Ngenyibungi, S. M. : Ngondu, F. L. : Sabuni, L. P. : Dean, L.	2023	Mental health, stigma and the quality of life of people affected by neglected tropical diseases of the skin in Kasai Province, Democratic Republic of the Congo: a sex-disaggregated analysis	Int. Health
Selçukoğlu Kilimci, Ö : İşler, C. : Kara Esen, B. : Baş, G. : Özkara, Ç	2024	Psychosocial outcomes six months after epilepsy surgery: A perspective on coping strategies	Epilepsy Behav.
Seo, Kawoun	2023	The Mediating Effect of Experiential Avoidance on the Relationship between Diabetes Distress and Self-Stigma in People with Diabetes Mellitus Type 2 in Republic of Korea	Healthcare (2227-9032)
Sevincer, G. M. : Kaya, A. : Bozkurt, S. : Akin, E. : Kose, S.	2017	Reliability, validity, and factorial structure of the Turkish version of the weight self-stigma questionnaire (Turkish WSSQ)	Psychiatry and Clinical Psychopharmacology
Shafik Ibrahim Mohamed, H. : Hamad Alhulaibi, A. : Awadh Alawadh, R. : Jamaan Alanzi, A.	2023	Perceived Stress and Internalized Stigma among Patients with Mental Disorders in Al Ahsa Governorate - Saudi Arabia	International Journal of Africa Nursing Sciences
Shah, B. : Mahapatra, A. : Singh, U. N. : Mishra, V. : Daha, S. K. : Pande, R. : Neupane, M. R. : Banjade, A. : Khatik, C. B. : K. C, T. B. : Mandal, R. K. : Pokharel, S. : Gupta, R. : G. C, K. B.	2023	Internalized stigma related to COVID-19 and its psychosocial and mental health correlates: a multicentric health facility based observational study from Nepal	Frontiers in Psychiatry
Sharifi, Nader : Kohpeima Jahromi, Vahid : Zahedi, Razieh : Aram, Shabnam : Ahmadi, Maryam	2023	Social stigma and its relationship with quality of life in multiple sclerosis patients	BMC neurology
Sharma, Pawan : Shakya, Rabi : Singh, Swarndeep : Bhandari, Anup : Shakya, Rajesh : Amatya, Amit : Joshi, Chunauti : Gurung, Grisha	2022	Prevalence of anxiety and depression among people living with leprosy and its relationship with leprosy-related stigma	Indian Journal of Dermatology
Sharma, Ratanpriya : Dale, Sannisha K.	2023	Using Network Analysis to Assess the Effects of Trauma, Psychosocial, and Socioeconomic Factors on Health Outcomes Among Black Women Living with HIV	AIDS & Behavior
Shi, J. : Chen, Y. : Jiang, Y. : Li, Y. : Wang, W. : Zhao, H. : Guo, L. : Liao, Y. : Zhang, H. : Gao, C. : McIntyre, R. S. : Zhang, W. H. : Han, X. : Lu, C.	2024	Stigma and its associations with medication adherence in major depressive disorder	Psychiatry Research

Shi, X. : Sun, X. : Zhang, C. : Li, Z.	2024	Individual stigma in people with severe mental illness: Associations with public stigma, psychological capital, cognitive appraisal and coping orientations	Comprehensive Psychiatry
Shi, Y. : Dong, S. : Liang, Z. : Xie, M. : Zhang, H. : Li, S. : Li, J.	2024	Affiliate Stigma among family caregivers of individuals with dementia in China: a cross-sectional study	Frontiers in public health
Shih, Chieh-An : Huang, Jiun-Hau : Yang, Man-Hua	2022	Anti-stigma psychosocial intervention effects on reducing mental illness self-stigma and increasing self-esteem among patients with schizophrenia in Taiwan: A quasi-experiment	Asian journal of psychiatry
Sianturi, E. I. : Longe, V. S. : Arjadi, R. : Bakri, N. F. : Gunawan, E. : Sinaga, E. S.	2024	Suicide Behavior Among Indigenous and Non-Indigenous Living with HIV: A Cross-Sectional Study in Indonesia	Journal of immigrant and minority health
Sidhu, G. S. : Garg, K. : Chopra, V.	2023	Stigma and self-esteem in patients of bronchial asthma	Monaldi archives for chest disease = Archivio Monaldi per le malattie del torace
Singh, Varsha : Anupriya, : Verma, Shikha : Lata, Swaran	2023	HIV/AIDS stigma, perceived social support and mental health problems among HIV/AIDS children: a mediation analysis	Vulnerable Children & Youth Studies
Sińska, Beata I. : Kucharska, Alicja : Panczyk, Mariusz : Matejko, Bartłomiej : Traczyk, Iwona : Harton, Anna : Jaworski, Mariusz	2023	Identification of Sociodemographic and Clinical Factors Influencing the Feeling of Stigmatization in People with Type 1 Diabetes	Healthcare (2227-9032)
Sivaganesh, S. : Pathmeswaran, A.	2023	Self-stigma of leprosy affected persons in Jaffna District	Jaffna Medical Journal
So, Y. K. : Chan, C. Y. : Fung, S. C. : Lui, T. T. : Lau, F. C. : Chan, K. W. : Lee, H. M. : Lui, S. Y. : Hui, L. M. : Chen, E. : Chang, W. C.	2024	Rates and correlates of medication non-adherence behaviors and attitudes in adult patients with early psychosis	Social psychiatry and psychiatric epidemiology
Sokhela, D. G. : Orton, P. M. : Nokes, K. M. : Samuels, W. E.	2023	Exploring intersectionality and HIV stigma in persons receiving HIV care in nurse-led public clinics in Durban, South Africa	International Journal of Africa Nursing Sciences
Solokhina, Tatiana : Oshevsky, Dmitry : Barkhatova, Aleksandra : Kuzminova, Marianna : Tiumenkova, Galina : Alieva, Leyla : Shteinberg, Alisa : Churkina, Anna	2024	Self-Stigma in Patients with Endogenous Mental Disorders: A Cross-Sectional Comparative Study	Consortium psychiatricum
Solomon, Y. : Teshome, Y. : Ejigu, S. : Bezabih, M.	2023	Prevalence of anti-seizure medication nonadherence and its associated factors, among people with epilepsy in North Shewa, Ethiopia, 2021	Epilepsy Behav.
Sommers-Spijkerman, M. : Kavanaugh, M. S. : Kruitwagen-Van Reenen, E. : Zwarts-Engelbert, A. : Visser-Meily, J. M. A. : Beelen, A.	2023	Stigma experienced by ALS/PMA patients and their caregivers: a mixed-methods study	Amyotrophic Lateral Scler. Frontotemporal Degener.
Sonik-Włodarczyk, Janina : Grygiel, Paweł : Anczewska, Marta : Świtaj, Piotr	2022	How Are Various Aspects of Personal Stigma Related to Secrecy About Mental Health Problems Among People Diagnosed With Psychotic Disorders? A Cross-Sectional Analysis	Psychiatric Rehabilitation Journal
Sori, L. M. : Sema, F. D. : Tekle, M. T.	2022	Internalized stigma and associated factors among people with mental illness at University of Gondar Comprehensive Specialized Hospital, Northwest, Ethiopia, 2021	International Journal of Mental Health Systems
Sorokin, M. : Lutova, N. : Bocharova, M. : Khobeysh, M. : Wied, V.	2023	Computational Psychiatry Approach to Stigma Subtyping in Patients with Mental Disorders: Explicit and Implicit Internalized Stigma	Consort. Psychiatr.
Sorokin, Mikhail Yu : Neznanov, Nikolay G. : Lutova, Natalia B. : Wied, Viktor D.	2021	Revisiting Drug Compliance: The Need for a Holistic Approach in the Treatment of Severe Mental Disorders	Consortium psychiatricum
Steinhauser, S. : Haroz, R. : Jones, I. : Skelton, W. : Fuller, B. M. : Roberts, M. B. : Jones, C. W. : Trzeciak, S. : Roberts, B. W.	2024	Emergency department staff compassion is associated with lower fear of enacted stigma among patients with opioid use disorder	Academic Emergency Medicine
Stopic, V. : Jost, S. T. : Baldermann, J. C. : Petry-Schmelzer, J. N. : Fink, G. R. : Dembek, T. A. : Dafsari, H. S. : Kessler, J. : Barbe, M. T. : Sauerbier, A.	2023	Parkinson's Disease Stigma Questionnaire (PDStigmaQuest): Development and Pilot Study of a Questionnaire for Stigma in Patients with Idiopathic Parkinson's Disease	Journal of Parkinson's Disease
Stringer, Kristi Lynn : Norcini Pala, Andrea : Cook, Robert L. : Kempf, Mirjam-Colette : Konkle-Parker, Deborah : Wilson, Tracey E. : Tien, Phyllis C. : Wingood, Gina : Neilands, Torsten B. : Johnson, Mallory O. : Logie, Carmen H. : Weiser, Sheri D. : Turan, Janet M. : Turan, Bulent	2024	Intersectional Stigma, Fear of Negative Evaluation, Depression, and ART Adherence Among Women Living with HIV Who Engage in Substance Use: A Latent Class Serial Mediation Analysis	AIDS & Behavior
Styła, R. : Świtaj, P.	2024	Time perspective and self-stigma in schizophrenia	Journal of Mental Health
Sugisawa, Hidehiro : Shimizu, Yumiko : Kumagai, Tamaki : Shishido, Kanji : Koda, Yutaka : Shinoda, Toshio	2023	Influence of dialysis-related stigma on health-related indicators in Japanese patients undergoing hemodialysis	Therapeutic apheresis and dialysis : official peer-reviewed journal of the International Society for Apheresis, the Japanese Society for Apheresis, the Japanese Society for Dialysis Therapy
Sukut, Ö : Buzlu, S.	2022	Psychometric Properties of the Turkish Version of the Substance Abuse Self-Stigma Scale	Addict. Turk. J.
Szepietowska, M. : Stefaniak, A. A. : Krajewski, P. K. : Matusiak, L.	2024	Females May Have Less Severe Acne, but They Suffer More: A Prospective Cross-Sectional Study on Psychosocial Consequences in 104 Consecutive Polish Acne Patients	J. Clin. Med.
Tabas, M. Y. : Momeni, F. : Bakhshani, N. M. : Pourshahbaz, A. : Rezaei, O.	2023	Effectiveness of Family-Focused Therapy in Bipolar Disorder: A Randomized Controlled Trial	Iran. J. Psychiatr. Behav. Sci.
Taher, Taqi M. J. : Ahmed, Hussein A. : Abutiheen, Ali A. : Alfadhul, Shaymaa A. : Ghazi, Hasanain F.	2023	Stigma perception and determinants among patients with type 2 diabetes mellitus in Iraq	The Journal of the Egyptian Public Health Association
Talibov, Tural : Inci, Meltem : Ismayilov, Rashad : Elmas, Sibel : Buyuktopcu, Emiralp : Kepenek, Ata Onur : Sirin, Gorkem : Polat, Irmak : Ozkan, Mine : Bebek, Nerses	2024	The relationship of psychiatric comorbidities and symptoms, quality of life, and stigmatization in patients with epilepsy	Epilepsy & behavior : E&B
Tam, C. : Wang, L. : Salters, K. : Moore, D. : Wesseling, T. : Grieve, S. : Parry, R. : Barath, J. : Hogg, R. : Barrios, R.	2024	Evaluating experiences of HIV-related stigma among people living with HIV diagnosed in different treatment eras in British Columbia, Canada	AIDS Care - Psychological and Socio-Medical Aspects of AIDS/HIV
Tandiawan, F. : Gunadharma, S. : Gamayani, U. : Ong, A. : Ganiem, A. R. : Iskandar, S.	2023	Depression as comorbidity in Sundanese epilepsy patients	Neurol. Asia
Tang, Lichen : Lin, Zheng	2024	The Mediating and Moderating Role of Resilience Between Stigma and Illness Identity Among People with Inflammatory Bowel Disease	Psychology research and behavior management
Tang, Ming-Wen : Cheng, Yin : Zhang, Yan-Hong : Liu, Shou-Juan	2023	Effect of a Positive Psychology Expressive Writing on Stigma, Hope, Coping Style, and Quality of Life in Hospitalized Female Patients with Schizophrenia: A Randomized, Controlled Trial	Perspectives in Psychiatric Care
Tang, Xuan : Chen, Shuang-Qin : Huang, Jiang-Hua : Deng, Cai-Feng : Zou, Jie-Qiong : Zuo, Juan	2024	Assessing the current situation and the influencing factors affecting perceived stigma among older patients after leukemia diagnosis	World journal of psychiatry
Techapoonpon, K. : Kerdchareon, N. : Polruamngern, N. : Chalermrungroj, T. : Srikhamdokkhae, O. : Matangkarat, P.	2023	Stigma Experienced by Patients Who Recovered from COVID-19 in Post-Vaccination Period in Thailand; Prevalence and Associated Factors	Psychol. Res. Behav. Manage.
Telaak, Sydney H. : Costabile, Kristi A. : Persky, Susan	2023	The influence of weight on psychosocial well-being in diabetes	BMC psychology
Thapa, D. K. : Dahal, H. R. : Chaulagain, D. R. : Karki, A. : Sharma, N. : Baral, B. : Nepal, C. : Paudel, P. : Thapa, J.	2023	Stigma, depression and quality of life among people affected by neglected tropical diseases in Nepal	Int. Health
Thomford, N. E. : Dampson, F. N. : Adjei, G. : Eliason, S. : Ekor, M. : Kyei, G. B.	2023	Psychometric assessment of HIV stigma in patients attending a tertiary facility: An initial validation of the Berger HIV stigma scale in a Ghanaian perspective	PLoS ONE
Tomaz Santos, Nuno : Ramos, Catarina : de Almeida, Margarida Ferreira : Leal, Isabel	2024	Group Intervention Program to Facilitate Post-Traumatic Growth and Reduce Stigma in HIV	Healthcare (2227-9032)
Tombini, M. : Narducci, F. : Ricci, L. : Sancetta, B. : Boscarino, M. : Quintiliani, L. : Lanzone, J. : Straffi, M. : Di Lazzaro, V. : Assenza, G.	2024	Resilience and psychosocial factors in adult with epilepsy: A longitudinal study	Epilepsy & behavior : E&B
Topi, Maria : Mangoulia, Polyxeni : Koukia, Evmorfia	2023	Adaption and Construct Validity in the Greek Context of the Appraisal of Self-Care Agency Scale -Revised (ASAS - R scale)	International Journal of Caring Sciences
Tshuma, N. : Ngbede, E. D. : Nyengerai, T. : Mtapuri, O. : Moyo, S. : Mphuthi, D. D. : Nyasulu, P.	2023	Understanding health outcome drivers among adherence club patients in clinics of Gauteng, South Africa: a structural equation modelling (SEM) approach	AIDS Research and Therapy
Tu, Chao-Ying : Liu, Wei-Shih : Huang, Wei-Lieh	2023	Associations of internalized stigma with psychosocial functioning and quality of life in people with schizophrenia	The International journal of social psychiatry
Tuot, S. : Sim, J. W. : Nagashima-Hayashi, M. : Chhoun, P. : Teo, A. K. J. : Prem, K. : Yi, S.	2023	What are the determinants of antiretroviral therapy adherence among stable people living with HIV? A cross-sectional study in Cambodia	AIDS Research and Therapy
Turan, B. : Budhwani, H. : Yigit, I. : Ofotokun, I. : Konkle-Parker, D. J. : Cohen, M. H. : Wingood, G. M. : Metsch, L. R. : Adimora, A. A. : Taylor, T. N. : Wilson, T. E. : Weiser, S. D. : Kempf, M. C. : Brown-Friday, J. : Gange, S. : Kassaye, S. : Pence, B. W. : Turan, J. M.	2022	Resilience and Optimism as Moderators of the Negative Effects of Stigma on Women Living with HIV	AIDS Patient Care and STDs
Turen, S. : Yurtsever, M.	2024	Stigma and its relationship with life satisfaction in patients with type 2 diabetes mellitus	Eur. J. Clin. Exp. Med.
Türk, A. : Uğurlu, N. B.	2023	Internalized stigma and the quality of life and self-esteem of individuals with bipolar disorder	J. Psyc. Nurs.
Tworek, G. : Thompson, N. R. : Kane, A. : Sullivan, A. B.	2023	The impact of stigma on perceived quality of life and experience of anxiety and depression in individuals diagnosed with MS	Multiple Sclerosis and Related Disorders
Uçan Tokuç, F. E. : Göksu, E. Ö : Katı, Ş D.	2024	Internal stigmatization in patients with chronic migraine and medication overuse headache	BMC Neurology

Umar, Muhammad : Basit, Anila : Ali, Sher : Iqbal, Zafar	2022	Understanding the Burden: Prevalence and Predictors of Depression and Anxiety among Multidrug-Resistant Tuberculosis Patients and their Household Contacts	Pakistan Journal of Chest Medicine
Valery, Kevin-Marc : Fournier, Thomas : Violeau, Louis : Guionnet, Sarah : Bonilla-Guerrero, Julien : Caria, Aude : Carrier, Antonin : Destaillats, Jean-Marc : Follenfant, Alice : Laberon, Sonia : Lalbin-Wander, Nadeja : Martinez, Eric : Quemper, Danielle : Staedel, Bérénice : Touroude, Roselyne : Vigneault, Luc : Prouteau, Antoinette	2023	When Mental Health Care Is Stigmatizing: A Participative Study in Schizophrenia	Psychiatric Rehabilitation Journal
van den Berg, K. : Murphy, E. L. : Louw, V. J. : Maartens, G. : Hughes, S. D.	2023	Motivations for blood donation by HIV-positive individuals on antiretrovirals in South Africa: A qualitative study	Transfusion Medicine
Van Loey, Nancy E. E. : Boersma-van Dam, Elise : Boekelaar, Anita : van de Steenoven, Anneke : de Jong, Alette E. E. : Hofland, Helma W. C.	2024	Development and Testing of the Aftercare Problem List, a Burn Aftercare Screening Instrument	European Burn Journal (EBJ)
Vasiliou, V. S. : Russell, H. : Cockayne, S. : Coelho, G. L. D. H. : Thompson, A. R.	2023	A network analysis of psychological flexibility, coping, and stigma in dermatology patients	Frontiers in Medicine
Vazquez Morejon, Antonio J. : Jackson, Chris : Vazquez-Morejon, Raquel : Leon-Perez, Jose M.	2022	Personal Beliefs about Illness Questionnaire-Revised (PBIQ-R): Spanish adaptation in a clinical sample with psychotic disorders	Behavioural and cognitive psychotherapy
Ventura, Carla Aparecida Arena : Carrara, Bruna Sordi : da Silva, Felicialle Pereira : Vedana, Kelly Graziani Giacchero : dos Reis, Leonardo Naves : Vidigal, Brenda Alice : de Moura, Ana Luiza Martins	2023	Factors associated with internalized stigma in people with common mental disorders using services of Family Health Strategy	Archives of Psychiatric Nursing
Verinumbe, Tarfa : Katomski, Anna-Sophia : Turpin, Gnilane : Syarif, Omar : Looze, Pim : Lalak, Katarzyna : Anoubissi, Jean : Brion, Sophie : Dunaway, Keren : Sprague, Laurel : Matyushina, Daria : De Leon Moreno, Carlos Garcia : Baral, Stefan D. : Rucinski, Katherine : Lyons, Carrie	2024	Characterizing the Relationship between HIV Peer Support Groups and Internalized Stigma Among People Living with HIV in Nigeria	AIDS & Behavior
Villarejo-Galende, A. : García-Arcelay, E. : Piñol-Ripoll, G. : Del Olmo-Rodríguez, A. : Viñuela, F. : Boada, M. : Franco-Macías, E. : Ibañez De La Peña, A. : Riverol, M. : Puig-Pijoan, A. : Abizanda-Soler, P. : Arroyo, R. : Baquero-Toledo, M. : Feria-Vilar, I. : Balasa, M. : Berbel, Á : Rodríguez-Rodríguez, E. : Vieira-Campos, A. : García-Ribas, G. : Rodrigo-Herrero, S. : Terrancle, Á : Prefasi, D. : Lleó, A. : Maurino, J. : Clements-Cortes, A.	2022	Quality of Life and the Experience of Living with Early-Stage Alzheimer's Disease	J. Alzheimer's Dis.
Viscardi, L. H. : Rosa, V. : Vilanova, F. : Grevet, E. H. : Dotto-Bau, C. H. : Rodrígues, M. I. : Cavalcante-Passos, I. : Costa, A. B.	2024	The association between personal stigma and adherence to treatment in patients diagnosed with bipolar mood disorder, attention deficit/hyperactivity disorder and schizophrenia	Rev. Psicopatol. Psicol. Clin.
Viteva, E.	2016	Relation of Perceived Stigma to Adverse Events of Medications in Patients with Epilepsy	Epilepsy Research and Treatment
Vizin, G. : Szekeres, T. : Juhász, A. : Márton, L. : Dank, M. : Perczel-Forintos, D. : Urbán, R.	2023	The role of stigma and depression in the reduced adherence among young breast cancer patients in Hungary	BMC psychology
Vogel, J. S. : Bruins, J. : Swart, M. : Liemburg, E. : van der Gaag, M. : Castelein, S.	2023	Effects of an eating club for people with a psychotic disorder on personal recovery: Results of a randomized controlled trial	Journal of Behavior Therapy and Experimental Psychiatry
Vrbova, K. : Prasko, J. : Holubova, M. : Slepecky, M. : Ociskova, M.	2018	Positive and negative symptoms in schizophrenia and their relation to depression, anxiety, hope, self-stigma and personality traits - A cross-sectional study	Neuroendocrinology Letters
Walsh, J. L. : Quinn, K. G. : Hirshfield, S. : John, S. A. : Algiers, O. : Al-Shalby, K. : Giuca, A. M. : McCarthy, C. : Petroll, A. E.	2024	Acceptability, Feasibility, and Preliminary Impact of 4 Remotely-Delivered Interventions for Rural Older Adults Living with HIV	AIDS and behavior
Wang, D. : Zhou, Y. : Chen, S. : Wu, Q. : He, L. : Wang, Q. : Hao, Y. : Liu, Y. : Peng, P. : Li, M. : Liu, T. : Ma, Y.	2024	Employing Bayesian analysis to establish a cut-off point and assess stigma prevalence in substance use disorder: a comprehensive study of the Chinese version of the Substance Use Stigma Mechanism Scale	Social psychiatry and psychiatric epidemiology
Wang, Jie-yu : Luo, Gui-ying : Lv, Xiao-qing : Liang, Chun-mei : Wang, Dan-ni : Li, Guan-jian : Hong, Jing-fang : Cao, Yun-xia	2023	Resilience of infertile families undergoing in vitro fertilization: An application of the double ABC-X model	Applied Nursing Research
Wang, Qian-Qian : Cheng, Li : Wu, Bi-Yu : Qiu, Hong-Yi : Xu, Ping : Wang, Bo : Yan, Xiu-Juan : Chen, Sheng-Liang	2023	Stigma and Efficacy of Zhizhu Kuanzhong Capsules Versus Doxepin in the Treatment of Refractory Functional Dyspepsia: A Randomized Controlled Trial	Journal of neurogastroenterology and motility
Wang, R. : Zheng, S. : Ouyang, X. : Zhang, S. : Ge, M. : Yang, M. : Sheng, X. : Yang, K. : Xia, L. : Zhou, X.	2023	Suicidality and Its Association with Stigma in Clinically Stable Patients with Schizophrenia in Rural China	Psychol. Res. Behav. Manage.
Wang, S. : Deng, Y. : Zhang, Y. : Guo, V. Y. : Zhang, B. : Cheng, X. : Xin, M. : Hao, Y. : Hou, F. : Li, J.	2023	The role of illness-related cognition in the relationships between resilience and depression/anxiety in nasopharyngeal cancer patients	Cancer Medicine
Wang, X. : Ye, J. : Tian, X. : Wang, F. : Guo, X.	2024	Affiliate stigma and caregiver burden in parents of children with epilepsy	Preventive Medicine Reports
Wardojo, S. S. I. : Rosadi, R.	2023	Associations between social support, resilience, HIV stigma, and depression among people living with HIV in Malang, Indonesia: A cross-sectional study	Russian Open Medical Journal
Weinstein, E. R. : Mendez, N. A. : Jones, M. A. : Safren, S. A.	2024	The impact of syndemic burden, age, and sexual minority status on internalized HIV stigma among people living with HIV in South Florida	Journal of health psychology
Weiser, S. D. : Sheira, L. A. : Weke, E. : Zakaras, J. M. : Wekesa, P. : Frongillo, E. A. : Burger, R. L. : Mocello, A. R. : Thirumurthy, H. : Dworkin, S. L. : Tsai, A. C. : Kahn, J. G. : Butler, L. M. : Bukusi, E. A. : Cohen, C. R.	2024	An agricultural livelihood intervention is associated with reduced HIV stigma among people with HIV	AIDS
Wendeln, Beatrice Meda : Sheehan, Lindsay	2023	Exploratory study of an ally training program for mental health support	Journal of Public Mental Health
Wiis, M. A. K. : Heilskov, S. : Sørensen, J. : Bang, K. : Egekvist, H. : Deleuran, M. : Thyssen, J. P. : Thomsen, S. F. : Vestergaard, C.	2022	Self-reported stigmatisation among patients with atopic dermatitis (AD) or chronic spontaneous urticaria (CSU): A cross-sectional study	JEADV Clin. Pract.
Williamson, T. J. : Garon, E. B. : Irwin, M. R. : Choi, A. K. : Goldman, J. W. : Stanton, A. L.	2024	Sleep Disturbance as a Mediator of Lung Cancer Stigma on Psychological Distress and Physical Symptom Burden	Psychosomatic Medicine
Wu, X. : Hu, Y. : Li, Y. : Li, S. : Li, H. : Ye, X. : Hu, A.	2024	Stigma and self-esteem in facial burn patients: A correlation study	Burns
Wu, Yi-jin : Dai, Zhen-wei : Xiao, Wei-jun : Wang, Hao : Huang, Yi-man : Si, Ming-yu : Fu, Jia-qi : Chen, Xu : Jia, Meng-meng : Leng, Zhi-wei : Cui, Dan : Mak, Winnie W. S. : Su, Xiao-you	2023	Perceived stigma among discharged patients of COVID-19 in Wuhan, China: a latent profile analysis	Frontiers in Public Health
Wu, Yu : Yan, Zeping : Fornah, Lovel : Zhao, Jun : Wu, Shicai	2023	The mediation effect of social support between stigma and social alienation in patients with stroke	Frontiers in public health
Xi, Z. : Rong, C. M. : Ling, L. J. : Hua, Z. P. : Rui, G. : Fang, H. G. : Long, W. : Zhen, Z. H. : Hong, L.	2022	The influence of stigma and disability acceptance on psychosocial adaptation in patients with stoma: A multicenter cross-sectional study	Front. Psychol.
Xiao, Wei-jun : Liu, Xiao-yang : Wang, Hao : Huang, Yi-man : Dai, Zhen-wei : Si, Ming-yu : Fu, Jia-qi : Chen, Xu : Jia, Meng-meng : Leng, Zhi-wei : Cui, Dan : Mak, Winnie W. S. : Dong, Li-ming : Su, Xiao-you	2023	Mediating role of resilience in the relationship between COVID-19 related stigma and mental health among COVID-19 survivors: a cross-sectional study	Infectious Diseases of Poverty
Xing, Shuping : Liu, Yeling : Zhang, Hua : Li, Bin : Jiang, Xinjun	2023	The mediating role of diabetes stigma and self-efficacy in relieving diabetes distress among patients with type 2 diabetes mellitus: a multicenter cross-sectional study	Frontiers in psychology
Xing, Yanqing : Zhao, Wenxiao : Duan, Chenchen : Zheng, Jun : Zhao, Xuelian : Yang, Jingyu : Sun, Na : Chen, Jie	2023	Developing a visual model for predicting depression in patients with lung cancer	Journal of Clinical Nursing (John Wiley & Sons, Inc.)
Xu, Guangyi : Liu, Ting : Jiang, Yunxia : Xu, Yanhong : Zheng, Taohua : Li, Xiaona	2024	Heterogeneity in Psychological Adaptation Patterns and Its Predictive Factors Among Patients with Inflammatory Bowel Disease: A Latent Profile Analysis	Psychology research and behavior management
Xue, H. : Wang, C. : Tian, Y. : Guo, Z. : Zhang, C. : Liu, L. : An, C. : Zhang, L. : Niu, S. : Cao, J. : Di, Y. : Li, N.	2024	The correlation between cumulative burden of mental disorders and self-harm, shame, and insight in young female patients with schizophrenia	European Archives of Psychiatry and Clinical Neuroscience
Yadav, J. : Mishra, S. : Nischal, A. : Prakash, A. J.	2023	Relationship between stigma, self-esteem and quality of life among patients with depression at a tertiary care unit of North India: a cross-sectional study	Minerva Psychiatry
Yan, F. : Wu, K. : Wan, Q. : Zhang, M. : Zhang, Y. : Li, N. : Wang, X.	2023	Assessing the effectiveness of biofeedback therapy in the rehabilitation of limb motor dysfunction after stroke and the influencing factors of disease-related shame	American Journal of Translational Research
Yani, D. I. : Juniarti, N. : Lukman, M.	2022	Factors Related to Complying with Anti-TB Medications Among Drug-Resistant Tuberculosis Patients in Indonesia	Patient Preference and Adherence
Ye, Fei : Wu, YouHong	2023	Impacts of self-confidence cultivation combined with family collaborative nursing on the hope level, stigma and exercise tolerance in patients undergoing radical resection of pulmonary carcinoma	Frontiers in surgery
Yenealem, B. : Negash, M. : Madoro, D. : Molla, A. : Nenko, G. : Nakie, G. : Getnet, B.	2024	Prevalence and associated factors of maternal depression among mothers of children with undernutrition at comprehensive specialized hospitals in Northwest Ethiopia in 2023: a cross–sectional study	Frontiers in Psychiatry
Yeni, K.	2023	Anticipated Stigma and Related Factors in Patients with Chronic Diseases	Arch. Health. Sci Res.
Yeni, K. : Ozdelikara, A. : Terzi, M.	2023	Relationship between stigma and symptom burden in patients with multiple sclerosis	Neurology Asia
Yeni, K. : Terzi, M.	2023	Relationship of personality traits with stigmatization, depression, and quality of life in patients with multiple sclerosis	Neurology Asia

Yeni, K. : Tulek, Z. : Cavusoglu, A. : Dunya, C. P. : Erden, S. O. : Bostan, N. S. : Bebek, N. : Baykan, B.	2024	Caregiver burden and its predictors in adult epilepsy patients	Epilepsy and Behavior
Yesuf, W. : Hiko, D. : Alemayehu, E. : Kusheta, S. : Shita, A. : Beyene, M.	2024	Health-related quality of life in epilepsy and its associated factors among adult patients with epilepsy attending Mizan Tepi University Teaching Hospital, Southwest Ethiopia: a cross-sectional study	BMJ Open
Yeung, N. C. Y. : Lee, E. K. P. : Kong, A. P. S. : Leung, M. K. W.	2024	"Shame on Me": Exploring the Role of Self-Stigma in Psychological Outcomes Among Type 2 Diabetes Patients in Hong Kong	International journal of behavioral medicine
Yi-Ling, Hu : Chih-Cheng, Chang : Chiu-Hsiang, Lee : Chieh-Hsiu, Liu : Yi-Jung, Chen : Jian-An, Su : Chung-Ying, Lin : Griffiths, Mark D.	2023	Associations between Affiliate Stigma and Quality of Life among Caregivers of Individuals with Dementia: Mediated Roles of Caregiving Burden and Psychological Distress	Asian Journal of Social Health & Behavior
Yigit, I. : Paulino-Ramírez, R. : Waters, J. : Long, D. M. : Turan, J. M. : Budhwani, H.	2024	A Moderated Mediation Analysis of HIV and Intersectional Stigmas and Antiretroviral Adherence in People Living with HIV in the Dominican Republic	AIDS and behavior
Yigit, I. : Turan, B. : Kurt, G. : Weiser, S. D. : Johnson, M. O. : Mugavero, M. J. : Turan, J. M.	2024	Longitudinal Associations of Experienced and Perceived Community Stigma With Antiretroviral Therapy Adherence and Viral Suppression in New-to-Care People With HIV: Mediating Roles of Internalized Stigma and Depression Symptoms	J. Acquired Immune Defic. Syndr.
Yildiz, E. : Iwayama, T. : Kuramochi, I.	2024	Validity and reliability study of the turkish version epilepsy self-stigma scale (ESSS-T)	Epilepsy and Behavior
Yip, C. C. H. : Fung, W. T. W. : Leung, D. C. K. : Chan, K. K. S.	2023	The impact of stigma on engaged living and life satisfaction among people with mental illness in Hong Kong	Quality of Life Research
Yoo-Jeong, M. : Nguyen, A. L.	2024	Combined effects of social isolation and loneliness on patient-reported outcomes in older adults with HIV	AIDS Care - Psychological and Socio-Medical Aspects of AIDS/HIV
Young, C. A. : Rog, D. J. : Tanasescu, R. : Kalra, S. : Langdon, D. : Tennant, A. : Mills, R. J.	2023	Multiple Sclerosis vision questionnaire (MSVQ-7): Reliability, validity, precision and discrimination	Multiple Sclerosis and Related Disorders
Younossi, Z. M. : Alqahtani, S. A. : Alswat, K. : Yilmaz, Y. : Keklikkiran, C. : Funuyet-Salas, J. : Romero-Gómez, M. : Fan, J. G. : Zheng, M. H. : El-Kassas, M. : Castera, L. : Liu, C. J. : Wai-Sun Wong, V. : Zelber-Sagi, S. : Allen, A. M. : Lam, B. : Treeprasertsuk, S. : Hameed, S. : Takahashi, H. : Kawaguchi, T. : Schattenberg, J. M. : Duseja, A. : Newsome, P. N. : Francque, S. : Spearman, C. W. : Castellanos Fernández, M. I. : Burra, P. : Roberts, S. K. : Chan, W. K. : Arrese, M. : Silva, M. : Rinella, M. : Singal, A. K. : Gordon, S. : Fuchs, M. : Alkhouri, N. : Cusi, K. : Loomba, R. : Ranagan, J. : Eskridge, W. : Kautz, A. : Ong, J. P. : Kugelmas, M. : Eguchi, Y. : Diago, M. : Yu, M. L. : Gerber, L. : Fornaresio, L. : Nader, F. : Henry, L. : Racila, A. : Golabi, P. : Stepanova, M. : Carrieri, P. : Lazarus, J. V.	2024	Global survey of stigma among physicians and patients with nonalcoholic fatty liver disease	Journal of Hepatology
Younossi, Z. M. : AlQahtani, S. A. : Funuyet-Salas, J. : Romero-Gómez, M. : Yilmaz, Y. : Keklikkiran, C. : Alswat, K. : Yu, M. L. : Liu, C. J. : Fan, J. G. : Zheng, M. H. : Burra, P. : Francque, S. M. : Castera, L. : Schattenberg, J. M. : Newsome, P. N. : Allen, A. M. : El-Kassas, M. : Treeprasertsuk, S. : Hameed, S. : Wai-Sun Wong, V. : Zelber-Sagi, S. : Takahashi, H. : Kawaguchi, T. : Castellanos Fernández, M. I. : Duseja, A. : Arrese, M. : Rinella, M. : Singal, A. K. : Gordon, S. C. : Fuchs, M. : Eskridge, W. : Alkhouri, N. : Cusi, K. : Loomba, R. : Ranagan, J. : Kautz, A. : Ong, J. P. : Kugelmas, M. : Eguchi, Y. : Diago, M. : Gerber, L. : Lam, B. : Fornaresio, L. : Nader, F. : Spearman, C. W. : Roberts, S. K. : Chan, W. K. : Silva, M. : Racila, A. : Golabi, P. : Ananchuensook, P. : Henry, L. : Stepanova, M. : Carrieri, P. : Lazarus, J. V.	2024	The impact of stigma on quality of life and liver disease burden among patients with nonalcoholic fatty liver disease	JHEP Reports
Yousefi, Majid : Narafshan, Mehry Haddad : Tajadini, Massoud	2022	English language learning in patients suffering from mental disorders: A different concept of self-stigma	Research & Development in Medical Education
Yu, Yong : Qin, Keke : Xiao, Fuqun : Cai, Huilin : Sun, Yinmeng : Li, Jiahong : Cao, Zhi : Yang, Kun : Huang, Qianyue : Wei, Ziyu	2023	HIV Disclosure Among Sexually Infected People Living with HIV and AIDS in China: Prevalence, Influencing Factors, and Negative Outcomes	AIDS & Behavior
Yuan, Guangzhe Frank : Qiao, Shan : Li, Xiaoming : Zhou, Yuejiao : Shen, Zhiyong	2024	Psychological Mechanisms of Internalized HIV Stigma Affect Sleep Impairment among People Living with HIV in China: A follow-up Study	AIDS & Behavior
Yuan, Guangzhe Frank : Zhang, Ran : Qiao, Shan : Li, Xiaoming : Shen, Zhiyong : Zhou, Yuejiao	2024	Exploring the Longitudinal Influence of Perceived Social Support, HIV Stigma, and Future Orientation on Depressive Symptoms Among People Living with HIV in China	AIDS & Behavior
Yuksel, Ruveyda : Arslantas, Hulya : Dereboy, Ferhan : Kizilkaya, Mehtap : Inalkac, Seher	2023	Validity and Reliability Study of Mental Health Recovery Measure's Turkish Version	Ruh Sagligi Iyilesme Olcegi'nin Turkce Formunun Gecerlik ve Guvenirlik Calismasi.
Yunhe, Huang : Trollor, Julian N. : Foley, Kitty-Rose : Arnold, Samuel R. C.	2023	"I've Spent My Whole Life Striving to Be Normal": Internalized Stigma and Perceived Impact of Diagnosis in Autistic Adults	Autism in Adulthood
Zahra-Zeitoun, Y. : Elran-Barak, R. : Salameh-Dakwar, R. : Froylich, D. : Sroka, G. : Assalia, A. : Latzer, Y.	2024	Weight stigma in healthcare settings: the experience of Arab and Jewish bariatric surgery candidates in Israel	Israel Journal of Health Policy Research
Zarbah, Abdulmajeed A. : Al Alfard, Hayfa A. : Alamri, Hassan S. : Al Edrees, Nada : Alshahrani, Nouf S. : Alshehri, Ali F.	2023	Prevalence of internalized stigma in patients with psychiatric illness in Abha, Southern Region, Saudi Arabia	Journal of family & community medicine
Zhang, C. : Xu, M. : Yu, H. : Hua, Y. : Wang, X. : Nan, X. : Zhang, J.	2024	Relationships Among Demographic Factors, Stigma, Social Support, and Self-Management in Individuals With Bipolar Disorder in Remission	Journal of psychosocial nursing and mental health services
Zhang, E. : Wu, T. : Zhang, Y. : Wan, W. : Chong, W. : Qin, X. : Wu, J. : Che, X.	2023	A comparative study of stigma and mental health status between patients with erectile dysfunction and patients with erectile dysfunction and diabetes	Journal of Men's Health
Zhang, J. : Ding, L. : Wu, Y. : Yao, M. : Ma, Q.	2023	Perceived stigma in burn survivors: Associations with resourcefulness and alexithymia	Burns
Zhang, L. : Zhu, W. : Ye, J.	2024	Psychological status is associated with the perceived illness stigma in Chinese systemic lupus erythematosus patients	Psychology, health & medicine
Zhang, Qi-wen : Deng, Jing : Luo, Huan-yue : Wang, Li	2023	Senile dementia and psychiatric stigma among community health service providers and relatives of diagnosed and suspected dementia patients: a cross-sectional study	PeerJ
Zhang, Y. : Chai, C. : Xiong, J. : Zhang, L. : Zheng, J. : Ning, Z. : Wang, Y.	2023	The impact of anxiety, depression, and social support on the relationship between HIV-related stigma and mental health-related quality of life among Chinese patients: a cross-sectional, moderate-mediation study	BMC Psychiatry
Zheng, S. : Wang, R. : Zhang, S. : Ou, Y. : Sheng, X. : Yang, M. : Ge, M. : Xia, L. : Li, J. : Zhou, X.	2023	Depression severity mediates stigma and quality of life in clinically stable people with schizophrenia in rural China	BMC Psychiatry
Zheng, Z. : Song, R. : Zhao, Y. : Lv, H. : Wang, Y. : Yu, C.	2023	An investigation of the level of stigma and the factors influencing it in the rehabilitation of young and middle-aged stroke patients-a cross-sectional study	BMC Neurology
Zhou, C. : Li, H. : Zhang, Z. : Li, S. : Wu, S. : Dai, Y. : Zhang, X. : Lai, H. : Zhou, Q. : Yang, M. : Zuo, X. : Ning, J. : Wu, J. : Zheng, T. : Li, N. : Liu, L. : Zou, X. : Liu, L. : Luo, X. : Yang, J. : Wang, Y. : Zheng, L. : Luo, Y. : Chen, Y. : Deng, H.	2024	Effectiveness of video-based psychiatric rehabilitation for patients with early-phase schizophrenia spectrum: A randomized controlled trial	Early Intervention in Psychiatry
