# Supplementary material for: Stigma measurement in health: a systematic review
Source: eClinicalMedicine. 2025 Jul 24;86:103360. doi: 10.1016/j.eclinm.2025.103360 (PMC12311962; doi:10.1016/j.eclinm.2025.103360)
Supplement: Appendix C [file mmc3.docx]

Narrative Depictions of Documented Interventions to Reduce Stigma

| **Types of interventions** | **Description** |
| --- | --- |
| Psychosocial | Interventions that are psychologically based. |
| Medication/Pharmacological | Interventions that are medication based. |
| Device | Interventions that include implanted or wearable devices. |
| Surgical | Interventions that are surgical procedures. |
| Nutrition | Interventions that include nutrition advice or plans. |
| Exercise | Interventions that include a prescribed individual or group exercise. |
| Psychoeducation | Intervention that is a structured educational format for individual or group participants. |
| Cosmetic | Intervention that is a wearable cosmetic. |
| Procedural | Medical procedures that are not surgical, such as wound debridement, blood testing, etc. |
| Music | Interventions that provide music therapy. |
| Care model | Interventions that are directly related to plan of care or modality of care including policy and practice change and nursing care models. |
| Financial | Interventions that provide financial assistance. |
