# Supplementary material for: Stigma measurement in health: a systematic review
Source: eClinicalMedicine. 2025 Jul 24;86:103360. doi: 10.1016/j.eclinm.2025.103360 (PMC12311962; doi:10.1016/j.eclinm.2025.103360)
Supplement: Authorship Team [file mmc4.docx]

RESPECT Authorship Team:

Shayla Lawrence, MPH

Department of Global Pediatric Medicine, St. Jude Children’s Research Hospital, Memphis, TN

Jocelyn Rivera, MD, MS

Department of Global Pediatric Medicine, St. Jude Children’s Research Hospital, Memphis, TN

Farris Abutineh, BS

University of Tennessee Health Sciences Center

Memphis, TN

Doris Maldonado, PhD, MA

Department of Global Pediatric Medicine, St. Jude Children’s Research Hospital, Memphis, TN
